# Profiling molecular factors associated with pyknosis and developmental arrest induced by an opioid receptor antagonist and dihydroartemisinin in *Plasmodium falciparum*

**DOI:** 10.1371/journal.pone.0184874

**Published:** 2017-09-21

**Authors:** Hiroko Asahi, Shin-Ichi Inoue, Mamoru Niikura, Keisuke Kunigo, Yutaka Suzuki, Fumie Kobayashi, Fujiro Sendo

**Affiliations:** 1 Department of Infectious Diseases, Division of Tropical Diseases and Parasitology, Kyorin University School of Medicine, Tokyo, Japan; 2 Department of Computational Biology and Medical Sciences, Graduate School of Frontier Sciences, The University of Tokyo, Chiba, Japan; 3 Miyuki no Oka, Geriatric Health Service Facilities, Yamagata, Japan; Institut national de la santé et de la recherche médicale - Institut Cochin, FRANCE

## Abstract

Malaria continues to be a devastating disease, largely caused by *Plasmodium falciparum* infection. We investigated the effects of opioid and cannabinoid receptor antagonists on the growth of intraerythrocytic *P*. *falciparum*. The delta opioid receptor antagonist 7-benzylidenenaltrexone (BNTX) and the cannabinoid receptor antagonists rimonaband and SR144528 caused growth arrest of the parasite. Notably BNTX and the established antimalarial drug dihydroartemisinin induced prominent pyknosis in parasite cells after a short period of incubation. We compared genome-wide transcriptome profiles in *P*. *falciparum* with different degrees of pyknosis in response to drug treatment, and identified 11 transcripts potentially associated with the evoking of pyknosis, of which three, including glutathione reductase (PfGR), triose phosphate transporter (PfoTPT), and a conserved *Plasmodium* membrane protein, showed markedly different gene expression levels in accordance with the degree of pyknosis. Furthermore, the use of specific inhibitors confirmed PfGR but not PfoTPT as a possible factor contributing to the development of pyknosis. A reduction in total glutathione levels was also detected in association with increased pyknosis. These results further our understanding of the mechanisms responsible for *P*. *falciparum* development and the antimalarial activity of dihydroartemisinin, and provide useful information for the development of novel antimalarial agents.

## Introduction

Malaria is one of the world’s most devastating diseases, particularly in the tropics, with an estimated global annual incidence of 212 million clinical cases and mortality of 429,000 in 2015 [1], largely due to *Plasmodium falciparum* infection.

The rapid emergence of drug-resistant *Plasmodium* strains has severely reduced the therapeutic efficacy of conventional antimalarial drugs and threatens the effectiveness of artemisinin (ART) combination therapy, which is currently used widely in the field [2–5]. In humans, the *P*. *falciparum* parasite lives mainly within red blood cells (RBCs) and develops through three distinct stages (ring, trophozoite, and schizont) during its cycle lasting approximately 48 h [6–8]. However, the mechanisms responsible for regulating the developmental cycle are poorly understood, and a more complete understanding of the functional molecules involved in developmental succession/arrest is needed [9–11]. Such information would facilitate the development of new classes of anti-malarial drugs targeting innovative metabolic pathways, with different mechanisms of action from currently available drugs, thus furthering the fight against malaria [12–14].

Miyata et al. [15–16] reported that several opioid receptor antagonists, including 7-benzylidenenaltrexone (BNTX), reversed chloroquine (CQ)-resistance in murine malaria caused by *Plasmodium chabaudi*, but had virtually no antimalarial effects themselves. These antagonists are interesting because opioids and opioid receptors are known to not only exert profound analgesic activity, but to also exert many pharmacological effects and activate second-messenger signaling cascades in various cells.

In the present study, we investigated the effects of opioid receptor antagonists, as well as cannabinoid receptor antagonists, which exert physiological functions similar to opioid receptor antagonists, on the intraerythrocytic development of *P*. *falciparum*. We also used genome-wide transcriptome profiling to identify genes that were differentially expressed in parasites exposed to BNTX and an antimalarial drug dihydroarthemisinin (DHART) that caused prominent pyknosis, which preceded parasite death, to identify putative key molecules associated with the evoking of pyknosis in the parasite.

## Materials and methods

### Parasites, cultures, and synchronization

The FCR-3/FMG (strain number: ATCC 30932) strain of *P*. *falciparum* was used in all experiments. Parasites were maintained *in vitro* in culture medium devoid of whole serum and containing basal medium supplemented with 10% growth-promoting fraction derived from adult bovine plasma (GF21; Wako Pure Chemical Industries, Osaka, Japan), as reported [17–18]. Basal medium consisted of RPMI-1640 containing 2 mM glutamine, 25 mM 4-(2-hydroxylethyl)-piperazine ethanesulfonic acid, 24 mM sodium bicarbonate (Invitrogen Ltd., Carlsbad, CA, USA), 25 μg/ml gentamicin (Sigma-Aldrich Corp., St. Louis, MO, USA), and 0.15 mM hypoxanthine (Sigma-Aldrich). The complete medium was referred to as GFSRPMI. Briefly, RBCs were preserved in Alsever’s solution [17] for 3–30 days, washed, dispensed into 24-well culture plates at a hematocrit of 2% (1 ml of suspension/well), and cultured in a humidified atmosphere of 5% CO_2_, 5% O_2_, and 90% N_2_ at 37°C. RBCs were provided by the Japanese Red Cross Society under the contract (no 28J0062). Parasitemia (percent of infected RBCs [PfRBCs]) was adjusted to 0.1% (for subculture) or 0.3% (for growth tests) by adding uninfected RBCs, unless specified otherwise, and the hematocrit was adjusted to 2% by adding the appropriate volume of culture medium.

Cultures were synchronized at the ring stage by three successive exposures to 5% (w/v) D-sorbitol (Sigma-Aldrich) at 41- and 46-h intervals [19]. After the third sorbitol treatment, residual schizonts and cell debris were removed by isopycnic density centrifugation on 63% Percoll PLUS (GE Healthcare Bio-Sciences, Tokyo, Japan). Parasites synchronized at the ring stage were adjusted to 5.0% parasitemia, unless specified otherwise, and maintained for development experiments and for RNA preparation.

A step-by-step protocol is presented on protocols.io: dx.doi.org/10.17504/protocols.io.i36cgre.

### Assessment of parasite growth and evaluation of growth inhibition

Samples were taken at the indicated times after inoculation. Thin smears were made and stained with Giemsa. Parasitemia was determined by examining more than 10000 PfRBCs and/or uninfected RBCs. The growth rate was estimated by dividing the parasitemia of the test sample after the indicated incubation period by the initial parasitemia.

Growth inhibition was measured by adding graded concentrations of reagents individually or in combination. These included the following: (1) the opioid receptor antagonists BNTX (Sigma-Aldrich), naltriben methanesulfonate hydrate (NTB, Sigma-Aldrich), and naltrexone hydrochloride (NTX, Sigma-Aldrich); (2) opioid agonist, (D-Pen^2^, D-Pen^5^)-enkephalin hydrate (DPDPE, Sigma-Aldrich); (3) the cannabinoid receptor antagonists, rimonabant hydrochloride (RIMO, *N*-(piperidin-1-yl)-5-(4-chlorophenyl)-1-(2,4-dichlorophenyl)-4-methyl-1*H*- pyrazole-3-carboxamidehydrochloride, SR141716A; Sigma-Aldrich) and SR144528 (5-(4-chloro-3-methylphenyl)-1-[(4-methylphenyl)methyl]-*N*-[(1*S*,2*S*,4*R*)-1,3,3- trimethylbicyclo[2.2.1]heptan-2-yl]-1*H*-pyrazole-3-carboxamide (Cayman Chemical, Ann Arbor, MI, USA); (4) the antimalarial drugs DHART (Sigma-Aldrich) and CQ (N'-(7-chloroquinolin-4-yl)-N,N-diethyl-pentane-1,4- diamine, diphosphate salt, Sigma-Aldrich); (5) the glutathione reductase (GR) inhibitors 2-acetylamino-3-[4-(2-acetylamino-2-carboxyethylsulfanylthio-carbonyl amino) phenylthiocarbamoyl sulfanyl] propionic acid hydrate (2-AAPA, Sigma-Aldrich) and carmustine (BCNU, 1,3-bis(2-chloroethyl)-1-nitrosourea; Sigma-Aldrich); and (6) the triose phosphate transporter (TPT) inhibitor 4,4’-diisothio cyanatostilbene-2,2’-disulfonic acid disodium salt (DIDS; Sigma-Aldrich). The solvents used were ethanol (<0.05% v/v) and dimethylsulfoxide (<0.4% v/v), and PfRBCs cultured with these solvents showed no noticeable morphological or growth rate variations compared with the control cultures

The concentrations at which the drugs were able to inhibit 50% of parasite growth (IC_50_) were calculated using ICEstimator version 1.2 (http://www.antimalarial-icestimator.net/runregression1.2.htm).

In all the experiments, culture wells were run in triplicate or quadruplicate. All experiments were repeated two to four times.

### Determination of total glutathione (GSH) and oxidized glutathione (GSSG) levels

Cultured PfRBCs/RBCs (5.4 × 10^8^ cells) were harvested, washed with phosphate-buffered saline, and disrupted by three freeze-thaw cycles. The cells were then resuspended in 100 μl 5% (w/v) sulfosalicylic acid solution and vortexed. Supernatants were obtained by centrifugation at 8,000 ×*g* for 5 min at 4°C, and transferred to 500 μl chilled, ultrapure water (final volume 600 μl). Total glutathione (GSH + GSSG) and GSSG in the supernatants were quantified using a GSSG/GSH quantification kit (Dojindo Molecular Technologies, Inc., Rockville, MD, USA).

A step-by-step protocol is presented on protocols.io: dx.doi.org/10.17504/protocols.io.i35cgq6.

### RNA preparation

Total parasite RNA was harvested using an RNase plus Mini Kit (Qiagen GmbH, Hilden, Germany) as described previously [11]. Briefly *P*. *falciparum* was isolated from infected RBCs at the end of the incubation period by lysing infected cells, and was preserved in RNAprotect Cell Reagent (Qiagen) to protect the nucleic acids from degradation. The concentration and purity of the harvested RNA were confirmed using an Agilent 2100 Bioanalyzer (Agilent Technologies Japan, Ltd., Tokyo, Japan) and NanoDrop ND-100 spectrophotometer (Thermo Fisher Scientific Inc., Tokyo, Japan).

A step-by-step protocol is presented on protocols.io: dx.doi.org/10.17504/protocols.io.i33cgqn.

### Gene expression analysis and transcriptome profiling

Genome-wide transcriptome profiling of *P*. *falciparum* during the various developmental stages was performed by Cell Innovator Inc. (Fukuoka, Japan), using the Affymetrix GeneChip Plasmodium/Anopheles Genome Array (Affymetrix Inc., Cleveland, OH, USA) and GeneChip 3’ IVT PLUS Expression kit (Affymetrix). The assay contains 5407 probe sets designed on the basis of the annotated genome and expressed sequence tags determined by the Malaria Genome-sequencing consortium. Each probe consists of 11–20 different oligonucleotide sequences (25-mers) designed to be exactly complementary to different parts of the coding region of the transcript.

The Plasmodium/Anopheles Genome Array was hybridized with 12.5 μg of amplified RNA, incubated for 16 h at 45°C, and automatically washed and stained using a GeneChip Hybridization, Wash and Stain Kit (Affymetrix) on an Affymetrix GeneChip Fluidics station. The arrays were analyzed using a GeneChip Scanner 3000. All preparations were run on quality-controlled chips and had 3/5 signal ratios of <3. The expression values of the transcripts were computed using Affymetrix GeneChip Command Console Software, with the MAS5 algorithm. For analysis, the data were normalized using Gene Spring GX11.0 (Agilent Technologies) data-mining software, per-chip normalization to the 50th percentile of the measurements for the array, and per-gene normalization to the median measurement for the gene across all the arrays in the data set. In addition, the software was used to calculate fold changes between the experimental groups and controls for each gene. All experiments for transcriptome profiling were repeated twice using separate cultures.

### Statistical analysis

The significance of differences between means was evaluated using multifactorial analysis of variance (ANOVA). All calculations were performed using GraphPad PRISM 5 (GraphPad Software, Inc., La Jolla, CA, USA). The *P* value for significance was 0.05, and all pairwise comparisons were made post hoc with Bonferroni’s test. Error bars in the graphs indicate standard deviations. Statistically significant differences were also determined by unpaired *t*-tests for gene expression analysis. Differences in gene expression (transcript) levels with *P* < 0.05 and at least a 2.0-fold increase or decrease were considered significant.

## Results

### Effect of opioid- and cannabinoid-receptor antagonists on growth of *P*. *falciparum*

**Fig 1:**
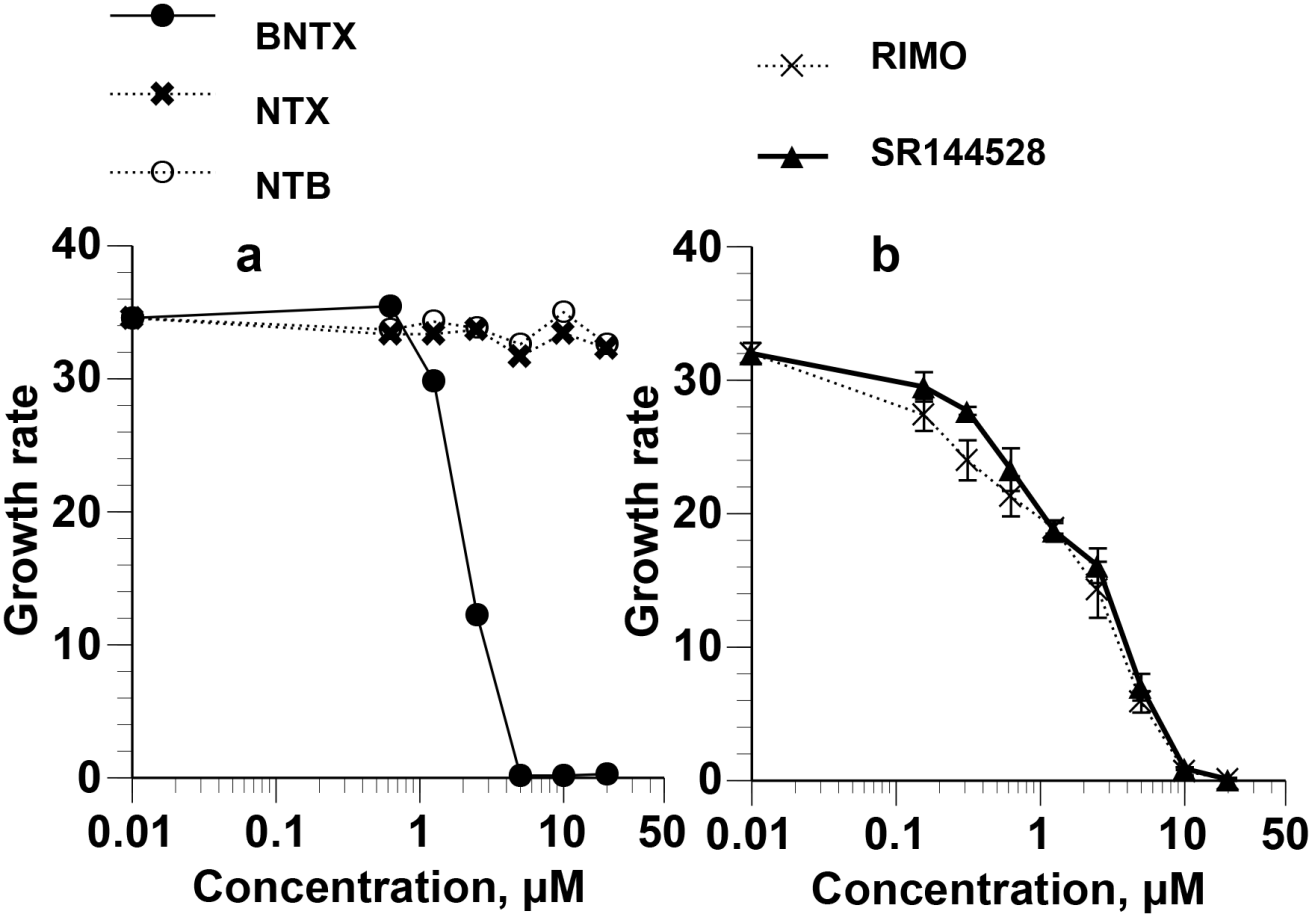
Growth-arresting effects of BNTX, RIMO, and SR144528 in asynchronous *P*. *falciparum*. Parasites were cultured for 95 h in the presence of graded concentrations of BNTX, NTB, and NTX **(a)**, and RIMO and SR144528 **(b)**. The IC_50_ values were 1.69 ± 0.10, 1.75 ± 0.21, and 1.85 ± 0.30 μM for BNTX, RIMO, and SR144528, respectively.

We determined at first the effects of increasing concentrations of the opioid receptor antagonists BNTX δ1 opioid receptor antagonist), NTB (delta 2 opioid receptor antagonist), and NTX (mu opioid receptor antagonist) on the growth of asynchronous *P*. *falciparum* in PfRBCs. The addition of BNTX, but not NTB or NTX, caused cessation of growth in parasite cultures (Fig 1a).

We also examined the effects of different concentrations of the cannabinoid receptor antagonists (reverse agonists) RIMO (CB1 receptor antagonist) and SR144528 (CB2 receptor antagonist) on the growth of asynchronous *P*. *falciparum* and showed that both cannabinoid receptor antagonists caused cessation of parasite growth (Fig 1b).

*P*. *falciparum* parasites were cultured in the presence of graded concentrations of the delta opioid receptor agonist DPDPE [20–21] (0–40 μM) for 45 and 95 h, with or without BNTX. Parasites grown in the presence of DPDPE without BNTX resembled control parasites grown in complete medium at the 2 different time points, while BNTX-induced (4 μM) growth arrest was not recovered by the addition of DPDPE. This suggests that uncertain factor(s) other than delta opioid receptors may be involved in the effect of BNTX on parasite growth. It is also possible that the intracellular processing of DPDPE may be altered and it may thus fail to affect the parasites. Further studies are needed to clarify this issue.

### Morphological arrest of development with BNTX

**Fig 2:**
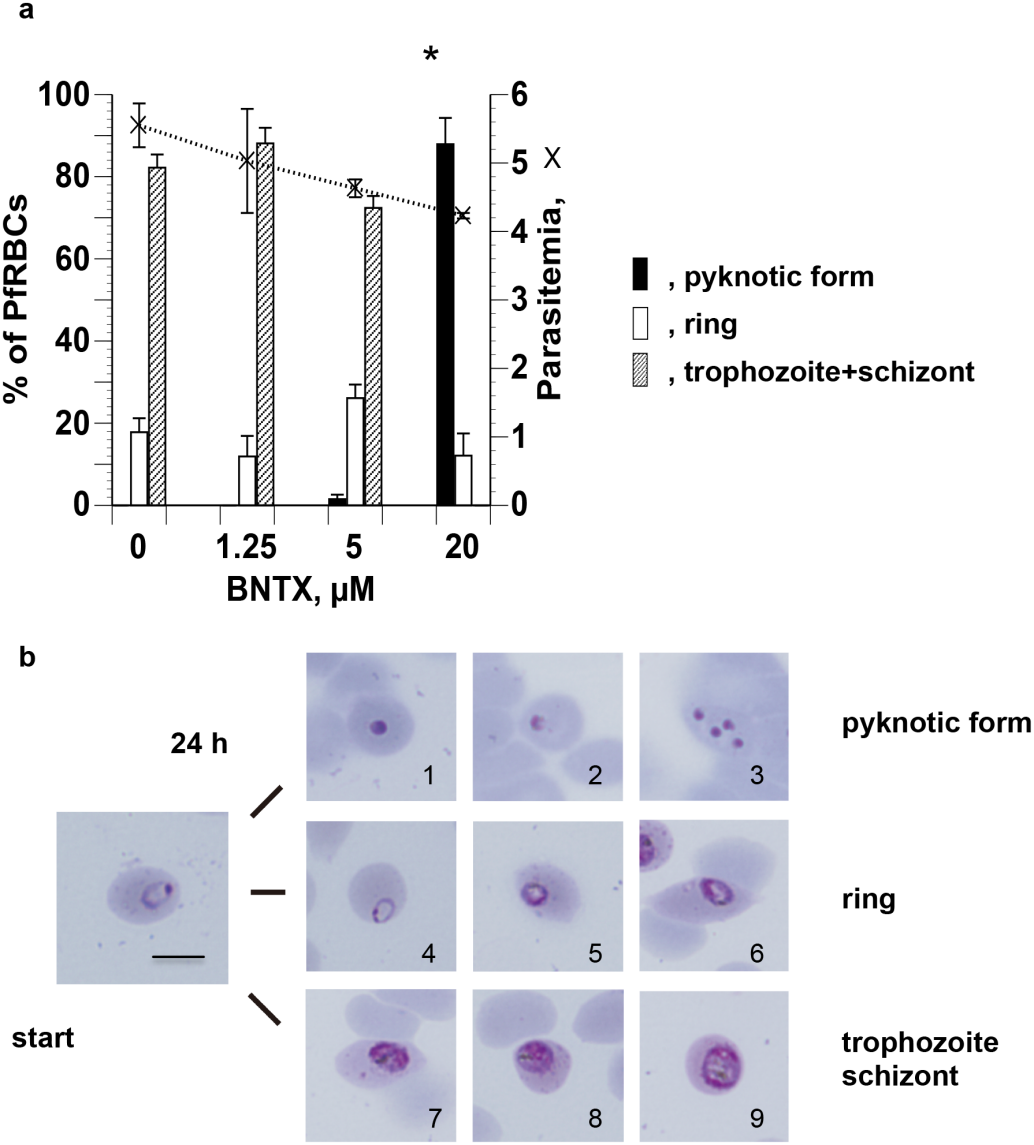
Effect of BNTX on the development and morphology of synchronized *P*. *falciparum*. **(a)** Parasites synchronized at the ring stage were cultured for 24 h in the presence of graded concentrations of BNTX. Each developmental stage and parasitemia (×) were counted after Giemsa staining. **(b)** PfRBCs showed pyknosis (1–3), arrested growth at the ring stage (4–6), and the normal development (7–9). Morphological changes were observed microscopically after culture of synchronous ring stages for 24 h in the presence of BNTX, following Giemsa staining. *Significant difference versus no BNTX. The scale bar depicts 5 μm.

We also determined the effects of different concentrations of BNTX on the developmental progression of *P*. *falciparum* parasites synchronized at the ring stage and allowed to develop for 24 h, sufficient time for development to the early schizont. BNTX arrested parasite development during the ring—trophozoite progression. All stages of parasite development were observed at the concentrations of BNTX at 1.25 μM (0.74 x IC_50_) and 5 μM (2.96 x IC_50_) (Fig 2a), but morphological developmental arrest occurred at the high concentration at 20 μM (11.8 x IC_50_), with the appearance of features such as cytoplasmic elimination, decreased size, and pyknosis (nuclear shrinkage) (Fig 2a and 2b). We referred to parasites in this state, which preceded parasite death, as pyknotic.

### Early development of pyknosis in *P*. *falciparum* following treatment with BNTX and DHART

**Fig 3:**
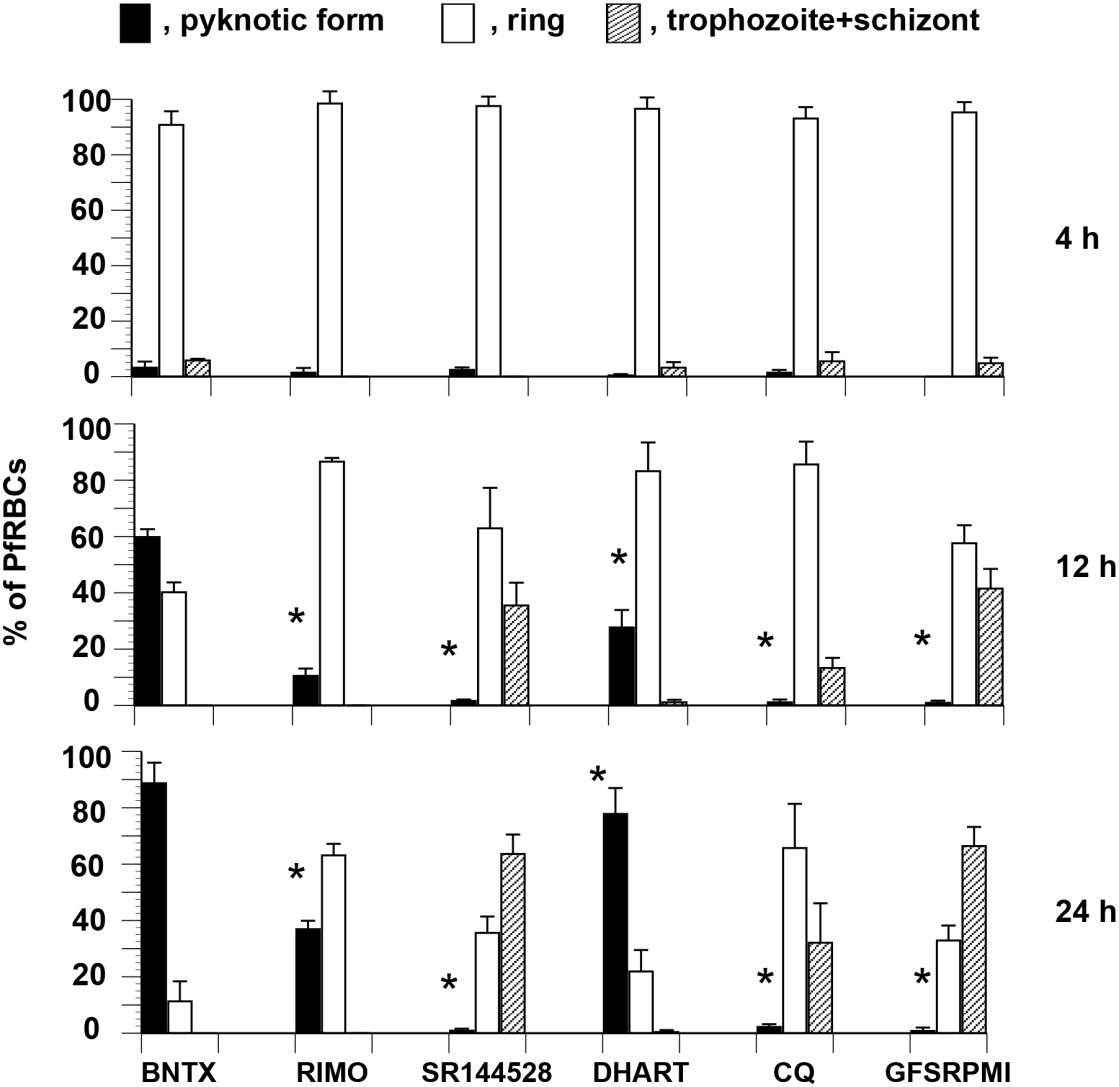
Early occurrence of pyknosis in *P*. *falciparum* cultured in the presence of BNTX, RIMO, and DHART. Parasites were characterized after 4, 12, and 24 h of synchronized culture at the ring stage. Data are presented as percentages of pyknotic form, ring form, and trophozoites + schizonts. Reagents were supplemented at concentrations of 20 μM BNTX (11.83 × IC_50_), 20 μM RIMO (11.43 × IC_50_), 20 μM SR144528 (10.81 × IC_50_), 50 nM DHART (9.98 × IC_50_), and 200 nM CQ (9.94 × IC_50_). Based on the dose-response curves, the IC_50_ values of DHART and CQ were 5.01 ± 0.11 and 20.12 ± 0.15 nM, respectively. *Significant difference versus BNTX in each culture period.

We examined the timing of pyknosis after incubation with the test drugs. *P*. *falciparum* synchronized at the ring stage were cultured in the presence of BNTX, RIMO, SR144528, and DHART and CQ (antimalarial drugs for a control), respectively, and in control medium. The distribution of the parasites among the different developmental stages was determined at 4, 12, and 24 h during the first cycle of growth. Pyknotic forms accumulated notably among *P*. *falciparum* cultured for 12 h and 24 h in the presence of BNTX or DHART, and to a lesser extent RIMO, but not in parasites cultured in SR144528 or CQ, and in control medium (Fig 3). The high incidence of pyknosis led to severe general growth arrest of parasites incubated with BNTX or DHART.

### Resumption of parasite development after exposure to DHART but not BNTX

Some parasites treated with ART derivatives are known to enter a state of quiescence, or dormancy [22–23]. To test for this possibility, we exposed parasites to BNTX (20 μM and 80 μM) or DHART (50 nM and 200 nM) for 24 or 72 h, followed by culture for 10–21 days, to detect any resumption of parasite growth. There was no growth recovery of parasites cultured with BNTX, even after 21 days. In contrast, parasites cultured with DHART showed initial growth arrest, but a few parasites (<0.001%) resumed growth as early as 3 days (50 nM) or 5 days (200 nM), even after 72 h exposure. These parasites grew and demonstrated a normal morphology. These imply that BNTX killed all the parasites at the tested doses and durations, while DHART killed most parasites, but a small percentage was still alive, suggesting that the tested doses and durations of DHART may have been inadequate for complete killing of the parasites, or induce dormant parasites.

### Transcriptome profiling of development-arrested parasites

BNTX, DHART, and RIMO arrested development of early intraerythrocytic stages of *P*. *falciparum* by inducing pyknosis. We aimed to identify the factors associated with the observed pyknosis and subsequent developmental arrest in *P*. *falciparum* by genome-wide transcriptome profiling of parasites cultured in the presence of BNTX (BNTX-12h), DHART (DHART-12h), or RIMO (RIMO-12h), respectively, to identify genes that were differentially expressed in line with the extent of pyknosis.

**Fig 4:**
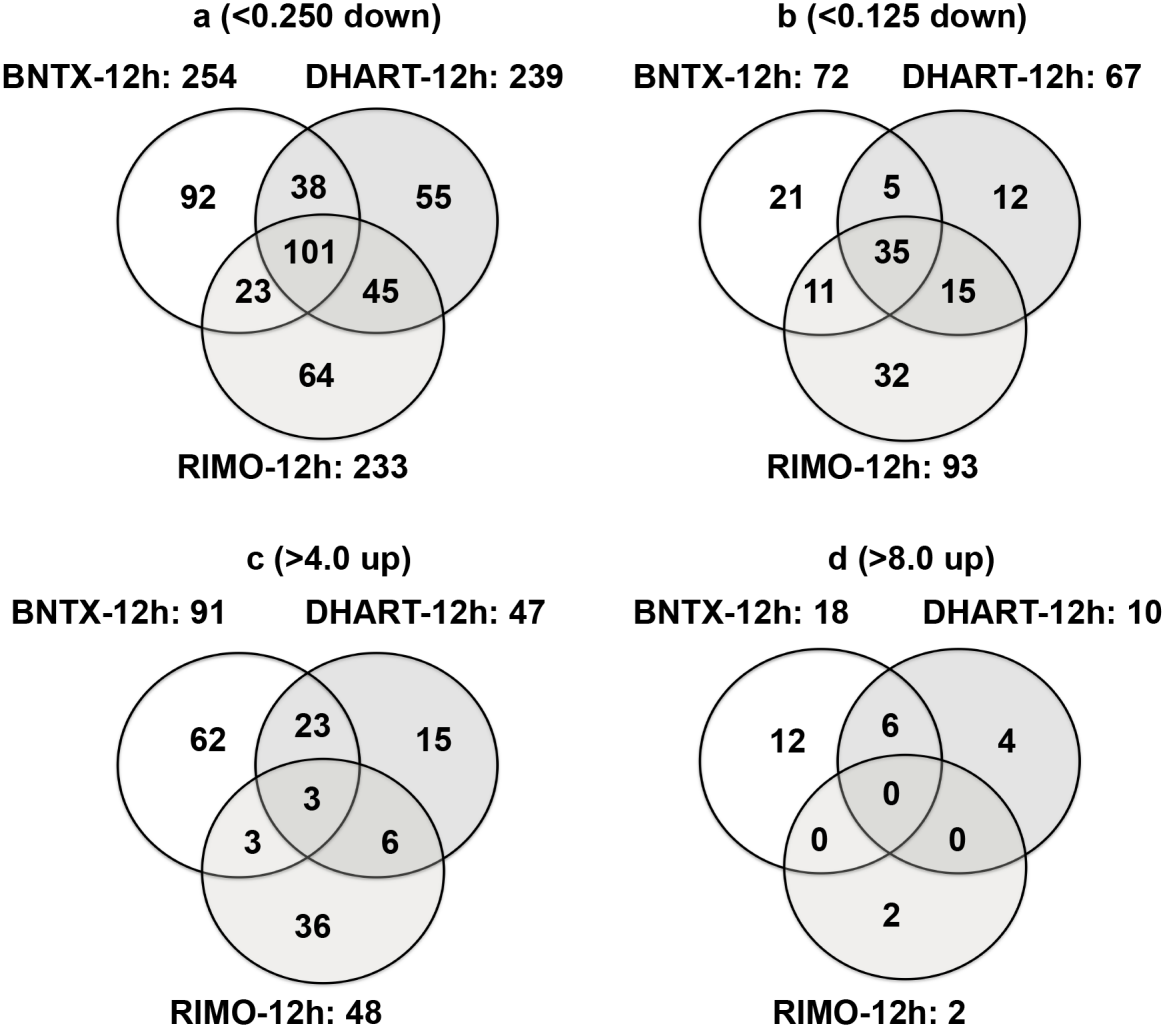
Overview of shared and unique transcripts in *P*. *falciparum* cultures with different levels of pyknosis. *P*. *falciparum* were cultured in the presence of 20 μM BNTX (BNTX-12h), 50 nM DHART (DHART-12h), and 20 μM RIMO (RIMO-12h), and in complete medium for 12 h (GFSRPMI-12h). Circles denote numbers of transcripts that were down-regulated less than 0.25-fold (<0.250 down, **a**) or less than 0.125-fold (<0.125 down, **b**), and up-regulated more than 4-fold (>4.0 up, **c**) or more than 8-fold (>8.0 up, **d**), in comparison with transcripts in GFSRPMI-12h control. Transcripts with <0.25-fold changes include those with <0.125-fold changes. Transcripts with >4-fold changes include those with >8-fold changes. Levels of parasitemia in the samples were 6.38 ± 0.15 (BNTX-12h), 6.53 ± 0.02 (DHART-12h), 6.76 ± 0.08 (RIMO-12h), and 6.54 ± 0.08 (GFSRPMI-12h). The total numbers of transcripts were 3530 for BNTX-12h, 3364 for DHART-12h, 3426 for RIMO-12h, and 3570 for GFSRPMI-12h.

Genes related to transcripts that were up- or down-regulated in *P*. *falciparum* cultured in BNTX-12h, DHART-12h, or RIMO-12h in comparison with those kept for 12 h in complete medium (no antagonist/inhibitor, GFSRPMI-12h) were selected and predicted using genome data for *P*. *falciparum* (3D7 strain) in PlasmoDB (http://www.plasmodb.org) and GenBank (National Center for Biotechnology Information, NCBI; http://www.ncbi.nlm.nih.gov). In line with the morphological assessment of parasite development, the numbers of transcripts with profoundly changed levels in comparison with GFSRPMI-12h varied among the BNTX-12h, DHART-12h, and RIMO-12h groups (Fig 4). BNTX-12h (high incidence of pyknosis) induced transcripts of <0.25-, <0.125-, >4-, and >8-fold changes in 36.2% (92/254), 29.2% (21/72), 68.1% (62/91), and 66.7% (12/18) unique transcripts, respectively. DHART-12h (moderate incidence of pyknosis) induced equivalent-fold changes in 23.0% (55/239), 17.9% (12/67), 31.9% (15/47), and 40.0% (4/10) unique transcripts, respectively, while RIMO-12h (low incidence of pyknosis) induced equivalent changes in 27.5% (64/233), 34.4% (32/93), 75.0% (36/48), and 100% (2/2) unique transcripts, respectively (Fig 4). The large number of unique transcripts detected suggested that the impacts on gene transcription varied markedly between the different drugs, and that parasite-growth arrest may be induced by different factors.

We compared further transcript levels among BNTX-12h (high pyknosis), DHART-12h (moderate pyknosis), RIMO-12h (low pyknosis), and GFSRPMI-12h (no arrest). We tentatively chose 11 transcripts that were remarkably up- or down- regulated (>5 times difference in fold changes between BNTX-12h and RIMO-12h), as being potentially associated with the development of pyknosis (Table 1). Three of these transcripts, including a conserved *Plasmodium* membrane protein (PF3D7_1472300 at PlasmoDB), GR of the parasite (PfGR; PF3D7_1419800.2), and TPT of the parasite (PfoTPT; PF3D7_0508300), showed markedly different gene expression levels (>3.0 times) between BNTX-12h and DHART-12h, or between DHART-12h and RIMO-12h cultures. We subsequently focused on these three transcripts, particularly PfGR and PfoTPT.

**Table 1:** Transcripts associated with pyknotic parasite formation.

	Target description	Pfdb ^(a)^	Signal in:	Fold change ^(b)^:			Fold difference between:	
		PF3D7	GFSRPMI-12h	BNTX-12h	DHART-12h	RIMO-12h	BNTX-12h and RIMO-12h	Regulation
1	tryptophan/threonine-rich antigen	_830500	46	14.95	2.37	0.95	15.7	up
2	**conserved Plasmodium membrane protein, unknown function**	_1472300	169	0.17	**0.40**	**1.55**	9.1	down/up
3	**glutathione reductase**	_1419800.2	2347	**0.08**	**0.32**	0.72	9.0	down
4	ubiquitin-protein ligase, putative (HRD3)	_1448400	248	0.10	0.25	0.73	7.3	down
5	**triose phosphate transporter (PfoTPT)**	_0508300	4006	0.12	**0.26**	**0.82**	6.8	down
6	conserved Plasmodium protein, unknown function	_1355600	28	10.34	4.28	1.55	6.7	up
7	pyruvate kinase 2, putative	_1037100	605	0.21	0.45	1.31	6.2	down
8	gametocyte-implicated protein (Fragment)	_935600	517	6.08	2.58	0.98	6.2	up/down
9	leucine-tRNA ligase	_828200	62	0.18	0.45	0.96	5.3	down
10	signal peptidase 21 kDa subunit	_1331300	2474	0.06	0.14	0.31	5.2	down
11	plasmepsin II	_1408000	3861	0.22	0.48	1.12	5.1	down

Targets in bold font were associated with high levels of pyknosis: fold differences between BNTX-12h and DHART-12h, or DHART-12h and RIMO-12h, >3.

^(a)^ Gene identifier in *P*. *falciparum* genomic database (PlasmoDB).

^(b)^ Fold change compared with signals in GFSRPMI-12h.

All gene IDs are prefixed by PF3D7.

### Effect of GR inhibitors on growth of *P*. *falciparum*

**Fig 5:**
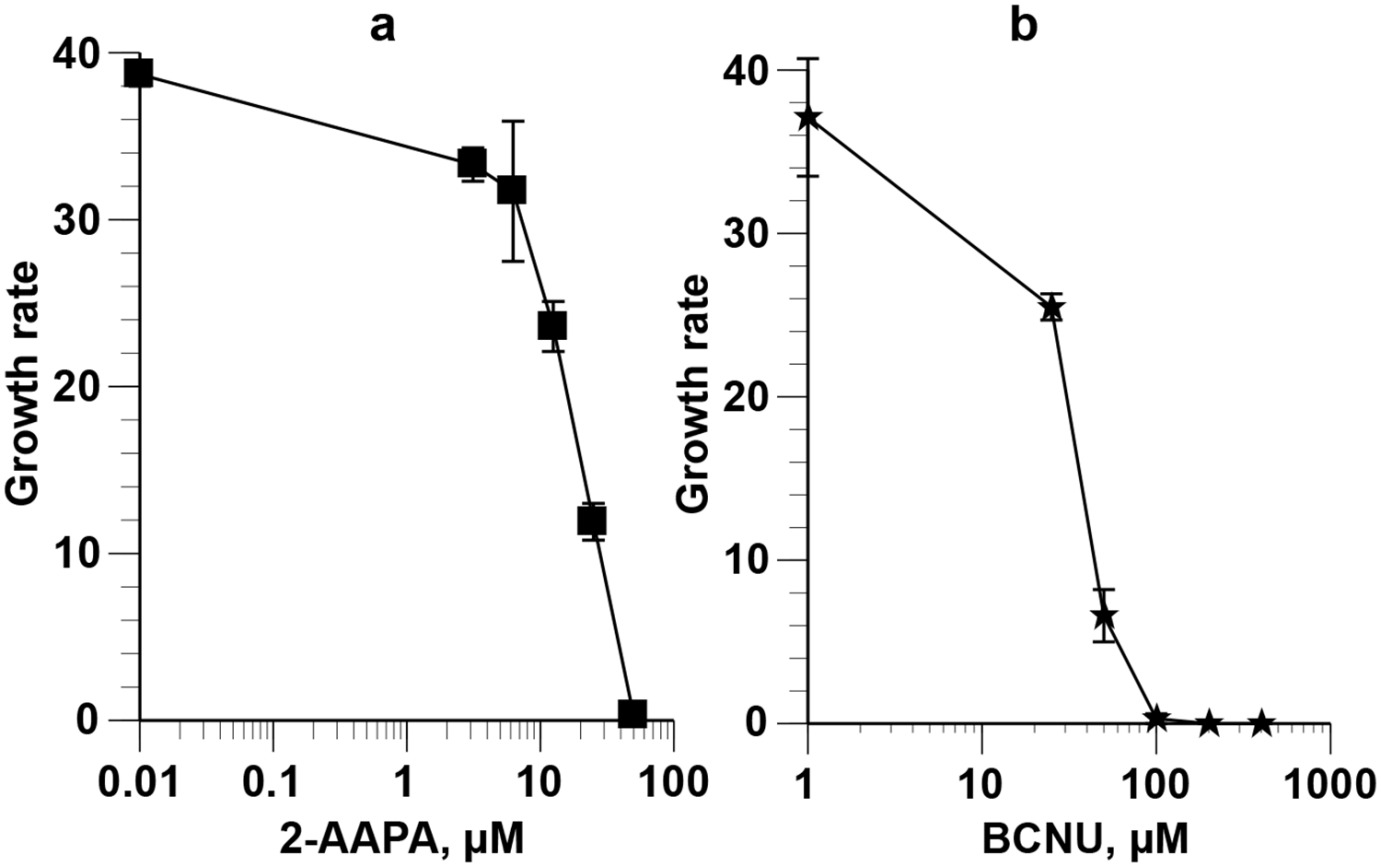
Growth-arresting effects of 2-AAPA and BCNU on asynchronous *P*. *falciparum*. Asynchronous parasites were cultured for 95 h in the presence of graded concentrations of 2-AAPA **(a)** and BCNU **(b)**. Parasitemia were counted after Giemsa staining. The IC_50_ were 15.06 ± 1.20 and 31.36 ± 0.47 μM for 2-AAPA and BCNU, respectively.

The possible involvement of PfGR in parasite pyknosis following exposure to BNTX and DHART was examined by adding the GR inhibitors, 2-AAPA and BCNU to the cultures. These inhibitors stopped the growth of parasites in asynchronous cultures (Fig 5a and 5b).

**Fig 6:**
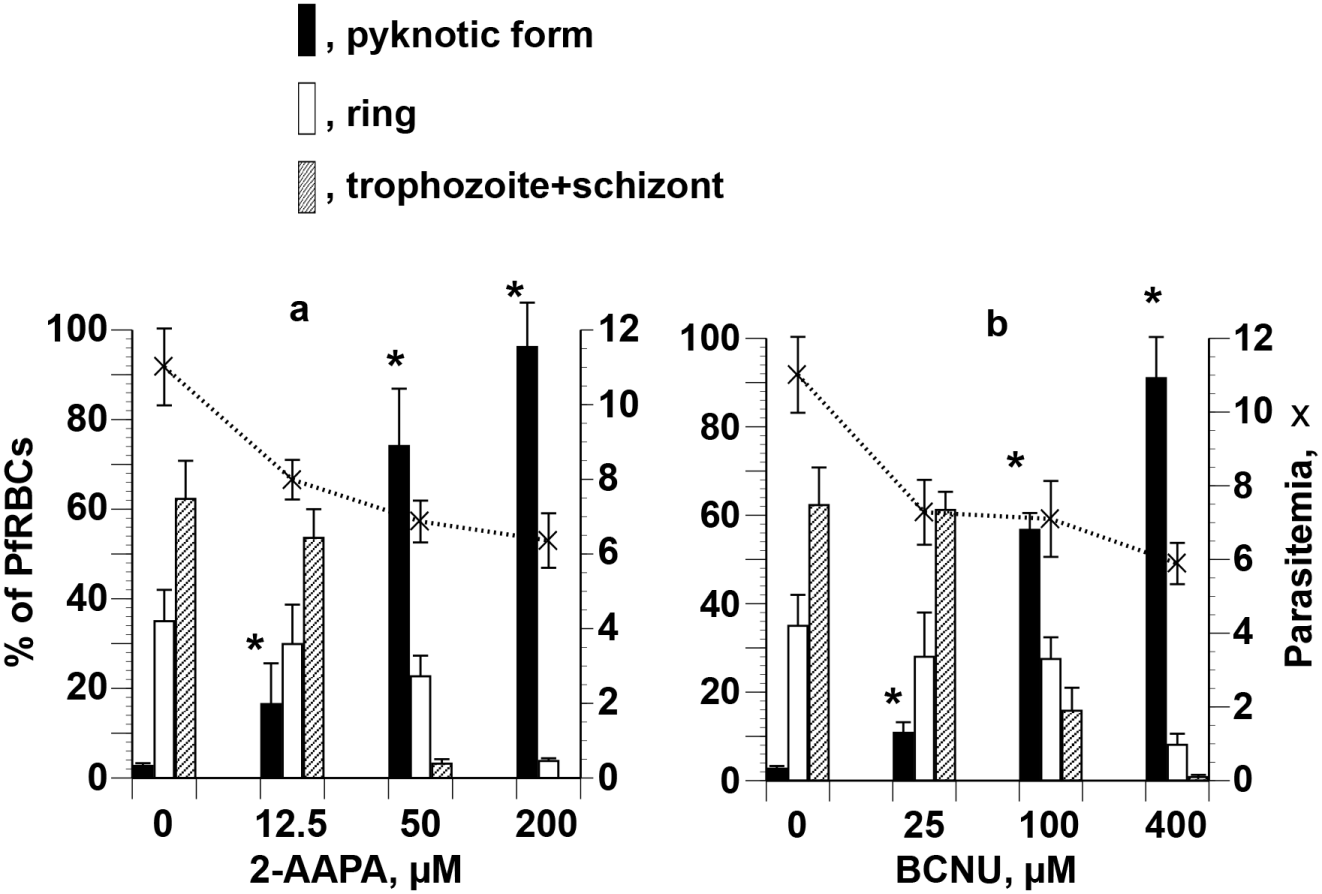
Growth-arresting effects of 2-AAPA and BCNU on synchronized *P*. *falciparum*. Parasites synchronized at the ring stage were cultured for 24 h in the presence of graded concentrations of 2-AAPA **(a)** and BCNU **(b)**. Each developmental stage was counted after Giemsa staining. *Significant difference versus no 2-AAPA or BCNU.

The effects of graded concentrations of 2-AAPA and BCNU on the development of parasites synchronized at the ring stage were also tested. 2-AAPA and BCNU arrested parasites during the ring—trophozoite—schizont-stage transitions in a concentration-dependent manner. High levels of pyknotic parasites were observed at high concentrations of both drugs (50 [3.32 x IC_50_] and 200 μM [13.28 x IC_50_] 2-AAPA, 100 [3.19 x IC_50_] and 400 μM [12.76 x IC_50_] BCNU) (Fig 6a and 6b).

### Change in redox state among development-arrested parasites

**Fig 7:**
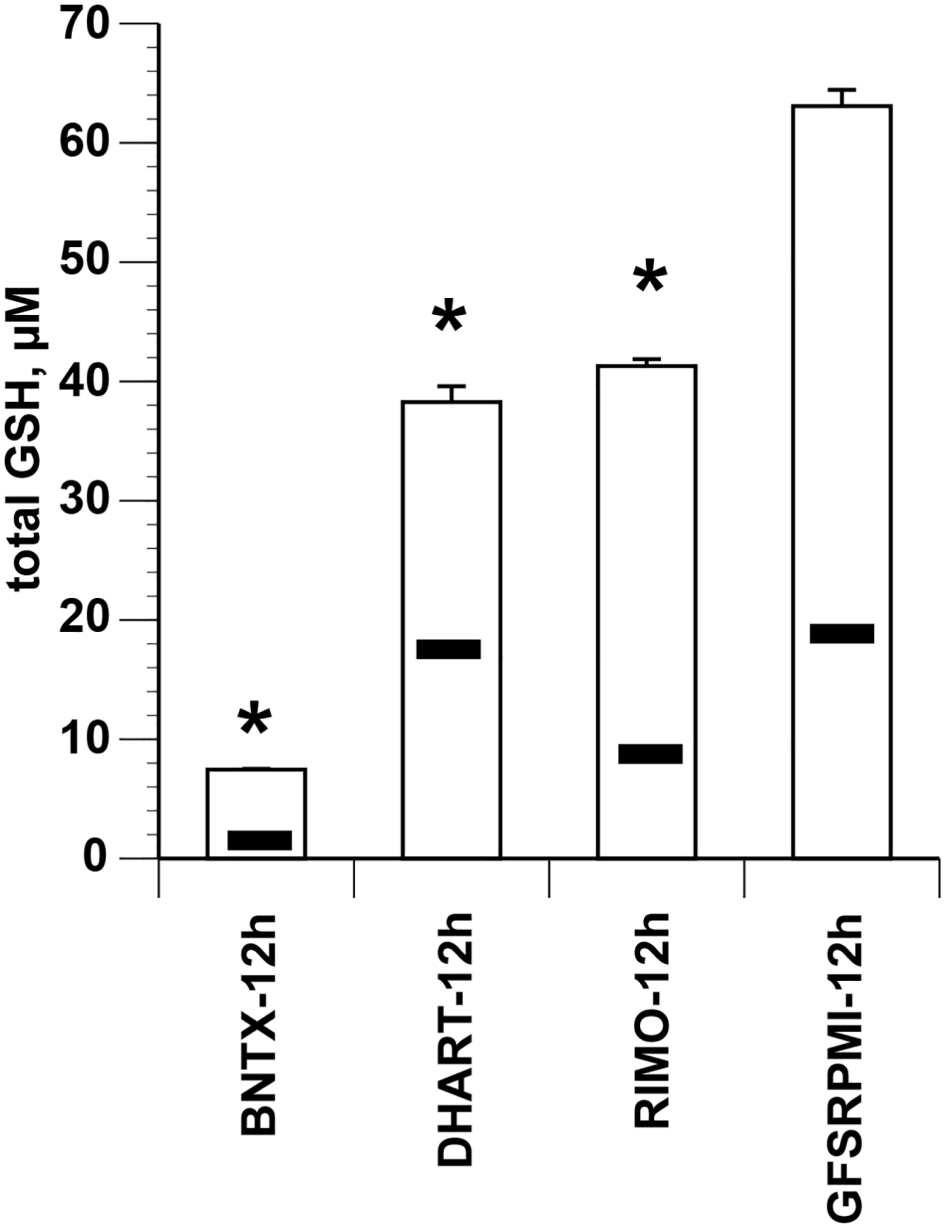
Change in levels of total GSH and GSSG in development-arrested parasites. Parasites of 5.0 ± 0.02 parasitemia synchronized at the ring stage were cultured for 12 h in the presence of 20 μM BNTX, 50 nM DHART, or 20 μM RIMO, respectively. The incidences of pyknotic forms were 54.1% ± 2.7%, 27.7% ± 2.4%, and 9.4% ± 1.8% for BNTX-12h, DHART-12h, and RIMO-12h, respectively. No pyknotic forms were detected in the GFSRPMI-12h group. GSSG levels are depicted by horizontal bars in the chart. *Significant change versus GFSRPMI-12h. The total GSH level in RBCs was 100.24 ± 1.02 μM.

The fact that GR maintains intracellular concentrations of GSH suggests that pyknosis may be linked to the cellular redox state. We therefore quantified total GSH and GSSG levels under each experimental condition, and in GFSRPMI-12h (control) and normal RBCs. The levels of total GSH and GSSG were significantly decreased in all PfRBC samples compared with normal RBCs. The greatest significant reductions in total GSH levels occurred in the BNTX-12h (high pyknosis), followed by the DHART-12h (moderate pyknosis), and RIMO-12h (low pyknosis) groups (Fig 7). These results suggest that PfGR may be involved in pyknosis by affecting GSH levels (redox state).”

### Effect of TPT inhibitor on growth of *P*. *falciparum*

**Fig 8:**
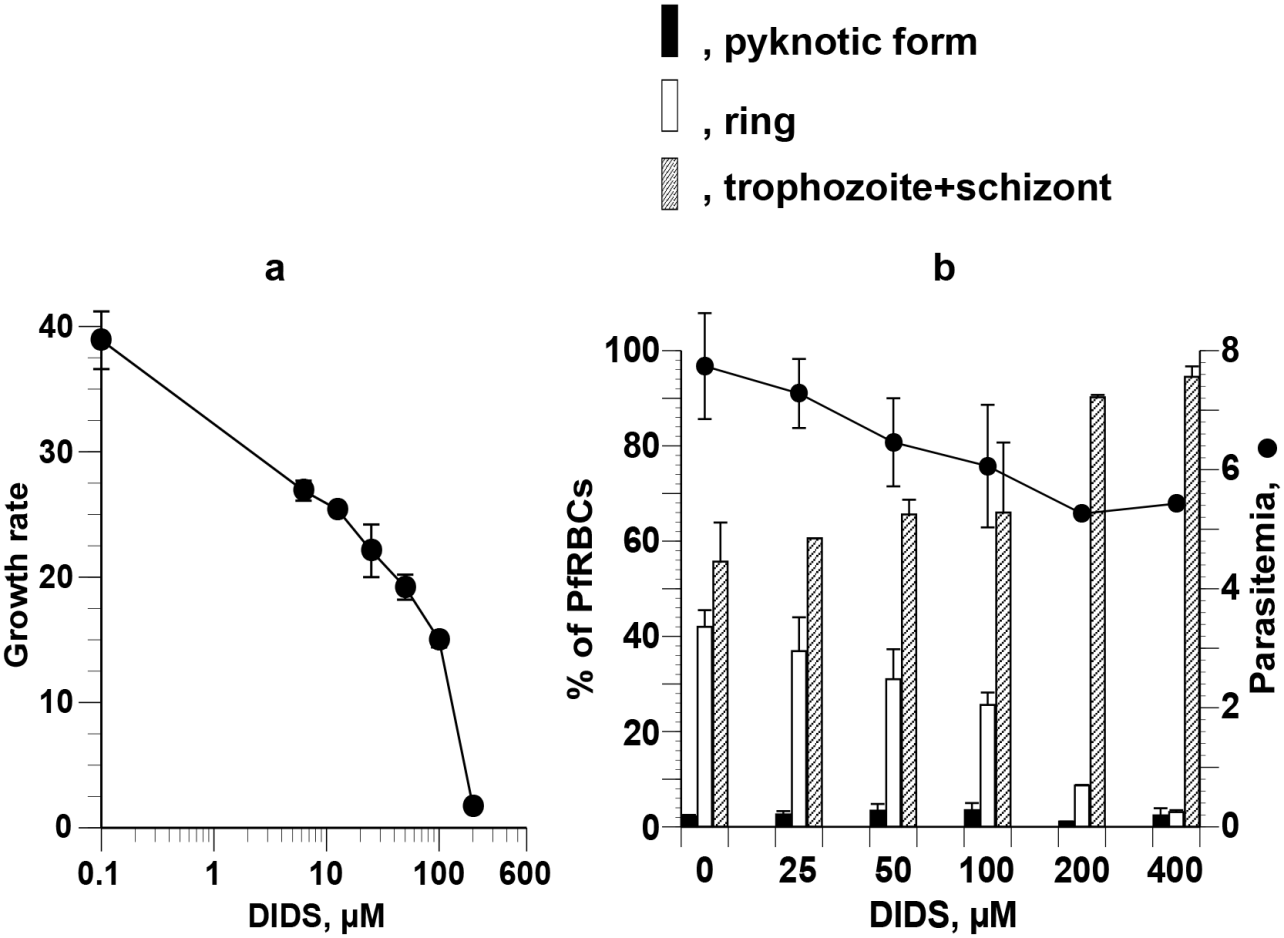
Growth-arresting effects of DIDS on asynchronous and synchronized *P*. *falciparum*. **(a)** Asynchronous parasites were cultured for 95 h in the presence of graded concentrations of DIDS. The IC_50_ was 25.21 ± 4.34 μM. **(b)** Parasites synchronized at the ring stage were cultured for 24 h in the presence of graded concentrations of DIDS. Each developmental stage and parasitemia were counted after Giemsa staining. % of pyknotic forms are not significantly different at various concentrations versus no DIDS.

To examine the possible effects of PfoTPT activity on the development of *P*. *falciparum*, we cultured PfRBCs in the presence of graded concentrations of the selective TPT inhibitor DIDS. DIDS prevented parasite growth in asynchronous cultures at relatively high concentrations (Fig 8a).

The effects of different concentrations of DIDS on the development of parasites synchronized at the ring stage were also tested. DIDS did not arrest parasite development during the ring—trophozoite—schizont-stage transitions, and the levels of pyknosis were similar to those in control cultures, even at high concentrations of DIDS (Fig 8b).

## Discussion

The delta 1 opioid receptor antagonist, BNTX, and cannabinoid receptor antagonists, RIMO and SR144528, caused growth arrest of intraerythrocytic *P*. *falciparum*. BNTX, as well as the antimalarial drug DHART, notably induced pyknosis in the parasite cells after a short period of incubation. We compared genome-wide transcriptome responses in *P*. *falciparum* following exposure to BNTX, DHART, and RIMO, which induced different pyknotic responses, and selected 11 transcripts potentially associated with pyknosis. Three of these, PfGR, PfoTPT, and conserved *Plasmodium* membrane protein, showed markedly different gene expression levels among parasites exposed to the three different agents, reflecting the degree of pyknosis. The genes identified in this study may encode key factor(s) triggering pyknosis, or factors concomitant with developmental arrest. We further examined the roles of PfGR and PfoTPT using specific inhibitors. The GR inhibitors 2-AAPA and BCNU [24–26], but not the TPT inhibitor DIDS [27–28], caused marked cessation of *P*. *falciparum* growth associated with a high degree of pyknosis, similar to the levels caused by BNTX and DHART. These results suggest that PfGR may play a major role in the development of pyknosis in *P*. *falciparum*. However, the additional involvement of other proteins associated with pyknosis cannot be completely excluded.

The use of opioid analgesics has a long history in clinical settings, though the comprehensive action of opioids is still not fully understood [29–30]. Opioids, including endogenous and exogenous opioids, act at opioid receptors (namely, kappa, mu, delta, and nociceptin/orphanin opioid receptors) [30–31]. In addition to pain modulation and addiction, opioid receptors are widely involved in various physiological and pathophysiological activities, including the regulation of membrane-ion homeostasis, cell proliferation, and neurodegenerative disorders [29]. Opioid receptors are typical G protein-coupled receptors and activate canonical second-messenger signaling cascades to influence diverse cellular functions [29–30]. In the current study, BNTX, but not NTB and NTX, effectively inhibited the successive ring—trophozoite—schizont progression of *P*. *falciparum* during early developmental stages, associated with the development of pyknosis in ring forms. BNTX demonstrated a resistance-reversing effect in CQ-resistant murine malaria caused by *P*. *chabaudi* infection *in vivo*, but virtually no antimalarial effects [15–16]. In contrast, BNTX exhibited a direct antimalarial effect on *P*. *falciparum in vitro* in the current study. These apparently conflicting results may be produced by *in vivo* versus *in vitro* experiments. Among the opioid antagonists tested, only the delta 1 antagonist BNTX exerted a profound growth-arresting effect on *P*. *falciparum*. BNTX has a Michael acceptor structure, which differs from NTX which otherwise has a similar formula to BNTX [15–16], suggesting that the Michael acceptor moiety may be involved in the induction of developmental arrest by BNTX. However, no opioid-receptor gene with clear homology to human opioid receptors has yet been annotated in *P*. *falciparum* (PlasmoDB). The molecule(s) involved in the interaction between BNTX and *P*. *falciparum*, and the mechanisms responsible for growth arrest and gene-expression regulation in the parasite remain to be determined.

Cannabinoids are known to exert a variety of central and peripheral physiological functions similar to opioids. Endogenous and exogenous cannabinoids act at cannabinoid receptors, which comprise two pharmacologically distinct receptors, CB1 and CB2, both of which regulate a variety of central and peripheral physiological functions in humans and other animals. At the cellular level, these receptors are critically involved in proliferation, motility, adhesion, and apoptosis of various cell types [32–34]. Cannabinoid receptors are typical G protein-coupled receptors, and their activation leads to inhibition of adenylyl cyclase and consequent reductions in cyclic AMP accumulation. In addition, both CB1 and CB2 receptors regulate the phosphorylation and activation of different members of the mitogen-activated protein kinase family, and mitogen-activated protein kinase cascades are thus involved in cannabinoid regulation of cell survival/death, glucose metabolism, and ionic current control in various cells [32]. Notably, although the selective CB1 antagonist RIMO and the selective CB2 antagonist SR144528 induced few populations of pyknotic parasites, their IC_50_ values were almost comparable to that of BNTX. Additional studies are currently underway to elucidate the roles of cannabinoid antagonists in *P*. *falciparum* growth.

The antimalarial activity of ART derivatives is known to result from alterations in the parasite’s redox balance caused by their endoperoxides. ART derivatives interact with hemin to produce reactive oxygen species, resulting cellular damage [35–36]. In the current study, PfGR gene expression was markedly down-regulated, and it was thus predicted to be the main molecular factor associated with the development of pyknosis or dormancy in *P*. *falciparum* following treatment with either DHART or BNTX. A reduction in intracellular levels of GSH, as detected in the current study, may also increase reactive oxygen species—induced damage. GR is a homodimeric flavoenzyme that maintains high intracellular concentrations of GSH by catalyzing the reduction of glutathione disulfide (GSSG). GR and GSH thus perform central functions in cellular redox metabolism in various cells and in *P*. *falciparum* [37–38]. Although the functions of GSH and GSH-related enzymes, including GR, in relation to the antimalarial activity of DHART in *P*. *falciparum* remain poorly understood, intracellular GSH levels and protein S-glutathionylation have been proposed to be involved in the mechanism of ART-derivative action in *Plasmodium* spp. [39–40]. Kelch propeller domain (K13-propeller) proteins have also been associated with ART resistance in *P*. *falciparum* [41–42]. The human kelch domain-containing protein KEAP1, which showed maximum homology with the *P*. *falciparum* K13 propeller protein, induced the expression of antioxidant enzymes [43], and it is possible that the *P*. *falciparum* K13-propeller protein may perform a similar function, inducing the expression of antioxidant enzymes. Consistent with this possible mechanism, DHART reduced the expression of PfGR in association with growth arrest of *P*. *falciparum* in the current study. In addition to inducing endoperoxides, the prominent antimalarial effect of ART derivatives may involve the redox machinery via PfGR. Further studies are needed to determine the role of PfGR in the efficacy of DHART in terms of arresting parasite development.

During the erythrocytic stage of their life cycle, malaria parasites are exposed to oxidative stress produced by toxic heme and other hemoglobin-decomposition products [44–45]. The GSH system is believed to play a key role in the defense of malaria parasites against oxidative stress. This is particularly important in the development of CQ-resistance, and CQ-resistant parasites have been reported to exhibit increased GSH content. Despite the lack of conclusive evidence for a link between GSH levels and CQ response, currently available information suggests that *P*. *falciparum* could enhance its resistance to CQ by bolstering GSH levels [38, 44–47]. However, it should be noted that CQ only caused limited pyknosis in the current study, implying that the antimalarial effect of CQ may differ from that of DHART. Further research is needed to identify a link between GSH, PfGR, CQ, and the development of pyknosis.

Two sugar phosphate transporters are present in apicoplast membranes of *P*. *falciparum*: PfoTPT (PF3D7_0508300), which is located in the outermost apicoplast membrane, and a phosphoenolpyruvate/phosphate translocator (PfiTPT, PF3D7_0530200), located in the innermost apicoplast membrane [48–49]. PfoTPT and PfiTPT transport substrates, including dihydroxyacetone phosphate, glyceraldehyde-3-phosphate, and phosphoenolpyruvate. TPT gene knockout was lethal for *Plasmodium berghei* in a murine model [50], while transcripts corresponding to PfoTPT were markedly reduced in *P*. *falciparum* in cultures with added BNTX or DHART. However, DIDS, which selectively binds TPTs and blocks their transporting ability [51–52], had no visible effect on the development of pyknosis, in contrast to parasites cultured in the presence of BNTX, 2-AAPA, and BCNU, despite causing general growth arrest. This implies that decreased PfoTPT transcription may not be responsible for pyknosis.

In conclusion, we identified molecular factors potentially associated with the development of pyknosis in *P*. *falciparum* by genome-wide transcriptome profiling of parasites exposed to BNTX and DHART, and by using specific inhibitors. The results suggest that PfGR may play a critical role in the development of pyknosis in *P*. *falciparum*, and provide important information for the development of novel, effective antimalarial strategies. Our understanding of PfGR functions remains incomplete, and genetic evidence is required to confirm the mechanisms underlying the role of PfGR in parasite pyknosis. Further studies are also needed to clarify the target molecules of BNTX, DHART, and cannabinoid antagonists in *P*. *falciparum*.

## Supporting information

S1 Table Fold changes in transcript levels detected in BNTX-12h, DHART-12h and RIMO-12h compared to GFSRPMI-12h. (PDF)

S2 Table Gene IDs and target description of the selected transcripts. (DOC)
