# Supplementary material for: Profiling molecular factors associated with pyknosis and developmental arrest induced by an opioid receptor antagonist and dihydroartemisinin in Plasmodium falciparum
Source: PLoS One. 2017 Sep 21;12(9):e0184874. doi: 10.1371/journal.pone.0184874 (PMC5608265; doi:10.1371/journal.pone.0184874)
Supplement: S1 Table — (PDF) [file pone.0184874.s001.pdf]

S1. Fold changes in transcript levels detected in BNTX-12h, DHART-12h and RIMO-12h compared to GFSRPMI-12h.

	Signal in:	Fold change:		
ProbeSetID	GFSRPMI-12h	BNTX-12h	DHART-12h	RIMO-12h
AB016617.1_RC_s_at	29	0.668	0.846	1.506
AF008978.1_RC_at	12	0.951	1.138	1.177
AF008978.1_x_at	5	0.992	1.121	1.006
AF008979.1_at	5	1.004	0.818	0.757
AF008979.1_RC_at	6	1.115	1.447	1.150
AF008979.1_x_at	6	1.083	0.922	0.953
AF008980.1_at	5	1.101	0.946	1.054
AF008980.1_RC_at	6	1.194	0.997	1.062
AF008980.1_RC_x_at	8	0.993	0.987	0.986
AF008980.1_x_at	5	1.177	1.058	1.161
AF008981.1_RC_at	14	1.505	0.855	0.899
AF008981.1_x_at	10	1.230	0.806	0.946
AF008982.1_at	6	1.083	0.928	0.951
AF008982.1_RC_at	7	0.976	1.085	1.009
AF008983.1_at	6	0.878	0.796	0.905
AF008983.1_x_at	6	1.103	0.901	0.932
AF008984.1_at	6	0.889	0.924	0.896
AF008984.1_RC_at	6	1.310	1.322	1.177
AF008985.1_at	6	1.333	0.972	0.986
AF008986.1_at	8	1.310	0.790	1.122
AF008986.1_x_at	9	1.586	1.158	1.251
AF008987.1_RC_at	14	1.705	1.538	1.319
AF008987.1_x_at	7	0.931	0.905	1.172
AF008988.1_at	16	**8.743**	**8.315**	1.841
AF008988.1_RC_at	10	1.309	0.806	0.899
AF008988.1_x_at	17	**5.534**	**5.206**	1.302
AF008989.1_at	7	1.041	1.212	0.798
AF008989.1_x_at	7	1.520	1.120	0.755
AF039275.1_at	5	1.240	1.073	0.971
AF039276.1_s_at	5	1.258	1.135	1.033
AF039276.1_x_at	17	0.881	1.091	1.040
AF039277.1_at	6	1.159	1.140	1.035
AF039277.1_RC_at	7	0.929	0.850	1.000
AF039278.1_at	14	1.485	0.853	0.992
AF039278.1_RC_at	18	1.414	0.543	0.745
AF039279.1_at	6	1.306	0.987	1.233
AF039279.1_RC_at	9	0.926	0.978	0.919
AF039280.1_at	5	1.307	1.136	1.152
AF039280.1_RC_at	41	1.280	1.068	1.865
AF039280.1_RC_x_at	19	1.302	1.165	**2.127**
AF039280.1_x_at	7	1.094	1.027	1.004
AF039281.1_at	5	1.184	0.996	1.065
AF039282.1_s_at	5	1.219	0.907	0.916
AF039283.1_at	14	1.379	1.071	1.477
AF039283.1_x_at	10	1.536	1.055	1.774
AF041425.1_RC_at	6	1.035	0.995	1.005
AF041425.1_RC_x_at	7	1.075	0.950	1.006
AF041425.1_x_at	6	0.883	0.917	0.909
AF042160.1_RC_at	10	1.452	0.731	0.868
AF042160.1_RC_x_at	8	1.118	0.645	0.811
AF042160.1_s_at	5	0.983	1.053	0.912
AF057712.1_s_at	550	**0.343**	0.736	0.679
AF061079.1_s_at	26741	0.690	0.850	0.929
AF061155.1_at	6	1.856	1.016	1.041
AF061155.1_RC_at	143	1.426	1.140	0.793
AF061155.1_RC_x_at	33	1.464	1.359	0.920
AF061960.1_at	7	0.863	1.080	0.910
AF062397.1_at	8	0.772	0.888	0.725

AF065205.1_RC_s_at	5	0.972	0.864	0.905
AF065205.1_s_at	5	0.973	1.038	1.127
AF065206.1_RC_at	5	0.984	1.160	1.164
AF065207.1_RC_at	6	1.000	0.836	1.153
AF065207.1_s_at	5	0.948	1.143	1.004
AF087698.1_RC_at	9	0.916	1.175	1.205
AF087698.1_s_at	5	0.921	0.870	0.904
AF087699.1_s_at	4	1.032	1.018	1.002
AF091994.1_at	5	0.944	0.899	1.025
AF091994.1_RC_at	11	1.096	1.147	0.983
AF091994.1_s_at	475	0.509	1.128	0.625
AF091994.1_x_at	6	0.854	0.914	0.919
AF091995.1_RC_at	43	1.580	1.105	1.347
AF091995.1_s_at	872	**2.268**	1.069	1.563
AF115767.1_at	177	1.909	0.512	**2.385**
AF115767.1_s_at	263	1.604	1.484	**3.626**
AF132898.1_RC_at	9	0.999	0.719	0.872
AF145237.1_RC_at	18	0.789	0.615	**0.393**
AF161312.1_at	7	0.919	0.899	0.864
AF221775.1_at	7	1.350	1.309	1.047
AF221775.1_RC_at	10	0.904	0.958	0.897
AF221776.1_at	6	0.800	0.832	0.758
AF221776.1_RC_at	7	0.844	1.028	1.338
AF221776.1_RC_x_at	6	1.132	1.047	1.181
AF221776.1_s_at	19	1.537	**2.141**	1.567
AF221776.1_x_at	5	0.840	0.903	0.831
AF221777.1_RC_x_at	10	1.236	1.523	1.494
AF221777.1_x_at	6	1.099	0.961	1.030
AF221778.1_RC_at	5	1.010	1.054	0.954
AF221778.1_RC_x_at	6	0.882	0.969	1.014
AF221778.1_x_at	6	0.945	0.922	1.003
AF221779.1_at	6	0.845	0.895	0.796
AF221780.1_at	5	1.015	0.930	0.924
AF221780.1_x_at	5	0.886	0.844	0.820
AF221781.1_at	5	1.117	1.038	1.136
AF221781.1_RC_at	6	0.960	0.981	0.979
AF221781.1_RC_x_at	6	1.079	1.069	1.096
AF221781.1_x_at	5	0.939	1.006	1.060
AF221782.1_at	5	1.066	1.207	1.114
AF221782.1_s_at	6	0.981	0.826	0.868
AF221782.1_x_at	5	0.888	0.925	0.982
AF221783.1_at	5	1.246	1.055	1.031
AF221784.1_at	5	1.010	1.030	1.029
AF221784.1_RC_at	7	0.832	0.866	0.945
AF221785.1_RC_at	6	0.721	0.977	1.245
AF221785.1_RC_x_at	6	1.260	1.235	1.162
AF221785.1_x_at	5	1.010	0.990	1.165
AF221786.1_at	5	0.929	0.985	0.996
AF221786.1_RC_at	22	0.870	0.910	0.715
AF221786.1_RC_x_at	9	0.765	0.989	1.015
AF221786.1_x_at	6	0.916	0.903	0.917
AF221787.1_at	6	0.978	0.870	0.902
AF221787.1_RC_at	7	1.189	0.712	0.776
AF221787.1_RC_x_at	8	0.838	0.811	0.898
AF221787.1_s_at	8	1.661	1.117	0.903
AF221787.1_x_at	7	0.888	0.903	0.773
AF221788.1_at	7	0.798	0.741	1.127
AF221789.1_x_at	5	1.062	0.959	0.951
AF221790.1_at	6	1.143	0.961	0.829
AF221790.1_RC_at	6	1.099	1.235	1.173
AF221790.1_x_at	5	0.942	0.999	0.952
AF221791.1_at	5	0.984	1.017	0.878

AF221791.1_RC_at	7	1.061	0.977	1.087
AF221791.1_RC_x_at	7	1.295	1.176	1.349
AF221791.1_x_at	5	1.110	1.137	1.210
AF221792.1_at	5	0.857	0.963	0.898
AF221792.1_RC_at	6	0.846	1.128	1.115
AF221792.1_RC_x_at	7	1.036	0.988	1.208
AF221792.1_x_at	4	1.120	1.014	1.177
AF221793.1_s_at	5	1.057	1.082	1.176
AF221793.1_x_at	5	1.075	1.097	1.077
AF221794.1_at	6	0.768	0.669	0.797
AF221794.1_RC_at	9	0.936	1.124	0.915
AF221794.1_RC_x_at	8	1.167	1.038	1.022
AF221794.1_x_at	6	1.022	0.958	0.849
AF221795.1_at	5	0.981	0.938	0.878
AF221796.1_at	8	1.135	1.277	0.869
AF221796.1_RC_at	6	0.922	0.949	1.059
AF221796.1_RC_x_at	7	0.985	1.323	0.962
AF221796.1_s_at	5	1.070	0.921	0.902
AF221796.1_x_at	14	0.922	1.142	0.843
AF221797.1_RC_at	8	0.897	0.964	0.972
AF221797.1_s_at	5	0.985	0.860	0.837
AF221798.1_at	6	0.798	0.772	0.780
AF221798.1_RC_at	6	0.876	0.941	0.959
AF221798.1_x_at	5	1.153	1.014	0.896
AF221799.1_at	5	1.009	1.129	1.037
AF221799.1_RC_at	6	1.049	1.072	1.141
AF221800.1_RC_x_at	10	0.901	1.127	1.087
AF221800.1_x_at	5	1.140	0.986	1.133
AF221801.1_at	38	**2.712**	1.101	0.622
AF221801.1_RC_at	31	0.722	0.684	0.532
AF221801.1_RC_x_at	22	1.199	0.637	0.612
AF221801.1_x_at	207	**2.420**	1.090	0.726
AF221802.1_at	5	1.006	1.000	1.006
AF221802.1_RC_at	6	1.085	0.973	1.073
AF221802.1_RC_x_at	7	1.002	1.021	1.024
AF221802.1_x_at	5	1.020	1.072	0.936
AF221803.1_at	7	0.803	0.747	0.878
AF221804.1_at	5	0.908	0.816	0.921
AF221804.1_RC_at	7	1.010	1.015	1.011
AF221804.1_RC_x_at	6	1.134	1.220	1.193
AF221804.1_x_at	5	1.024	0.949	0.964
AF221805.1_at	5	1.063	0.928	0.994
AF221806.1_at	8	0.951	1.008	0.878
AF221806.1_RC_at	6	0.957	1.224	0.981
AF221806.1_x_at	9	0.912	0.843	0.925
AF221807.1_RC_at	9	0.954	0.803	0.823
AF221807.1_RC_x_at	6	1.031	1.079	0.810
AF221807.1_x_at	5	1.048	0.885	1.059
AF221808.1_RC_at	28	1.141	1.405	1.170
AF221808.1_RC_x_at	21	0.843	1.114	1.092
AF221808.1_s_at	7	0.720	0.706	0.667
AF221808.1_x_at	7	0.986	0.868	0.884
AF221809.1_at	5	1.195	1.337	1.040
AF221809.1_RC_at	14	1.090	0.522	0.545
AF221810.1_at	6	0.984	0.899	0.954
AF221810.1_RC_at	6	1.070	0.961	0.961
AF221811.1_at	7	1.076	1.110	1.201
AF221811.1_RC_at	7	1.056	1.346	1.251
AF221811.1_RC_x_at	10	0.856	0.856	0.895
AF221812.1_at	5	0.955	1.017	0.954
AF221812.1_x_at	5	0.929	0.906	0.947
AF221813.1_at	5	1.040	1.144	1.132

AF221814.1_RC_at	6	1.191	1.023	1.201
AF221814.1_RC_s_at	7	0.746	1.015	0.993
AF221814.1_RC_x_at	6	1.144	1.229	1.189
AF221814.1_s_at	8	1.085	0.748	0.800
AF221814.1_x_at	7	0.831	0.718	0.775
AF221815.1_at	5	0.972	0.882	0.939
AF221815.1_RC_at	5	1.078	1.078	1.078
AF221816.1_at	10	**2.813**	1.333	1.624
AF221816.1_x_at	5	1.118	0.971	1.255
AF221817.1_at	5	0.995	0.922	0.968
AF221817.1_x_at	5	1.068	0.975	1.038
AF221818.1_at	5	1.003	1.005	1.078
AF221818.1_s_at	7	0.772	0.753	0.851
AF221818.1_x_at	5	1.016	0.896	1.030
AF221819.1_at	5	1.075	1.030	1.033
AF221819.1_RC_at	6	1.190	1.046	0.921
AF221819.1_x_at	6	0.999	1.049	0.873
AF221820.1_at	5	1.099	0.959	0.939
AF221820.1_RC_at	7	1.094	1.347	1.415
AF221820.1_x_at	8	0.876	0.622	0.732
AF221821.1_at	6	0.835	0.984	0.906
AF221821.1_RC_at	5	0.909	0.940	0.976
AF221821.1_RC_x_at	5	0.893	1.125	0.999
AF221822.1_RC_at	8	0.899	0.863	0.866
AF221822.1_x_at	5	1.163	1.089	1.103
AF221823.1_s_at	13	1.099	1.240	1.028
AF221824.1_at	5	0.957	1.035	0.895
AF221824.1_RC_at	6	1.058	1.092	0.880
AF221824.1_RC_x_at	6	1.237	0.993	1.289
AF221824.1_x_at	5	0.930	0.931	1.002
AF221825.1_at	5	0.931	0.800	0.946
AF221825.1_RC_at	7	0.755	1.130	1.241
AF221825.1_RC_x_at	6	0.981	1.004	1.075
AF221825.1_x_at	5	1.098	1.033	1.034
AF221826.1_at	90	**4.206**	**5.699**	**2.261**
AF221826.1_RC_at	6	1.183	1.255	1.297
AF221826.1_RC_x_at	6	1.391	1.234	1.314
AF221827.1_at	6	1.012	0.852	0.799
AF221827.1_RC_at	6	1.086	1.176	1.201
AF221827.1_RC_x_at	7	0.918	1.028	0.966
AF221827.1_x_at	5	0.970	0.888	1.072
AF221828.1_at	7	0.768	0.801	0.785
AF221828.1_x_at	5	0.966	0.805	0.821
AF221829.1_RC_s_at	5	0.961	0.930	1.071
AF221829.1_RC_x_at	7	1.044	0.785	0.953
AF221829.1_s_at	6	1.222	0.941	0.841
AF234186.1_RC_at	7	1.152	0.749	0.817
AF234187.1_RC_s_at	1240	1.201	**0.260**	**0.497**
AF237575.1_RC_at	42	**2.083**	**0.438**	**8.377**
AF250760.1_s_at	174	0.992	0.779	1.123
AF250762.1_RC_at	31	1.570	1.478	1.536
AF250762.1_s_at	259	**3.710**	**3.071**	**2.107**
AF269253.1_s_at	3414	**0.238**	0.532	0.727
AF275840.1_RC_s_at	7	1.864	1.202	1.256
AF275840.1_x_at	17	**2.682**	1.324	0.775
AF275841.1_x_at	8	1.112	0.870	0.869
AF275842.1_at	61	0.333	0.813	**0.412**
AF275842.1_RC_s_at	35	**4.573**	0.819	**4.420**
AF275842.1_RC_x_at	10	1.199	0.625	0.734
AF275842.1_x_at	9	0.882	0.934	0.731
AF275843.1_RC_s_at	14	**2.358**	0.655	0.714
AF275843.1_s_at	8	1.058	0.747	0.715

AF275844.1_x_at	8	1.209	0.706	0.955
AF275845.1_RC_s_at	16	1.199	0.622	1.136
AF275845.1_s_at	5	1.413	1.035	0.931
AF275846.1_RC_x_at	18	1.839	1.051	1.499
AF275846.1_x_at	8	1.375	0.862	0.793
AF275847.1_s_at	6	0.925	1.355	1.000
AF275848.1_at	12	1.899	0.552	0.881
AF275848.1_x_at	21	1.776	0.361	0.771
AF275849.1_RC_at	5	1.007	0.976	1.032
AF275849.1_RC_x_at	6	1.005	1.014	1.068
AF275849.1_x_at	5	0.882	0.887	0.953
AF275850.1_RC_s_at	6	1.349	1.074	1.349
AF275850.1_x_at	10	**2.691**	**3.315**	**2.289**
AF275851.1_RC_x_at	8	0.835	1.073	0.908
AF275851.1_s_at	5	0.958	0.979	1.000
AF275851.1_x_at	5	1.022	0.924	0.987
AF275852.1_RC_at	82	1.257	**0.385**	**0.407**
AF275852.1_RC_x_at	90	1.497	**0.399**	0.629
AF275852.1_x_at	11	1.577	0.567	0.600
AF275853.1_at	7	0.956	0.798	0.684
AF275853.1_RC_at	20	1.937	1.392	1.909
AF275853.1_RC_x_at	18	**2.130**	0.843	**2.350**
AF275853.1_x_at	6	1.002	1.131	0.730
AF275854.1_at	11	**6.184**	**8.967**	0.890
AF275854.1_RC_at	30	**2.446**	0.649	0.959
AF275854.1_x_at	10	**5.393**	**9.445**	1.179
AF275855.1_at	6	0.943	0.878	1.021
AF275855.1_x_at	5	1.105	1.042	1.132
AF275856.1_RC_at	48	1.481	**0.445**	**0.489**
AF275856.1_RC_x_at	33	1.732	0.631	0.689
AF275856.1_x_at	76	1.354	**0.297**	**0.489**
AF275857.1_at	5	0.992	0.855	0.822
AF275857.1_RC_at	17	1.118	1.046	1.027
AF275857.1_RC_x_at	22	1.254	1.127	0.850
AF275857.1_x_at	5	1.019	0.872	0.872
AF286876.1_RC_s_at	10	0.591	0.637	0.848
AF286876.1_s_at	3208	**0.020**	**0.111**	**0.030**
AF288172.1_RC_s_at	20	0.623	0.506	**0.386**
AF288172.1_s_at	220	**0.044**	**0.052**	**0.039**
AF291745.1_RC_at	9	**2.539**	1.077	1.004
AF294425.1_RC_at	85	0.767	0.625	1.150
AF294425.1_RC_s_at	200	0.845	0.918	1.524
AF294425.1_s_at	5629	0.824	0.573	0.710
AF306395.1_RC_at	7	0.811	0.769	0.904
AF306395.1_RC_s_at	6	1.310	0.876	1.044
AF306395.1_RC_x_at	8	0.838	0.824	0.802
AF306395.1_x_at	5	0.979	0.931	0.927
AF306396.1_RC_s_at	56	**2.959**	1.473	1.814
AF306396.1_s_at	5	1.668	1.291	0.943
AF306397.1_x_at	5	0.986	0.852	0.895
AF306398.1_x_at	5	0.994	0.937	0.916
AF306399.1_s_at	7	1.034	1.010	0.851
AF306400.1_RC_at	7	0.879	0.923	0.786
AF306400.1_RC_x_at	5	1.113	1.223	1.228
AF306400.1_x_at	5	1.109	1.072	1.100
AF306401.1_RC_x_at	7	1.020	0.967	1.021
AF306401.1_x_at	7	1.290	1.318	1.119
AF306402.1_RC_at	6	1.012	1.025	0.983
AF306402.1_x_at	6	1.067	0.975	0.898
AF306403.1_RC_at	9	0.818	0.835	0.733
AF306403.1_RC_x_at	9	0.891	0.847	0.818
AF306403.1_x_at	4	0.987	0.974	0.989

AF306404.1_s_at	5	1.093	0.996	1.056
AF306405.1_RC_x_at	8	0.956	0.731	0.839
AF306405.1_x_at	4	1.010	0.974	0.969
AF306406.1_RC_at	8	0.917	0.956	0.950
AF306406.1_RC_x_at	7	0.942	1.188	1.006
AF306406.1_x_at	5	1.121	1.019	0.966
AF306407.1_RC_at	6	1.135	1.328	1.222
AF306407.1_x_at	5	1.054	0.938	0.910
AF306408.1_RC_at	269	0.883	1.196	0.959
AF306408.1_s_at	5	0.963	0.894	1.017
AF306409.1_RC_at	7	1.053	1.088	0.988
AF306409.1_RC_x_at	8	1.487	1.186	0.980
AF306409.1_s_at	5	0.976	0.920	0.909
AF306410.1_RC_at	7	0.807	0.889	0.673
AF306410.1_RC_x_at	7	1.247	0.915	1.094
AF306410.1_s_at	6	1.521	0.884	1.238
AF306411.1_RC_at	5	1.039	1.098	0.996
AF306411.1_s_at	5	1.013	0.848	1.038
AF306412.1_s_at	6	1.117	0.886	0.879
AF306413.1_RC_at	5	1.100	1.309	1.365
AF306413.1_s_at	6	0.930	1.132	0.932
AF306414.1_s_at	5	1.084	1.078	1.033
AF306415.1_RC_at	6	1.011	1.175	1.079
AF306415.1_s_at	5	1.034	0.890	0.972
AF306416.1_s_at	5	1.031	1.117	1.026
AF306417.1_x_at	5	1.063	1.017	1.042
AF306418.1_RC_at	6	1.176	1.139	1.055
AF306418.1_RC_x_at	6	1.086	1.053	1.035
AF306418.1_s_at	5	0.897	0.962	0.901
AF325919.1_RC_s_at	11	0.689	0.650	1.381
AF326354.1_RC_at	7	1.051	1.102	0.899
AF326354.1_s_at	464	**2.211**	**2.566**	1.983
AF332608.1_RC_s_at	10	**2.949**	1.016	**3.120**
AF334803.1_at	5	0.949	0.945	1.020
AF334803.1_RC_at	8	0.900	1.116	0.813
AF334804.1_s_at	5	0.961	0.866	1.007
AF334806.1_x_at	6	1.039	0.969	1.002
AF334955.1_at	5	1.023	1.159	1.262
AF334955.1_RC_at	296	1.457	**0.208**	**0.308**
AF352829.1_s_at	1777	**0.449**	0.621	**0.065**
AF366567.1_at	150	1.493	**0.488**	0.777
AF366567.1_RC_at	8	1.471	1.050	1.190
AF368922.1_at	7	1.067	0.958	1.083
AF368922.1_RC_at	6	1.108	1.001	1.113
AF368922.1_x_at	6	1.112	1.178	1.033
AF368923.1_at	6	1.119	0.869	0.729
AF368923.1_RC_at	24	1.004	1.105	0.878
AF368923.1_RC_x_at	9	0.916	1.257	1.004
AF368923.1_x_at	5	1.115	1.102	0.957
AF368924.1_RC_x_at	18	0.869	0.960	0.942
AF368924.1_x_at	5	1.126	1.076	1.162
AF368925.1_at	82	**0.298**	0.522	**0.314**
AF368925.1_RC_at	133	0.831	1.022	0.840
AF368925.1_RC_x_at	7	1.073	1.026	0.967
AF368925.1_s_at	9	1.433	1.214	1.115
AF368925.1_x_at	24	0.727	0.786	0.704
AF368926.1_at	5	1.064	1.014	1.034
AF368926.1_RC_at	6	1.185	1.145	1.097
AF368926.1_RC_x_at	8	0.765	0.809	0.772
AF368926.1_x_at	5	1.107	1.127	1.044
AF368927.1_at	5	0.893	0.929	0.933
AF368927.1_x_at	5	1.155	0.870	1.009

AF368928.1_at	6	0.952	1.033	0.946
AF368928.1_RC_at	6	1.007	1.059	1.062
AF368929.1_at	20	1.359	1.370	1.067
AF368929.1_RC_at	5	1.067	1.029	1.037
AF368929.1_RC_x_at	6	1.024	1.001	0.993
AF368930.1_at	6	0.949	0.924	1.098
AF368931.1_at	7	0.884	0.842	0.943
AF368931.1_RC_at	13	0.651	0.768	0.856
AF368931.1_RC_x_at	9	0.715	0.968	0.806
AF368932.1_x_at	8	0.788	0.737	0.629
AF368933.1_RC_s_at	5	1.019	1.003	1.002
AF368933.1_RC_x_at	13	1.077	0.722	0.784
AF368933.1_s_at	6	0.877	0.906	0.692
AF368933.1_x_at	9	0.663	0.804	0.805
AF368934.1_at	6	0.947	1.020	1.029
AF368934.1_RC_at	12	1.200	1.161	0.962
AF368935.1_RC_x_at	8	0.899	1.077	0.845
AF368935.1_x_at	5	1.027	0.930	0.971
AF368936.1_s_at	5	1.119	1.361	1.153
AF368937.1_at	9	0.924	0.874	0.875
AF368937.1_RC_at	10	1.503	1.146	0.829
AF368937.1_RC_x_at	9	1.052	0.944	0.912
AF368937.1_x_at	6	0.885	0.822	1.056
AF368938.1_at	5	1.097	0.943	0.921
AF368938.1_RC_at	6	0.915	1.082	1.024
AF368938.1_RC_x_at	6	1.131	1.002	1.073
AF368939.1_at	5	0.991	0.949	0.933
AF368939.1_RC_at	12	0.952	1.009	1.565
AF368939.1_RC_x_at	12	0.890	1.168	1.039
AF368940.1_RC_at	47	1.076	0.823	0.988
AF368940.1_x_at	6	0.895	1.126	1.158
AF368941.1_x_at	5	1.209	0.888	0.881
AF368942.1_at	6	0.921	0.883	0.863
AF368942.1_s_at	6	1.316	1.064	1.111
AF368942.1_x_at	6	1.002	1.158	1.120
AF368943.1_at	7	0.786	0.727	0.696
AF368943.1_RC_at	6	0.862	1.496	0.957
AF368943.1_RC_x_at	6	1.137	0.904	1.274
AF368943.1_x_at	6	1.003	0.911	0.927
AF368944.1_at	4	1.040	1.074	1.140
AF368944.1_RC_at	8	0.870	1.081	1.020
AF368944.1_x_at	5	1.101	0.910	1.101
AF368945.1_x_at	6	0.901	0.834	0.917
AF368946.1_at	26	0.608	0.633	0.739
AF368946.1_x_at	17	0.873	0.896	0.954
AF368947.1_x_at	5	0.958	0.998	0.956
AF368948.1_RC_at	7	1.015	0.878	0.786
AF368948.1_RC_x_at	6	0.989	1.157	1.064
AF368948.1_x_at	5	1.140	1.033	1.181
AF368949.1_x_at	2678	**2.871**	1.901	1.874
AF368950.1_s_at	16	0.787	0.946	0.862
AF378132.1_s_at	214	**0.392**	1.147	1.566
AF378135.1_s_at	9	0.565	0.962	1.433
AF394663.1_s_at	20	1.294	1.144	0.572
AF394664.1_x_at	7	0.947	0.856	0.788
AF394665.1_s_at	5	1.123	1.123	1.078
AF406762.1_RC_at	40	0.558	0.526	**0.323**
AF406762.1_s_at	50	**0.249**	**0.195**	**0.178**
AF411601.1_at	5	1.098	1.292	1.062
AF411601.1_RC_at	45	**2.293**	1.341	1.363
AF411601.1_RC_x_at	12	**3.110**	1.597	1.667
AF416572.1_at	5	1.113	1.223	1.261

AF416572.1_RC_at	10	0.790	1.266	0.998
AF416572.1_x_at	5	1.128	1.073	0.999
AF416573.1_at	6	0.856	0.805	0.876
AF416573.1_RC_at	9	1.000	0.812	0.882
AF416574.1_at	4	0.942	1.061	1.255
AF416574.1_x_at	5	1.158	0.973	0.964
AF416575.1_at	5	1.195	1.048	1.009
AF416575.1_RC_at	9	0.901	0.887	0.984
AF416576.1_at	5	1.105	0.923	1.053
AF416576.1_RC_at	6	1.284	1.133	1.154
AF416576.1_x_at	5	1.159	1.088	1.184
AF416577.1_x_at	6	1.415	1.070	0.796
AF416578.1_at	141	**6.524**	1.770	1.321
AF416578.1_RC_at	20	**2.401**	0.658	0.810
AF416578.1_RC_x_at	18	1.806	0.695	0.566
AF416578.1_x_at	155	**7.561**	**2.281**	1.436
AF416579.1_s_at	8	0.949	0.694	0.705
AF416580.1_RC_s_at	6	1.028	1.167	1.266
AF416580.1_s_at	7	1.047	0.859	0.935
AF416581.1_s_at	7	0.882	0.905	0.762
AF416581.1_x_at	7	1.063	0.893	1.142
AF416582.1_RC_s_at	5	1.198	1.112	1.313
AF416582.1_s_at	8	1.369	1.131	0.853
AF416584.1_at	5	0.962	0.986	0.961
AF416584.1_x_at	5	1.008	0.997	1.033
AF416585.1_at	5	1.062	0.892	1.089
AF416585.1_RC_at	5	1.032	0.987	0.966
AF416585.1_RC_x_at	6	0.970	0.915	0.914
AF416585.1_x_at	5	0.997	0.947	1.002
AF441843.1_at	7	0.814	0.855	0.892
AF441843.1_RC_at	8	0.888	1.052	0.847
AF441843.1_x_at	6	0.985	0.947	1.061
AF441845.1_at	11	**2.089**	**2.491**	1.591
AF441845.1_RC_at	27	**2.673**	1.195	1.219
AF441845.1_RC_x_at	24	**2.344**	1.286	1.730
AF441845.1_s_at	5	1.072	1.085	1.103
AF441845.1_x_at	6	1.430	1.322	1.330
AF441846.1_at	79	**4.906**	**3.996**	**3.770**
AF441846.1_RC_at	58	**2.388**	1.824	1.521
AF441846.1_RC_x_at	35	1.585	1.752	1.648
AF441846.1_s_at	651	**3.876**	**3.603**	1.934
AF441846.1_x_at	121	**4.400**	**3.878**	**3.673**
AF441847.1_at	5	0.996	0.855	0.834
AF441847.1_RC_at	9	1.038	1.132	1.099
AF441847.1_RC_x_at	9	1.067	0.993	1.043
AF441847.1_s_at	621	**4.339**	**3.699**	1.964
AF441847.1_x_at	4	1.075	1.029	1.033
AF461093.1_x_at	6	0.937	0.867	0.838
AF461094.1_RC_x_at	13	0.623	0.555	0.609
AF461094.1_s_at	556	**0.211**	**0.190**	**0.146**
AF461095.1_s_at	40	**0.209**	**0.183**	**0.213**
AF461096.1_s_at	56	**0.463**	**0.470**	**0.175**
AF461097.1_RC_at	35	1.992	1.216	**4.072**
AF461097.1_s_at	6	**11.031**	**2.051**	1.401
AF461098.1_RC_x_at	5	0.921	0.847	0.927
AF461098.1_x_at	7	1.305	1.150	1.174
AF465487.1_s_at	18	1.445	0.451	1.045
AF465490.1_RC_s_at	6	0.911	1.139	1.196
AF465490.1_s_at	5	1.108	0.970	0.911
AF465491.1_s_at	13	**2.766**	0.816	1.041
AF465491.1_x_at	9	1.259	1.024	1.019
AF465492.1_at	6	0.901	0.922	0.736

AF465492.1_RC_at	18	1.090	0.684	0.784
AF465492.1_s_at	7	1.292	1.088	0.906
AF465493.1_RC_s_at	6	1.262	1.169	1.123
AF465493.1_s_at	5	1.000	1.019	0.960
AF465494.1_s_at	15	**2.261**	0.964	0.928
AF465495.1_s_at	18	1.648	0.974	1.030
AF465497.1_s_at	211	**4.894**	**3.581**	**2.193**
AF465497.1_x_at	6	1.083	0.968	0.994
AF465498.1_at	5	1.067	1.077	1.019
AF465498.1_RC_at	6	0.838	1.063	1.064
AF465499.1_RC_s_at	7	1.126	1.038	1.071
AF465499.1_s_at	5	0.886	1.021	0.951
AF465500.1_RC_s_at	6	1.051	0.978	1.014
AF465500.1_s_at	5	0.976	0.894	0.956
AF465501.1_at	4	1.177	1.185	0.968
AF465501.1_RC_at	11	0.872	0.881	0.764
AF465501.1_s_at	6	1.032	0.770	0.931
AF465501.1_x_at	4	1.113	0.971	0.977
AF465502.1_at	6	1.118	0.832	0.941
AF465502.1_RC_at	6	1.204	0.991	1.051
AF465502.1_RC_x_at	6	0.960	0.794	0.986
AF465504.1_s_at	5	0.997	1.036	0.939
AF465505.1_RC_at	5	1.291	1.504	1.110
AF465505.1_RC_x_at	6	0.922	1.221	1.249
AF465505.1_x_at	5	1.109	0.969	0.865
AF465506.1_s_at	230	**4.765**	**3.397**	**2.287**
AF465507.1_s_at	218	**5.157**	**3.561**	**2.174**
AF465507.1_x_at	6	1.235	0.900	0.995
AF465508.1_s_at	241	**5.047**	**3.342**	**2.165**
AF465508.1_x_at	5	1.123	0.956	1.004
AF465509.1_s_at	222	**5.395**	**3.689**	**2.274**
AF465509.1_x_at	5	1.102	1.024	0.957
AF465510.1_RC_s_at	94	**3.265**	**2.252**	1.535
AF465510.1_s_at	208	**4.852**	**3.407**	**2.152**
AF465510.1_x_at	7	2.094	1.789	1.687
AF480451.1_s_at	2174	**0.068**	**0.238**	**0.074**
AF528100.1_RC_s_at	6	1.165	1.092	1.056
AF528100.1_s_at	10	0.899	1.115	1.053
AF528101.1_s_at	13	1.023	1.042	1.045
AF528102.1_s_at	19	0.922	1.066	0.752
AF528103.1_s_at	13	0.992	0.987	0.988
AF528104.1_s_at	10	1.335	1.139	1.108
AF528105.1_s_at	15	0.986	1.004	0.837
AF528106.1_s_at	15	0.810	1.008	0.895
AF528107.1_s_at	10	0.870	1.045	0.928
AF528108.1_s_at	14	0.974	1.082	0.938
AF528109.1_s_at	11	0.918	1.138	1.065
AF528110.1_at	6	0.994	0.889	1.034
AF528110.1_RC_at	7	0.956	1.048	1.013
AF528110.1_RC_x_at	5	1.010	1.452	1.129
AF528110.1_x_at	5	0.889	0.911	0.883
AF528111.1_RC_x_at	6	1.112	1.174	0.872
AF528113.1_RC_x_at	6	1.102	0.957	0.889
AF528113.1_s_at	5	0.932	1.050	1.005
AF528114.1_RC_at	6	1.388	0.905	1.208
AF528114.1_RC_x_at	7	1.149	1.083	1.151
AF528114.1_x_at	8	0.798	0.742	0.794
AF528115.1_at	20	1.140	1.383	1.086
AF528115.1_x_at	12	0.834	0.745	0.757
AF528116.1_RC_at	20	0.732	0.861	0.853
AF528116.1_RC_x_at	15	0.782	0.856	1.108
AF528116.1_x_at	7	1.242	0.906	0.932

AF528117.1_at	6	1.003	0.911	0.879
AF528117.1_RC_at	8	1.269	1.857	1.450
AF528117.1_RC_x_at	9	1.118	1.598	1.251
AF528117.1_x_at	5	1.030	1.062	0.999
AF528118.1_RC_s_at	36	1.676	0.461	0.524
AF528118.1_s_at	164	**2.406**	**0.318**	**0.421**
AF528119.1_at	6	0.885	1.149	0.990
AF528119.1_RC_at	6	0.988	1.067	1.084
AF528120.1_at	5	0.961	0.908	0.977
AF528120.1_RC_at	6	0.987	1.011	0.839
AF528120.1_RC_x_at	5	1.054	1.136	0.986
AF528120.1_s_at	6	0.952	0.764	0.854
AF528121.1_RC_at	6	1.424	1.271	1.039
AF528121.1_RC_x_at	5	1.361	1.330	1.204
AF528121.1_s_at	5	0.915	0.854	0.998
AF528121.1_x_at	7	0.790	0.829	0.790
AF528122.1_at	5	1.147	1.144	1.072
AF528122.1_RC_at	9	0.874	0.954	1.022
AF528122.1_RC_x_at	8	0.943	1.035	1.075
AF528123.1_at	9	1.009	0.802	0.731
AF528123.1_RC_at	12	1.058	1.128	1.220
AF528123.1_RC_x_at	13	1.432	1.550	1.494
AF528123.1_x_at	7	1.233	1.050	1.156
AF528124.1_at	5	1.051	1.208	1.135
AF528124.1_RC_at	9	1.272	1.106	1.083
AF528124.1_RC_x_at	8	1.209	1.402	1.022
AF528124.1_x_at	6	0.950	0.959	1.001
AF528125.1_at	6	1.115	0.772	0.832
AF528125.1_RC_at	5	0.987	0.938	1.066
AF528125.1_RC_x_at	7	0.832	0.821	0.881
AF528126.1_at	5	1.197	1.180	1.004
AF528126.1_s_at	9	1.600	1.080	0.748
AF528126.1_x_at	5	1.081	1.037	1.091
AF528127.1_RC_at	6	1.191	1.472	1.269
AF528127.1_RC_x_at	6	1.317	1.191	1.193
AF528127.1_s_at	9	1.039	0.991	0.916
AF528127.1_x_at	6	1.540	0.923	1.009
AF528128.1_at	6	1.154	1.050	0.744
AF528128.1_RC_at	6	1.041	1.368	1.115
AF528128.1_RC_x_at	7	1.006	1.050	1.342
AF528128.1_x_at	6	1.080	0.993	0.939
AF528129.1_x_at	5	0.987	0.954	0.983
AF528130.1_at	5	1.121	1.031	0.964
AF528130.1_x_at	5	1.030	0.955	0.978
AF528131.1_at	6	0.984	0.859	0.855
AF528131.1_RC_at	18	1.478	0.808	0.766
AF528132.1_RC_x_at	14	0.905	1.195	1.080
AF528132.1_x_at	7	0.890	1.141	1.002
AF528133.1_at	5	1.095	0.986	1.056
AF528133.1_RC_at	5	1.021	1.317	1.259
AF528133.1_RC_x_at	6	0.943	0.949	1.175
AF528133.1_x_at	5	1.007	0.914	0.999
AF528134.1_RC_s_at	13	0.996	1.348	1.203
AF528135.1_at	5	0.985	1.253	1.127
AF528135.1_RC_at	6	1.142	0.980	1.225
AF528136.1_RC_x_at	8	0.940	0.850	1.021
AF528136.1_x_at	5	0.930	0.987	1.011
AF528137.1_x_at	5	0.866	0.926	0.881
AF528138.1_at	6	1.020	0.979	1.086
AF528138.1_RC_at	6	1.032	0.961	1.076
AF528138.1_x_at	5	1.019	0.913	0.902
AF528139.1_RC_s_at	12	1.166	0.718	1.067

AF528139.1_RC_x_at	26	1.501	1.361	1.218
AF528139.1_s_at	6	0.990	0.939	0.990
AF528139.1_x_at	6	1.323	1.233	1.082
AF528140.1_at	5	1.006	0.979	0.903
AF528141.1_RC_at	5	1.024	0.902	1.041
AF528141.1_RC_x_at	6	0.863	0.924	0.923
AF528141.1_x_at	6	1.125	0.873	0.818
AF528142.1_at	5	1.156	1.003	0.996
AF528143.1_RC_x_at	6	1.020	1.043	0.978
AF528143.1_x_at	6	1.081	1.047	0.969
AF528144.1_at	5	1.058	0.990	1.016
AF528144.1_RC_x_at	7	0.874	0.881	0.860
AF528144.1_x_at	5	1.087	0.975	1.103
AF528145.1_x_at	6	0.943	0.853	0.978
AF528146.1_RC_x_at	46	1.034	1.117	1.071
AF528146.1_x_at	7	0.927	0.808	0.676
AF528147.1_at	6	1.314	0.848	0.914
AF528147.1_RC_at	8	1.081	1.064	0.937
AF528148.1_at	5	1.033	1.016	0.998
AF528148.1_x_at	5	1.023	0.999	1.023
AF528149.1_at	4	1.055	0.973	1.090
AF528149.1_RC_at	7	1.032	1.106	1.029
AF528149.1_RC_x_at	8	1.351	1.710	1.228
AF528149.1_x_at	5	1.023	1.073	1.067
AF528150.1_at	5	0.990	0.863	0.962
AF528150.1_RC_at	5	1.065	1.149	1.149
AF528150.1_RC_x_at	6	0.989	0.993	1.030
AF528150.1_x_at	5	0.882	1.025	0.986
AF528151.1_at	6	1.065	1.025	0.917
AF528151.1_RC_at	7	0.834	0.935	0.831
AF528151.1_RC_x_at	6	1.071	1.025	1.025
AF528151.1_x_at	6	0.779	0.820	0.784
AF528152.1_at	7	1.349	0.881	0.892
AF528152.1_x_at	7	1.000	0.827	0.895
AF528153.1_RC_x_at	5	0.998	0.887	0.889
AF528153.1_s_at	5	1.014	0.923	1.061
AF528154.1_at	5	1.058	1.012	1.068
AF528154.1_RC_at	10	0.905	1.125	0.859
AF528155.1_at	12	0.926	0.746	0.802
AF528155.1_RC_at	23	1.100	1.344	0.987
AF528155.1_RC_x_at	16	0.897	1.071	0.960
AF528155.1_x_at	10	0.934	0.940	0.928
AF547155.1_s_at	4	1.033	0.994	0.950
AF547155.1_x_at	7	0.832	0.662	0.845
AF547160.1_RC_s_at	6	1.067	1.008	0.895
AF547160.1_s_at	5	1.031	1.083	1.104
AF547161.1_RC_s_at	7	1.135	1.262	1.596
AF547161.1_s_at	6	1.001	0.972	0.834
AF547162.1_RC_at	11	1.022	1.075	1.084
AF547162.1_RC_s_at	6	1.126	1.367	1.061
AF547162.1_s_at	5	1.012	0.952	0.899
AF547162.1_x_at	6	1.132	1.022	1.094
AF547163.1_RC_s_at	9	0.870	0.849	0.834
AF547163.1_s_at	6	0.963	0.851	0.826
AFFX-Pf-Gapdh-3_at	1415	1.390	1.110	1.818
AFFX-Pf-Gapdh-3_x_at	1399	1.417	1.115	1.643
AFFX-Pf-Gapdh-5_at	566	1.606	0.945	**2.315**
AFFX-Pf-Gapdh-M_at	879	1.575	1.063	**2.109**
AJ005572.1_s_at	621	1.675	1.749	1.796
AJ007940.1_at	6	1.045	1.034	1.081
AJ007940.1_x_at	5	1.464	1.294	1.186
AJ007941.1_at	5	1.069	1.142	0.937

AJ007941.1_x_at	7	1.034	0.828	0.948
AJ007942.1_at	17	**2.022**	1.105	1.237
AJ007942.1_RC_at	12	1.036	0.903	0.727
AJ290918.1_RC_at	40	1.314	1.813	**2.287**
AJ290918.1_s_at	6503	**2.468**	1.992	1.680
AJ290919.1_RC_at	21	0.889	1.442	1.863
AJ290919.1_s_at	2025	**4.520**	**2.614**	1.932
AJ290920.2_s_at	3925	**2.681**	1.967	1.638
AJ290922.1_s_at	187	**2.401**	1.851	1.859
AJ290925.2_at	1995	**3.763**	**3.539**	1.971
AJ290925.2_RC_at	105	**3.343**	**2.846**	**2.151**
AJ290927.1_RC_s_at	35	1.163	1.995	1.818
AJ290927.1_s_at	9379	1.291	0.893	1.184
AJ290928.1_s_at	1667	1.773	1.443	1.617
AJ290929.1_s_at	5395	**2.061**	**2.103**	1.650
AJ290930.1_s_at	1155	**3.642**	**2.950**	**2.610**
AJ290931.1_at	10	1.220	1.557	1.513
AJ290931.1_RC_s_at	623	**2.618**	**3.154**	**2.903**
AJ290931.1_x_at	10	1.322	1.953	1.728
AJ290934.1_RC_at	40	**0.385**	0.770	0.655
AJ290935.1_s_at	2071	1.145	0.726	1.266
AJ290936.1_s_at	9	**0.439**	0.552	0.547
AJ290937.1_RC_at	61	1.121	**0.426**	1.798
AJ290937.1_s_at	1977	**0.271**	**0.334**	**0.342**
AJ290939.1_RC_at	74	1.364	1.196	1.632
AJ290939.1_s_at	167	0.625	0.825	1.482
AJ290940.1_RC_at	24	**0.295**	**0.273**	**0.389**
AJ290940.1_s_at	867	**0.055**	**0.055**	**0.110**
AJ290941.1_RC_s_at	678	1.044	1.264	**2.442**
AJ290941.1_s_at	172	0.636	0.741	1.149
AJ290942.1_RC_at	9	1.229	0.667	0.941
AJ290942.1_s_at	526	**0.393**	0.636	1.025
AJ420379.1_RC_at	10	0.968	1.230	0.808
AJ420379.1_RC_x_at	8	0.916	0.889	0.865
AJ420379.1_x_at	6	1.026	0.893	0.940
AJ420380.1_at	5	1.159	1.092	0.980
AJ420380.1_x_at	5	1.213	0.974	0.867
AJ420381.1_s_at	6	0.989	0.820	0.805
AJ420382.1_at	5	0.840	0.846	0.973
AJ420382.1_x_at	5	1.044	1.062	1.151
AJ420383.1_s_at	25	0.647	0.984	0.798
AJ420384.1_at	4	1.171	1.169	1.227
AJ420384.1_x_at	5	0.987	1.097	1.140
AJ420385.1_at	8	1.059	1.031	1.171
AJ420385.1_RC_at	5	1.147	1.201	1.113
AJ420385.1_RC_x_at	6	1.195	1.164	1.239
AJ420386.1_at	4	1.060	1.050	1.184
AJ420386.1_RC_at	7	1.268	0.915	1.075
AJ420387.1_s_at	10	0.760	1.156	0.973
AJ420388.1_at	5	0.998	1.092	0.994
AJ420388.1_x_at	5	0.964	1.183	1.044
AJ420389.1_at	6	1.163	0.863	0.798
AJ420389.1_x_at	6	1.729	1.305	1.518
AJ420390.1_at	5	0.994	0.953	0.859
AJ420390.1_RC_at	6	1.203	0.936	0.905
AJ420390.1_RC_x_at	7	1.122	1.105	1.182
AJ420390.1_x_at	6	0.977	0.934	0.906
AJ420391.1_at	7	0.992	0.852	0.809
AJ420391.1_x_at	7	1.028	0.782	0.877
AJ420392.1_at	68	1.881	0.970	0.966
AJ420392.1_RC_at	9	0.846	1.004	0.941
AJ420392.1_RC_x_at	7	0.911	0.885	1.054

AJ420392.1_x_at	66	1.122	0.809	0.932
AJ420393.1_at	5	1.028	0.972	1.118
AJ420393.1_x_at	5	1.024	1.055	1.083
AJ420394.1_x_at	5	0.965	0.849	0.945
AJ420395.1_s_at	5	1.099	0.939	1.043
AJ420395.1_x_at	5	1.126	0.971	0.960
AJ420396.1_at	6	0.915	0.895	0.817
AJ420396.1_RC_at	5	0.987	1.021	1.021
AJ420396.1_RC_x_at	6	0.932	1.105	1.039
AJ420396.1_x_at	5	1.059	1.035	1.008
AJ420397.1_at	5	0.954	0.927	0.876
AJ420397.1_x_at	5	1.152	0.927	1.043
AJ420398.1_at	6	0.931	0.978	0.842
AJ420399.1_s_at	5	0.996	1.153	1.040
AJ420400.1_s_at	13	0.675	0.923	0.607
AJ420401.1_x_at	6	1.089	0.971	0.789
AJ420402.1_at	8	**4.249**	**4.292**	**3.039**
AJ420402.1_RC_x_at	15	1.254	1.231	1.134
AJ420402.1_x_at	17	**2.219**	**2.461**	1.401
AJ420403.1_at	6	0.964	0.937	0.900
AJ420403.1_RC_at	5	0.974	1.266	0.929
AJ420403.1_RC_x_at	7	0.890	1.068	1.030
AJ420403.1_x_at	6	0.886	0.975	0.976
AJ420404.1_RC_x_at	64	1.474	**0.141**	**0.238**
AJ420404.1_x_at	9	1.067	1.068	0.789
AJ420405.1_at	4	1.058	0.921	1.075
AJ420405.1_x_at	5	1.094	0.974	1.006
AJ420406.1_at	5	0.969	0.918	0.973
AJ420406.1_x_at	6	0.900	0.953	0.940
AJ420407.1_at	6	0.790	0.800	0.850
AJ420408.1_s_at	11	0.952	1.243	0.919
AJ420409.1_x_at	5	0.975	0.991	1.051
AJ420410.1_at	5	1.113	1.091	0.999
AJ420670.1_RC_at	98	**2.228**	**3.116**	**3.277**
AJ420670.1_RC_x_at	92	1.717	**2.812**	**2.587**
AJ420670.1_s_at	1476	**3.221**	1.738	1.950
AJ420671.1_s_at	10390	1.661	0.755	1.287
AJ493426.1_s_at	464	**3.350**	**2.245**	1.895
AY138256.1_at	4	1.185	1.129	1.186
AY138256.1_RC_x_at	5	0.879	0.871	1.020
AY138256.1_x_at	5	0.868	0.895	0.983
AY138257.1_RC_at	5	0.985	0.821	0.801
AY138257.1_RC_x_at	5	1.057	0.943	1.057
AY138257.1_x_at	5	0.864	0.916	0.907
AY138258.1_RC_at	5	1.020	0.871	1.045
AY138258.1_RC_x_at	7	1.078	0.888	1.186
AY138258.1_x_at	4	1.081	0.987	1.138
AY189720.1_s_at	6	0.940	0.830	0.893
AY208964.1_RC_at	40	0.812	0.689	0.664
AY208964.1_x_at	34	0.608	0.848	0.972
AY208965.1_s_at	324	0.740	1.898	1.314
AY208966.1_RC_at	10	0.916	0.757	0.856
AY208966.1_RC_x_at	9	1.002	0.797	0.745
AY208966.1_s_at	5	1.091	1.032	1.158
AY277506.1_RC_at	157	**0.307**	**0.444**	**0.370**
AY277506.1_s_at	10	0.722	0.702	0.547
AY277507.1_RC_at	423	1.130	1.291	1.521
AY277507.1_RC_x_at	467	0.987	1.249	1.682
AY277507.1_s_at	75	0.731	1.285	**3.388**
J04007.1_RC_at	8	1.860	0.988	1.072
J04007.1_s_at	284	**5.894**	**2.901**	1.616
K03509.1_RC_x_at	308	1.308	0.838	1.178

L12976.1_RC_s_at	6	1.201	1.117	1.053
L12976.1_x_at	5	0.850	0.894	0.965
L12977.1_x_at	5	1.005	1.028	1.093
L12978.1_RC_s_at	6	1.156	1.334	1.112
L12978.1_x_at	8	0.796	0.775	0.768
L12979.1_RC_s_at	7	1.141	0.984	0.922
L12979.1_x_at	6	1.319	0.922	1.087
L29343.1_RC_at	9	0.869	0.792	0.918
L29343.1_s_at	12	0.644	0.619	0.947
L29344.1_RC_s_at	6	1.021	1.210	1.590
L29344.1_s_at	5422	1.367	1.266	1.313
L38450.1_at	26	0.988	0.667	0.755
L38453.1_at	5	1.019	1.322	1.107
L38453.1_x_at	6	1.148	1.076	1.076
L38454.1_RC_at	8	1.280	1.265	1.121
L38455.1_at	6	1.089	0.944	0.978
L38455.1_RC_at	7	1.258	1.113	1.144
L40600.2_s_at	323	1.525	1.423	1.388
L40600.2_x_at	11	0.872	0.888	0.982
L40601.1_s_at	1848	1.193	0.986	0.564
L40602.1_RC_at	16	0.839	0.533	0.613
L40602.1_RC_x_at	48	1.292	**0.265**	**0.344**
L40602.1_s_at	1895	1.113	1.049	0.582
L40603.1_s_at	110	1.484	1.436	0.991
L40604.1_at	107	**2.137**	**0.125**	**0.314**
L40605.1_RC_s_at	385	1.411	**0.287**	**0.334**
L40605.1_s_at	2019	1.193	1.074	0.567
L40605.1_x_at	9	0.996	1.087	0.823
L40606.1_s_at	8	1.202	0.839	1.145
L40607.1_x_at	727	1.626	1.531	0.811
L42245.1_at	18	**10.442**	**5.353**	**0.457**
L42245.1_RC_at	19	**5.216**	1.567	1.350
L42245.1_x_at	68	**6.558**	**3.565**	**0.473**
M10236.1_s_at	7568	**2.155**	1.994	0.858
M10985.1_at	3014	**0.441**	0.525	0.867
M14655.1_s_at	10003	1.495	**1.741**	0.958
M15212.1_s_at	23975	0.690	0.848	0.931
M17530.1_RC_s_at	265	**0.209**	**0.156**	**0.101**
M17530.1_s_at	10476	**0.054**	**0.108**	**0.027**
M18825.1_at	1831	1.796	1.837	1.582
M18825.1_RC_at	699	1.760	1.704	1.610
M18825.1_s_at	371	1.839	1.675	1.636
M18825.1_x_at	1967	1.947	1.876	1.702
M23646.1_RC_at	38	0.559	1.336	1.050
M23646.1_RC_x_at	98	0.647	1.895	1.367
M23646.1_s_at	40132	1.000	1.000	1.004
M24328.1_s_at	409	1.824	**2.384**	1.743
M35727.1_s_at	5	1.025	1.031	1.040
M64106.1_s_at	6	1.328	1.255	1.009
Pf.1.1.0_CDS_at	688	**0.269**	0.583	**0.309**
Pf.1.10.0_CDS_at	1269	1.038	0.861	0.632
Pf.1.101.0_CDS_at	48	0.754	0.900	**2.541**
Pf.1.105.0_CDS_at	5	1.238	1.046	1.119
Pf.1.106.0_CDS_s_at	12	0.828	0.902	0.902
Pf.1.107.0_CDS_at	7	0.997	0.914	0.948
Pf.1.108.0_CDS_at	7	1.242	1.274	1.066
Pf.1.109.0_CDS_at	9	0.946	1.293	1.303
Pf.1.109.0_CDS_x_at	6	1.200	1.176	1.406
Pf.1.11.0_CDS_at	56	**0.232**	**0.187**	**0.172**
Pf.1.110.0_CDS_s_at	9	1.570	1.215	0.800
Pf.1.110.0_CDS_x_at	4	0.998	0.986	0.943
Pf.1.111.0_CDS_x_at	118	1.205	**0.283**	**0.352**

Pf.1.112.0_CDS_at	5	1.062	0.936	0.899
Pf.1.112.0_CDS_s_at	8	1.701	0.947	0.729
Pf.1.114.0_CDS_at	5	1.070	1.157	0.939
Pf.1.115.0_CDS_at	6	0.939	0.975	0.922
Pf.1.116.0_CDS_s_at	45	1.604	0.947	1.101
Pf.1.117.0_CDS_s_at	48	**2.249**	**3.842**	**0.428**
Pf.1.117.0_CDS_x_at	6	0.966	1.570	0.813
Pf.1.118.0_CDS_at	8	0.792	0.716	0.929
Pf.1.12.0_CDS_x_at	4	1.121	1.046	0.986
Pf.1.120.0_CDS_at	9	1.099	1.171	1.151
Pf.1.121.0_CDS_at	5	1.105	1.240	1.067
Pf.1.123.0_CDS_at	7	1.327	1.220	1.016
Pf.1.124.0_CDS_at	35	**2.022**	**8.308**	**5.520**
Pf.1.126.0_CDS_at	86	0.666	**0.457**	**0.462**
Pf.1.128.0_CDS_at	109	1.536	0.847	0.650
Pf.1.129.0_CDS_at	1962	0.776	0.945	1.303
Pf.1.13.0_CDS_at	512	1.405	1.193	1.051
Pf.1.130.0_CDS_at	35	1.428	**0.435**	0.608
Pf.1.131.0_CDS_at	15	0.936	0.871	0.940
Pf.1.132.0_CDS_at	33	1.220	1.812	1.135
Pf.1.133.0_CDS_s_at	6	1.208	1.661	1.264
Pf.1.134.0_CDS_at	6540	1.237	0.797	1.094
Pf.1.136.0_CDS_at	64	1.257	1.995	**2.083**
Pf.1.137.0_CDS_at	21	1.510	**2.767**	**5.632**
Pf.1.138.0_CDS_at	12	1.408	1.017	1.336
Pf.1.14.0_CDS_at	440	1.484	1.437	1.473
Pf.1.143.0_CDS_at	18	0.671	1.386	0.715
Pf.1.144.0_CDS_at	9	1.686	1.527	0.959
Pf.1.145.0_CDS_at	10	**8.462**	**8.106**	0.930
Pf.1.148.0_CDS_at	115	1.175	0.618	1.072
Pf.1.149.0_CDS_at	9	1.039	0.590	0.908
Pf.1.15.0_CDS_at	46	**0.261**	**0.197**	**0.173**
Pf.1.150.0_CDS_at	45	**2.133**	1.322	**0.320**
Pf.1.151.0_CDS_at	10	1.128	1.347	0.641
Pf.1.152.0_CDS_at	36	1.428	**0.322**	0.449
Pf.1.154.0_CDS_at	440	1.427	**0.182**	**0.258**
Pf.1.155.0_CDS_x_at	67	1.298	0.988	0.992
Pf.1.157.0_at	137	**0.360**	**0.124**	**0.096**
Pf.1.16.0_CDS_at	139	0.649	0.675	1.230
Pf.1.162.0_at	65	0.903	1.017	0.528
Pf.1.17.0_CDS_at	340	**0.158**	0.704	1.337
Pf.1.176.0_at	68	0.620	0.887	1.669
Pf.1.18.0_CDS_at	89	**0.329**	**0.384**	1.321
Pf.1.181.0_at	191	1.488	**0.366**	**0.430**
Pf.1.183.0_at	55	1.117	0.750	1.190
Pf.1.2.0_CDS_at	123	**0.296**	**0.473**	**0.374**
Pf.1.20.0_CDS_at	5395	**0.475**	0.561	0.716
Pf.1.21.0_CDS_at	1380	1.007	1.173	1.135
Pf.1.22.0_CDS_at	474	1.170	1.401	1.836
Pf.1.23.0_CDS_a_at	5	0.989	0.880	0.891
Pf.1.23.1_at	6	1.028	1.000	0.979
Pf.1.24.0_CDS_at	595	1.338	1.226	1.371
Pf.1.25.0_CDS_at	6	1.336	0.803	1.376
Pf.1.27.0_CDS_at	249	**0.418**	0.953	0.997
Pf.1.28.0_CDS_at	298	**0.282**	**0.382**	**0.488**
Pf.1.29.0_CDS_at	133	0.842	**0.140**	**0.193**
Pf.1.30.0_CDS_at	19	0.605	1.025	1.174
Pf.1.31.0_CDS_at	6	0.996	1.125	1.259
Pf.1.32.0_CDS_at	4741	0.619	0.616	0.800
Pf.1.33.0_CDS_at	578	0.765	1.337	**2.116**
Pf.1.34.0_CDS_at	19	0.976	0.906	1.513
Pf.1.35.0_CDS_at	366	1.280	1.590	1.690

Pf.1.36.0_CDS_at	11	0.819	0.855	1.103
Pf.1.37.0_CDS_at	1298	1.022	1.044	1.052
Pf.1.38.0_CDS_at	92	**0.365**	**0.407**	0.742
Pf.1.39.0_CDS_at	175	0.517	0.703	0.763
Pf.1.4.0_CDS_at	532	**0.319**	**0.327**	**0.336**
Pf.1.40.0_CDS_at	513	**0.389**	0.690	**0.301**
Pf.1.41.0_CDS_at	23	1.097	0.836	0.803
Pf.1.42.0_CDS_at	19	0.844	1.074	1.018
Pf.1.43.0_CDS_at	88	1.140	0.634	1.894
Pf.1.44.0_CDS_at	574	1.613	1.281	1.368
Pf.1.45.0_CDS_s_at	54	**2.422**	**4.469**	**0.440**
Pf.1.46.0_CDS_at	26	0.517	**0.352**	**0.312**
Pf.1.47.0_CDS_at	2347	0.968	1.571	1.529
Pf.1.47.0_CDS_x_at	2568	0.970	1.543	1.536
Pf.1.48.0_CDS_a_at	4221	0.761	0.563	1.138
Pf.1.49.0_CDS_at	277	0.518	**0.256**	**0.258**
Pf.1.5.0_CDS_at	324	**2.549**	1.510	1.759
Pf.1.50.0_CDS_at	177	1.238	1.340	1.256
Pf.1.51.0_CDS_at	37	0.790	**0.469**	**2.020**
Pf.1.52.0_CDS_at	379	0.820	0.872	1.329
Pf.1.53.0_CDS_a_at	64	0.981	0.915	**2.011**
Pf.1.54.0_CDS_at	44	0.677	**0.388**	0.848
Pf.1.56.0_CDS_at	10	**3.278**	1.421	1.984
Pf.1.56.0_CDS_x_at	5	1.649	1.649	1.742
Pf.1.57.0_CDS_at	1424	1.090	0.742	1.017
Pf.1.58.0_CDS_at	573	1.435	1.017	1.587
Pf.1.59.0_CDS_at	7	0.879	1.000	0.841
Pf.1.6.0_CDS_at	82	**0.497**	0.927	0.963
Pf.1.60.0_CDS_at	7166	1.198	1.140	1.089
Pf.1.61.0_CDS_at	146	**2.654**	1.718	1.382
Pf.1.62.0_CDS_at	511	1.317	0.856	1.537
Pf.1.63.0_CDS_at	7	0.859	1.034	0.922
Pf.1.64.0_CDS_at	5	1.141	1.032	0.969
Pf.1.64.0_CDS_x_at	6	0.983	1.009	0.956
Pf.1.65.0_CDS_s_at	599	1.832	**0.230**	**0.275**
Pf.1.67.0_CDS_s_at	5	1.079	0.937	0.989
Pf.1.68.0_CDS_at	6	1.023	0.951	1.120
Pf.1.69.0_CDS_at	209	0.857	**0.434**	0.758
Pf.1.70.0_CDS_at	6	1.210	0.956	0.978
Pf.1.71.0_CDS_at	106	0.593	**0.486**	1.348
Pf.1.72.0_CDS_at	34	1.389	0.800	**0.460**
Pf.1.73.0_CDS_at	571	**0.442**	0.877	1.449
Pf.1.74.0_CDS_at	22	**2.130**	0.607	0.883
Pf.1.75.0_CDS_at	2292	**0.117**	**0.091**	**0.218**
Pf.1.76.0_CDS_at	193	1.272	1.104	**0.407**
Pf.1.76.0_CDS_x_at	164	1.547	1.162	**0.513**
Pf.1.77.0_CDS_at	168	1.792	**2.077**	**3.491**
Pf.1.79.1_a_at	53	1.209	**2.283**	**2.388**
Pf.1.8.0_CDS_at	38	0.903	1.349	1.660
Pf.1.80.0_CDS_at	11	0.889	0.614	0.927
Pf.1.82.0_CDS_at	2541	**3.487**	**2.244**	**1.584**
Pf.1.83.0_CDS_at	1034	0.844	1.416	0.957
Pf.1.84.0_CDS_at	202	**0.449**	**0.100**	**0.129**
Pf.1.85.0_CDS_at	563	1.633	**2.910**	**2.520**
Pf.1.86.0_CDS_at	120	0.921	1.211	0.907
Pf.1.88.0_CDS_at	465	1.263	1.566	1.858
Pf.1.89.0_CDS_at	15	1.963	0.553	0.888
Pf.1.9.0_CDS_at	386	**0.456**	0.859	1.091
Pf.1.90.0_CDS_s_at	5	1.061	1.154	1.034
Pf.1.91.0_CDS_x_at	5	1.120	0.961	0.979
Pf.1.92.0_CDS_at	38	1.671	0.622	0.740
Pf.1.92.0_CDS_x_at	23	1.582	0.720	0.668

Pf.1.93.0_CDS_at	37	1.569	1.269	1.103
Pf.1.94.0_CDS_at	54	1.945	**0.272**	**0.349**
Pf.1.95.0_CDS_at	12	0.862	0.752	1.404
Pf.1.97.0_CDS_at	6	0.933	0.962	0.737
Pf.1.99.0_CDS_at	75	**7.869**	**6.095**	**4.916**
Pf.10.1.0_CDS_at	405	1.526	0.935	0.538
Pf.10.1.0_CDS_x_at	940	1.069	0.820	**0.407**
Pf.10.10.0_CDS_at	113	**0.121**	**0.171**	**0.090**
Pf.10.10.0_CDS_x_at	47	**0.165**	**0.168**	**0.112**
Pf.10.101.0_CDS_at	4182	1.289	**2.247**	1.494
Pf.10.103.0_CDS_at	777	0.951	0.692	0.853
Pf.10.104.0_CDS_at	53	0.567	**0.484**	1.410
Pf.10.105.0_CDS_at	1321	1.512	1.863	1.533
Pf.10.106.0_CDS_at	22	**2.135**	**2.378**	1.597
Pf.10.107.0_CDS_at	189	**0.255**	**0.154**	**0.295**
Pf.10.108.1_at	67	1.227	1.653	0.997
Pf.10.109.0_CDS_at	10	1.188	0.821	0.765
Pf.10.11.0_CDS_at	62	**2.169**	0.700	**0.398**
Pf.10.112.0_CDS_at	750	**0.393**	**0.170**	**0.256**
Pf.10.116.0_CDS_at	351	0.775	1.252	0.837
Pf.10.117.0_CDS_at	464	**0.251**	**0.497**	1.308
Pf.10.118.0_CDS_at	1668	0.639	0.783	1.045
Pf.10.119.0_CDS_at	293	**0.323**	0.601	1.093
Pf.10.12.0_CDS_at	812	0.837	0.820	0.599
Pf.10.120.0_CDS_at	1253	1.696	**2.632**	**2.217**
Pf.10.121.0_CDS_at	44	1.671	**2.620**	1.970
Pf.10.122.0_CDS_at	14	0.833	0.613	0.755
Pf.10.123.0_CDS_at	26	**0.297**	0.642	1.146
Pf.10.125.0_CDS_at	152	1.871	1.051	1.139
Pf.10.126.0_CDS_at	242	**2.108**	1.905	1.245
Pf.10.127.0_CDS_at	47	0.740	0.907	0.526
Pf.10.128.0_CDS_at	563	**0.132**	**0.173**	**0.093**
Pf.10.129.0_CDS_at	126	**0.433**	0.547	1.345
Pf.10.13.0_CDS_a_at	6872	0.558	1.007	1.026
Pf.10.131.0_CDS_at	33	**2.034**	**2.180**	**3.030**
Pf.10.131.0_CDS_x_at	291	1.446	1.590	1.845
Pf.10.132.0_CDS_at	6	1.282	1.377	1.471
Pf.10.133.0_CDS_at	755	**2.015**	**2.016**	**2.011**
Pf.10.134.0_CDS_at	419	0.883	0.975	**2.267**
Pf.10.135.0_CDS_a_at	370	0.890	0.572	1.065
Pf.10.136.0_CDS_a_at	250	**0.467**	0.719	0.944
Pf.10.137.0_CDS_at	1757	**0.477**	**0.455**	**0.294**
Pf.10.138.0_CDS_at	31	**2.807**	**2.462**	**2.715**
Pf.10.139.0_CDS_a_at	4519	0.820	0.866	0.941
Pf.10.140.0_CDS_a_at	107	1.731	1.200	**2.358**
Pf.10.141.0_CDS_at	86	0.517	0.594	**2.459**
Pf.10.142.0_CDS_at	16	0.899	0.831	1.484
Pf.10.143.0_CDS_at	11	1.066	**2.102**	**2.896**
Pf.10.144.0_CDS_at	19	0.864	1.351	1.130
Pf.10.145.0_CDS_at	68	1.133	1.903	1.358
Pf.10.146.0_CDS_at	126	**0.292**	0.099	0.106
Pf.10.147.0_CDS_at	97	1.434	0.969	1.914
Pf.10.148.0_CDS_at	277	0.502	1.208	1.548
Pf.10.15.0_CDS_at	4013	1.695	1.591	1.169
Pf.10.150.0_CDS_at	15	0.974	1.248	1.090
Pf.10.151.0_CDS_at	26	0.734	0.228	**2.009**
Pf.10.154.0_CDS_at	74	1.292	1.085	**2.458**
Pf.10.155.0_CDS_at	3874	0.712	0.665	0.717
Pf.10.156.0_CDS_at	299	0.657	1.455	**2.036**
Pf.10.157.0_CDS_at	59	0.349	0.242	0.182
Pf.10.159.0_CDS_at	610	**0.133**	0.774	1.658
Pf.10.16.0_CDS_at	270	1.660	1.458	1.421

Pf.10.161.0_CDS_a_at	206	**0.305**	**0.269**	**0.320**	
Pf.10.163.0_CDS_at	26	1.088	0.550	0.804	
Pf.10.164.0_CDS_at	35	1.824	**2.205**	**2.211**	
Pf.10.166.0_CDS_at	614	**0.215**	**0.418**	**0.273**	
Pf.10.168.0_CDS_at	78	0.563	0.810	0.618	
Pf.10.169.0_CDS_at	40	1.721	1.489	1.652	
Pf.10.17.0_CDS_at	3409	**0.070**	**0.065**	**0.024**	
Pf.10.170.0_CDS_at	135	1.516	1.515	**2.655**	
Pf.10.171.0_CDS_at	14	0.873	0.628	1.162	
Pf.10.172.0_CDS_at	12	1.744	0.760	0.766	
Pf.10.173.0_CDS_at	836	**0.135**	**0.169**	**0.043**	
Pf.10.174.0_CDS_at	3085	**0.161**	**0.108**	**0.077**	
Pf.10.177.0_CDS_at	16	0.977	0.862	**2.021**	
Pf.10.178.0_CDS_at	1387	0.836	1.213	**2.221**	
Pf.10.179.0_CDS_at	5	0.942	1.046	1.150	
Pf.10.18.0_CDS_at	436	0.887	0.766	1.217	
Pf.10.18.0_CDS_s_at	761	0.570	0.637	1.036	
Pf.10.18.0_CDS_x_at	294	0.758	0.784	1.400	
Pf.10.180.0_CDS_at	9401	1.552	1.267	1.215	
Pf.10.180.0_CDS_x_at	9271	1.654	1.333	1.275	
Pf.10.181.0_CDS_at	2487	**0.371**	0.562	**0.454**	
Pf.10.184.0_CDS_at	64	**0.282**	0.503	1.445	
Pf.10.185.0_CDS_at	1957	1.660	1.598	1.786	
Pf.10.187.0_CDS_at	32	**0.494**	**0.399**	**0.245**	
Pf.10.188.0_CDS_a_at	59	1.597	1.824	1.076	
Pf.10.19.0_CDS_at	2742	0.832	0.964	0.683	
Pf.10.190.0_CDS_at	626	**2.309**	**2.288**	**2.364**	
Pf.10.192.0_CDS_at	1527	1.790	1.595	1.245	
Pf.10.193.0_CDS_at	104	**0.088**	**0.171**	1.104	
Pf.10.194.0_CDS_at	9	1.021	0.685	0.916	
Pf.10.197.0_CDS_a_at	348	0.691	0.859	**2.740**	
Pf.10.198.0_CDS_at	334	**0.496**	0.688	1.379	
Pf.10.199.0_CDS_at	243	**2.419**	1.741	**2.213**	
Pf.10.2.0_CDS_at	13841	1.237	1.237	1.139	
Pf.10.200.0_CDS_at	69	0.866	1.260	**2.048**	
Pf.10.201.0_CDS_at	65	**0.297**	0.514	1.008	
Pf.10.202.0_CDS_at	386	**0.347**	**0.167**	**0.396**	
Pf.10.203.0_CDS_at	10	1.066	0.725	0.741	
Pf.10.205.0_CDS_at	149	0.611	0.599	1.130	
Pf.10.206.0_CDS_at	1998	**2.416**	**2.416**	**3.169**	
Pf.10.207.0_CDS_at	421	0.826	0.805	1.522	
Pf.10.208.0_CDS_at	15	0.969	0.547	0.656	
Pf.10.209.0_CDS_at	8	0.854	1.119	0.776	
Pf.10.21.0_CDS_at	456	**0.307**	0.582	1.678	
Pf.10.211.0_CDS_at	605	**0.213**	**0.452**	1.314	#1
Pf.10.213.0_CDS_x_at	5	0.943	0.872	0.757	
Pf.10.214.0_CDS_x_at	5	1.403	0.955	0.985	
Pf.10.215.0_CDS_at	6	1.023	1.066	0.959	
Pf.10.216.0_CDS_at	8	1.007	0.831	1.243	
Pf.10.217.0_CDS_at	5	1.302	0.819	1.014	
Pf.10.218.0_CDS_at	42	0.619	**0.286**	1.418	
Pf.10.22.0_CDS_at	146	**3.320**	1.891	**2.471**	
Pf.10.220.0_CDS_at	10	0.811	0.604	1.118	
Pf.10.221.0_CDS_at	9	0.966	0.707	**2.243**	
Pf.10.222.0_CDS_at	76	1.083	**0.439**	**0.474**	
Pf.10.223.0_CDS_at	45	**0.497**	1.091	1.606	
Pf.10.223.0_CDS_x_at	71	**0.414**	1.018	1.718	
Pf.10.224.0_CDS_at	17	**0.435**	**0.328**	**0.360**	
Pf.10.227.0_CDS_at	36	**0.488**	**0.387**	**0.348**	
Pf.10.228.0_CDS_at	230	1.491	1.928	1.964	
Pf.10.229.0_CDS_at	555	0.536	**0.448**	**0.377**	
Pf.10.23.0_CDS_at	20	1.015	0.944	0.768	
Pf.10.231.0_CDS_at	148	0.876	1.073	**2.428**	

Pf.10.232.0_CDS_at	235	0.635	1.415	1.427
Pf.10.233.0_CDS_at	23	**2.331**	**4.390**	**4.622**
Pf.10.234.0_CDS_at	54	1.362	0.942	1.755
Pf.10.235.0_CDS_at	811	0.967	**0.464**	**0.425**
Pf.10.236.0_CDS_at	107	1.240	**0.476**	**0.314**
Pf.10.237.0_CDS_at	150	0.964	1.225	1.788
Pf.10.239.1_at	155	**0.120**	**0.261**	0.545
Pf.10.24.0_CDS_at	57	**0.487**	**0.204**	**0.376**
Pf.10.241.0_CDS_at	325	**0.467**	**0.496**	0.735
Pf.10.242.0_CDS_at	294	**0.055**	**0.038**	**0.046**
Pf.10.243.0_CDS_at	359	1.253	**0.305**	**0.317**
Pf.10.244.0_CDS_at	398	1.398	1.854	1.924
Pf.10.245.0_CDS_at	227	**0.203**	**0.388**	0.513
Pf.10.247.0_CDS_at	105	0.755	1.496	**3.003**
Pf.10.248.0_CDS_at	18	1.144	0.661	0.654
Pf.10.249.0_CDS_at	16	0.617	0.751	1.555
Pf.10.25.0_CDS_at	104	**0.183**	**0.258**	**0.132**
Pf.10.251.0_CDS_at	25	0.944	0.669	0.728
Pf.10.252.0_CDS_at	1343	**0.427**	0.655	1.457
Pf.10.253.0_CDS_at	413	0.808	1.246	1.059
Pf.10.254.0_CDS_at	37	0.673	0.676	1.646
Pf.10.255.0_CDS_at	122	1.252	1.080	1.915
Pf.10.256.0_CDS_at	1017	0.616	**0.425**	0.734
Pf.10.257.0_CDS_at	23	**0.333**	**0.270**	**0.469**
Pf.10.259.0_CDS_at	55	**0.434**	**0.422**	0.814
Pf.10.26.0_CDS_at	14	0.631	0.627	0.556
Pf.10.260.0_CDS_at	15	**10.451**	1.049	0.645
Pf.10.263.0_CDS_at	402	**0.242**	**0.338**	0.579
Pf.10.264.0_CDS_at	93	0.827	0.711	0.658
Pf.10.266.0_CDS_at	1546	0.918	0.874	1.234
Pf.10.268.0_CDS_at	81	1.110	**0.310**	1.164
Pf.10.269.0_CDS_at	365	**2.827**	1.931	**2.226**
Pf.10.27.0_CDS_at	1803	1.368	1.078	1.697
Pf.10.270.0_CDS_at	20	1.103	0.829	1.412
Pf.10.272.0_CDS_at	69	0.712	0.706	1.178
Pf.10.273.0_CDS_at	517	**3.263**	**2.134**	**2.646**
Pf.10.274.0_CDS_at	203	1.537	**2.208**	**2.417**
Pf.10.275.0_CDS_at	30	0.869	1.231	0.703
Pf.10.276.0_CDS_at	230	0.568	0.838	1.454
Pf.10.279.0_CDS_at	10	0.779	0.898	0.970
Pf.10.28.0_CDS_at	111	**0.326**	0.569	0.675
Pf.10.280.0_CDS_at	137	0.867	1.164	1.074
Pf.10.285.0_CDS_at	17	0.949	1.355	0.769
Pf.10.286.0_CDS_at	6	0.999	0.841	0.864
Pf.10.287.0_CDS_at	14	0.863	1.036	**0.413**
Pf.10.289.0_CDS_at	57	1.755	0.762	0.588
Pf.10.29.0_CDS_at	1371	**2.292**	**2.570**	1.779
Pf.10.290.0_CDS_at	7	1.119	1.016	0.903
Pf.10.291.0_CDS_at	16	**10.101**	**4.390**	**5.239**
Pf.10.293.0_CDS_at	186	0.696	0.667	0.633
Pf.10.3.0_CDS_at	3728	0.936	1.033	1.054
Pf.10.30.0_CDS_at	110	**0.255**	**0.152**	**0.184**
Pf.10.301.0_CDS_at	5	1.279	1.013	0.961
Pf.10.302.0_CDS_at	8	1.185	1.062	0.789
Pf.10.303.0_CDS_at	6	0.992	1.085	1.111
Pf.10.304.0_CDS_at	5	1.012	1.104	1.037
Pf.10.305.0_CDS_at	6	0.908	0.971	0.925
Pf.10.306.0_CDS_at	5	1.006	1.173	0.969
Pf.10.307.0_CDS_at	5	1.213	1.146	1.159
Pf.10.308.0_CDS_at	5	0.946	1.038	0.919
Pf.10.309.0_CDS_at	7	0.886	0.742	0.797
Pf.10.31.0_CDS_at	1911	0.814	1.024	1.030

Pf.10.310.0_CDS_at	7	1.160	1.071	1.167
Pf.10.311.0_CDS_at	6	1.019	0.960	1.064
Pf.10.312.0_CDS_at	5	1.068	0.985	0.964
Pf.10.312.0_CDS_x_at	6	0.960	0.843	1.076
Pf.10.313.0_CDS_at	6	0.972	0.843	0.858
Pf.10.314.0_CDS_at	6	0.836	0.893	0.862
Pf.10.315.0_CDS_at	6	1.109	0.921	1.025
Pf.10.318.0_CDS_at	6	1.128	0.793	0.898
Pf.10.319.0_CDS_at	65	**8.802**	1.563	1.066
Pf.10.32.0_CDS_at	4910	1.044	0.784	1.433
Pf.10.321.0_CDS_at	316	**2.212**	**2.788**	1.945
Pf.10.322.0_CDS_at	22	1.614	1.269	**2.355**
Pf.10.324.0_CDS_at	29	0.901	0.851	1.368
Pf.10.324.0_CDS_x_at	115	0.959	0.818	1.460
Pf.10.325.0_CDS_at	34	0.575	0.507	1.034
Pf.10.326.0_CDS_at	2414	1.371	1.425	**2.287**
Pf.10.327.0_CDS_at	8	1.162	1.318	0.876
Pf.10.328.0_CDS_at	854	**2.142**	1.597	1.692
Pf.10.329.0_CDS_at	3780	1.146	0.996	1.344
Pf.10.33.0_CDS_at	139	0.744	1.130	1.782
Pf.10.330.0_CDS_at	17	0.706	**0.469**	0.575
Pf.10.333.0_CDS_at	69	**2.263**	1.921	**3.545**
Pf.10.334.0_CDS_at	4003	1.345	1.260	1.565
Pf.10.335.0_CDS_at	7	1.075	0.817	0.947
Pf.10.337.0_CDS_at	55	0.907	1.115	1.101
Pf.10.338.0_CDS_at	8	0.993	0.693	0.847
Pf.10.339.0_CDS_at	16	**0.439**	**0.462**	1.135
Pf.10.34.0_CDS_a_at	1461	1.364	1.218	0.933
Pf.10.340.0_CDS_at	494	**0.173**	0.712	1.420
Pf.10.341.0_CDS_at	274	**0.417**	0.601	1.881
Pf.10.342.0_CDS_at	388	0.728	1.225	1.295
Pf.10.344.0_CDS_at	7	1.414	0.737	0.869
Pf.10.345.0_CDS_at	315	0.992	1.499	1.776
Pf.10.35.0_CDS_at	17	**0.323**	0.644	0.840
Pf.10.350.0_CDS_at	55	0.998	**2.181**	**0.256**
Pf.10.351.0_CDS_at	386	**0.199**	**0.448**	0.673
Pf.10.352.0_CDS_at	12	1.079	0.735	0.859
Pf.10.353.0_CDS_at	7	0.978	0.882	0.838
Pf.10.354.0_CDS_at	1498	0.835	**0.461**	**0.332**
Pf.10.355.0_CDS_a_at	340	0.773	**0.266**	**0.370**
Pf.10.356.0_CDS_at	33	1.029	**0.313**	**0.241**
Pf.10.357.1_at	17	0.887	0.551	1.167
Pf.10.359.0_CDS_at	87	**3.503**	**0.273**	**0.409**
Pf.10.36.0_CDS_at	338	0.797	**0.429**	**0.427**
Pf.10.360.0_CDS_at	62	0.610	1.145	1.053
Pf.10.362.0_CDS_at	14	1.229	**0.383**	**0.431**
Pf.10.365.0_CDS_at	480	1.760	**0.175**	**0.272**
Pf.10.366.0_CDS_at	85	0.642	0.510	0.506
Pf.10.368.0_CDS_at	21	**2.386**	**0.315**	**0.393**
Pf.10.37.0_CDS_a_at	6094	0.764	1.069	1.013
Pf.10.370.0_CDS_at	189	**2.139**	**2.286**	**2.353**
Pf.10.371.0_CDS_at	375	0.892	0.978	1.867
Pf.10.372.0_CDS_at	38	**0.460**	0.559	0.549
Pf.10.374.0_CDS_a_at	259	**0.336**	**0.350**	**0.278**
Pf.10.375.0_CDS_at	10	0.959	0.762	0.911
Pf.10.377.0_CDS_at	35	1.371	0.963	0.886
Pf.10.378.0_CDS_at	213	**0.474**	1.260	1.136
Pf.10.38.0_CDS_at	178	1.839	1.489	0.698
Pf.10.38.0_CDS_x_at	490	1.263	1.020	**0.411**
Pf.10.380.0_CDS_at	67	1.387	**2.853**	1.988
Pf.10.381.0_CDS_at	31	1.088	0.417	0.623
Pf.10.382.0_CDS_at	62	0.961	0.895	**0.271**

Pf.10.383.0_CDS_at	26	0.921	0.900	1.228
Pf.10.384.0_CDS_at	18	0.597	0.577	0.640
Pf.10.385.0_CDS_at	1477	**0.251**	0.521	0.568
Pf.10.386.0_CDS_at	130	**2.554**	**2.782**	1.916
Pf.10.387.0_CDS_at	6	1.009	1.111	1.298
Pf.10.388.0_CDS_at	149	**0.221**	1.229	0.766
Pf.10.39.2_a_at	2305	0.663	0.658	0.555
Pf.10.390.0_CDS_at	78	**0.226**	**0.287**	**0.284**
Pf.10.392.0_CDS_at	7	0.836	0.830	0.913
Pf.10.394.0_CDS_at	33	**3.473**	**6.915**	0.605
Pf.10.395.0_CDS_at	114	**2.094**	**5.199**	**0.393**
Pf.10.397.0_CDS_x_at	6	0.999	1.113	0.967
Pf.10.398.0_CDS_s_at	116	1.854	1.090	0.523
Pf.10.40.0_CDS_a_at	522	**0.427**	0.767	1.146
Pf.10.406.0_at	190	**0.436**	**0.206**	**0.237**
Pf.10.408.0_at	2520	0.545	0.712	1.144
Pf.10.41.0_CDS_at	111	1.699	**0.269**	**0.428**
Pf.10.413.0_at	251	0.864	0.611	0.788
Pf.10.419.0_at	193	**0.390**	**0.282**	**0.297**
Pf.10.42.0_CDS_a_at	5889	1.579	1.103	1.202
Pf.10.427.0_at	1559	**0.303**	**0.468**	0.916
Pf.10.43.0_CDS_at	611	0.779	1.270	0.915
Pf.10.433.1_a_at	36	0.853	0.791	**2.169**
Pf.10.44.0_CDS_at	742	1.301	1.199	1.853
Pf.10.442.0_at	250	1.755	0.610	0.846
Pf.10.45.0_CDS_a_at	14	1.696	1.457	**2.155**
Pf.10.46.0_CDS_at	891	**0.118**	**0.212**	**0.133**
Pf.10.48.1_a_at	234	**0.451**	0.574	**0.457**
Pf.10.49.0_CDS_at	218	0.992	1.791	**2.586**
Pf.10.5.0_CDS_a_at	7919	1.731	1.690	1.783
Pf.10.503.0_at	61	**0.255**	**0.166**	**0.142**
Pf.10.51.0_CDS_at	258	**0.391**	**0.330**	**0.332**
Pf.10.518.0_at	38	1.000	0.555	1.685
Pf.10.52.0_CDS_at	141	**0.446**	1.156	1.623
Pf.10.528.0_at	7	0.954	0.863	1.005
Pf.10.53.0_CDS_at	26	0.580	0.504	**0.367**
Pf.10.530.0_at	31	**0.265**	**0.248**	**0.263**
Pf.10.54.0_CDS_at	7467	0.652	0.896	0.529
Pf.10.55.0_CDS_at	1035	**0.464**	**0.300**	**0.103**
Pf.10.56.0_CDS_at	296	1.842	1.341	1.857
Pf.10.57.0_CDS_at	339	**0.182**	**0.083**	**0.127**
Pf.10.6.0_CDS_at	5	1.091	1.034	1.184
Pf.10.6.0_CDS_x_at	5	0.925	0.967	0.982
Pf.10.6.0_UTR_at	9	0.808	0.832	0.824
Pf.10.6.0_UTR_x_at	8	0.953	1.075	1.000
Pf.10.61.0_CDS_at	11862	1.117	0.785	1.148
Pf.10.62.0_CDS_at	112	**3.117**	**2.404**	**2.435**
Pf.10.64.0_CDS_at	177	1.386	1.971	**2.744**
Pf.10.65.0_CDS_at	237	0.735	0.626	1.141
Pf.10.67.0_CDS_at	16	1.051	0.816	1.034
Pf.10.68.0_CDS_at	12	0.900	0.473	1.424
Pf.10.69.0_CDS_at	77	**0.266**	**0.226**	**0.382**
Pf.10.7.0_CDS_a_at	9096	0.942	0.629	0.850
Pf.10.70.0_CDS_at	1393	1.007	1.115	1.527
Pf.10.71.0_CDS_at	254	**3.075**	**3.374**	1.942
Pf.10.72.0_CDS_at	1879	**0.140**	**0.380**	**0.098**
Pf.10.73.0_CDS_at	369	1.412	1.695	1.833
Pf.10.74.0_CDS_at	763	1.106	1.870	1.738
Pf.10.76.0_CDS_at	150	0.891	0.947	0.966
Pf.10.77.0_CDS_at	195	1.010	1.172	1.483
Pf.10.78.0_CDS_at	70	0.537	**0.454**	**0.306**
Pf.10.79.0_CDS_at	871	**0.370**	1.183	1.171

Pf.10.8.0_CDS_at	13765	1.128	0.824	1.108
Pf.10.80.0_CDS_s_at	27040	0.686	0.884	0.956
Pf.10.81.0_CDS_at	240	0.643	1.023	0.984
Pf.10.82.0_CDS_at	546	1.820	1.661	**2.308**
Pf.10.83.0_CDS_at	2121	**0.185**	**0.149**	**0.080**
Pf.10.84.0_CDS_at	1329	0.504	0.765	**0.410**
Pf.10.86.0_CDS_at	687	**2.273**	1.982	1.018
Pf.10.87.0_CDS_at	27	0.425	0.464	0.383
Pf.10.88.0_CDS_at	136	1.220	1.842	**2.212**
Pf.10.9.0_CDS_at	4805	0.965	0.907	1.037
Pf.10.90.0_CDS_at	67	**2.342**	1.263	**2.711**
Pf.10.91.0_CDS_at	294	1.242	1.805	1.885
Pf.10.92.0_CDS_at	191	0.726	0.792	1.187
Pf.10.93.0_CDS_at	25	0.527	0.657	**0.478**
Pf.10.94.0_CDS_at	177	0.963	**0.226**	**0.249**
Pf.10.95.0_CDS_at	712	0.704	0.993	1.046
Pf.10.97.0_CDS_at	5	1.725	**2.287**	**2.021**
Pf.10.98.0_CDS_at	201	0.530	**0.205**	**0.287**
Pf.10.99.0_CDS_a_at	39	0.635	**0.226**	**0.442**
Pf.11.1.0_CDS_a_at	3834	0.936	0.941	0.731
Pf.11.10.0_CDS_at	9381	1.276	0.848	1.243
Pf.11.100.0_CDS_at	109	1.049	0.695	**0.398**
Pf.11.101.0_CDS_a_at	1348	**0.362**	0.520	0.791
Pf.11.101.1_CDS_a_at	363	**0.246**	**0.385**	0.724
Pf.11.102.0_CDS_at	139	**2.731**	**2.995**	**2.214**
Pf.11.103.0_CDS_at	217	0.720	1.183	1.515
Pf.11.104.0_CDS_at	49	0.830	0.871	1.134
Pf.11.105.0_CDS_at	395	1.603	1.973	**2.185**
Pf.11.106.0_CDS_a_at	467	0.569	1.158	0.512
Pf.11.108.0_CDS_at	22	1.118	0.946	1.166
Pf.11.109.0_CDS_s_at	5	1.038	0.927	1.004
Pf.11.11.0_CDS_at	21	1.369	0.979	1.182
Pf.11.11.1_CDS_a_at	13	0.868	0.514	0.560
Pf.11.111.0_CDS_s_at	6246	0.724	0.869	1.174
Pf.11.112.0_CDS_at	251	**0.489**	**0.361**	1.359
Pf.11.112.0_CDS_x_at	548	0.511	**0.385**	1.253
Pf.11.113.0_CDS_at	1641	0.926	1.097	1.766
Pf.11.116.0_CDS_at	208	1.788	1.702	1.468
Pf.11.117.0_CDS_at	59	**0.491**	**0.237**	**0.205**
Pf.11.117.1_CDS_a_at	93	**0.406**	**0.300**	**0.266**
Pf.11.118.0_CDS_at	231	**2.561**	1.852	**2.328**
Pf.11.119.0_CDS_at	451	**0.309**	**0.390**	**0.326**
Pf.11.12.0_CDS_at	25703	1.100	1.005	1.216
Pf.11.120.0_CDS_at	66	**0.254**	**0.202**	**0.133**
Pf.11.124.0_CDS_at	38	1.272	1.179	1.583
Pf.11.125.0_CDS_at	10	1.140	0.998	1.004
Pf.11.126.0_CDS_at	177	0.876	0.840	1.015
Pf.11.127.0_CDS_at	124	1.220	1.277	0.614
Pf.11.128.0_CDS_at	229	1.357	1.562	**2.861**
Pf.11.129.0_CDS_at	571	0.586	0.533	0.697
Pf.11.13.0_CDS_at	314	0.548	0.677	0.955
Pf.11.130.0_CDS_at	89	0.505	1.215	0.857
Pf.11.131.0_CDS_at	28	1.206	1.536	0.895
Pf.11.132.0_CDS_at	762	**2.936**	**2.666**	1.984
Pf.11.134.0_CDS_at	1319	1.181	1.301	1.710
Pf.11.135.0_CDS_a_at	7082	1.150	1.006	1.257
Pf.11.137.0_CDS_at	7	0.710	0.699	0.604
Pf.11.137.0_CDS_x_at	11	0.596	0.591	0.638
Pf.11.138.0_CDS_at	606	0.728	1.343	1.223
Pf.11.139.0_CDS_at	3419	0.884	0.907	1.164
Pf.11.14.0_CDS_a_at	62	0.543	**0.163**	**0.401**
Pf.11.140.0_CDS_at	41	0.906	0.992	1.042

Pf.11.144.0_CDS_at	6	1.053	0.812	0.829
Pf.11.145.0_CDS_at	18	0.628	0.512	**0.340**
Pf.11.146.0_CDS_at	122	0.889	**0.255**	0.609
Pf.11.148.0_CDS_at	1487	1.126	1.764	**2.996**
Pf.11.149.0_CDS_at	74	0.728	1.922	0.638
Pf.11.150.0_CDS_at	116	0.604	0.779	1.541
Pf.11.150.0_CDS_x_at	32	0.632	0.889	1.653
Pf.11.151.0_CDS_at	38	**2.409**	**3.186**	0.569
Pf.11.153.0_CDS_at	1590	1.070	0.818	0.963
Pf.11.155.0_CDS_at	74	0.572	0.628	**0.368**
Pf.11.158.0_CDS_at	951	0.833	1.473	1.531
Pf.11.16.0_CDS_at	1068	**0.162**	**0.080**	**0.071**
Pf.11.160.0_CDS_at	407	**2.253**	1.975	**2.324**
Pf.11.163.0_CDS_at	212	**0.246**	**0.405**	**0.323**
Pf.11.164.0_CDS_at	390	**2.108**	1.624	1.746
Pf.11.164.1_CDS_a_at	268	1.604	1.185	1.543
Pf.11.165.0_CDS_at	174	1.476	1.667	**2.125**
Pf.11.166.1_a_at	11	1.624	0.535	0.634
Pf.11.167.0_CDS_at	159	0.547	0.504	0.711
Pf.11.168.0_CDS_at	9	0.996	0.763	0.767
Pf.11.17.0_CDS_at	5932	1.036	1.096	1.261
Pf.11.170.0_CDS_at	7384	1.678	1.165	1.383
Pf.11.171.0_CDS_at	1112	**0.225**	**0.409**	0.715
Pf.11.172.0_CDS_at	3039	**0.366**	0.569	0.781
Pf.11.173.0_CDS_at	935	1.319	1.826	1.167
Pf.11.176.0_CDS_at	782	1.978	**2.849**	**2.314**
Pf.11.177.0_CDS_at	242	**0.280**	0.766	**0.492**
Pf.11.179.0_CDS_at	35	1.370	1.483	1.431
Pf.11.180.0_CDS_at	9	0.718	1.048	1.286
Pf.11.181.0_CDS_at	605	0.954	0.769	1.198
Pf.11.182.0_CDS_at	1981	1.068	1.052	0.692
Pf.11.183.0_CDS_at	101	0.661	**0.420**	0.743
Pf.11.184.0_CDS_at	161	1.223	1.865	1.267
Pf.11.185.0_CDS_at	77	**0.260**	**0.244**	**0.389**
Pf.11.186.0_CDS_at	16	1.308	1.025	0.950
Pf.11.188.0_CDS_at	40	0.907	**0.480**	**0.358**
Pf.11.189.0_CDS_at	226	0.506	0.658	0.792
Pf.11.19.0_CDS_at	628	0.545	0.832	1.545
Pf.11.190.0_CDS_at	81	0.763	1.068	**3.139**
Pf.11.191.0_CDS_at	358	1.382	**2.525**	1.830
Pf.11.192.0_CDS_at	8	0.999	0.838	0.746
Pf.11.195.0_CDS_at	26	1.261	0.870	**0.466**
Pf.11.197.0_CDS_a_at	38	1.005	**2.713**	**2.340**
Pf.11.198.0_CDS_at	52	1.101	**0.315**	**0.192**
Pf.11.199.0_CDS_at	13	1.671	0.731	0.730
Pf.11.2.0_CDS_at	90	**0.312**	**0.145**	**0.153**
Pf.11.201.0_CDS_at	118	1.279	1.019	1.953
Pf.11.203.0_CDS_at	26	0.827	1.395	0.692
Pf.11.204.0_CDS_at	17	0.507	**0.457**	**0.337**
Pf.11.205.0_CDS_at	10	1.027	0.647	0.745
Pf.11.206.0_CDS_at	129	0.811	1.514	**2.062**
Pf.11.207.0_CDS_s_at	6	1.078	0.937	0.866
Pf.11.208.0_CDS_at	76	1.615	**0.226**	**0.136**
Pf.11.209.0_CDS_at	848	1.633	1.329	1.574
Pf.11.21.0_CDS_at	6179	**0.150**	**0.214**	**0.325**
Pf.11.21.0_UTR_at	95	**0.123**	**0.150**	**0.251**
Pf.11.21.0_UTR_x_at	94	**0.125**	**0.165**	**0.282**
Pf.11.210.0_CDS_at	106	0.884	1.734	**2.539**
Pf.11.211.0_CDS_at	237	**0.384**	0.621	1.216
Pf.11.212.0_CDS_at	286	0.809	1.674	**3.008**
Pf.11.215.0_CDS_at	15	0.675	**2.056**	1.043
Pf.11.217.0_CDS_at	13	0.566	0.738	1.985

Pf.11.218.0_CDS_a_at	33	**0.293**	**0.227**	**0.246**
Pf.11.218.2_a_at	18	**0.375**	**0.353**	**0.385**
Pf.11.219.0_CDS_at	30	0.647	1.003	**0.240**
Pf.11.22.0_CDS_at	854	**0.292**	**0.320**	0.585
Pf.11.221.0_CDS_at	1365	0.593	0.678	**0.196**
Pf.11.222.0_CDS_at	1175	1.571	1.619	1.876
Pf.11.223.0_CDS_at	1049	1.302	1.905	1.534
Pf.11.224.0_CDS_at	545	0.801	1.556	**3.080**
Pf.11.225.0_CDS_at	10	1.046	0.957	0.982
Pf.11.226.0_CDS_at	8	1.187	1.243	1.636
Pf.11.227.0_CDS_at	6	1.239	1.031	1.516
Pf.11.227.0_CDS_x_at	54	1.173	0.949	1.444
Pf.11.228.0_CDS_at	12	1.394	**2.001**	**2.136**
Pf.11.229.0_CDS_at	369	1.010	0.853	1.025
Pf.11.23.0_CDS_at	477	0.526	0.823	**0.328**
Pf.11.230.0_CDS_at	738	0.610	**0.394**	**0.451**
Pf.11.231.0_CDS_at	129	**0.448**	**0.304**	**0.508**
Pf.11.232.0_CDS_at	1715	1.922	1.456	1.561
Pf.11.234.1_a_at	194	0.656	0.722	1.394
Pf.11.235.0_CDS_at	1734	1.352	1.282	0.722
Pf.11.236.0_CDS_at	581	0.525	1.045	1.310
Pf.11.237.0_CDS_at	217	**0.147**	**0.142**	**0.077**
Pf.11.238.0_CDS_at	35	0.682	**0.497**	0.985
Pf.11.239.0_CDS_at	7	0.924	0.876	0.838
Pf.11.240.0_CDS_at	1249	**0.302**	0.758	**2.002**
Pf.11.241.0_CDS_at	116	1.365	**2.807**	**2.724**
Pf.11.242.0_CDS_a_at	422	0.858	1.153	1.069
Pf.11.243.0_CDS_at	105	0.781	1.130	**0.287**
Pf.11.244.0_CDS_at	27	1.379	1.699	**2.205**
Pf.11.245.0_CDS_at	34	0.627	**0.186**	**0.290**
Pf.11.246.0_CDS_at	31	**2.145**	**0.288**	0.549
Pf.11.247.0_CDS_at	6	0.818	0.849	0.923
Pf.11.247.0_CDS_x_at	5	1.022	1.113	1.055
Pf.11.248.0_CDS_at	16	1.838	0.546	0.667
Pf.11.248.0_CDS_s_at	35	1.628	**0.368**	**0.429**
Pf.11.249.0_CDS_at	6	1.149	1.101	1.121
Pf.11.25.0_CDS_at	27	**2.212**	**2.759**	1.582
Pf.11.250.0_CDS_at	6	0.985	0.889	0.929
Pf.11.251.0_CDS_at	11	0.969	1.085	1.090
Pf.11.252.0_CDS_x_at	19	0.956	0.828	0.708
Pf.11.255.0_CDS_s_at	5	1.054	1.057	0.990
Pf.11.256.0_CDS_at	15	1.321	1.217	1.020
Pf.11.257.0_CDS_s_at	493	1.671	0.538	1.158
Pf.11.259.0_CDS_at	11	1.056	0.806	1.456
Pf.11.26.0_CDS_at	4938	1.570	1.673	1.521
Pf.11.260.0_CDS_at	34	**4.609**	0.858	0.548
Pf.11.261.0_CDS_at	57	**2.069**	1.207	1.570
Pf.11.262.0_CDS_at	810	0.828	1.214	1.969
Pf.11.264.0_CDS_at	9	1.307	0.768	1.254
Pf.11.265.0_CDS_at	1850	**0.197**	**0.341**	0.914
Pf.11.266.0_CDS_at	9	0.808	0.767	0.954
Pf.11.267.0_CDS_at	45	1.837	1.868	1.876
Pf.11.268.0_CDS_at	109	1.013	0.949	1.804
Pf.11.27.0_CDS_at	1090	**0.071**	**0.068**	**0.045**
Pf.11.270.0_CDS_at	34	1.095	1.924	1.603
Pf.11.273.0_CDS_at	253	0.575	0.622	**0.315**
Pf.11.274.0_CDS_at	129	**0.297**	0.675	**2.188**
Pf.11.275.0_CDS_at	12	0.756	0.548	0.651
Pf.11.276.0_CDS_at	38	0.847	0.828	0.828
Pf.11.278.0_CDS_at	29	**0.472**	0.633	0.966
Pf.11.28.0_CDS_at	7181	0.891	1.071	1.164
Pf.11.281.0_CDS_at	23	0.986	0.539	1.115

Pf.11.282.0_CDS_at	10	0.971	0.541	0.912
Pf.11.283.0_CDS_at	19	0.594	**0.428**	0.730
Pf.11.285.0_CDS_at	125	0.756	0.703	0.984
Pf.11.286.0_CDS_at	38	1.839	**0.383**	0.843
Pf.11.287.0_CDS_at	21	1.112	0.695	0.687
Pf.11.288.0_CDS_at	403	0.530	0.553	0.799
Pf.11.289.0_CDS_at	19	0.556	1.134	1.298
Pf.11.29.0_CDS_at	7	1.106	0.992	0.941
Pf.11.291.0_CDS_at	124	**0.244**	**0.416**	1.327
Pf.11.292.0_CDS_at	899	**0.180**	**0.276**	0.849
Pf.11.293.0_CDS_at	20	1.317	0.788	0.969
Pf.11.294.0_CDS_at	173	0.550	**0.495**	0.663
Pf.11.295.0_CDS_at	6	0.836	0.771	0.939
Pf.11.296.0_CDS_at	299	0.873	1.101	1.272
Pf.11.297.0_CDS_at	1351	**2.463**	**2.685**	1.469
Pf.11.298.0_CDS_at	19	0.840	0.574	0.914
Pf.11.299.0_CDS_at	587	0.527	0.856	1.031
Pf.11.3.0_CDS_a_at	1640	1.287	1.109	1.123
Pf.11.30.0_CDS_at	1525	1.489	1.855	1.716
Pf.11.300.0_CDS_at	23	1.163	0.732	1.214
Pf.11.301.0_CDS_a_at	656	1.306	0.833	1.110
Pf.11.302.0_CDS_at	14	0.664	0.783	1.162
Pf.11.304.0_CDS_at	233	1.613	**2.954**	**2.154**
Pf.11.306.0_CDS_at	41	1.179	0.298	0.324
Pf.11.308.0_CDS_at	22	1.051	0.601	0.465
Pf.11.31.0_CDS_at	1234	**0.099**	**0.117**	**0.246**
Pf.11.310.0_CDS_at	213	**3.323**	**2.969**	1.291
Pf.11.311.0_CDS_at	115	1.222	0.505	**0.374**
Pf.11.312.0_CDS_at	62	1.310	1.964	**2.241**
Pf.11.313.0_CDS_at	47	**2.044**	**2.273**	1.899
Pf.11.314.0_CDS_at	10	1.026	0.733	**2.038**
Pf.11.315.0_CDS_at	47	1.477	1.949	0.877
Pf.11.317.0_CDS_at	13	1.155	1.052	1.310
Pf.11.318.0_CDS_at	282	0.921	0.926	1.420
Pf.11.319.0_CDS_at	12	1.968	**4.834**	1.671
Pf.11.32.0_CDS_at	47	**0.426**	**0.181**	**0.154**
Pf.11.321.0_CDS_at	80	0.763	0.774	1.649
Pf.11.322.0_CDS_at	36	0.974	0.595	0.924
Pf.11.323.0_CDS_at	325	0.630	1.204	1.817
Pf.11.325.0_CDS_at	2062	0.843	0.984	1.540
Pf.11.326.0_CDS_at	681	0.831	1.157	1.498
Pf.11.327.0_CDS_at	16	1.228	0.900	1.524
Pf.11.328.0_CDS_at	12	0.544	0.665	1.537
Pf.11.329.0_CDS_at	12	1.321	1.174	1.048
Pf.11.33.0_CDS_at	855	**0.353**	0.767	1.190
Pf.11.330.0_CDS_at	479	1.669	**2.030**	1.465
Pf.11.331.0_CDS_at	9	0.953	0.823	0.974
Pf.11.333.0_CDS_at	27	1.284	0.898	1.155
Pf.11.334.0_CDS_at	190	0.572	**2.153**	0.814
Pf.11.336.0_CDS_at	14	0.902	0.631	0.858
Pf.11.337.0_CDS_at	181	1.839	1.816	**2.475**
Pf.11.338.0_CDS_at	238	1.221	1.803	1.395
Pf.11.34.0_CDS_at	2439	**0.185**	**0.320**	**0.488**
Pf.11.340.0_CDS_at	116	**0.174**	**0.100**	**0.373**
Pf.11.341.0_CDS_at	9	1.179	0.736	0.830
Pf.11.345.0_CDS_at	6	0.894	1.062	**3.844**
Pf.11.346.0_CDS_at	68	1.623	1.175	1.504
Pf.11.35.0_CDS_at	238	**2.021**	1.373	1.400
Pf.11.350.0_CDS_at	35	**2.121**	**0.427**	**0.460**
Pf.11.351.0_CDS_at	48	0.950	0.618	1.009
Pf.11.354.0_CDS_at	21	**0.329**	1.124	**0.463**
Pf.11.355.0_CDS_at	877	0.891	1.591	1.736
Pf.11.358.0_CDS_at	25	1.500	**2.043**	**4.982**

Pf.11.359.0_CDS_at	97	**0.150**	**0.277**	1.073
Pf.11.36.0_CDS_at	32	**0.435**	**0.286**	**0.204**
Pf.11.364.0_CDS_at	4329	1.446	1.834	**0.434**
Pf.11.365.0_CDS_x_at	5	1.031	0.991	0.932
Pf.11.366.0_CDS_s_at	13	0.894	**0.493**	0.703
Pf.11.368.0_CDS_at	759	**2.330**	**2.403**	1.605
Pf.11.369.0_CDS_at	624	1.269	1.344	**2.200**
Pf.11.37.0_CDS_at	1212	1.145	1.155	0.717
Pf.11.370.0_CDS_at	35	**4.809**	**2.450**	1.060
Pf.11.371.0_CDS_at	27	**2.332**	1.754	1.962
Pf.11.373.0_CDS_at	5	1.066	0.950	1.087
Pf.11.374.0_CDS_s_at	6	1.074	1.183	1.327
Pf.11.375.0_CDS_at	6	0.959	1.018	1.071
Pf.11.376.0_CDS_x_at	22	**4.537**	1.448	0.585
Pf.11.38.0_CDS_at	7583	0.679	0.749	0.646
Pf.11.380.0_CDS_at	358	**2.407**	**0.019**	**0.052**
Pf.11.381.0_CDS_x_at	8	0.859	0.924	0.755
Pf.11.386.0_CDS_at	5	1.122	1.070	0.887
Pf.11.387.0_CDS_at	8	1.365	1.213	1.007
Pf.11.388.0_CDS_at	5	1.126	0.997	1.070
Pf.11.39.0_CDS_at	111	0.618	1.264	1.615
Pf.11.394.0_CDS_at	19225	1.484	0.850	1.105
Pf.11.395.0_CDS_at	17	**2.918**	0.877	0.671
Pf.11.396.0_CDS_at	22	1.311	0.592	0.908
Pf.11.397.0_CDS_at	366	**2.041**	1.254	**2.082**
Pf.11.398.0_CDS_at	4334	0.692	0.904	0.746
Pf.11.399.0_CDS_at	18	1.371	1.382	0.917
Pf.11.4.0_CDS_at	2519	0.830	1.109	1.207
Pf.11.40.0_CDS_at	1029	1.357	1.118	1.633
Pf.11.400.0_CDS_at	6	0.792	0.806	1.389
Pf.11.401.0_CDS_at	2631	0.714	1.060	1.850
Pf.11.402.0_CDS_at	1086	**0.471**	0.612	1.266
Pf.11.404.0_CDS_at	1768	1.831	**2.287**	1.891
Pf.11.405.0_CDS_at	223	0.628	0.890	1.680
Pf.11.406.0_CDS_at	408	1.198	0.769	1.042
Pf.11.409.0_CDS_a_at	482	**0.458**	**0.275**	**0.367**
Pf.11.410.0_CDS_at	8	0.802	0.830	1.702
Pf.11.411.0_CDS_at	168	0.879	**2.050**	**2.455**
Pf.11.413.0_CDS_at	12	0.926	**0.443**	0.662
Pf.11.415.0_CDS_at	57	1.423	1.070	1.106
Pf.11.416.0_CDS_at	976	**0.111**	**0.167**	**0.321**
Pf.11.417.0_CDS_at	12	1.334	1.155	1.537
Pf.11.418.0_CDS_at	92	1.088	1.114	1.364
Pf.11.42.0_CDS_at	4101	1.168	1.002	1.173
Pf.11.421.0_CDS_at	9	0.844	0.783	0.834
Pf.11.422.0_CDS_at	144	**7.350**	**5.854**	1.179
Pf.11.423.0_CDS_at	16	1.695	0.762	1.790
Pf.11.424.0_CDS_at	662	**3.221**	**2.362**	1.913
Pf.11.425.0_CDS_at	7315	1.085	0.831	1.020
Pf.11.426.0_CDS_at	444	**0.330**	**0.073**	**0.335**
Pf.11.427.0_CDS_at	135	**2.236**	**2.523**	1.359
Pf.11.428.0_CDS_at	66	1.129	1.536	**2.367**
Pf.11.429.0_CDS_at	68	0.971	0.823	**0.464**
Pf.11.43.0_CDS_at	54	0.961	1.466	0.703
Pf.11.430.0_CDS_at	466	0.842	1.479	1.481
Pf.11.431.0_CDS_at	66	0.677	0.611	**0.418**
Pf.11.432.0_CDS_at	624	**3.289**	1.721	1.758
Pf.11.433.0_CDS_at	804	1.006	1.780	1.520
Pf.11.434.0_CDS_at	654	0.737	1.200	1.473
Pf.11.435.0_CDS_at	35	0.901	1.873	**3.917**
Pf.11.436.0_CDS_at	83	1.088	0.770	1.390
Pf.11.437.0_CDS_at	80	1.258	0.957	0.727

Pf.11.44.0_CDS_a_at	528	1.101	1.395	1.757
Pf.11.44.0_CDS_x_at	353	1.319	1.532	1.981
Pf.11.442.0_CDS_at	11	0.854	0.832	0.723
Pf.11.443.0_CDS_at	120	**0.142**	**0.161**	**0.242**
Pf.11.444.0_CDS_at	7	2.300	1.712	1.506
Pf.11.445.0_CDS_at	95	**0.188**	**0.157**	**0.113**
Pf.11.446.0_CDS_at	87	0.974	1.258	1.807
Pf.11.447.0_CDS_at	38	0.547	1.268	**3.524**
Pf.11.448.0_CDS_at	15	0.837	0.688	1.158
Pf.11.449.0_CDS_at	349	1.147	1.280	**2.388**
Pf.11.45.0_CDS_at	4405	**0.058**	**0.081**	**0.397**
Pf.11.451.0_CDS_at	53	1.480	0.592	**0.249**
Pf.11.452.0_CDS_at	37	1.319	0.688	**2.066**
Pf.11.454.1_CDS_a_at	93	0.561	1.130	1.693
Pf.11.456.0_CDS_at	36	0.788	**0.288**	1.120
Pf.11.457.0_CDS_at	25	1.323	0.599	**0.355**
Pf.11.458.0_CDS_at	439	1.620	**0.364**	**0.449**
Pf.11.459.0_CDS_at	76	**2.213**	1.302	0.544
Pf.11.46.0_CDS_at	215	**2.525**	**3.214**	1.961
Pf.11.460.0_CDS_at	493	**2.469**	**2.394**	**2.800**
Pf.11.461.0_CDS_at	338	0.610	**0.229**	1.015
Pf.11.462.0_CDS_at	23	0.808	0.936	1.940
Pf.11.463.0_CDS_at	16	0.719	0.726	0.600
Pf.11.464.0_CDS_at	1684	**0.250**	**0.363**	**0.373**
Pf.11.465.0_CDS_at	89	**0.395**	0.557	1.592
Pf.11.466.0_CDS_at	32	**1.926**	**2.114**	0.441
Pf.11.468.0_CDS_at	163	1.327	1.223	1.342
Pf.11.469.0_CDS_a_at	75	**0.481**	0.571	1.168
Pf.11.469.0_CDS_x_at	395	**0.421**	0.511	1.322
Pf.11.469.1_a_at	68	**0.422**	**0.469**	1.380
Pf.11.470.0_CDS_at	3510	1.265	1.966	1.263
Pf.11.472.0_CDS_a_at	230	1.409	**2.232**	**0.523**
Pf.11.473.0_CDS_at	28	0.904	**0.495**	**0.337**
Pf.11.475.0_CDS_at	2864	1.662	1.203	1.902
Pf.11.476.0_CDS_at	42	1.496	**0.288**	**0.352**
Pf.11.477.0_CDS_at	193	1.278	0.795	1.063
Pf.11.479.0_CDS_at	432	**0.104**	**0.167**	**0.330**
Pf.11.480.0_CDS_at	13	0.977	0.972	0.736
Pf.11.481.0_CDS_at	543	**2.269**	**2.584**	1.911
Pf.11.482.0_CDS_at	609	**3.069**	**3.601**	**2.662**
Pf.11.483.1_at	10	1.040	0.702	0.978
Pf.11.487.0_CDS_at	8	**7.656**	**4.108**	0.931
Pf.11.488.0_CDS_a_at	1103	**0.193**	**0.253**	**0.320**
Pf.11.488.0_CDS_x_at	314	**0.231**	**0.247**	**0.318**
Pf.11.489.0_CDS_at	229	0.956	0.975	0.508
Pf.11.490.0_CDS_at	421	**0.336**	**0.443**	1.356
Pf.11.491.0_CDS_at	2033	0.924	0.613	0.823
Pf.11.492.0_CDS_at	6	1.170	0.809	0.942
Pf.11.493.0_CDS_at	6	0.879	0.919	0.919
Pf.11.494.0_CDS_at	6	0.978	0.918	0.905
Pf.11.495.0_CDS_at	6	0.996	0.893	0.772
Pf.11.495.0_CDS_s_at	39	1.576	**0.292**	**0.438**
Pf.11.499.0_at	17	0.742	0.955	1.439
Pf.11.50.0_CDS_at	263	1.745	**2.067**	**2.316**
Pf.11.508.0_at	7	1.273	0.940	0.912
Pf.11.512.0_at	280	**0.145**	**0.357**	1.038
Pf.11.521.0_at	91	0.791	**0.361**	1.090
Pf.11.53.0_CDS_at	2181	0.533	0.708	**0.401**
Pf.11.53.0_UTR_at	7	0.872	0.833	0.851
Pf.11.53.0_UTR_x_at	13	0.522	0.596	0.600
Pf.11.54.0_CDS_at	1398	0.715	1.085	1.424
Pf.11.543.0_at	759	1.329	1.169	1.146

Pf.11.548.0_at	95	**0.254**	0.812	1.693
Pf.11.55.0_CDS_at	52	1.394	1.638	**2.463**
Pf.11.56.0_CDS_at	3810	0.624	0.942	1.592
Pf.11.564.0_at	277	1.089	**2.491**	**2.371**
Pf.11.569.0_at	27	1.897	0.671	0.586
Pf.11.57.0_CDS_at	2061	0.832	1.071	1.322
Pf.11.59.0_CDS_at	47	**2.309**	**2.481**	**2.575**
Pf.11.6.0_CDS_at	3404	1.105	0.944	1.157
Pf.11.60.0_CDS_at	1851	1.729	1.418	1.672
Pf.11.61.0_CDS_at	207	**0.241**	0.536	**0.293**
Pf.11.616.0_at	18	0.934	0.603	0.813
Pf.11.618.0_at	26	1.612	0.680	**2.589**
Pf.11.619.0_at	709	**3.212**	**3.652**	1.720
Pf.11.62.0_CDS_at	445	0.589	0.615	1.469
Pf.11.63.0_CDS_at	8515	1.448	1.090	1.358
Pf.11.64.0_CDS_at	50	0.908	**0.221**	0.694
Pf.11.66.0_CDS_at	8144	0.916	0.920	1.049
Pf.11.66.0_UTR_at	3488	**0.387**	0.758	0.889
Pf.11.66.0_UTR_x_at	729	**0.370**	0.807	0.848
Pf.11.68.0_CDS_at	281	1.219	0.722	**0.171**
Pf.11.69.0_CDS_a_at	924	0.511	**0.434**	**0.283**
Pf.11.7.0_CDS_at	57	0.987	0.782	0.788
Pf.11.71.0_CDS_s_at	29	1.391	0.671	0.590
Pf.11.73.0_CDS_at	1699	**0.111**	**0.408**	0.575
Pf.11.74.0_CDS_at	102	0.965	1.565	1.935
Pf.11.75.0_CDS_at	778	0.556	1.572	0.798
Pf.11.76.0_CDS_at	210	1.087	1.769	**0.304**
Pf.11.77.0_CDS_at	25	0.632	0.768	0.695
Pf.11.78.0_CDS_at	7	0.993	0.892	0.974
Pf.11.79.0_CDS_at	721	1.634	1.990	**2.377**
Pf.11.8.1_a_at	354	1.291	1.174	1.161
Pf.11.80.0_CDS_a_at	3931	0.637	0.572	0.664
Pf.11.82.0_CDS_a_at	215	1.420	1.381	1.287
Pf.11.83.0_CDS_at	22	1.186	0.510	1.316
Pf.11.85.0_CDS_at	11	1.209	**2.585**	0.846
Pf.11.86.0_CDS_at	31	**2.413**	1.656	0.568
Pf.11.87.0_CDS_at	239	1.444	1.748	1.362
Pf.11.88.0_CDS_at	8	0.738	0.730	0.630
Pf.11.89.0_CDS_at	1834	0.540	**0.498**	1.156
Pf.11.90.0_CDS_at	60	0.685	0.939	1.861
Pf.11.92.0_CDS_at	55	0.863	1.279	0.942
Pf.11.93.0_CDS_a_at	67	0.874	**0.457**	0.603
Pf.11.94.0_CDS_a_at	953	1.351	1.716	**2.415**
Pf.11.95.0_CDS_at	415	1.300	**2.226**	**2.012**
Pf.11.96.0_CDS_at	427	1.239	1.139	1.112
Pf.11.98.0_CDS_at	26	**2.560**	0.935	1.077
Pf.11.99.0_CDS_at	393	1.644	0.693	1.019
Pf.12.1.0_CDS_at	19504	**0.114**	**0.417**	**0.063**
Pf.12.10.0_CDS_at	1027	0.745	0.980	1.145
Pf.12.100.0_CDS_at	16	**0.420**	0.523	0.935
Pf.12.101.0_CDS_at	686	1.551	0.662	0.743
Pf.12.102.0_CDS_at	234	**0.482**	0.718	0.757
Pf.12.103.0_CDS_at	95	1.167	**2.348**	**2.466**
Pf.12.104.0_CDS_at	622	0.545	1.306	0.668
Pf.12.105.0_CDS_at	16	0.532	0.474	0.663
Pf.12.107.0_CDS_at	87	1.217	1.171	0.921
Pf.12.108.0_CDS_at	1051	**0.267**	**0.386**	**0.326**
Pf.12.109.0_CDS_at	99	0.516	0.912	1.482
Pf.12.110.0_CDS_at	91	1.847	**3.184**	**4.207**
Pf.12.111.0_CDS_at	73	1.081	1.889	**2.193**
Pf.12.112.0_CDS_at	237	0.941	1.427	1.823
Pf.12.114.0_CDS_at	87	**0.433**	**0.305**	**0.318**

Pf.12.115.0_CDS_at	667	0.677	1.497	1.537
Pf.12.117.0_CDS_at	54	0.949	0.628	1.232
Pf.12.118.0_CDS_at	13	0.829	1.017	**2.083**
Pf.12.119.0_CDS_at	753	1.096	1.537	1.679
Pf.12.12.0_CDS_at	2708	1.757	1.797	**2.072**
Pf.12.120.0_CDS_at	1018	**0.202**	**0.258**	**0.176**
Pf.12.121.0_CDS_at	1053	1.032	1.007	0.648
Pf.12.122.0_CDS_at	278	1.901	**2.439**	**2.627**
Pf.12.123.0_CDS_at	161	1.184	**2.411**	1.102
Pf.12.124.0_CDS_at	71	1.260	0.728	1.467
Pf.12.125.0_CDS_at	734	1.841	1.923	1.903
Pf.12.126.0_CDS_at	137	**0.366**	**0.233**	**0.307**
Pf.12.127.0_CDS_at	179	**0.217**	**0.137**	**0.251**
Pf.12.129.0_CDS_at	76	**0.451**	0.575	**2.129**
Pf.12.13.0_CDS_at	11570	0.606	0.692	0.902
Pf.12.130.0_CDS_at	8	0.771	0.647	0.803
Pf.12.131.0_CDS_at	33	1.656	0.686	1.064
Pf.12.132.0_CDS_at	14	0.549	0.598	0.514
Pf.12.133.0_CDS_at	14	**0.486**	0.520	**0.432**
Pf.12.135.0_CDS_at	55	**0.421**	0.704	0.637
Pf.12.136.0_CDS_at	25	0.994	0.902	0.708
Pf.12.138.0_CDS_at	13	0.561	**0.494**	0.521
Pf.12.139.0_CDS_at	39	**0.388**	0.529	0.844
Pf.12.14.0_CDS_a_at	308	1.129	1.308	1.636
Pf.12.140.0_CDS_at	396	1.322	1.547	**2.362**
Pf.12.141.0_CDS_at	975	**0.114**	**0.160**	0.532
Pf.12.142.0_CDS_at	12	0.534	0.653	1.045
Pf.12.143.0_CDS_at	285	1.411	**2.251**	**2.028**
Pf.12.144.0_CDS_at	15	1.122	0.638	0.547
Pf.12.145.0_CDS_at	4200	**0.284**	0.665	**0.117**
Pf.12.146.0_CDS_at	9	0.966	1.092	1.487
Pf.12.147.0_CDS_at	10	0.925	0.671	0.710
Pf.12.148.0_CDS_at	7241	1.653	1.262	1.413
Pf.12.149.0_CDS_at	1639	**2.084**	1.665	1.104
Pf.12.15.0_CDS_at	387	0.788	1.780	1.052
Pf.12.150.0_CDS_at	61	**12.152**	**18.57**	**0.294**
Pf.12.152.0_CDS_at	184	0.675	0.778	1.619
Pf.12.154.0_CDS_at	338	**2.851**	**2.149**	1.872
Pf.12.157.0_CDS_a_at	97	**0.297**	0.734	0.814
Pf.12.158.0_CDS_at	490	**0.050**	**0.312**	**0.315**
Pf.12.159.0_CDS_at	1745	0.785	1.247	**2.192**
Pf.12.16.0_CDS_at	1952	0.600	1.012	0.934
Pf.12.161.0_CDS_at	111	**2.624**	0.744	1.774
Pf.12.162.0_CDS_at	181	0.913	1.273	**2.177**
Pf.12.163.0_CDS_at	1597	1.308	1.583	1.417
Pf.12.165.0_CDS_at	4918	1.590	1.190	1.249
Pf.12.167.0_CDS_at	148	1.048	**2.330**	**2.182**
Pf.12.169.0_CDS_at	14	1.195	0.630	0.608
Pf.12.17.0_CDS_at	2944	1.890	1.359	1.490
Pf.12.17.0_UTR_at	515	**2.616**	1.965	1.715
Pf.12.17.0_UTR_x_at	1489	**2.207**	1.718	1.456
Pf.12.170.0_CDS_at	633	0.996	1.289	**0.369**
Pf.12.171.0_CDS_at	2212	**0.442**	0.619	0.988
Pf.12.173.0_CDS_at	385	1.612	1.876	**2.331**
Pf.12.174.0_CDS_at	99	1.245	1.070	1.191
Pf.12.175.0_CDS_at	10	0.753	0.780	0.714
Pf.12.176.0_CDS_at	2233	0.794	0.945	0.895
Pf.12.179.0_CDS_at	1956	**0.328**	**0.263**	0.542
Pf.12.18.0_CDS_at	1212	0.587	0.581	0.995
Pf.12.182.0_CDS_at	594	**0.372**	0.723	0.926
Pf.12.183.0_CDS_at	23	0.534	**0.492**	0.508
Pf.12.184.0_CDS_at	388	1.901	**2.366**	1.838

Pf.12.185.0_CDS_at	6	1.004	0.856	0.998
Pf.12.186.0_CDS_at	87	0.812	1.792	**2.968**
Pf.12.186.0_CDS_x_at	118	0.879	1.845	**2.596**
Pf.12.188.0_CDS_at	81	0.920	1.686	1.509
Pf.12.189.0_CDS_at	29	1.218	0.727	0.964
Pf.12.19.0_CDS_at	531	1.593	1.536	1.285
Pf.12.190.0_CDS_at	617	**3.770**	**2.997**	**2.222**
Pf.12.191.0_CDS_at	1102	1.675	**2.488**	**2.195**
Pf.12.193.0_CDS_at	14	0.913	1.015	0.893
Pf.12.195.0_CDS_at	663	0.742	1.104	1.069
Pf.12.196.0_CDS_a_at	119	0.749	1.155	0.988
Pf.12.196.1_a_at	215	0.607	0.910	0.862
Pf.12.197.0_CDS_at	5	0.884	1.005	1.667
Pf.12.197.0_CDS_x_at	6	1.173	1.069	1.422
Pf.12.198.0_CDS_at	1643	**0.027**	**0.046**	**0.018**
Pf.12.199.0_CDS_at	309	**2.661**	1.920	**2.375**
Pf.12.2.0_CDS_a_at	5274	**0.181**	**0.283**	**0.137**
Pf.12.20.0_CDS_at	85	1.426	1.210	**2.106**
Pf.12.200.0_CDS_at	421	**0.109**	**0.141**	**0.625**
Pf.12.200.1_a_at	1204	**0.123**	**0.160**	**0.467**
Pf.12.202.0_CDS_at	236	**2.432**	1.909	**3.776**
Pf.12.203.0_CDS_at	184	1.015	1.073	1.897
Pf.12.204.0_CDS_at	625	0.944	1.002	**2.045**
Pf.12.205.0_CDS_at	12	0.580	0.500	**0.498**
Pf.12.207.0_CDS_at	17	0.579	0.666	1.193
Pf.12.208.0_CDS_at	37	**4.352**	**3.612**	**3.965**
Pf.12.209.0_CDS_at	428	**0.254**	0.798	0.821
Pf.12.21.0_CDS_at	42	1.593	1.290	1.072
Pf.12.210.0_CDS_at	139	1.682	1.168	0.872
Pf.12.211.0_CDS_at	235	1.110	1.735	**2.391**
Pf.12.212.0_CDS_at	66	0.508	0.671	**0.291**
Pf.12.213.0_CDS_at	82	**0.293**	**0.466**	0.550
Pf.12.214.0_CDS_at	7	0.858	0.796	1.078
Pf.12.215.0_CDS_at	25	0.952	1.289	1.738
Pf.12.216.0_CDS_at	144	1.665	1.242	1.849
Pf.12.217.0_CDS_at	45	1.027	**0.430**	1.168
Pf.12.22.0_CDS_at	5534	1.106	0.901	1.045
Pf.12.221.0_CDS_at	161	1.291	1.904	**2.253**
Pf.12.222.0_CDS_at	439	0.738	0.999	1.869
Pf.12.223.0_CDS_at	316	0.639	1.010	1.880
Pf.12.224.0_CDS_at	15	0.834	0.759	1.158
Pf.12.225.0_CDS_at	23	**0.247**	**0.485**	1.320
Pf.12.225.0_CDS_x_at	9	0.560	0.566	1.015
Pf.12.226.0_CDS_x_at	7	1.553	1.007	0.985
Pf.12.227.0_CDS_at	465	0.665	**0.267**	0.788
Pf.12.228.0_CDS_at	11	1.399	1.151	0.898
Pf.12.231.0_CDS_at	926	1.518	1.855	1.261
Pf.12.232.0_CDS_at	410	1.539	0.693	0.827
Pf.12.234.0_CDS_at	70	0.683	0.768	1.375
Pf.12.235.0_CDS_at	51	**0.493**	0.959	0.547
Pf.12.236.0_CDS_a_at	20	1.174	**0.486**	0.591
Pf.12.237.0_CDS_at	113	0.903	1.386	1.836
Pf.12.238.0_CDS_at	568	**0.144**	**0.277**	**0.255**
Pf.12.239.0_CDS_at	115	0.794	1.835	**3.169**
Pf.12.24.0_CDS_a_at	470	1.035	**2.198**	**2.004**
Pf.12.24.0_CDS_at	2157	1.004	**2.021**	1.977
Pf.12.24.0_CDS_x_at	4519	0.900	1.699	1.472
Pf.12.240.0_CDS_at	14	1.641	1.950	1.734
Pf.12.240.0_CDS_x_at	1753	**2.018**	**2.098**	**2.107**
Pf.12.241.0_CDS_s_at	42	1.918	0.662	0.682
Pf.12.242.0_CDS_s_at	7	1.010	0.853	0.770
Pf.12.244.0_CDS_at	31	**0.302**	0.689	0.931

Pf.12.245.0_CDS_at	15	0.579	**0.451**	**0.416**
Pf.12.246.0_CDS_at	1069	**0.075**	**0.108**	**0.099**
Pf.12.248.0_CDS_at	10	0.963	0.761	0.923
Pf.12.249.0_CDS_at	673	1.926	1.319	1.326
Pf.12.25.0_CDS_at	423	0.812	1.258	1.223
Pf.12.250.0_CDS_at	210	**0.401**	**0.421**	0.984
Pf.12.251.0_CDS_at	72	0.834	1.434	**2.216**
Pf.12.252.0_CDS_at	231	**3.042**	**3.360**	1.998
Pf.12.254.0_CDS_at	866	1.758	**2.172**	**2.122**
Pf.12.255.0_CDS_at	65	**2.710**	1.904	**2.163**
Pf.12.257.0_CDS_at	12	**2.300**	1.845	**2.638**
Pf.12.258.0_CDS_at	3614	**0.280**	0.571	0.976
Pf.12.259.0_CDS_at	28	1.103	1.876	1.049
Pf.12.260.1_a_at	9	1.653	0.655	0.692
Pf.12.261.0_CDS_at	296	**0.447**	0.623	**0.187**
Pf.12.262.0_CDS_at	17	**2.093**	**2.074**	**2.036**
Pf.12.263.0_CDS_at	10	0.998	1.163	**5.106**
Pf.12.264.0_CDS_at	20	0.862	1.237	1.418
Pf.12.265.0_CDS_at	501	0.903	**0.429**	**0.488**
Pf.12.266.0_CDS_at	12	0.769	0.692	0.657
Pf.12.267.0_CDS_at	233	**0.316**	0.594	1.312
Pf.12.268.0_CDS_at	10	0.778	0.616	0.803
Pf.12.269.0_CDS_at	49	0.921	0.710	1.127
Pf.12.27.0_CDS_at	161	0.727	0.751	**0.320**
Pf.12.27.0_CDS_x_at	216	0.850	0.688	**0.349**
Pf.12.270.0_CDS_at	835	**0.207**	**0.069**	**0.038**
Pf.12.271.0_CDS_at	72	**0.404**	**0.410**	**0.137**
Pf.12.272.0_CDS_at	209	**0.205**	**0.360**	**2.076**
Pf.12.273.0_CDS_at	21	1.897	**2.431**	**2.562**
Pf.12.274.0_CDS_at	5	1.254	1.077	0.930
Pf.12.275.0_CDS_at	10	0.669	0.832	1.145
Pf.12.276.0_CDS_at	13	0.668	0.690	0.451
Pf.12.277.0_CDS_at	138	0.800	**2.119**	1.706
Pf.12.277.0_CDS_x_at	109	0.966	**2.212**	1.997
Pf.12.278.0_CDS_at	1169	1.533	1.311	1.619
Pf.12.279.0_CDS_at	76	**0.416**	**0.218**	1.363
Pf.12.28.0_CDS_at	1550	**0.384**	0.817	0.697
Pf.12.280.0_CDS_at	85	0.953	**2.030**	**2.042**
Pf.12.281.0_CDS_at	227	0.501	0.786	**0.542**
Pf.12.282.0_CDS_at	471	0.872	1.161	1.280
Pf.12.283.0_CDS_at	423	**2.641**	**3.198**	1.390
Pf.12.285.0_CDS_at	337	0.553	**0.393**	1.073
Pf.12.286.0_CDS_at	502	1.295	1.825	1.409
Pf.12.287.0_CDS_at	4880	1.862	1.820	1.435
Pf.12.29.0_CDS_at	636	1.883	1.550	1.523
Pf.12.29.0_UTR_at	4	0.925	0.921	0.935
Pf.12.29.0_UTR_x_at	5	0.999	1.196	0.962
Pf.12.290.0_CDS_x_at	5	0.914	1.002	1.135
Pf.12.291.0_CDS_at	366	**2.999**	1.091	0.871
Pf.12.292.0_CDS_at	59	**3.076**	0.909	**0.315**
Pf.12.293.0_CDS_at	321	**2.611**	**3.634**	**2.363**
Pf.12.294.0_CDS_at	19	**2.833**	1.855	**2.280**
Pf.12.296.0_CDS_at	39	0.999	1.107	0.899
Pf.12.296.0_CDS_x_at	183	1.145	1.239	0.984
Pf.12.297.0_CDS_at	9	**2.264**	0.889	0.949
Pf.12.298.0_CDS_at	762	**3.143**	1.633	1.578
Pf.12.299.0_CDS_at	118	**0.384**	0.511	0.545
Pf.12.3.0_CDS_at	20	**0.329**	**0.320**	**0.260**
Pf.12.30.0_CDS_at	6730	**0.471**	0.732	**0.463**
Pf.12.301.0_CDS_at	18	1.162	0.541	0.508
Pf.12.302.0_CDS_at	77	**0.426**	**0.204**	1.089
Pf.12.303.0_CDS_at	30	1.233	**0.263**	**0.278**

Pf.12.304.0_CDS_at	18	**3.058**	**4.196**	**5.197**
Pf.12.306.0_CDS_at	6	1.188	0.814	0.822
Pf.12.306.0_CDS_x_at	6	0.975	0.762	0.807
Pf.12.307.0_CDS_at	645	0.996	0.846	1.849
Pf.12.308.0_CDS_at	299	0.999	1.322	1.424
Pf.12.309.0_CDS_at	12	1.281	1.462	0.601
Pf.12.31.0_CDS_at	259	0.927	0.922	1.236
Pf.12.310.0_CDS_at	144	**0.259**	0.878	1.032
Pf.12.312.0_CDS_at	20	1.006	1.084	0.650
Pf.12.313.0_CDS_at	43	0.965	0.977	1.051
Pf.12.313.1_a_at	262	0.875	1.065	1.071
Pf.12.314.0_CDS_at	28	**0.297**	**0.335**	1.556
Pf.12.315.0_CDS_at	33	1.063	0.504	1.077
Pf.12.316.0_CDS_at	709	0.650	0.789	0.886
Pf.12.317.0_CDS_at	632	**0.100**	**0.144**	**0.201**
Pf.12.318.0_CDS_at	44	1.656	1.708	1.663
Pf.12.319.0_CDS_at	33	1.657	**0.372**	**0.434**
Pf.12.32.0_CDS_at	7	0.919	0.977	0.895
Pf.12.320.0_CDS_at	1301	1.009	1.579	**2.005**
Pf.12.321.0_CDS_at	139	0.712	0.564	0.557
Pf.12.323.0_CDS_at	11	0.874	0.803	0.803
Pf.12.325.0_CDS_at	6	1.570	0.915	3.025
Pf.12.326.0_CDS_at	109	**3.466**	0.628	**2.579**
Pf.12.326.0_CDS_x_at	132	**2.777**	0.518	**2.035**
Pf.12.327.0_CDS_at	6	1.544	1.043	1.255
Pf.12.328.0_CDS_at	423	**0.229**	**0.176**	**0.145**
Pf.12.329.0_CDS_at	82	1.335	0.611	**0.492**
Pf.12.33.0_CDS_at	8	1.048	0.701	0.794
Pf.12.330.0_CDS_at	57	1.350	1.125	1.170
Pf.12.331.0_CDS_at	25	1.411	**0.471**	0.800
Pf.12.332.0_CDS_at	261	0.719	**2.019**	**2.038**
Pf.12.333.0_CDS_at	70	1.771	1.842	**2.344**
Pf.12.334.0_CDS_at	29	0.694	0.854	1.394
Pf.12.335.0_CDS_at	15	0.750	0.886	0.896
Pf.12.336.0_CDS_at	69	0.730	**2.036**	**2.886**
Pf.12.337.0_CDS_at	189	0.591	0.623	1.121
Pf.12.338.0_CDS_at	72	0.788	1.534	1.137
Pf.12.339.0_CDS_at	37	0.604	1.072	1.976
Pf.12.341.0_CDS_at	66	**0.495**	0.591	1.030
Pf.12.342.0_CDS_at	1128	**0.372**	0.533	0.969
Pf.12.343.0_CDS_at	42	0.849	1.975	**2.342**
Pf.12.344.0_CDS_at	408	1.424	**2.012**	**2.524**
Pf.12.345.0_CDS_at	410	0.821	1.131	0.934
Pf.12.346.0_CDS_at	53	0.713	0.691	1.552
Pf.12.347.0_CDS_at	51	0.925	1.994	1.955
Pf.12.348.0_CDS_at	45	0.832	**0.465**	1.510
Pf.12.349.0_CDS_at	1130	**3.310**	**3.658**	**2.604**
Pf.12.35.0_CDS_at	91	**0.164**	**0.135**	**0.127**
Pf.12.350.0_CDS_at	14	1.509	0.637	**2.023**
Pf.12.351.0_CDS_at	113	**0.329**	0.931	1.781
Pf.12.352.0_CDS_at	76	**0.129**	**0.326**	**0.203**
Pf.12.354.0_CDS_at	652	0.587	0.957	1.880
Pf.12.355.0_CDS_at	259	1.672	**2.027**	1.758
Pf.12.356.0_CDS_at	137	1.552	**3.623**	**4.212**
Pf.12.357.0_CDS_at	7	1.065	0.783	0.827
Pf.12.36.0_CDS_at	4209	1.337	1.552	1.230
Pf.12.360.0_CDS_at	9	1.212	0.729	0.970
Pf.12.361.0_CDS_at	128	1.571	1.387	**2.799**
Pf.12.362.0_CDS_at	6	1.261	1.215	1.039
Pf.12.363.0_CDS_at	363	0.768	1.994	**2.123**
Pf.12.364.0_CDS_at	143	**0.446**	**0.432**	**0.358**
Pf.12.365.0_CDS_at	117	0.825	1.462	**2.254**

Pf.12.366.0_CDS_at	39	1.669	**2.863**	1.589
Pf.12.367.0_CDS_at	114	0.782	1.361	1.965
Pf.12.369.0_CDS_at	236	0.567	0.714	0.783
Pf.12.37.0_CDS_at	2293	1.057	1.080	1.067
Pf.12.371.0_CDS_at	25	**3.589**	**2.750**	**3.341**
Pf.12.371.0_CDS_x_at	24	**3.869**	**3.394**	**4.096**
Pf.12.373.0_CDS_x_at	6	1.184	0.980	1.024
Pf.12.374.0_CDS_at	8	0.906	0.722	0.683
Pf.12.375.0_CDS_at	4	0.983	1.063	0.895
Pf.12.375.0_CDS_x_at	5	1.062	0.947	0.921
Pf.12.376.0_CDS_at	89	**0.361**	0.839	0.788
Pf.12.38.0_CDS_at	643	0.778	1.099	1.102
Pf.12.380.0_CDS_at	266	**2.093**	1.484	**2.101**
Pf.12.381.0_CDS_at	496	1.264	0.849	1.237
Pf.12.382.0_CDS_at	6	1.024	1.172	1.022
Pf.12.383.0_CDS_at	103	1.115	0.944	1.561
Pf.12.384.0_CDS_at	10	0.948	0.730	0.801
Pf.12.385.0_CDS_at	16	0.632	0.832	0.717
Pf.12.386.0_CDS_at	96	0.585	0.188	0.793
Pf.12.387.0_CDS_at	236	1.404	**2.396**	**2.476**
Pf.12.388.0_CDS_at	8	0.977	1.032	0.855
Pf.12.39.0_CDS_at	179	0.517	0.530	**0.146**
Pf.12.391.0_CDS_at	530	1.318	1.673	1.866
Pf.12.392.0_CDS_a_at	379	1.534	1.433	1.527
Pf.12.394.0_CDS_at	211	1.007	0.881	1.949
Pf.12.395.0_CDS_x_at	259	**4.459**	0.566	0.575
Pf.12.396.0_CDS_at	33	**0.405**	**0.314**	**0.249**
Pf.12.397.0_CDS_at	19	0.710	0.511	**0.442**
Pf.12.398.0_CDS_at	174	1.432	1.552	0.551
Pf.12.399.0_CDS_at	750	0.563	0.666	0.614
Pf.12.4.0_CDS_at	7275	1.297	0.994	1.095
Pf.12.40.0_CDS_at	14	1.448	0.454	0.801
Pf.12.402.0_CDS_at	15	**5.349**	1.588	1.507
Pf.12.407.0_CDS_at	6	1.007	0.850	0.901
Pf.12.408.0_CDS_at	6	1.032	0.909	1.091
Pf.12.409.0_CDS_at	5	1.102	1.002	0.917
Pf.12.41.0_CDS_at	262	**0.222**	0.622	**0.319**
Pf.12.410.0_CDS_s_at	5	0.886	1.017	0.979
Pf.12.411.0_CDS_at	6	0.906	0.975	0.865
Pf.12.412.0_CDS_at	5	1.330	0.907	1.241
Pf.12.415.0_CDS_at	5	1.028	1.066	1.255
Pf.12.416.0_CDS_at	6	0.832	1.039	0.898
Pf.12.417.0_CDS_at	6	1.269	1.030	1.338
Pf.12.418.0_CDS_at	6	0.968	0.884	0.904
Pf.12.419.0_CDS_at	145	**5.139**	**2.112**	1.919
Pf.12.42.0_CDS_at	1056	1.784	1.842	1.232
Pf.12.420.0_CDS_at	147	**5.348**	**3.042**	**2.222**
Pf.12.421.0_CDS_at	56	**16.691**	**7.136**	**3.951**
Pf.12.426.0_CDS_at	149	1.241	1.746	1.760
Pf.12.428.0_CDS_at	177	1.490	**3.241**	1.928
Pf.12.429.0_CDS_at	61	1.159	1.005	0.975
Pf.12.43.0_CDS_at	235	0.931	1.529	1.910
Pf.12.430.0_CDS_at	14	1.576	1.102	0.909
Pf.12.431.0_CDS_at	84	**0.482**	1.294	**2.222**
Pf.12.432.0_CDS_at	20	0.850	0.897	0.730
Pf.12.433.0_CDS_at	1300	**2.965**	**2.494**	**2.171**
Pf.12.436.0_CDS_at	10	1.099	0.807	0.926
Pf.12.437.0_CDS_at	11	1.311	1.207	**2.228**
Pf.12.438.0_CDS_at	67	1.946	**3.423**	1.744
Pf.12.44.0_CDS_at	192	0.837	0.519	1.148
Pf.12.44.0_UTR_at	129	0.786	1.006	**2.027**
Pf.12.440.0_CDS_at	28	1.046	**2.357**	1.479

Pf.12.441.0_CDS_at	182	1.491	1.796	1.572
Pf.12.443.0_CDS_at	32	1.237	**3.719**	**2.312**
Pf.12.444.0_CDS_at	343	0.563	**0.493**	0.835
Pf.12.446.0_CDS_at	6	1.051	0.809	0.914
Pf.12.447.0_CDS_at	637	1.553	1.673	1.951
Pf.12.447.0_CDS_x_at	767	1.580	1.543	1.703
Pf.12.448.0_CDS_at	1186	1.500	1.944	1.834
Pf.12.45.1_a_at	149	1.674	**0.153**	**0.215**
Pf.12.450.0_CDS_at	9	1.034	0.719	1.800
Pf.12.451.0_CDS_at	418	**0.356**	0.744	1.201
Pf.12.452.0_CDS_at	427	1.047	1.630	1.283
Pf.12.453.0_CDS_at	2102	**0.323**	0.739	1.576
Pf.12.453.0_CDS_x_at	2733	**0.338**	0.756	1.623
Pf.12.454.0_CDS_at	169	**0.367**	**0.389**	**0.536**
Pf.12.456.0_CDS_at	210	1.021	0.523	**0.218**
Pf.12.457.0_CDS_at	1342	0.885	0.652	0.663
Pf.12.458.0_CDS_at	16	1.387	0.596	**4.938**
Pf.12.46.0_CDS_at	101	1.014	**2.075**	**2.238**
Pf.12.461.0_CDS_at	234	0.754	0.746	1.522
Pf.12.462.0_CDS_at	816	1.117	1.706	1.948
Pf.12.463.0_CDS_at	64	0.946	1.401	1.478
Pf.12.464.0_CDS_at	18	1.631	0.832	1.041
Pf.12.465.0_CDS_at	122	1.536	0.728	**0.396**
Pf.12.466.0_CDS_at	54	**2.726**	0.952	0.659
Pf.12.467.0_CDS_at	11	1.936	0.984	1.698
Pf.12.468.0_CDS_at	8	1.277	0.779	1.162
Pf.12.469.0_CDS_at	63	1.335	**2.508**	**2.464**
Pf.12.47.0_CDS_at	109	0.668	**0.220**	**0.192**
Pf.12.470.0_CDS_at	10	1.494	0.736	0.681
Pf.12.471.0_CDS_at	10	0.756	0.565	0.699
Pf.12.472.0_CDS_at	599	**0.130**	**0.220**	0.642
Pf.12.473.0_CDS_at	124	**0.246**	**0.143**	**0.134**
Pf.12.474.0_CDS_at	355	1.170	0.551	0.805
Pf.12.475.0_CDS_at	762	1.737	1.705	**2.343**
Pf.12.476.0_CDS_at	14	1.119	0.951	1.217
Pf.12.48.0_CDS_at	174	1.517	**2.131**	**2.499**
Pf.12.480.0_CDS_at	86	**2.574**	**3.011**	1.217
Pf.12.482.0_CDS_at	276	**0.271**	0.896	1.029
Pf.12.484.0_CDS_at	348	1.150	**0.310**	**0.486**
Pf.12.485.0_CDS_at	398	0.886	1.047	1.842
Pf.12.486.0_CDS_at	10	1.211	1.206	**4.600**
Pf.12.488.0_CDS_at	17	0.883	0.748	0.584
Pf.12.489.0_CDS_at	6	1.073	1.035	1.388
Pf.12.490.0_CDS_at	193	**0.306**	0.634	1.459
Pf.12.491.0_CDS_at	174	0.652	0.607	1.815
Pf.12.492.0_CDS_at	12	**0.430**	0.815	0.830
Pf.12.493.0_CDS_at	158	0.807	0.523	1.339
Pf.12.495.0_CDS_at	186	0.660	0.731	0.689
Pf.12.496.0_CDS_at	8	1.160	0.720	0.773
Pf.12.498.0_CDS_at	100	**2.221**	0.904	**0.265**
Pf.12.499.0_CDS_at	20	0.830	0.510	**0.444**
Pf.12.5.0_CDS_at	100	1.545	**0.209**	**0.336**
Pf.12.50.0_CDS_at	674	1.014	1.691	0.641
Pf.12.500.0_CDS_at	542	1.631	1.767	**2.195**
Pf.12.501.0_CDS_at	8	1.320	0.853	0.958
Pf.12.502.0_CDS_at	11	1.857	0.836	0.730
Pf.12.504.0_CDS_at	5	1.170	1.006	1.279
Pf.12.505.0_CDS_at	1034	0.541	1.228	1.375
Pf.12.506.0_CDS_at	214	1.987	**0.333**	0.517
Pf.12.508.0_CDS_at	144	**3.618**	**4.841**	**2.254**
Pf.12.509.0_CDS_at	582	**2.540**	**2.735**	1.229
Pf.12.510.0_CDS_at	180	1.308	0.588	1.199

Pf.12.511.0_CDS_at	7	1.339	1.298	0.830
Pf.12.512.0_CDS_at	7	0.983	0.847	1.926
Pf.12.513.0_CDS_at	563	**0.208**	**0.289**	0.528
Pf.12.514.0_CDS_at	657	1.123	1.534	1.389
Pf.12.516.0_CDS_at	685	0.541	0.778	1.313
Pf.12.517.0_CDS_at	110	**0.387**	0.590	0.688
Pf.12.518.0_CDS_at	21	0.806	1.996	**2.984**
Pf.12.521.0_CDS_at	292	0.549	**0.385**	0.656
Pf.12.522.0_CDS_at	883	1.013	0.743	**2.249**
Pf.12.524.0_CDS_at	34	**2.002**	0.696	1.010
Pf.12.526.0_CDS_at	20	**2.211**	0.530	**0.473**
Pf.12.527.0_CDS_at	10	1.434	0.848	1.020
Pf.12.53.0_CDS_at	29	**0.361**	0.745	0.799
Pf.12.530.0_CDS_at	17	1.684	1.350	0.809
Pf.12.531.0_CDS_at	6	0.961	0.747	0.865
Pf.12.532.0_CDS_at	6	1.004	1.068	0.987
Pf.12.54.0_CDS_at	115	1.380	1.702	1.520
Pf.12.55.0_CDS_at	1560	0.508	**0.478**	1.235
Pf.12.553.0_at	388	**0.378**	**0.240**	**0.149**
Pf.12.557.0_at	26	1.401	0.611	0.657
Pf.12.57.0_CDS_at	1585	1.866	**2.343**	1.590
Pf.12.570.0_at	239	**0.397**	**0.435**	**0.444**
Pf.12.58.0_CDS_at	69	0.504	**0.462**	**0.471**
Pf.12.588.0_at	37	**0.289**	**0.302**	1.656
Pf.12.59.0_CDS_at	372	**0.098**	**0.126**	**0.091**
Pf.12.594.0_at	49	1.033	**3.811**	1.153
Pf.12.596.0_at	151	1.120	1.493	0.776
Pf.12.6.0_CDS_at	190	0.708	1.481	1.425
Pf.12.60.0_CDS_at	107	1.440	1.433	1.736
Pf.12.610.0_at	448	**0.405**	0.697	**0.169**
Pf.12.62.0_CDS_at	241	0.900	1.327	1.830
Pf.12.626.0_at	3109	1.183	0.609	0.505
Pf.12.63.0_CDS_at	367	1.653	1.742	1.269
Pf.12.64.0_CDS_at	38	0.740	0.558	**0.437**
Pf.12.65.0_CDS_at	10	1.213	1.184	1.149
Pf.12.67.0_CDS_at	170	**0.468**	**0.247**	**0.365**
Pf.12.673.0_at	126	0.996	0.726	0.560
Pf.12.68.0_CDS_a_at	948	1.668	1.422	1.728
Pf.12.69.2_a_at	750	1.727	**0.414**	**0.448**
Pf.12.7.0_CDS_a_at	673	1.588	1.763	0.792
Pf.12.70.0_CDS_at	103	0.860	0.869	1.167
Pf.12.71.0_CDS_at	21	0.612	0.551	0.510
Pf.12.72.0_CDS_at	1566	1.424	1.036	1.680
Pf.12.73.0_CDS_at	275	**0.343**	0.634	0.674
Pf.12.75.0_CDS_at	16	1.728	1.679	**2.279**
Pf.12.76.0_CDS_at	26	1.534	1.463	**2.660**
Pf.12.78.0_CDS_at	1690	1.068	1.342	1.462
Pf.12.79.0_CDS_at	351	1.213	1.697	1.325
Pf.12.8.0_CDS_at	47	**2.291**	**2.073**	**0.457**
Pf.12.80.0_CDS_at	3098	**0.314**	0.514	0.918
Pf.12.81.0_CDS_at	79	**0.181**	**0.118**	**0.124**
Pf.12.82.0_CDS_at	543	**2.110**	0.690	**0.254**
Pf.12.82.0_UTR_at	40	1.995	0.537	0.697
Pf.12.82.0_UTR_x_at	23	1.433	0.648	0.784
Pf.12.83.0_CDS_at	235	1.089	0.980	1.858
Pf.12.84.0_CDS_at	818	**2.969**	1.757	1.942
Pf.12.86.0_CDS_at	184	**0.254**	0.537	1.334
Pf.12.87.0_CDS_at	110	**9.171**	**8.871**	1.928
Pf.12.88.0_CDS_at	286	1.223	1.016	1.507
Pf.12.89.0_CDS_at	7	1.204	1.010	1.207
Pf.12.9.0_CDS_a_at	5523	0.678	0.558	1.070
Pf.12.90.0_CDS_at	360	1.895	1.228	**0.381**

Pf.12.91.0_CDS_at	99	**0.406**	0.688	1.418
Pf.12.92.0_CDS_at	165	1.674	0.859	**0.390**
Pf.12.93.0_CDS_at	17	1.122	0.950	1.890
Pf.12.94.0_CDS_at	806	1.191	1.258	**0.387**
Pf.12.95.0_CDS_at	156	1.582	**2.068**	1.708
Pf.12.96.0_CDS_at	29	0.833	0.859	**2.452**
Pf.12.97.0_CDS_at	132	0.570	1.339	1.086
Pf.12.98.0_CDS_at	1495	0.532	**0.492**	0.868
Pf.12.99.0_CDS_at	139	**0.316**	**0.312**	**0.405**
Pf.13_1.1.0_UTR_a_at	13981	0.756	1.101	0.870
Pf.13_1.1.0_UTR_x_at	2503	0.846	1.276	0.991
Pf.13_1.1.1_UTR_a_at	14333	0.746	1.152	0.880
Pf.13_1.1.6_CDS_a_at	3593	0.760	1.321	0.895
Pf.13_1.10.0_CDS_at	1282	0.505	0.669	0.743
Pf.13_1.101.0_CDS_at	276	1.192	1.304	0.631
Pf.13_1.102.0_CDS_a_at	1592	1.837	1.672	**2.055**
Pf.13_1.103.0_CDS_at	1607	**0.284**	0.758	**0.363**
Pf.13_1.104.0_CDS_at	642	0.937	1.227	1.546
Pf.13_1.105.1_s_at	13	1.023	0.691	0.951
Pf.13_1.106.0_CDS_at	170	**2.107**	**2.451**	**2.152**
Pf.13_1.107.0_CDS_at	970	**0.192**	**0.448**	0.615
Pf.13_1.109.0_CDS_at	41	0.865	0.962	1.449
Pf.13_1.11.0_CDS_at	676	0.941	1.355	0.631
Pf.13_1.110.0_CDS_at	676	0.927	0.776	0.554
Pf.13_1.111.0_CDS_at	160	1.396	1.184	**2.914**
Pf.13_1.112.1_a_at	31	0.746	1.100	0.984
Pf.13_1.113.0_CDS_at	1981	1.416	1.452	1.474
Pf.13_1.114.0_CDS_at	411	0.642	**0.464**	1.101
Pf.13_1.115.0_CDS_at	513	**0.434**	0.586	0.538
Pf.13_1.116.0_CDS_at	7	0.917	0.853	0.840
Pf.13_1.116.0_CDS_x_at	7	0.821	1.125	0.816
Pf.13_1.117.0_CDS_at	25	0.516	**0.257**	**0.260**
Pf.13_1.118.0_CDS_a_at	4022	1.460	1.143	1.373
Pf.13_1.119.0_CDS_at	1740	0.977	1.247	1.544
Pf.13_1.120.0_CDS_at	1814	1.226	0.987	1.545
Pf.13_1.122.0_CDS_at	1310	1.739	1.420	1.610
Pf.13_1.123.0_CDS_at	694	0.808	**0.488**	1.120
Pf.13_1.124.0_CDS_at	276	**0.222**	**0.349**	0.672
Pf.13_1.125.0_CDS_at	250	0.590	0.626	1.056
Pf.13_1.126.0_CDS_at	178	**0.212**	**0.177**	1.312
Pf.13_1.129.0_CDS_at	1529	1.201	1.598	1.402
Pf.13_1.13.0_CDS_at	652	0.576	0.817	0.563
Pf.13_1.130.0_CDS_at	179	**0.243**	**0.290**	**0.114**
Pf.13_1.131.0_CDS_at	23	1.318	1.447	0.948
Pf.13_1.133.0_CDS_at	624	1.627	1.881	1.015
Pf.13_1.134.0_CDS_at	3963	**0.374**	0.613	0.866
Pf.13_1.135.0_CDS_at	16	0.971	0.768	0.681
Pf.13_1.137.0_CDS_at	1647	1.524	1.720	1.260
Pf.13_1.139.0_CDS_at	714	1.440	1.509	1.863
Pf.13_1.14.0_CDS_at	16	1.373	0.882	0.803
Pf.13_1.141.0_CDS_at	1176	**0.344**	**0.474**	**0.211**
Pf.13_1.142.0_CDS_at	167	0.904	0.929	1.089
Pf.13_1.143.0_CDS_a_at	152	**0.405**	**0.653**	**0.442**
Pf.13_1.144.0_CDS_at	518	**0.453**	**0.310**	**0.290**
Pf.13_1.146.0_CDS_at	3113	**0.329**	0.716	0.621
Pf.13_1.147.0_CDS_at	8	1.023	0.899	0.991
Pf.13_1.148.0_CDS_at	446	0.579	**0.385**	0.850
Pf.13_1.149.0_CDS_at	50	**2.080**	0.540	**0.416**
Pf.13_1.15.0_CDS_at	1477	**0.162**	0.573	**0.259**
Pf.13_1.150.0_CDS_at	443	1.350	1.279	1.367
Pf.13_1.151.0_CDS_at	427	1.819	**2.134**	1.329
Pf.13_1.152.0_CDS_at	73	0.740	1.321	1.567

Pf.13_1.153.0_CDS_at	57	**2.243**	**2.602**	**2.536**
Pf.13_1.154.0_CDS_at	23	0.526	1.373	**0.316**
Pf.13_1.155.0_CDS_at	3142	0.881	0.660	1.058
Pf.13_1.156.0_CDS_at	2171	**0.128**	**0.319**	**0.240**
Pf.13_1.157.0_CDS_at	199	0.697	0.684	1.464
Pf.13_1.158.0_CDS_at	32	0.678	0.985	**0.456**
Pf.13_1.159.0_CDS_at	850	1.807	**2.434**	**2.215**
Pf.13_1.16.0_CDS_at	10	1.198	1.040	1.945
Pf.13_1.16.0_CDS_x_at	166	1.056	1.062	1.404
Pf.13_1.160.0_CDS_at	238	1.292	1.865	1.751
Pf.13_1.161.0_CDS_at	7	2.003	1.470	1.536
Pf.13_1.162.0_CDS_at	174	**2.220**	**2.602**	**2.282**
Pf.13_1.163.0_CDS_at	12	0.817	0.686	0.984
Pf.13_1.164.0_CDS_at	25	0.714	1.366	1.405
Pf.13_1.165.0_CDS_at	2529	**0.461**	**0.496**	1.139
Pf.13_1.167.0_CDS_at	200	1.643	0.773	**0.300**
Pf.13_1.168.0_CDS_at	139	0.565	0.767	1.053
Pf.13_1.17.0_CDS_at	12	1.135	0.938	1.139
Pf.13_1.170.0_CDS_at	77	**0.483**	0.724	1.378
Pf.13_1.172.0_CDS_at	83	0.627	0.518	0.530
Pf.13_1.173.0_CDS_at	472	1.082	1.234	**3.466**
Pf.13_1.174.0_CDS_at	135	1.346	1.881	**2.082**
Pf.13_1.177.0_CDS_at	380	0.567	**0.498**	0.536
Pf.13_1.178.0_CDS_at	125	0.986	1.058	**2.422**
Pf.13_1.179.0_CDS_at	8	1.416	0.885	0.977
Pf.13_1.18.0_CDS_a_at	23	**0.273**	**0.399**	**0.309**
Pf.13_1.180.0_CDS_at	337	**0.345**	0.691	1.019
Pf.13_1.181.0_CDS_at	894	0.950	0.753	1.247
Pf.13_1.182.0_CDS_at	1815	0.557	1.043	1.428
Pf.13_1.183.0_CDS_at	8	**2.795**	**4.560**	**3.250**
Pf.13_1.184.0_CDS_at	3644	1.523	1.440	1.375
Pf.13_1.185.0_CDS_at	432	**0.359**	**0.496**	**0.331**
Pf.13_1.186.0_CDS_at	757	1.192	1.428	1.558
Pf.13_1.187.0_CDS_at	53	0.799	1.062	**0.380**
Pf.13_1.188.0_CDS_at	10	0.633	1.135	1.814
Pf.13_1.189.0_CDS_at	1260	1.539	0.768	0.628
Pf.13_1.19.0_CDS_at	802	**0.158**	**0.343**	**0.347**
Pf.13_1.190.0_CDS_at	432	**0.036**	**0.105**	**0.131**
Pf.13_1.192.0_CDS_a_at	2988	1.234	1.282	1.607
Pf.13_1.193.0_CDS_at	30	0.697	0.907	0.751
Pf.13_1.194.0_CDS_at	406	0.854	1.561	1.502
Pf.13_1.195.0_CDS_at	176	0.890	0.952	1.467
Pf.13_1.196.0_CDS_at	1312	0.715	0.832	1.094
Pf.13_1.197.0_CDS_at	31	1.000	1.356	**2.887**
Pf.13_1.198.0_CDS_at	49	0.761	1.307	**2.682**
Pf.13_1.199.0_CDS_at	102	**2.239**	1.839	1.728
Pf.13_1.2.0_CDS_at	3963	**0.391**	**0.473**	**0.403**
Pf.13_1.20.0_CDS_at	333	1.692	1.542	1.691
Pf.13_1.20.0_CDS_x_at	97	1.683	1.543	1.795
Pf.13_1.200.0_CDS_at	48	1.982	1.417	**2.848**
Pf.13_1.201.0_CDS_at	606	0.846	0.918	1.390
Pf.13_1.202.0_CDS_at	356	0.810	1.319	**2.186**
Pf.13_1.203.0_CDS_at	50	1.124	**0.292**	**0.250**
Pf.13_1.204.0_CDS_at	10	0.697	1.339	1.088
Pf.13_1.206.0_CDS_at	24	**0.190**	0.593	0.913
Pf.13_1.207.0_CDS_at	360	1.322	**2.265**	**2.208**
Pf.13_1.208.0_CDS_at	1250	1.487	0.955	1.590
Pf.13_1.209.0_CDS_at	19	1.185	1.399	**2.936**
Pf.13_1.21.0_CDS_at	4700	1.314	1.185	1.187
Pf.13_1.211.0_CDS_at	49	1.903	0.791	**0.484**
Pf.13_1.213.0_CDS_a_at	496	1.737	1.572	1.723
Pf.13_1.214.0_CDS_at	31	0.636	**0.417**	0.645

Pf.13_1.215.0_CDS_at	809	**3.468**	**3.227**	1.955	
Pf.13_1.217.0_CDS_at	190	0.645	0.577	0.720	
Pf.13_1.22.0_CDS_at	10523	**0.391**	**0.476**	0.576	
Pf.13_1.220.0_CDS_at	1449	**0.447**	0.539	0.867	
Pf.13_1.222.0_CDS_at	366	**0.285**	**0.203**	0.530	
Pf.13_1.223.0_CDS_at	1336	1.307	1.277	1.566	
Pf.13_1.224.0_CDS_at	23	0.682	0.750	0.850	
Pf.13_1.225.0_CDS_at	1036	1.451	1.403	1.773	
Pf.13_1.226.0_CDS_at	45	1.203	**2.041**	1.755	
Pf.13_1.227.0_CDS_at	155	**0.498**	0.997	0.864	
Pf.13_1.228.0_CDS_at	201	0.694	0.921	0.566	
Pf.13_1.23.0_CDS_at	1599	1.113	1.248	1.200	
Pf.13_1.230.0_CDS_at	121	1.650	**2.779**	1.231	
Pf.13_1.231.0_CDS_at	306	0.949	1.226	1.059	
Pf.13_1.232.0_CDS_at	377	0.761	**0.380**	1.041	
Pf.13_1.233.0_CDS_at	288	1.731	1.135	1.649	
Pf.13_1.234.0_CDS_at	1893	0.507	0.924	**2.115**	
Pf.13_1.235.0_CDS_at	816	**0.148**	0.508	1.044	
Pf.13_1.236.0_CDS_at	1873	0.500	1.158	1.447	
Pf.13_1.237.0_CDS_at	141	1.232	0.760	0.678	
Pf.13_1.238.0_CDS_at	173	**2.588**	**2.486**	**2.529**	
Pf.13_1.238.0_CDS_x_at	44	**2.427**	1.910	**2.784**	
Pf.13_1.242.0_CDS_at	379	0.791	1.376	1.470	
Pf.13_1.244.0_CDS_at	284	**0.372**	0.564	**0.436**	
Pf.13_1.245.0_CDS_at	99	0.901	1.136	**2.475**	
Pf.13_1.245.0_CDS_x_at	265	0.755	0.976	1.603	
Pf.13_1.246.0_CDS_at	81	**0.147**	**0.332**	**0.405**	
Pf.13_1.248.0_CDS_at	179	**2.813**	1.933	**2.711**	
Pf.13_1.249.0_CDS_at	206	1.281	1.445	1.691	
Pf.13_1.250.0_CDS_at	1098	0.799	1.190	1.811	
Pf.13_1.252.0_CDS_at	869	1.333	1.243	1.702	
Pf.13_1.253.0_CDS_at	381	0.952	0.809	1.040	
Pf.13_1.254.0_CDS_at	16	0.725	0.996	**2.195**	
Pf.13_1.256.0_CDS_at	861	**2.034**	1.885	1.541	
Pf.13_1.258.0_CDS_at	239	1.226	0.907	1.440	
Pf.13_1.259.0_CDS_at	102	**7.953**	1.899	**2.157**	
Pf.13_1.26.0_CDS_at	726	1.103	1.336	1.665	
Pf.13_1.260.0_CDS_at	531	**2.201**	1.494	1.321	
Pf.13_1.261.0_CDS_at	220	1.662	0.996	0.941	
Pf.13_1.262.0_CDS_at	479	0.700	1.054	1.294	
Pf.13_1.262.0_UTR_at	7	0.809	0.921	1.109	
Pf.13_1.262.0_UTR_x_at	7	0.956	0.983	1.314	
Pf.13_1.263.0_CDS_at	916	1.043	1.393	1.574	
Pf.13_1.264.0_CDS_at	167	0.860	1.014	1.779	
Pf.13_1.265.0_CDS_at	574	**0.269**	0.553	**0.352**	
Pf.13_1.266.0_CDS_at	490	0.874	1.606	1.426	
Pf.13_1.267.0_CDS_at	116	0.568	1.101	0.850	
Pf.13_1.268.0_CDS_a_at	261	**0.108**	**0.154**	**0.119**	
Pf.13_1.269.0_CDS_at	5	1.026	0.977	0.992	
Pf.13_1.269.0_CDS_x_at	5	0.980	0.929	0.882	
Pf.13_1.270.0_CDS_at	7	0.779	0.728	0.619	
Pf.13_1.271.0_CDS_a_at	161	1.033	1.123	0.750	
Pf.13_1.272.0_CDS_at	8	0.938	0.767	0.902	
Pf.13_1.273.0_CDS_at	107	1.569	1.535	1.596	
Pf.13_1.274.0_CDS_at	404	**5.069**	**2.691**	1.786	
Pf.13_1.276.0_CDS_at	5	0.998	0.903	1.188	
Pf.13_1.276.0_CDS_x_at	8	0.759	0.878	1.209	
Pf.13_1.277.0_CDS_at	563	**0.325**	0.929	1.123	
Pf.13_1.278.0_CDS_at	28	**10.344**	**4.281**	1.547	#2
Pf.13_1.279.0_CDS_at	494	**0.338**	**0.343**	1.343	
Pf.13_1.280.0_CDS_at	525	0.730	1.269	**2.016**	
Pf.13_1.281.0_CDS_at	172	**0.480**	**0.384**	0.552	

Pf.13_1.282.0_CDS_at	908	**0.496**	1.079	1.312	
Pf.13_1.283.0_CDS_at	105	0.690	**0.267**	**0.412**	
Pf.13_1.284.0_CDS_at	602	**0.297**	0.832	1.081	
Pf.13_1.285.0_CDS_at	262	0.632	1.278	1.529	
Pf.13_1.286.0_CDS_at	39	1.259	1.368	1.669	
Pf.13_1.287.0_CDS_at	10	1.204	0.773	0.947	
Pf.13_1.288.0_CDS_at	14	0.827	0.812	0.926	
Pf.13_1.289.0_CDS_at	683	0.964	1.121	1.451	
Pf.13_1.29.0_CDS_a_at	1971	0.591	0.915	1.066	
Pf.13_1.29.0_UTR_at	6	1.059	1.136	1.167	
Pf.13_1.291.0_CDS_at	35	1.123	1.623	1.231	
Pf.13_1.293.0_CDS_at	336	0.546	1.309	1.277	
Pf.13_1.294.0_CDS_at	197	**2.284**	1.575	**3.922**	
Pf.13_1.295.0_CDS_at	18	**0.463**	0.574	**0.465**	
Pf.13_1.296.0_CDS_at	163	0.740	**2.650**	**0.157**	
Pf.13_1.297.0_CDS_at	67	**2.899**	**2.844**	0.997	
Pf.13_1.299.0_CDS_at	91	0.951	0.970	1.134	
Pf.13_1.3.0_CDS_at	10501	0.597	**0.448**	0.651	
Pf.13_1.30.0_CDS_at	14	0.501	0.643	0.663	
Pf.13_1.301.0_CDS_at	466	1.611	1.851	1.871	
Pf.13_1.304.0_CDS_at	356	**2.949**	**3.081**	1.486	
Pf.13_1.307.0_CDS_at	34	**0.336**	**0.257**	**0.212**	
Pf.13_1.307.1_a_at	342	0.809	**0.200**	**0.187**	
Pf.13_1.308.0_CDS_at	82	1.159	1.304	0.827	
Pf.13_1.31.0_CDS_at	31	0.667	0.667	0.772	
Pf.13_1.310.0_CDS_at	222	0.824	0.951	1.474	
Pf.13_1.311.0_CDS_at	298	1.049	1.209	**2.662**	
Pf.13_1.313.0_CDS_at	12	1.290	1.031	1.572	
Pf.13_1.315.0_CDS_at	81	**0.224**	**0.361**	0.823	
Pf.13_1.315.0_CDS_x_at	272	**0.169**	**0.356**	**0.546**	
Pf.13_1.316.0_CDS_at	2474	**0.063**	**0.137**	**0.313**	#3
Pf.13_1.317.0_CDS_at	7	0.855	0.939	0.776	
Pf.13_1.318.0_CDS_at	54	1.220	1.023	1.174	
Pf.13_1.319.0_CDS_a_at	314	0.827	0.903	1.651	
Pf.13_1.32.0_CDS_at	440	1.213	1.476	1.502	
Pf.13_1.320.0_CDS_at	239	**3.550**	**3.061**	**2.620**	
Pf.13_1.321.0_CDS_at	77	**0.462**	0.570	**0.391**	
Pf.13_1.322.0_CDS_at	56	0.550	0.634	0.707	
Pf.13_1.323.0_CDS_at	615	0.689	**2.076**	1.605	
Pf.13_1.324.0_CDS_at	119	0.914	**0.472**	0.560	
Pf.13_1.326.0_CDS_at	333	**0.229**	0.535	0.791	
Pf.13_1.328.0_CDS_at	1434	**2.095**	1.442	1.241	
Pf.13_1.329.0_CDS_at	674	0.854	1.361	1.581	
Pf.13_1.33.0_CDS_at	268	**0.436**	0.541	0.724	
Pf.13_1.33.0_UTR_at	80	1.280	0.751	1.008	
Pf.13_1.330.0_CDS_at	592	1.052	1.851	1.108	
Pf.13_1.331.0_CDS_at	456	**2.437**	1.815	1.991	
Pf.13_1.333.0_CDS_at	182	1.306	0.903	0.829	
Pf.13_1.336.0_CDS_at	9	1.025	0.649	1.530	
Pf.13_1.337.0_CDS_at	78	0.936	0.739	0.963	
Pf.13_1.339.0_CDS_at	97	**3.070**	**3.845**	**2.921**	
Pf.13_1.34.0_CDS_at	7	1.024	0.796	0.969	
Pf.13_1.341.0_CDS_at	811	1.729	1.715	1.850	
Pf.13_1.342.0_CDS_at	126	**2.315**	1.523	1.671	
Pf.13_1.344.0_CDS_at	41	1.539	0.627	0.845	
Pf.13_1.345.0_CDS_at	143	**0.295**	1.072	1.816	
Pf.13_1.346.0_CDS_at	54	0.845	0.549	0.633	
Pf.13_1.347.0_CDS_at	29	**2.318**	**2.113**	**2.273**	
Pf.13_1.347.0_CDS_x_at	79	1.565	**2.138**	1.625	
Pf.13_1.349.0_CDS_at	295	0.965	0.684	**0.359**	
Pf.13_1.35.0_CDS_at	25	1.770	**0.341**	**0.456**	
Pf.13_1.350.0_CDS_at	2368	**2.111**	1.179	0.893	
Pf.13_1.351.0_CDS_at	112	0.934	0.773	0.630	
Pf.13_1.352.0_CDS_at	308	0.876	0.841	1.133	

Pf.13_1.356.0_CDS_at	6062	**0.434**	0.528	0.837
Pf.13_1.357.0_CDS_at	22	0.703	0.823	0.618
Pf.13_1.358.0_CDS_at	235	**0.437**	**0.434**	0.551
Pf.13_1.36.0_CDS_at	174	0.689	0.764	0.846
Pf.13_1.362.0_CDS_at	316	**2.661**	0.643	**6.359**
Pf.13_1.363.0_CDS_at	13	0.873	1.115	1.153
Pf.13_1.364.0_CDS_at	59	**2.994**	1.227	1.855
Pf.13_1.365.0_CDS_at	249	0.542	**0.260**	**0.277**
Pf.13_1.366.0_CDS_at	360	1.844	**0.330**	**0.234**
Pf.13_1.367.0_CDS_at	265	1.017	1.820	**2.770**
Pf.13_1.369.0_CDS_at	41	**0.155**	**0.226**	**0.491**
Pf.13_1.37.0_CDS_at	499	1.520	**2.120**	**2.578**
Pf.13_1.370.0_CDS_at	12	0.962	0.740	1.677
Pf.13_1.371.0_CDS_at	125	0.596	0.782	**2.574**
Pf.13_1.372.0_CDS_at	55	1.503	1.587	1.074
Pf.13_1.374.0_CDS_at	181	1.892	1.309	0.961
Pf.13_1.375.0_CDS_at	14	1.461	1.120	1.329
Pf.13_1.376.0_CDS_at	30	**2.876**	**2.165**	**3.965**
Pf.13_1.376.0_CDS_x_at	41	**2.653**	**2.380**	**3.439**
Pf.13_1.377.0_CDS_at	155	0.546	**0.313**	1.721
Pf.13_1.378.0_CDS_a_at	170	0.593	0.843	1.001
Pf.13_1.379.0_CDS_at	8	1.057	0.755	0.986
Pf.13_1.38.0_CDS_at	108	0.757	1.813	0.609
Pf.13_1.381.0_CDS_at	7	1.116	0.861	1.353
Pf.13_1.381.0_CDS_x_at	6	0.963	0.917	1.619
Pf.13_1.383.0_CDS_at	2334	**0.485**	0.524	0.657
Pf.13_1.385.0_CDS_at	8	1.651	1.896	2.416
Pf.13_1.387.0_CDS_at	34	**3.542**	**3.605**	1.885
Pf.13_1.388.0_CDS_at	298	**2.248**	**2.565**	**2.179**
Pf.13_1.389.0_CDS_at	626	1.212	**2.017**	1.736
Pf.13_1.39.0_CDS_at	6798	0.971	0.598	1.135
Pf.13_1.390.0_CDS_at	1126	0.829	1.182	1.341
Pf.13_1.391.0_CDS_at	333	1.147	1.270	**2.212**
Pf.13_1.393.0_CDS_at	1651	**0.251**	**0.458**	1.197
Pf.13_1.394.0_CDS_at	529	0.934	1.344	**2.421**
Pf.13_1.395.0_CDS_at	68	**0.451**	0.855	1.468
Pf.13_1.398.0_CDS_at	8	0.835	0.763	0.832
Pf.13_1.399.0_CDS_at	6	0.982	0.940	0.924
Pf.13_1.4.0_CDS_at	1655	**0.430**	0.508	0.925
Pf.13_1.40.0_CDS_at	345	**0.414**	0.595	0.821
Pf.13_1.400.0_CDS_at	118	**0.497**	0.900	**3.605**
Pf.13_1.402.0_CDS_at	17	1.752	0.555	0.618
Pf.13_1.403.0_CDS_at	24	0.842	0.679	1.817
Pf.13_1.404.0_CDS_at	32	**0.391**	1.077	0.731
Pf.13_1.406.0_CDS_at	113	0.783	1.182	0.822
Pf.13_1.408.0_CDS_at	11	1.188	0.964	1.003
Pf.13_1.409.0_CDS_at	6	0.833	1.224	0.997
Pf.13_1.41.0_CDS_at	421	1.735	1.908	1.683
Pf.13_1.410.0_CDS_at	112	0.586	1.254	**2.471**
Pf.13_1.412.0_CDS_at	880	0.894	1.068	1.790
Pf.13_1.413.0_CDS_at	53	1.415	**0.476**	0.607
Pf.13_1.414.0_CDS_at	31	0.628	1.207	**2.794**
Pf.13_1.415.0_CDS_at	7	1.128	0.850	0.920
Pf.13_1.416.0_CDS_at	22	0.876	1.140	0.927
Pf.13_1.417.0_CDS_at	6	0.976	0.951	1.916
Pf.13_1.418.0_CDS_a_at	213	0.715	**0.163**	**0.113**
Pf.13_1.42.0_CDS_at	2092	1.053	1.234	1.120
Pf.13_1.421.0_CDS_at	19	0.571	0.565	0.743
Pf.13_1.422.0_CDS_at	7	0.644	0.982	1.010
Pf.13_1.423.0_CDS_at	26	0.777	0.498	0.751
Pf.13_1.427.0_CDS_at	122	0.797	**0.450**	1.191
Pf.13_1.429.0_CDS_at	16	0.575	0.619	1.712

Pf.13_1.43.0_CDS_at	2615	1.710	1.895	1.703
Pf.13_1.430.0_CDS_at	220	1.063	1.304	**2.561**
Pf.13_1.431.0_CDS_at	7	0.849	0.955	1.153
Pf.13_1.432.0_CDS_at	1534	1.306	1.270	1.722
Pf.13_1.433.0_CDS_at	43	1.010	0.952	0.716
Pf.13_1.434.0_CDS_at	7	1.427	0.836	1.344
Pf.13_1.435.0_CDS_at	209	1.322	0.953	1.532
Pf.13_1.436.0_CDS_a_at	8464	1.271	1.090	1.168
Pf.13_1.437.0_CDS_at	12	0.970	0.706	0.867
Pf.13_1.439.0_CDS_at	8	1.103	0.845	1.019
Pf.13_1.44.0_CDS_at	14	0.552	0.591	**0.354**
Pf.13_1.442.0_CDS_at	13	0.971	0.441	0.646
Pf.13_1.442.0_CDS_x_at	13	0.921	0.517	0.545
Pf.13_1.443.0_CDS_at	276	**0.425**	0.650	0.645
Pf.13_1.444.0_CDS_at	226	**2.713**	**2.661**	1.622
Pf.13_1.445.0_CDS_at	111	1.186	0.584	1.175
Pf.13_1.446.0_CDS_at	106	1.147	**0.318**	**0.457**
Pf.13_1.447.0_CDS_at	375	**2.519**	**2.381**	**2.233**
Pf.13_1.449.0_CDS_at	143	**3.126**	0.675	0.721
Pf.13_1.45.0_CDS_at	5609	1.210	0.970	1.144
Pf.13_1.45.1_a_at	2258	1.380	1.037	1.264
Pf.13_1.450.0_CDS_at	146	1.972	1.673	1.608
Pf.13_1.451.0_CDS_at	10	0.549	0.461	0.840
Pf.13_1.452.0_CDS_at	25	1.317	1.495	0.680
Pf.13_1.453.0_CDS_a_at	16	1.324	0.438	1.138
Pf.13_1.454.0_CDS_at	478	**2.664**	1.752	**2.281**
Pf.13_1.455.0_CDS_at	67	1.110	1.582	1.478
Pf.13_1.456.0_CDS_at	106	**3.335**	**3.522**	**3.432**
Pf.13_1.457.0_CDS_at	166	0.922	0.851	**2.301**
Pf.13_1.458.0_CDS_at	239	0.781	1.212	1.139
Pf.13_1.459.0_CDS_at	18	**0.454**	0.872	1.305
Pf.13_1.459.0_CDS_x_at	13	0.870	0.938	1.342
Pf.13_1.46.0_CDS_at	24	1.467	0.642	1.064
Pf.13_1.461.0_CDS_at	3496	0.692	0.943	**2.083**
Pf.13_1.462.0_CDS_at	33	1.871	**0.482**	**2.014**
Pf.13_1.463.0_CDS_at	167	0.635	0.903	**3.325**
Pf.13_1.464.0_CDS_at	433	0.620	1.615	**2.197**
Pf.13_1.465.0_CDS_at	142	0.741	0.784	1.234
Pf.13_1.466.0_CDS_at	49	0.984	0.539	1.475
Pf.13_1.467.0_CDS_at	10	1.110	0.680	0.980
Pf.13_1.470.0_CDS_at	183	1.032	0.959	1.338
Pf.13_1.472.0_CDS_at	10	0.921	0.785	2.062
Pf.13_1.474.0_CDS_at	236	1.122	**0.370**	1.000
Pf.13_1.476.0_CDS_at	431	**5.039**	1.152	0.672
Pf.13_1.476.0_CDS_x_at	88	**3.730**	0.913	0.582
Pf.13_1.478.0_CDS_at	15	**0.443**	0.769	0.761
Pf.13_1.479.0_CDS_at	116	1.202	0.691	**0.270**
Pf.13_1.48.0_CDS_at	361	0.707	0.758	1.115
Pf.13_1.48.1_CDS_at	214	0.583	**0.499**	0.643
Pf.13_1.480.0_CDS_a_at	201	1.972	0.900	1.334
Pf.13_1.481.0_CDS_at	15	0.950	**0.484**	0.574
Pf.13_1.482.0_CDS_at	16	1.050	0.675	0.864
Pf.13_1.483.0_CDS_at	10	0.931	0.602	1.033
Pf.13_1.484.0_CDS_at	8	0.851	0.769	0.813
Pf.13_1.485.0_CDS_at	8	0.906	0.941	0.833
Pf.13_1.486.0_CDS_at	366	**0.061**	**0.089**	**0.402**
Pf.13_1.487.0_CDS_at	122	1.117	1.347	**2.332**
Pf.13_1.489.0_CDS_at	399	0.911	0.702	0.694
Pf.13_1.49.0_CDS_at	1550	**0.288**	0.509	0.629
Pf.13_1.491.0_CDS_at	8	1.029	0.968	1.518
Pf.13_1.492.0_CDS_at	118	**0.234**	0.763	**0.386**
Pf.13_1.495.0_CDS_at	39	1.548	1.445	1.776

Pf.13_1.496.0_CDS_at	153	1.187	1.020	1.836
Pf.13_1.497.0_CDS_at	238	0.503	**0.397**	**0.491**
Pf.13_1.498.0_CDS_at	44	0.842	1.146	1.676
Pf.13_1.499.0_CDS_at	13	1.612	0.774	0.833
Pf.13_1.5.0_CDS_at	23	0.964	1.318	0.691
Pf.13_1.50.0_CDS_at	2402	0.809	0.917	1.117
Pf.13_1.501.0_CDS_at	469	**2.183**	1.881	**2.574**
Pf.13_1.503.0_CDS_at	109	1.408	1.258	**2.622**
Pf.13_1.504.0_CDS_at	671	1.494	1.709	1.797
Pf.13_1.506.0_CDS_at	242	**0.393**	0.816	1.477
Pf.13_1.507.0_CDS_at	20	0.846	0.773	**2.523**
Pf.13_1.509.0_CDS_at	77	**0.309**	0.549	0.805
Pf.13_1.510.0_CDS_at	86	1.606	**2.838**	**2.020**
Pf.13_1.511.0_CDS_at	767	0.585	0.503	0.992
Pf.13_1.512.0_CDS_at	13	0.842	0.968	0.798
Pf.13_1.513.0_CDS_at	30	1.593	0.511	0.722
Pf.13_1.514.0_CDS_at	209	0.902	0.892	1.878
Pf.13_1.516.0_CDS_at	15	1.928	0.841	0.783
Pf.13_1.516.0_CDS_x_at	10	**2.141**	0.968	0.745
Pf.13_1.517.0_CDS_at	35	0.811	0.556	0.922
Pf.13_1.518.0_CDS_at	88	**2.240**	**3.314**	**3.149**
Pf.13_1.519.0_CDS_a_at	6	0.978	0.934	0.879
Pf.13_1.52.0_CDS_at	9	1.097	0.946	1.444
Pf.13_1.520.0_CDS_at	343	1.391	0.974	1.682
Pf.13_1.521.0_CDS_at	17	1.510	**0.415**	0.586
Pf.13_1.522.0_CDS_at	164	1.057	1.938	**2.113**
Pf.13_1.523.0_CDS_a_at	845	**2.684**	**2.481**	1.627
Pf.13_1.524.0_CDS_at	420	1.741	1.377	**0.256**
Pf.13_1.525.0_CDS_at	1004	**0.265**	**0.428**	0.737
Pf.13_1.526.0_CDS_at	10	0.943	0.664	0.816
Pf.13_1.528.0_CDS_at	119	1.021	0.856	1.023
Pf.13_1.531.0_CDS_at	119	1.216	1.201	**2.839**
Pf.13_1.533.0_CDS_at	10	0.800	0.657	0.753
Pf.13_1.534.0_CDS_at	27	**0.412**	0.631	1.164
Pf.13_1.535.0_CDS_at	270	0.679	1.725	1.856
Pf.13_1.537.0_CDS_at	156	**4.451**	**3.272**	**4.081**
Pf.13_1.539.0_CDS_at	6	0.814	0.824	0.792
Pf.13_1.54.0_CDS_at	1184	1.099	1.741	1.218
Pf.13_1.540.0_CDS_at	95	0.937	1.013	1.406
Pf.13_1.542.0_CDS_at	120	0.588	1.002	1.796
Pf.13_1.543.0_CDS_at	253	1.871	**0.227**	**0.295**
Pf.13_1.545.0_CDS_at	5	1.169	0.991	1.076
Pf.13_1.546.0_CDS_at	5	1.193	1.086	1.052
Pf.13_1.547.0_CDS_x_at	8	1.321	0.894	1.041
Pf.13_1.548.0_CDS_at	7	0.786	0.866	0.824
Pf.13_1.548.0_CDS_s_at	5	1.077	1.356	1.198
Pf.13_1.549.0_CDS_at	6	0.835	0.876	0.822
Pf.13_1.549.0_CDS_x_at	6	0.893	0.910	0.791
Pf.13_1.551.0_CDS_at	10	0.983	1.113	0.888
Pf.13_1.552.0_CDS_s_at	5	1.105	1.253	1.275
Pf.13_1.555.0_CDS_x_at	728	1.549	0.672	0.793
Pf.13_1.557.0_CDS_at	19	1.753	0.597	0.860
Pf.13_1.558.0_CDS_at	655	1.312	1.588	1.692
Pf.13_1.559.0_CDS_at	12	0.534	1.064	0.538
Pf.13_1.56.0_CDS_a_at	1509	0.641	0.976	0.625
Pf.13_1.560.0_CDS_at	53	1.003	1.157	1.442
Pf.13_1.561.0_CDS_at	97	0.705	1.357	1.955
Pf.13_1.562.0_CDS_at	194	**0.283**	**0.185**	**0.234**
Pf.13_1.563.0_CDS_at	12	1.658	1.640	**2.199**
Pf.13_1.566.0_CDS_at	590	0.661	0.798	**0.481**
Pf.13_1.567.0_CDS_at	380	1.153	0.776	1.671
Pf.13_1.568.0_CDS_at	234	0.766	1.290	**2.713**

Pf.13_1.570.0_CDS_at	14	1.734	0.807	1.179
Pf.13_1.575.0_CDS_at	89	0.545	0.866	0.858
Pf.13_1.575.0_CDS_x_at	124	0.639	0.889	1.097
Pf.13_1.576.0_CDS_at	6	1.160	1.176	1.297
Pf.13_1.578.0_CDS_at	5	1.476	1.330	1.331
Pf.13_1.578.0_CDS_x_at	6	1.115	1.069	0.971
Pf.13_1.579.0_CDS_at	25	1.279	0.862	1.207
Pf.13_1.58.0_CDS_at	5308	1.358	1.275	1.719
Pf.13_1.580.0_CDS_at	1057	0.915	0.930	1.579
Pf.13_1.581.0_CDS_at	1271	**0.143**	**0.277**	**0.360**
Pf.13_1.582.0_CDS_at	38	0.769	0.786	1.963
Pf.13_1.583.0_CDS_at	247	0.882	1.206	1.834
Pf.13_1.584.0_CDS_at	47	1.585	**2.202**	**2.769**
Pf.13_1.586.0_CDS_at	1054	**0.480**	**0.183**	**0.497**
Pf.13_1.588.0_CDS_at	83	1.330	1.887	**2.184**
Pf.13_1.589.0_CDS_at	21	1.009	0.839	0.929
Pf.13_1.591.0_CDS_at	241	1.790	1.081	1.609
Pf.13_1.592.0_CDS_at	10	1.131	1.037	1.166
Pf.13_1.593.0_CDS_at	15	0.964	1.226	**2.164**
Pf.13_1.593.0_CDS_x_at	137	0.667	1.274	1.935
Pf.13_1.595.0_CDS_at	271	0.977	0.799	0.510
Pf.13_1.596.0_CDS_at	100	**0.311**	0.736	**2.946**
Pf.13_1.599.0_CDS_at	444	0.801	1.077	0.845
Pf.13_1.6.0_CDS_at	8744	**0.492**	0.812	0.990
Pf.13_1.601.0_CDS_at	6	1.784	1.681	**2.382**
Pf.13_1.602.0_CDS_a_at	709	1.118	0.703	1.591
Pf.13_1.603.0_CDS_at	12	0.989	1.126	**2.248**
Pf.13_1.604.0_CDS_at	358	0.922	0.713	1.676
Pf.13_1.605.0_CDS_at	59	1.432	**2.403**	**0.481**
Pf.13_1.606.0_CDS_at	1979	**0.211**	**0.405**	0.988
Pf.13_1.607.0_CDS_at	266	0.947	1.275	0.960
Pf.13_1.608.0_CDS_at	15	1.502	1.227	0.607
Pf.13_1.608.0_CDS_x_at	13	1.191	1.297	**0.482**
Pf.13_1.609.0_CDS_at	43	1.366	0.745	**0.352**
Pf.13_1.610.0_CDS_at	8	1.487	1.122	0.827
Pf.13_1.611.0_CDS_at	35	0.962	0.751	0.777
Pf.13_1.612.0_CDS_at	174	1.226	1.202	0.701
Pf.13_1.613.0_CDS_at	264	**0.383**	1.260	**2.236**
Pf.13_1.614.0_CDS_at	374	1.503	**2.092**	**2.804**
Pf.13_1.615.0_CDS_at	148	**3.040**	0.638	0.600
Pf.13_1.616.0_CDS_at	17	0.631	**0.379**	**0.341**
Pf.13_1.618.0_CDS_at	115	0.866	**0.113**	**0.394**
Pf.13_1.619.0_CDS_at	15	**2.852**	0.591	0.863
Pf.13_1.621.0_CDS_at	333	1.385	1.712	**2.244**
Pf.13_1.623.0_CDS_at	219	1.261	0.906	0.516
Pf.13_1.624.0_CDS_at	399	1.366	**3.055**	**5.440**
Pf.13_1.625.0_CDS_at	481	**0.424**	0.981	**2.100**
Pf.13_1.626.0_CDS_at	25	**0.491**	**0.472**	**0.354**
Pf.13_1.627.0_CDS_at	814	1.599	1.022	1.258
Pf.13_1.629.0_CDS_at	1039	**0.304**	0.641	**1.522**
Pf.13_1.63.0_CDS_at	2292	0.961	0.966	**1.561**
Pf.13_1.630.0_CDS_at	8	1.247	0.864	0.927
Pf.13_1.632.0_CDS_s_at	10	1.222	0.975	0.836
Pf.13_1.633.0_CDS_at	115	1.819	**0.366**	0.549
Pf.13_1.634.0_CDS_at	98	**0.084**	**0.152**	**0.364**
Pf.13_1.635.0_CDS_at	10	0.709	0.580	0.543
Pf.13_1.636.0_CDS_at	166	**0.268**	**0.355**	**0.233**
Pf.13_1.637.0_CDS_at	439	**2.490**	**4.211**	**2.945**
Pf.13_1.638.0_CDS_at	227	**0.403**	**0.328**	0.824
Pf.13_1.639.0_CDS_at	44	**10.616**	**21.182**	**2.639**
Pf.13_1.64.0_CDS_a_at	2553	0.868	0.914	1.086
Pf.13_1.640.0_CDS_at	201	1.561	1.096	1.751

Pf.13_1.642.0_CDS_at	39	1.265	0.946	0.844
Pf.13_1.643.0_CDS_at	42	**4.655**	1.930	1.049
Pf.13_1.644.0_CDS_at	1115	**2.954**	**3.618**	**2.630**
Pf.13_1.645.0_CDS_at	175	**2.948**	**3.229**	**3.326**
Pf.13_1.646.0_CDS_at	1955	0.855	0.983	1.358
Pf.13_1.65.0_CDS_at	432	0.701	1.304	1.101
Pf.13_1.650.0_CDS_at	325	1.401	**0.391**	**0.386**
Pf.13_1.651.0_CDS_at	54	**4.270**	**3.441**	**4.674**
Pf.13_1.652.0_CDS_at	216	0.586	0.987	0.846
Pf.13_1.653.0_CDS_s_at	10474	1.015	0.882	1.220
Pf.13_1.654.0_CDS_at	48	1.436	1.573	1.642
Pf.13_1.656.0_CDS_at	60	0.645	0.876	0.867
Pf.13_1.657.0_CDS_at	478	0.603	0.814	**2.311**
Pf.13_1.66.0_CDS_at	51	1.590	**0.372**	0.551
Pf.13_1.660.0_CDS_at	17	1.437	1.908	**2.851**
Pf.13_1.661.0_CDS_at	189	**0.466**	**0.173**	**0.249**
Pf.13_1.663.0_CDS_at	56	**3.400**	1.057	1.229
Pf.13_1.664.0_CDS_at	18	0.679	0.514	1.290
Pf.13_1.666.0_CDS_at	1799	0.818	1.218	1.461
Pf.13_1.667.0_CDS_a_at	9	1.692	1.663	0.992
Pf.13_1.667.2_a_at	122	**2.467**	1.085	1.479
Pf.13_1.668.0_CDS_at	140	0.537	1.159	**2.511**
Pf.13_1.669.0_CDS_at	885	**0.368**	0.592	1.303
Pf.13_1.67.0_CDS_at	681	**0.281**	**0.351**	0.658
Pf.13_1.670.0_CDS_at	8889	0.675	0.897	1.284
Pf.13_1.671.0_CDS_at	681	0.950	1.098	1.815
Pf.13_1.672.0_CDS_at	26	0.957	**0.348**	0.794
Pf.13_1.673.0_CDS_at	190	1.977	1.661	**2.093**
Pf.13_1.674.0_CDS_at	46	1.814	1.181	1.665
Pf.13_1.674.0_CDS_x_at	64	**2.197**	1.249	**2.137**
Pf.13_1.676.0_CDS_at	9	1.004	0.672	0.617
Pf.13_1.68.0_CDS_at	592	**0.161**	**0.259**	**0.262**
Pf.13_1.688.0_at	16	0.947	0.577	0.575
Pf.13_1.69.0_CDS_at	2477	0.956	1.106	1.378
Pf.13_1.7.0_CDS_at	421	**2.123**	1.711	**2.339**
Pf.13_1.70.0_CDS_at	7	1.028	0.886	0.939
Pf.13_1.701.0_at	8	0.870	0.824	1.290
Pf.13_1.71.0_CDS_at	42	1.581	0.537	0.528
Pf.13_1.72.0_CDS_at	628	**0.190**	**0.182**	**0.118**
Pf.13_1.722.0_at	102	1.389	1.129	1.287
Pf.13_1.73.0_CDS_at	2945	**0.104**	**0.123**	**0.105**
Pf.13_1.733.0_at	432	1.748	1.120	1.172
Pf.13_1.74.0_CDS_at	38	1.028	1.157	1.125
Pf.13_1.75.0_CDS_at	24	1.645	0.861	1.176
Pf.13_1.76.0_CDS_at	6971	0.794	0.878	1.062
Pf.13_1.761.0_at	603	1.275	1.145	1.168
Pf.13_1.77.0_CDS_at	3418	1.891	1.585	1.320
Pf.13_1.78.0_CDS_at	74	**0.440**	0.781	**0.330**
Pf.13_1.79.0_CDS_at	1790	**0.101**	**0.106**	**0.071**
Pf.13_1.796.0_at	15	1.063	0.935	1.263
Pf.13_1.8.0_CDS_at	8675	**0.052**	**0.103**	**0.033**
Pf.13_1.806.0_at	10	0.934	0.867	0.908
Pf.13_1.810.0_at	86	0.932	0.851	1.482
Pf.13_1.82.0_CDS_at	351	1.518	**2.244**	**2.069**
Pf.13_1.821.0_at	284	**0.262**	**0.239**	1.336
Pf.13_1.83.0_CDS_at	768	0.534	0.637	0.863
Pf.13_1.84.0_CDS_a_at	717	**0.284**	**0.247**	**0.138**
Pf.13_1.85.0_CDS_at	268	0.552	0.755	1.231
Pf.13_1.853.0_at	174	**3.155**	1.492	1.145
Pf.13_1.86.0_CDS_at	12	0.616	0.550	0.758
Pf.13_1.88.0_CDS_at	209	1.110	1.213	**2.177**
Pf.13_1.89.0_CDS_at	583	**0.258**	**0.317**	0.894

Pf.13_1.90.0_CDS_at	266	**0.437**	0.744	1.107
Pf.13_1.91.0_CDS_at	61	1.188	1.680	**2.045**
Pf.13_1.92.0_CDS_at	1396	1.975	0.722	**0.487**
Pf.13_1.93.0_CDS_at	43	0.725	0.661	**2.058**
Pf.13_1.95.0_CDS_at	412	**0.223**	**0.419**	**0.376**
Pf.13_1.96.0_CDS_at	1022	1.706	1.912	1.659
Pf.13_1.97.0_CDS_at	90	0.769	1.356	0.791
Pf.13_1.98.0_CDS_a_at	5833	**0.401**	0.946	1.186
Pf.13_1.99.0_CDS_at	473	0.620	1.058	**0.499**
Pf.13_1.99.0_CDS_x_at	646	0.645	1.109	**0.412**
Pf.13_2.678.0_CDS_s_at	22	1.330	0.684	**0.499**
Pf.13_2.679.0_CDS_s_at	15	1.278	0.553	0.757
Pf.13_2.680.0_CDS_s_at	6	1.037	0.859	0.885
Pf.13_2.681.0_CDS_x_at	5	0.970	0.983	0.898
Pf.13_2.682.0_CDS_s_at	6	0.910	0.761	0.860
Pf.14.1.0_CDS_at	18218	1.058	1.003	1.022
Pf.14.10.0_CDS_at	473	0.829	0.815	0.896
Pf.14.101.0_CDS_at	360	0.565	**0.383**	0.796
Pf.14.102.0_CDS_at	47	0.742	1.071	1.029
Pf.14.104.0_CDS_a_at	63	0.745	0.677	**3.313**
Pf.14.104.0_UTR_a_at	43	0.963	1.474	1.013
Pf.14.105.0_CDS_at	8342	1.430	1.326	1.248
Pf.14.108.0_CDS_at	28	1.402	1.442	1.449
Pf.14.109.0_CDS_at	490	**0.396**	0.680	**0.244**
Pf.14.11.0_CDS_at	1081	**0.419**	**0.321**	1.238
Pf.14.110.0_CDS_at	3365	0.845	0.878	1.449
Pf.14.111.0_CDS_at	17	0.979	0.678	0.550
Pf.14.112.0_CDS_at	8	0.968	0.757	0.949
Pf.14.113.0_CDS_at	7	0.824	0.755	2.217
Pf.14.113.0_CDS_x_at	31	**0.473**	0.643	1.981
Pf.14.114.0_CDS_at	15	1.241	0.919	0.733
Pf.14.116.0_CDS_at	142	0.555	0.690	**0.390**
Pf.14.117.0_CDS_at	551	**2.019**	**2.063**	**2.169**
Pf.14.12.0_CDS_at	9713	0.871	0.839	1.094
Pf.14.120.0_CDS_at	8	0.833	0.926	0.949
Pf.14.121.0_CDS_at	7	1.329	0.896	1.044
Pf.14.122.0_CDS_at	235	**0.160**	0.557	0.661
Pf.14.123.0_CDS_at	382	0.691	0.927	1.497
Pf.14.124.0_CDS_at	1506	0.983	1.049	0.558
Pf.14.125.0_CDS_at	1685	1.696	1.417	1.604
Pf.14.126.0_CDS_at	460	**0.364**	0.595	0.966
Pf.14.127.2_a_at	14	0.694	0.670	0.739
Pf.14.128.0_CDS_at	166	1.491	0.843	0.933
Pf.14.129.0_CDS_at	156	0.545	1.095	0.810
Pf.14.13.0_CDS_at	413	1.071	1.878	0.587
Pf.14.131.0_CDS_at	13	0.766	0.904	0.640
Pf.14.132.0_CDS_at	467	1.634	**2.109**	**2.049**
Pf.14.133.0_CDS_at	94	0.827	1.020	0.998
Pf.14.134.0_CDS_at	672	**6.777**	**4.393**	1.490
Pf.14.135.0_CDS_at	357	0.655	0.942	0.928
Pf.14.136.0_CDS_at	586	1.107	1.394	1.017
Pf.14.137.0_CDS_at	139	**1.653**	1.340	**0.338**
Pf.14.139.0_CDS_a_at	30	**0.346**	**0.330**	**0.216**
Pf.14.14.0_CDS_at	139	0.831	**0.459**	**0.347**
Pf.14.140.0_CDS_at	820	**2.467**	1.200	1.290
Pf.14.142.0_CDS_at	193	1.749	1.087	0.657
Pf.14.143.0_CDS_at	3266	**0.256**	**0.423**	**0.431**
Pf.14.143.0_CDS_x_at	2251	**0.247**	**0.419**	0.517
Pf.14.144.0_CDS_at	23	**2.447**	0.703	**0.456**
Pf.14.146.0_CDS_at	2933	0.772	0.770	1.058
Pf.14.147.0_CDS_at	34	1.505	0.793	0.912
Pf.14.148.0_CDS_at	1628	0.939	0.856	1.171

Pf.14.149.0_CDS_at	80	**0.320**	1.074	1.195
Pf.14.15.0_CDS_at	344	**0.271**	1.016	1.367
Pf.14.150.0_CDS_at	270	**0.127**	**0.218**	0.593
Pf.14.151.0_CDS_at	126	**0.449**	0.774	**2.229**
Pf.14.151.0_CDS_x_at	115	0.510	0.745	**2.211**
Pf.14.153.0_CDS_at	203	0.978	1.403	1.505
Pf.14.154.0_CDS_at	164	**3.355**	**2.531**	**2.660**
Pf.14.155.0_CDS_at	158	1.982	**2.750**	**3.600**
Pf.14.156.0_CDS_at	8141	1.256	1.214	1.198
Pf.14.157.0_CDS_at	19	0.611	0.614	0.732
Pf.14.157.0_CDS_x_at	22	0.932	0.843	1.265
Pf.14.158.0_CDS_at	690	0.963	1.884	1.044
Pf.14.16.0_CDS_at	877	0.677	1.122	0.583
Pf.14.160.0_CDS_at	763	1.203	0.962	1.605
Pf.14.161.0_CDS_at	71	0.502	1.146	1.460
Pf.14.162.0_CDS_at	160	**0.499**	0.752	0.890
Pf.14.163.0_CDS_at	6	1.118	1.349	0.833
Pf.14.164.0_CDS_at	3278	0.805	0.886	1.447
Pf.14.166.0_CDS_at	7	2.027	1.376	1.675
Pf.14.167.0_CDS_at	14	0.608	1.256	**2.070**
Pf.14.168.0_CDS_at	12	0.871	0.870	0.743
Pf.14.169.0_CDS_at	484	**0.455**	0.740	0.702
Pf.14.17.0_CDS_at	9292	1.182	0.889	1.093
Pf.14.170.0_CDS_at	1037	1.046	1.120	**0.443**
Pf.14.171.0_CDS_at	528	**2.405**	**2.283**	**2.210**
Pf.14.172.0_CDS_at	115	0.986	**0.264**	**0.245**
Pf.14.173.0_CDS_a_at	73	1.054	1.126	1.145
Pf.14.174.0_CDS_at	105	1.040	1.261	1.237
Pf.14.174.0_CDS_x_at	188	0.967	0.945	1.069
Pf.14.175.0_CDS_at	49	0.534	0.930	0.625
Pf.14.176.0_CDS_a_at	352	**5.684**	**3.432**	1.496
Pf.14.177.0_CDS_at	177	**2.198**	**3.167**	**3.342**
Pf.14.178.0_CDS_at	60	0.694	1.151	**3.423**
Pf.14.18.0_CDS_at	285	1.118	1.354	1.336
Pf.14.180.0_CDS_at	979	**0.131**	**0.216**	**0.151**
Pf.14.181.0_CDS_at	8	1.413	0.869	0.950
Pf.14.182.0_CDS_at	10	1.229	1.778	0.685
Pf.14.183.0_CDS_at	18	1.047	0.845	1.543
Pf.14.184.0_CDS_at	45	0.913	1.072	0.973
Pf.14.186.0_CDS_at	299	1.218	0.767	1.124
Pf.14.187.0_CDS_at	54	**0.282**	0.674	1.754
Pf.14.188.0_CDS_at	21	**0.461**	0.772	1.122
Pf.14.189.0_CDS_at	6012	1.106	1.001	1.318
Pf.14.19.0_CDS_at	5888	1.001	0.965	1.078
Pf.14.190.0_CDS_at	1886	1.879	1.074	1.530
Pf.14.190.0_UTR_at	1347	1.701	1.217	1.724
Pf.14.191.0_CDS_at	19	0.531	1.042	0.619
Pf.14.192.0_CDS_at	8	0.854	0.736	0.889
Pf.14.193.0_CDS_at	390	1.030	1.714	**2.257**
Pf.14.194.0_CDS_at	336	0.646	1.032	0.865
Pf.14.195.0_CDS_at	210	0.925	1.372	1.335
Pf.14.196.0_CDS_at	21	0.643	0.546	**0.482**
Pf.14.198.0_CDS_at	645	0.673	1.131	1.870
Pf.14.2.0_CDS_at	3230	**0.029**	**0.072**	**0.035**
Pf.14.200.0_CDS_at	275	1.398	**0.633**	**0.361**
Pf.14.201.0_CDS_at	287	**2.477**	**2.358**	1.576
Pf.14.202.0_CDS_at	48	1.698	**0.451**	**0.260**
Pf.14.203.0_CDS_at	145	0.705	1.030	1.208
Pf.14.203.0_CDS_x_at	190	0.546	0.951	1.129
Pf.14.205.0_CDS_at	11	1.138	0.664	0.834
Pf.14.205.0_CDS_x_at	11	0.825	0.545	0.804
Pf.14.206.0_CDS_at	6477	**0.482**	0.865	1.238

Pf.14.207.0_CDS_at	7	0.875	0.853	0.951
Pf.14.208.0_CDS_at	169	1.452	1.998	**3.039**
Pf.14.209.0_CDS_at	1364	**2.636**	**2.486**	1.901
Pf.14.21.0_CDS_a_at	119	0.529	**0.416**	1.729
Pf.14.210.0_CDS_at	2222	**0.375**	0.789	1.287
Pf.14.211.0_CDS_at	114	0.804	0.569	1.177
Pf.14.212.0_CDS_at	533	**0.304**	**0.366**	**0.362**
Pf.14.213.0_CDS_at	688	0.581	1.481	1.462
Pf.14.214.0_CDS_at	2587	**0.234**	**0.499**	0.705
Pf.14.215.0_CDS_at	1151	**2.684**	1.750	1.286
Pf.14.216.0_CDS_at	1057	**2.052**	1.756	1.361
Pf.14.217.0_CDS_at	8	0.867	0.732	0.817
Pf.14.218.0_CDS_at	1585	0.782	1.334	1.084
Pf.14.219.0_CDS_at	39	**3.333**	**5.173**	**4.754**
Pf.14.221.0_CDS_at	42	**0.404**	1.047	0.726
Pf.14.223.0_CDS_at	8	0.950	0.880	0.999
Pf.14.224.0_CDS_at	19	**5.727**	**4.632**	**2.543**
Pf.14.224.0_CDS_x_at	214	**4.354**	**3.424**	1.980
Pf.14.225.0_CDS_at	92	1.810	0.952	1.100
Pf.14.226.0_CDS_at	1061	**0.488**	0.930	1.555
Pf.14.228.0_CDS_at	424	**0.105**	**0.231**	**0.153**
Pf.14.229.0_CDS_at	321	0.585	0.878	**2.078**
Pf.14.23.0_CDS_at	734	**2.087**	**2.114**	1.657
Pf.14.230.0_CDS_at	268	0.928	**0.412**	**0.386**
Pf.14.231.0_CDS_at	1753	**2.069**	1.339	1.727
Pf.14.232.0_CDS_at	770	0.878	0.705	0.558
Pf.14.233.0_CDS_at	39	0.881	0.701	1.096
Pf.14.233.1_CDS_at	80	0.629	**0.489**	0.699
Pf.14.234.0_CDS_at	13	**0.344**	**0.395**	0.765
Pf.14.236.0_CDS_at	20	0.791	1.309	0.584
Pf.14.237.0_CDS_at	482	1.215	1.922	1.446
Pf.14.238.0_CDS_at	112	0.983	1.414	**2.194**
Pf.14.239.0_CDS_at	153	0.786	1.205	1.507
Pf.14.24.0_CDS_at	8	0.782	0.647	0.858
Pf.14.240.0_CDS_at	8	1.248	0.737	1.277
Pf.14.241.0_CDS_a_at	258	1.397	**2.050**	1.617
Pf.14.243.0_CDS_at	90	**0.268**	**0.196**	**0.202**
Pf.14.245.0_CDS_at	159	**0.187**	0.515	**0.281**
Pf.14.247.0_CDS_at	11	0.510	**0.492**	0.635
Pf.14.249.0_CDS_at	125	**3.685**	1.735	**0.348**
Pf.14.25.0_CDS_at	41	**0.270**	**0.360**	**0.120**
Pf.14.250.0_CDS_at	102	1.577	1.799	**2.745**
Pf.14.252.0_CDS_at	1651	**0.249**	**0.102**	**0.439**
Pf.14.254.0_CDS_at	9491	0.742	1.052	1.286
Pf.14.256.0_CDS_a_at	198	1.594	1.767	1.728
Pf.14.257.0_CDS_at	413	**0.385**	**0.487**	0.937
Pf.14.258.0_CDS_at	34	**2.637**	1.749	**2.946**
Pf.14.259.0_CDS_at	1249	**0.362**	**0.364**	1.023
Pf.14.26.0_CDS_at	1146	**0.058**	**0.070**	**0.112**
Pf.14.260.0_CDS_at	507	0.816	0.964	1.980
Pf.14.261.0_CDS_at	43	**2.041**	**3.448**	**2.403**
Pf.14.262.0_CDS_at	200	0.619	0.919	0.963
Pf.14.263.0_CDS_at	591	0.617	0.738	1.333
Pf.14.264.0_CDS_at	223	**2.410**	1.944	**2.230**
Pf.14.265.0_CDS_a_at	740	1.108	1.452	**2.044**
Pf.14.267.0_CDS_at	187	1.718	0.998	**2.050**
Pf.14.268.0_CDS_at	41	1.838	**2.136**	**3.270**
Pf.14.269.0_CDS_at	480	0.983	1.854	1.305
Pf.14.27.0_CDS_at	15	1.294	1.992	1.073
Pf.14.270.0_CDS_at	543	**0.253**	0.655	**0.232**
Pf.14.271.0_CDS_at	317	**0.154**	0.512	**0.291**
Pf.14.272.0_CDS_at	257	**3.542**	**2.818**	**3.821**

Pf.14.273.0_CDS_at	26	0.680	**0.463**	0.805
Pf.14.274.0_CDS_at	51	1.767	**3.001**	**4.253**
Pf.14.275.0_CDS_a_at	517	**0.457**	0.718	1.018
Pf.14.277.0_CDS_at	1152	0.575	0.884	1.666
Pf.14.279.0_CDS_at	60	0.871	1.789	**2.339**
Pf.14.280.0_CDS_at	249	0.615	0.991	1.674
Pf.14.280.0_CDS_x_at	250	0.556	0.842	1.966
Pf.14.281.0_CDS_at	23	0.659	0.838	**2.227**
Pf.14.282.0_CDS_at	26	**0.317**	**0.381**	**0.326**
Pf.14.283.0_CDS_at	126	1.883	0.773	0.879
Pf.14.285.0_CDS_at	14	0.846	0.551	0.646
Pf.14.286.0_CDS_at	125	**0.413**	1.700	0.807
Pf.14.288.0_CDS_at	229	0.918	1.339	**2.081**
Pf.14.289.0_CDS_at	86	1.431	1.151	0.784
Pf.14.29.0_CDS_at	98	**5.348**	**4.853**	1.983
Pf.14.290.0_CDS_at	598	**0.134**	**0.209**	**0.407**
Pf.14.291.0_CDS_at	26	0.912	**2.088**	1.512
Pf.14.293.0_CDS_at	22	1.356	1.183	**0.366**
Pf.14.294.0_CDS_at	34	0.573	1.143	1.492
Pf.14.295.0_CDS_at	15	1.154	**0.487**	0.523
Pf.14.296.0_CDS_at	201	0.661	1.515	**2.147**
Pf.14.297.0_CDS_at	40	1.719	1.765	0.690
Pf.14.298.0_CDS_at	198	**0.432**	0.644	1.309
Pf.14.3.0_CDS_at	6624	0.896	0.795	1.185
Pf.14.30.0_CDS_at	1442	**0.469**	0.696	0.517
Pf.14.300.0_CDS_at	72	**5.937**	**2.781**	**3.674**
Pf.14.301.0_CDS_at	6399	1.481	0.915	1.336
Pf.14.302.0_CDS_at	350	**2.114**	**2.157**	**3.018**
Pf.14.303.0_CDS_at	68	1.377	1.101	1.395
Pf.14.306.0_CDS_at	38	1.186	1.589	**2.736**
Pf.14.307.0_CDS_at	265	1.915	**2.199**	**2.444**
Pf.14.31.0_CDS_at	414	**0.426**	**0.466**	0.776
Pf.14.310.0_CDS_at	32	**0.421**	0.772	1.335
Pf.14.311.0_CDS_at	1616	**0.387**	0.505	0.968
Pf.14.312.0_CDS_at	227	**0.363**	**0.424**	0.713
Pf.14.313.0_CDS_at	148	1.271	**2.036**	**2.719**
Pf.14.314.0_CDS_at	145	**0.154**	**0.520**	**0.378**
Pf.14.315.0_CDS_at	133	0.608	0.817	**0.452**
Pf.14.316.0_CDS_at	97	1.620	1.653	0.924
Pf.14.316.1_CDS_at	9	1.148	0.979	0.893
Pf.14.317.0_CDS_at	11	0.906	0.934	0.800
Pf.14.318.0_CDS_at	161	**3.428**	1.723	**0.304**
Pf.14.319.0_CDS_at	5	0.999	0.961	0.843
Pf.14.32.0_CDS_at	342	0.896	0.831	1.423
Pf.14.320.0_CDS_at	137	0.533	0.668	0.607
Pf.14.321.1_CDS_a_at	19	0.956	**0.486**	**0.437**
Pf.14.322.0_CDS_at	282	**0.441**	1.020	**0.446**
Pf.14.324.0_CDS_at	201	**0.327**	**0.475**	**0.400**
Pf.14.325.0_CDS_at	10	1.437	1.772	1.680
Pf.14.326.0_CDS_at	26	0.703	0.964	1.359
Pf.14.327.0_CDS_at	19	0.564	**0.252**	0.512
Pf.14.328.0_CDS_at	19	1.514	0.992	1.104
Pf.14.33.0_CDS_a_at	22	**0.364**	**0.384**	**0.263**
Pf.14.330.0_CDS_at	162	0.539	1.169	**0.153**
Pf.14.331.0_CDS_at	8	0.699	0.782	0.763
Pf.14.332.0_CDS_at	474	1.591	1.430	1.193
Pf.14.333.0_CDS_at	27	0.515	**0.369**	**0.492**
Pf.14.336.0_CDS_at	124	0.544	0.820	0.711
Pf.14.337.0_CDS_at	22	1.316	0.506	0.548
Pf.14.338.0_CDS_at	37	**5.137**	1.588	1.162
Pf.14.339.0_CDS_at	7	1.153	1.088	0.917
Pf.14.34.0_CDS_at	542	1.660	0.723	1.613

Pf.14.340.0_CDS_at	164	0.818	1.546	**2.060**	
Pf.14.341.0_CDS_at	516	0.706	0.829	0.570	
Pf.14.343.0_CDS_at	103	1.859	**2.366**	**3.010**	
Pf.14.345.0_CDS_at	1164	1.026	1.231	1.440	
Pf.14.346.0_CDS_at	13	0.993	0.539	0.648	
Pf.14.347.0_CDS_at	45	**2.505**	**5.074**	**2.501**	
Pf.14.349.0_CDS_at	19	0.551	0.731	0.367	
Pf.14.35.0_CDS_at	338	1.396	1.349	1.638	
Pf.14.350.0_CDS_a_at	64	0.867	0.688	1.945	
Pf.14.351.0_CDS_at	169	0.739	0.962	0.727	
Pf.14.352.0_CDS_at	38	0.569	0.676	0.629	
Pf.14.354.0_CDS_at	1096	**3.312**	**2.791**	1.832	
Pf.14.355.0_CDS_at	390	0.824	1.601	**0.457**	
Pf.14.357.0_CDS_at	444	1.028	0.703	1.476	
Pf.14.358.0_CDS_at	55	0.605	0.696	0.777	
Pf.14.360.0_CDS_at	80	1.278	1.962	**2.364**	
Pf.14.361.0_CDS_at	81	**0.409**	0.680	0.964	
Pf.14.362.0_CDS_at	47	1.026	1.594	1.106	
Pf.14.363.0_CDS_at	21	1.481	1.192	**2.476**	
Pf.14.365.0_CDS_at	276	1.343	1.330	1.462	
Pf.14.366.0_CDS_at	85	**2.810**	0.700	0.559	
Pf.14.368.0_CDS_at	1593	1.147	1.675	1.818	
Pf.14.369.0_CDS_at	14	1.456	0.653	0.840	
Pf.14.37.1_CDS_a_at	74	**0.105**	**0.108**	**0.192**	
Pf.14.37.1_UTR_a_at	1601	**0.206**	**0.090**	**0.141**	
Pf.14.372.0_CDS_at	166	0.507	0.700	0.980	
Pf.14.374.0_CDS_at	9	1.031	2.167	**2.233**	
Pf.14.375.0_CDS_at	155	**0.310**	0.694	1.284	
Pf.14.376.0_CDS_at	32	0.802	0.887	0.995	
Pf.14.377.0_CDS_at	1769	**2.293**	**2.067**	**2.362**	
Pf.14.378.0_CDS_at	487	0.949	1.124	1.798	
Pf.14.379.0_CDS_at	193	0.883	1.215	1.503	
Pf.14.38.0_CDS_at	2347	**0.085**	**0.316**	0.717	#4
Pf.14.380.0_CDS_at	3861	**0.224**	**0.476**	1.119	#5
Pf.14.383.0_CDS_at	196	**0.295**	0.859	1.336	
Pf.14.384.0_CDS_at	268	1.536	**2.118**	1.801	
Pf.14.385.0_CDS_at	824	**2.080**	**2.243**	**2.227**	
Pf.14.386.0_CDS_at	3805	1.459	1.512	1.379	
Pf.14.387.0_CDS_at	33	**0.324**	**0.248**	**0.225**	
Pf.14.389.0_CDS_at	64	0.985	**0.445**	0.823	
Pf.14.39.0_CDS_at	883	**2.952**	**2.403**	1.786	
Pf.14.390.0_CDS_at	607	0.832	0.502	**2.003**	
Pf.14.392.0_CDS_at	40	**0.331**	**0.186**	**0.285**	
Pf.14.393.0_CDS_at	184	**2.901**	**4.199**	1.552	
Pf.14.396.0_CDS_at	95	1.435	0.944	1.684	
Pf.14.397.0_CDS_at	454	1.288	1.327	**2.392**	
Pf.14.398.0_CDS_at	89	0.638	**2.856**	1.463	
Pf.14.4.0_CDS_at	10818	0.913	0.756	0.904	
Pf.14.40.0_CDS_a_at	213	**0.162**	**0.244**	**0.163**	
Pf.14.400.0_CDS_at	134	1.212	**2.362**	**2.756**	
Pf.14.401.0_CDS_at	32	1.250	**0.450**	0.897	
Pf.14.402.0_CDS_at	6	1.058	0.898	0.910	
Pf.14.403.0_CDS_at	15	0.560	0.603	0.702	
Pf.14.404.0_CDS_at	67	1.061	0.614	**0.249**	
Pf.14.405.0_CDS_at	63	1.361	0.705	**0.281**	
Pf.14.406.0_CDS_at	69	**0.481**	1.517	**2.076**	
Pf.14.407.0_CDS_x_at	231	1.264	**0.206**	**0.302**	
Pf.14.408.0_CDS_s_at	6869	1.252	0.634	**0.295**	
Pf.14.41.0_CDS_a_at	314	**0.361**	0.748	0.690	
Pf.14.41.0_CDS_x_at	300	**0.344**	0.645	0.605	
Pf.14.412.0_CDS_at	528	1.008	1.372	1.842	
Pf.14.413.0_CDS_at	188	**2.161**	1.000	0.913	
Pf.14.416.0_CDS_at	111	**0.135**	**0.082**	**0.111**	

Pf.14.417.0_CDS_at	14	0.654	0.674	1.238
Pf.14.419.0_CDS_at	5	1.164	1.097	1.061
Pf.14.420.0_CDS_at	40	**0.367**	**0.239**	**0.237**
Pf.14.420.1_CDS_a_at	142	**0.333**	**0.234**	**0.227**
Pf.14.421.0_CDS_a_at	100	1.915	1.580	1.920
Pf.14.422.0_CDS_at	9	0.741	1.145	1.200
Pf.14.423.0_CDS_at	6	1.249	0.888	1.002
Pf.14.424.0_CDS_at	213	**2.013**	1.453	1.940
Pf.14.425.0_CDS_at	1036	**2.858**	**2.596**	**2.090**
Pf.14.428.0_CDS_at	125	1.699	**2.010**	1.174
Pf.14.429.0_CDS_at	49	**0.285**	**0.209**	**0.435**
Pf.14.43.0_CDS_at	438	**2.338**	**2.205**	**2.153**
Pf.14.430.0_CDS_at	17	1.743	0.510	0.727
Pf.14.431.0_CDS_at	126	**0.428**	**0.409**	0.719
Pf.14.432.0_CDS_at	821	0.798	1.008	1.186
Pf.14.433.0_CDS_at	31	0.764	**0.358**	1.446
Pf.14.434.0_CDS_at	8	1.172	0.911	1.064
Pf.14.435.0_CDS_at	16	1.065	0.558	0.833
Pf.14.436.0_CDS_at	23	1.269	1.422	0.725
Pf.14.437.0_CDS_at	59	1.719	**2.968**	**3.859**
Pf.14.438.0_CDS_at	45	**0.346**	**0.220**	0.873
Pf.14.438.0_CDS_x_at	76	**0.201**	**0.191**	0.840
Pf.14.44.0_CDS_at	200	**0.259**	0.678	0.607
Pf.14.440.0_CDS_at	51	**0.441**	**0.330**	**0.389**
Pf.14.441.0_CDS_at	837	0.835	0.920	**2.178**
Pf.14.442.0_CDS_at	237	1.980	1.682	1.440
Pf.14.443.0_CDS_at	11	1.900	0.795	0.648
Pf.14.444.0_CDS_at	967	**0.206**	**0.261**	**0.376**
Pf.14.446.0_CDS_at	4847	1.702	**1.652**	1.556
Pf.14.447.0_CDS_at	197	1.735	0.718	1.071
Pf.14.448.0_CDS_at	18	0.759	0.535	0.979
Pf.14.449.0_CDS_at	60	**5.517**	1.622	1.103
Pf.14.45.0_CDS_at	255	**0.208**	**0.369**	0.829
Pf.14.450.0_CDS_at	278	1.230	1.618	1.750
Pf.14.451.0_CDS_at	1358	**0.077**	**0.081**	**0.102**
Pf.14.452.0_CDS_at	69	0.801	0.841	1.682
Pf.14.452.0_CDS_x_at	142	0.832	0.836	1.915
Pf.14.453.0_CDS_at	9	0.878	0.569	0.913
Pf.14.455.0_CDS_at	15	**2.710**	**3.414**	**3.314**
Pf.14.456.0_CDS_at	9	0.958	0.863	0.784
Pf.14.457.0_CDS_at	600	1.804	0.631	1.315
Pf.14.458.0_CDS_at	10	1.094	0.884	1.070
Pf.14.459.0_CDS_at	41	1.257	1.396	1.347
Pf.14.46.0_CDS_at	731	**0.396**	1.078	1.429
Pf.14.460.0_CDS_at	17	**2.753**	**2.769**	**2.685**
Pf.14.462.0_CDS_at	14	1.439	1.025	0.646
Pf.14.463.0_CDS_at	2812	**0.375**	**0.354**	0.636
Pf.14.464.0_CDS_at	13	0.755	0.658	1.824
Pf.14.465.0_CDS_at	39	0.939	0.603	0.833
Pf.14.467.0_CDS_at	41	0.679	0.951	1.067
Pf.14.468.0_CDS_at	146	1.427	1.781	**2.562**
Pf.14.469.0_CDS_at	193	**0.276**	0.607	1.556
Pf.14.47.0_CDS_a_at	478	0.828	1.334	**2.228**
Pf.14.470.0_CDS_at	220	0.812	0.635	1.044
Pf.14.471.0_CDS_at	56	**3.172**	**2.520**	1.382
Pf.14.473.0_CDS_at	28	0.599	0.788	**3.150**
Pf.14.474.0_CDS_at	398	**1.585**	**1.844**	**2.322**
Pf.14.475.0_CDS_at	194	0.993	1.012	**2.232**
Pf.14.476.0_CDS_at	406	**0.405**	0.664	**0.460**
Pf.14.478.0_CDS_at	3082	**0.076**	**0.041**	**0.035**
Pf.14.479.0_CDS_at	59	0.870	1.220	**3.157**
Pf.14.479.0_CDS_x_at	13	0.816	0.915	1.660

Pf.14.48.0_CDS_at	658	1.356	1.939	1.602	
Pf.14.480.0_CDS_at	60	1.427	1.007	1.839	
Pf.14.481.0_CDS_at	534	0.922	1.044	**2.068**	
Pf.14.482.0_CDS_at	22	0.976	0.718	0.965	
Pf.14.485.0_CDS_at	8	1.902	0.984	0.607	
Pf.14.486.0_CDS_at	636	**0.452**	0.965	1.799	
Pf.14.487.0_CDS_at	14	1.089	**0.385**	**0.391**	
Pf.14.488.0_CDS_at	459	**0.428**	**0.491**	0.689	
Pf.14.489.0_CDS_at	497	**2.633**	1.684	0.646	
Pf.14.49.0_CDS_at	261	0.607	0.547	0.651	
Pf.14.490.0_CDS_at	17	0.712	0.985	1.699	
Pf.14.491.0_CDS_at	12	1.765	1.191	0.856	
Pf.14.493.0_CDS_at	7	1.075	1.205	1.853	
Pf.14.494.0_CDS_at	1344	0.588	0.726	1.184	
Pf.14.494.0_CDS_x_at	1356	**0.461**	0.722	1.164	
Pf.14.496.0_CDS_at	424	0.900	0.945	1.045	
Pf.14.498.0_CDS_at	6	1.071	1.117	1.143	
Pf.14.499.0_CDS_at	25	1.735	0.606	1.291	
Pf.14.5.0_CDS_at	1916	0.607	0.889	1.321	
Pf.14.50.0_CDS_at	1445	**0.274**	**0.196**	**0.128**	
Pf.14.501.0_CDS_at	9	1.152	0.642	0.692	
Pf.14.502.0_CDS_at	54	**2.802**	0.902	0.719	
Pf.14.503.0_CDS_a_at	306	1.159	0.669	**3.202**	
Pf.14.504.0_CDS_at	35	**0.465**	0.642	**0.236**	
Pf.14.505.0_CDS_at	18	0.988	0.705	0.758	
Pf.14.506.0_CDS_at	9	0.775	1.144	1.043	
Pf.14.507.0_CDS_at	18	1.830	**2.804**	**3.140**	
Pf.14.508.0_CDS_at	175	0.629	1.128	**2.338**	
Pf.14.51.0_CDS_at	137	**0.470**	0.802	1.124	
Pf.14.510.0_CDS_at	12	1.273	0.597	0.910	
Pf.14.511.0_CDS_at	51	**0.345**	0.765	1.140	
Pf.14.512.0_CDS_at	300	**3.477**	**2.917**	**2.915**	
Pf.14.513.0_CDS_at	370	**0.442**	**0.444**	**0.346**	
Pf.14.515.0_CDS_at	214	0.922	0.804	1.919	
Pf.14.517.0_CDS_at	6	1.100	0.987	1.052	
Pf.14.518.0_CDS_at	6	1.118	0.850	0.979	
Pf.14.518.0_CDS_x_at	8	1.862	1.044	1.095	
Pf.14.519.0_CDS_at	18	0.694	1.157	**3.144**	
Pf.14.52.0_CDS_at	63	0.537	0.933	**2.567**	
Pf.14.520.0_CDS_at	49	**3.176**	**2.413**	**2.769**	
Pf.14.521.0_CDS_at	9	1.591	0.754	0.928	
Pf.14.522.0_CDS_at	131	**2.158**	**2.264**	1.862	
Pf.14.523.0_CDS_at	70	0.945	1.024	1.277	
Pf.14.524.0_CDS_at	200	1.434	1.307	1.014	
Pf.14.529.0_CDS_at	4867	**2.450**	1.638	1.683	
Pf.14.53.0_CDS_a_at	15937	1.251	1.090	1.097	
Pf.14.531.0_CDS_at	432	0.668	0.661	**2.197**	
Pf.14.533.0_CDS_at	53	**2.223**	**2.628**	**2.527**	
Pf.14.534.0_CDS_at	407	**2.160**	**2.143**	1.828	
Pf.14.535.0_CDS_at	1298	**0.213**	**0.288**	**0.440**	
Pf.14.536.0_CDS_at	76	1.275	**2.523**	**2.670**	
Pf.14.537.0_CDS_at	426	1.486	1.108	0.944	
Pf.14.538.0_CDS_at	169	**0.170**	**0.404**	1.554	#6
Pf.14.539.0_CDS_at	285	1.637	**3.283**	1.796	
Pf.14.54.0_CDS_at	4217	0.571	0.810	0.977	
Pf.14.540.0_CDS_at	5	1.316	**2.140**	1.329	
Pf.14.541.0_CDS_at	8	**2.946**	**2.227**	0.698	
Pf.14.543.0_CDS_at	74	**0.174**	**0.206**	0.941	
Pf.14.545.0_CDS_at	787	1.046	0.889	1.264	
Pf.14.546.0_CDS_at	888	0.543	0.942	1.197	
Pf.14.547.0_CDS_at	42	0.632	1.181	0.707	
Pf.14.549.0_CDS_at	9	**3.698**	0.972	1.514	
Pf.14.55.0_CDS_a_at	3220	0.619	0.618	1.091	

Pf.14.550.0_CDS_at	17	0.590	0.423	**0.400**
Pf.14.551.0_CDS_at	6	1.754	1.272	0.993
Pf.14.554.0_CDS_at	2573	1.670	1.266	1.283
Pf.14.555.0_CDS_at	22	1.281	**2.815**	1.373
Pf.14.556.0_CDS_at	380	1.091	**2.346**	0.899
Pf.14.557.0_CDS_at	8	0.988	1.119	0.703
Pf.14.558.0_CDS_at	30	0.804	0.741	**3.316**
Pf.14.559.0_CDS_at	35	**2.041**	**0.465**	**5.053**
Pf.14.561.0_CDS_s_at	585	1.023	1.540	1.815
Pf.14.562.0_CDS_at	93	1.534	0.803	**0.408**
Pf.14.563.0_CDS_at	316	**2.040**	**3.129**	**2.957**
Pf.14.565.0_CDS_at	12	0.776	0.690	0.757
Pf.14.566.0_CDS_at	9	1.349	0.724	0.669
Pf.14.567.0_CDS_at	6	1.126	1.039	0.810
Pf.14.568.0_CDS_at	6	1.009	0.787	0.944
Pf.14.569.0_CDS_at	15	1.093	0.509	0.883
Pf.14.57.0_CDS_at	3455	1.108	0.800	1.368
Pf.14.570.0_CDS_at	10	**2.269**	1.430	1.163
Pf.14.576.0_CDS_at	6	1.063	1.096	0.953
Pf.14.576.0_CDS_x_at	6	1.061	0.862	0.956
Pf.14.577.0_CDS_at	6	0.934	1.133	1.204
Pf.14.577.0_CDS_x_at	7	0.930	1.019	0.985
Pf.14.578.0_CDS_at	6	1.059	0.941	0.896
Pf.14.579.0_CDS_at	6	1.125	1.022	0.837
Pf.14.58.0_CDS_at	13	0.926	0.664	0.609
Pf.14.580.0_CDS_at	6	1.399	1.426	1.244
Pf.14.580.0_CDS_s_at	6	0.835	0.786	0.764
Pf.14.580.0_CDS_x_at	7	1.208	1.326	1.317
Pf.14.581.0_CDS_at	7	1.022	0.943	0.897
Pf.14.582.0_CDS_at	6	1.284	1.167	1.090
Pf.14.583.0_CDS_at	5	1.106	0.951	1.038
Pf.14.584.0_CDS_at	5	1.102	0.993	1.286
Pf.14.586.0_CDS_at	6	1.185	0.970	0.984
Pf.14.587.0_CDS_at	7	0.852	0.890	1.071
Pf.14.588.0_CDS_at	5	1.055	1.116	1.052
Pf.14.589.0_CDS_at	6	1.066	1.107	1.081
Pf.14.59.0_CDS_at	4398	1.007	0.929	0.507
Pf.14.590.0_CDS_at	5	1.195	0.962	1.103
Pf.14.592.0_CDS_x_at	6	1.481	2.045	1.288
Pf.14.593.0_CDS_at	118	**4.462**	1.804	1.701
Pf.14.594.0_CDS_at	284	**9.608**	**4.423**	**3.339**
Pf.14.595.0_CDS_at	7	1.142	0.990	1.136
Pf.14.596.0_CDS_at	16	0.756	1.303	1.893
Pf.14.598.0_CDS_at	67	0.534	0.988	0.592
Pf.14.599.0_CDS_at	396	**0.284**	**0.217**	**0.315**
Pf.14.60.1_CDS_a_at	54	0.641	0.657	1.044
Pf.14.600.0_CDS_at	148	0.630	1.198	**2.258**
Pf.14.601.0_CDS_a_at	20	0.581	**0.315**	0.529
Pf.14.602.0_CDS_at	8	0.868	0.865	0.783
Pf.14.603.0_CDS_at	4066	1.591	1.423	1.507
Pf.14.605.0_CDS_at	45	**3.068**	**2.766**	**3.033**
Pf.14.606.0_CDS_at	299	1.161	1.645	1.455
Pf.14.608.0_CDS_at	4170	**0.111**	**0.107**	**0.072**
Pf.14.61.0_CDS_at	217	0.643	1.008	0.941
Pf.14.610.0_CDS_at	984	1.170	0.906	1.680
Pf.14.614.0_CDS_at	50	1.212	0.816	1.861
Pf.14.615.0_CDS_at	155	1.467	**2.045**	**4.128**
Pf.14.616.0_CDS_at	7	0.848	0.817	1.708
Pf.14.618.0_CDS_at	105	**2.016**	**2.059**	1.949
Pf.14.62.0_CDS_at	1741	1.729	1.115	1.464
Pf.14.620.0_CDS_a_at	627	0.756	0.857	0.894
Pf.14.621.0_CDS_at	65	0.701	0.831	**0.360**

Pf.14.625.0_CDS_a_at	100	1.011	1.311	1.784	
Pf.14.626.0_CDS_at	8	0.833	0.810	0.756	
Pf.14.628.0_CDS_at	10	1.575	1.099	0.979	
Pf.14.629.0_CDS_at	35	0.920	1.343	1.489	
Pf.14.63.0_CDS_at	833	0.824	1.302	1.540	
Pf.14.630.0_CDS_at	300	1.547	**2.152**	**2.447**	
Pf.14.631.0_CDS_at	16	1.288	0.649	1.191	
Pf.14.632.0_CDS_at	6	1.127	1.536	1.568	
Pf.14.633.0_CDS_at	96	0.546	1.345	1.889	
Pf.14.634.0_CDS_at	27	0.941	0.576	1.229	
Pf.14.635.0_CDS_at	162	0.685	1.064	**3.473**	
Pf.14.636.0_CDS_at	7	0.839	0.689	0.757	
Pf.14.637.0_CDS_at	200	0.609	1.292	1.857	
Pf.14.637.0_CDS_x_at	220	0.530	1.131	1.995	
Pf.14.638.0_CDS_at	6	0.896	0.943	1.226	
Pf.14.639.0_CDS_at	88	**5.364**	**4.996**	**2.540**	
Pf.14.640.0_CDS_at	272	1.712	**2.912**	**2.730**	
Pf.14.641.0_CDS_at	795	1.011	1.672	1.660	
Pf.14.643.0_CDS_at	106	0.533	1.055	0.792	
Pf.14.645.0_CDS_at	24	0.818	1.431	**2.560**	
Pf.14.646.0_CDS_at	373	1.136	1.276	**2.706**	
Pf.14.647.0_CDS_at	563	0.737	1.761	**2.743**	
Pf.14.648.0_CDS_at	141	**3.197**	**3.675**	**2.576**	
Pf.14.650.0_CDS_at	253	**2.224**	1.969	**2.036**	
Pf.14.651.0_CDS_at	8	1.120	0.871	1.002	
Pf.14.652.0_CDS_at	5	3.128	1.779	1.168	
Pf.14.653.0_CDS_at	130	1.700	0.800	**2.096**	
Pf.14.654.0_CDS_at	410	0.551	1.250	1.610	
Pf.14.655.0_CDS_at	29	**0.348**	0.561	**0.357**	
Pf.14.656.0_CDS_at	28	**0.499**	**0.494**	0.679	
Pf.14.657.0_CDS_at	10	1.225	0.709	0.705	
Pf.14.659.0_CDS_at	8	1.012	0.858	1.519	
Pf.14.66.0_CDS_at	665	**0.462**	0.703	0.838	
Pf.14.661.0_CDS_at	224	0.736	0.977	**2.415**	
Pf.14.662.0_CDS_at	500	0.860	1.084	**2.271**	
Pf.14.663.0_CDS_at	26	0.668	0.756	1.130	
Pf.14.664.0_CDS_at	555	1.619	**2.164**	1.342	
Pf.14.665.0_CDS_at	84	0.777	**0.389**	0.746	
Pf.14.666.0_CDS_at	54	0.611	0.594	0.761	
Pf.14.667.0_CDS_at	11	0.713	**0.468**	0.685	
Pf.14.667.0_CDS_x_at	58	0.597	**0.388**	0.697	
Pf.14.668.0_CDS_at	254	0.543	0.533	1.427	
Pf.14.669.0_CDS_at	1323	**0.399**	0.529	**0.439**	
Pf.14.67.0_CDS_at	564	**0.267**	0.734	**0.350**	
Pf.14.670.0_CDS_at	893	0.897	1.299	**0.229**	
Pf.14.671.0_CDS_at	18	0.615	**0.359**	**0.368**	
Pf.14.674.0_CDS_at	529	0.679	0.539	1.379	
Pf.14.675.0_CDS_at	263	0.507	1.333	**2.036**	
Pf.14.676.0_CDS_at	6	0.926	0.998	1.105	
Pf.14.678.0_CDS_at	15	1.222	1.072	**2.028**	
Pf.14.68.0_CDS_at	248	**0.104**	**0.247**	0.734	#7
Pf.14.68.0_CDS_x_at	453	**0.123**	**0.245**	0.780	
Pf.14.680.0_CDS_at	8	1.052	1.046	0.927	
Pf.14.681.0_CDS_at	182	**2.249**	**2.314**	**2.084**	
Pf.14.681.0_CDS_x_at	239	1.873	**2.112**	1.918	
Pf.14.682.0_CDS_at	9	0.976	0.768	0.933	
Pf.14.683.0_CDS_at	3269	0.860	0.639	1.037	
Pf.14.684.0_CDS_at	95	**0.496**	0.643	**0.479**	
Pf.14.685.0_CDS_a_at	37	1.323	1.035	1.006	
Pf.14.687.0_CDS_at	12	0.553	0.528	1.557	
Pf.14.688.0_CDS_at	442	**0.342**	**0.444**	1.987	
Pf.14.689.0_CDS_at	66	1.319	1.793	**2.558**	
Pf.14.69.0_CDS_a_at	26	0.634	**0.430**	0.900	

Pf.14.690.0_CDS_at	17	1.104	0.717	0.806
Pf.14.692.0_CDS_at	118	0.872	1.017	1.454
Pf.14.693.0_CDS_at	6	0.952	1.040	0.990
Pf.14.694.0_CDS_at	63	1.066	**2.071**	**2.602**
Pf.14.695.0_CDS_at	246	1.075	1.114	1.445
Pf.14.696.0_CDS_at	21	0.813	**0.357**	0.850
Pf.14.698.0_CDS_at	620	1.741	**2.120**	**2.428**
Pf.14.699.0_CDS_at	9	0.770	0.625	0.734
Pf.14.7.0_CDS_a_at	7	0.823	0.744	0.729
Pf.14.70.0_CDS_at	593	**0.141**	**0.210**	0.519
Pf.14.701.0_CDS_at	6	1.056	1.084	1.030
Pf.14.702.0_CDS_at	35	0.813	0.625	**3.524**
Pf.14.703.0_CDS_at	14	1.288	1.909	**3.430**
Pf.14.704.0_CDS_at	16	1.278	0.820	0.892
Pf.14.705.0_CDS_a_at	58	**2.283**	1.590	0.633
Pf.14.705.1_a_at	19	1.385	0.677	0.609
Pf.14.706.0_CDS_at	490	0.762	**0.424**	**2.232**
Pf.14.707.0_CDS_at	126	0.916	0.539	1.570
Pf.14.708.0_CDS_at	149	1.799	1.089	**2.135**
Pf.14.71.0_CDS_at	7482	0.948	0.792	1.007
Pf.14.710.0_CDS_at	11	1.637	0.874	0.973
Pf.14.711.0_CDS_at	961	1.350	1.335	1.662
Pf.14.712.0_CDS_at	1450	1.206	0.849	**2.160**
Pf.14.715.0_CDS_at	757	0.733	0.666	0.505
Pf.14.716.0_CDS_at	532	0.529	**0.385**	0.632
Pf.14.717.0_CDS_at	20	1.588	0.570	0.730
Pf.14.718.0_CDS_at	19	1.988	0.666	1.425
Pf.14.719.0_CDS_at	709	0.954	1.529	**2.583**
Pf.14.72.0_CDS_at	335	1.262	**2.219**	**2.035**
Pf.14.720.0_CDS_at	12	0.821	1.117	1.048
Pf.14.723.0_CDS_at	2163	0.924	0.591	1.056
Pf.14.726.0_CDS_at	8	1.237	1.528	1.159
Pf.14.727.0_CDS_at	176	**0.495**	1.685	**2.347**
Pf.14.73.0_CDS_at	26	1.111	1.016	1.514
Pf.14.731.0_CDS_at	272	1.284	1.474	1.655
Pf.14.732.0_CDS_at	390	1.790	1.827	**2.265**
Pf.14.734.0_CDS_at	26	1.783	1.160	0.997
Pf.14.735.0_CDS_at	6	1.045	1.412	1.216
Pf.14.736.1_at	8	1.066	0.806	0.842
Pf.14.739.0_CDS_at	8	1.489	2.025	1.764
Pf.14.74.0_CDS_at	92	1.218	1.583	0.633
Pf.14.740.0_CDS_at	158	1.636	**3.162**	**2.590**
Pf.14.741.0_CDS_at	6	1.312	1.027	1.047
Pf.14.742.0_CDS_at	338	0.523	**0.390**	**0.337**
Pf.14.743.0_CDS_at	42	0.762	1.707	**2.384**
Pf.14.745.0_CDS_at	200	**0.315**	**0.292**	1.040
Pf.14.747.0_CDS_at	62	0.749	0.979	1.203
Pf.14.748.0_CDS_at	41	1.868	**3.719**	**4.245**
Pf.14.749.0_CDS_at	27	1.005	0.523	1.970
Pf.14.75.0_CDS_a_at	7	1.091	0.897	0.875
Pf.14.752.0_CDS_a_at	2499	0.632	0.527	1.279
Pf.14.752.0_CDS_at	393	**0.391**	**0.490**	1.702
Pf.14.752.1_at	1056	**0.470**	0.508	1.315
Pf.14.753.0_CDS_at	11	0.691	0.657	0.823
Pf.14.754.0_CDS_at	61	**0.160**	**0.179**	**0.141**
Pf.14.756.0_CDS_at	71	1.669	**2.455**	1.946
Pf.14.757.0_CDS_at	142	0.972	0.707	1.909
Pf.14.76.0_CDS_at	590	1.413	1.572	1.259
Pf.14.760.0_CDS_at	9	0.874	1.117	0.887
Pf.14.762.0_CDS_at	2837	0.732	**0.352**	1.482
Pf.14.763.0_CDS_at	7	0.911	0.960	0.922
Pf.14.764.0_CDS_at	13	1.570	**2.103**	**2.215**

Pf.14.769.0_at	56	1.647	1.913	**2.046**
Pf.14.77.0_CDS_at	808	1.934	0.847	1.906
Pf.14.773.0_at	371	**0.211**	**0.181**	0.507
Pf.14.776.0_at	1600	1.603	1.034	1.671
Pf.14.78.0_CDS_at	629	1.908	1.734	1.488
Pf.14.782.0_at	12	**2.299**	0.575	0.744
Pf.14.787.0_at	33	1.211	1.456	**2.261**
Pf.14.788.0_at	204	0.520	1.041	1.503
Pf.14.79.0_CDS_at	301	1.981	**2.949**	**2.244**
Pf.14.8.0_CDS_at	1564	1.344	0.915	1.473
Pf.14.81.0_CDS_at	296	**2.858**	**2.743**	**2.413**
Pf.14.82.0_CDS_at	26	0.548	0.889	**0.452**
Pf.14.835.0_at	81	1.687	0.694	1.128
Pf.14.84.0_CDS_at	125	1.251	**9.070**	**2.755**
Pf.14.846.0_at	139	1.863	1.571	1.385
Pf.14.85.0_CDS_a_at	2956	1.099	1.032	1.305
Pf.14.86.0_CDS_at	310	1.537	1.880	0.925
Pf.14.881.0_at	13	0.522	0.649	1.095
Pf.14.899.0_at	389	0.773	0.538	0.984
Pf.14.9.0_CDS_at	1219	1.376	1.126	1.621
Pf.14.90.0_CDS_at	1602	0.622	0.601	**0.366**
Pf.14.93.0_CDS_at	4897	**0.290**	**0.399**	0.842
Pf.14.930.0_at	202	**0.292**	**0.426**	1.051
Pf.14.94.0_CDS_at	923	0.693	1.317	1.395
Pf.14.95.0_CDS_at	738	1.858	**2.391**	**2.688**
Pf.14.96.0_CDS_at	173	1.572	1.994	1.971
Pf.14.97.0_CDS_a_at	1162	1.792	1.751	**2.327**
Pf.14.98.0_CDS_at	1066	1.793	1.471	1.020
Pf.2.1.0_CDS_a_at	12839	**0.112**	**0.381**	**0.054**
Pf.2.10.0_CDS_a_at	1600	**0.053**	**0.100**	**0.067**
Pf.2.100.0_CDS_at	140	0.545	0.546	1.264
Pf.2.101.0_CDS_at	816	1.375	0.937	0.648
Pf.2.103.0_CDS_x_at	6	1.213	0.986	1.022
Pf.2.104.0_CDS_at	5	1.014	0.892	0.851
Pf.2.105.0_CDS_at	6	0.890	0.954	0.935
Pf.2.106.0_CDS_at	5	1.135	1.008	1.097
Pf.2.108.0_CDS_at	6732	1.488	0.767	0.851
Pf.2.109.0_CDS_at	168	0.719	**0.260**	**0.442**
Pf.2.11.0_CDS_a_at	13535	0.889	0.950	1.198
Pf.2.110.0_CDS_at	12	1.000	0.952	1.056
Pf.2.111.0_CDS_at	205	**2.904**	0.781	**0.316**
Pf.2.111.0_CDS_x_at	192	**3.175**	0.686	**0.365**
Pf.2.112.0_CDS_at	22	0.594	0.862	0.721
Pf.2.113.0_CDS_at	145	**0.344**	0.813	1.085
Pf.2.114.0_CDS_at	70	0.817	1.082	1.413
Pf.2.115.0_CDS_at	111	0.902	0.618	0.785
Pf.2.116.0_CDS_at	121	**3.217**	1.674	**3.392**
Pf.2.118.0_CDS_at	1259	0.731	0.708	1.439
Pf.2.12.0_CDS_at	205	1.066	1.424	1.879
Pf.2.120.0_CDS_at	73	1.871	**2.497**	**2.643**
Pf.2.121.0_CDS_at	220	0.605	0.535	1.094
Pf.2.124.0_CDS_at	7	0.839	0.905	0.951
Pf.2.127.0_CDS_a_at	665	**0.209**	**0.346**	**0.352**
Pf.2.129.0_CDS_at	1549	**2.166**	**2.121**	1.598
Pf.2.13.0_CDS_at	320	**0.091**	**0.069**	**0.072**
Pf.2.131.0_CDS_at	244	1.532	0.599	**0.207**
Pf.2.133.0_CDS_at	188	1.021	1.151	1.423
Pf.2.134.0_CDS_at	74	0.579	**0.475**	1.539
Pf.2.135.0_CDS_at	3351	**0.408**	**0.327**	0.637
Pf.2.136.0_CDS_at	217	0.894	1.390	**3.774**
Pf.2.137.0_CDS_at	155	1.479	1.329	**2.365**
Pf.2.138.0_CDS_at	139	**3.052**	1.211	1.511

Pf.2.14.0_CDS_a_at	1346	0.863	0.863	1.590
Pf.2.140.0_CDS_at	253	0.853	1.084	1.211
Pf.2.140.1_a_at	3565	0.765	0.955	0.999
Pf.2.141.0_CDS_at	116	1.590	**2.305**	**4.353**
Pf.2.143.0_CDS_at	37	**4.916**	**3.358**	**3.578**
Pf.2.144.0_CDS_at	7	1.324	0.773	0.918
Pf.2.145.0_CDS_at	9	0.743	0.676	1.588
Pf.2.146.0_CDS_at	13	1.044	0.652	0.509
Pf.2.148.0_CDS_s_at	125	**2.177**	0.796	**2.334**
Pf.2.151.0_CDS_s_at	8	0.885	0.901	0.663
Pf.2.152.0_CDS_at	6	0.941	0.958	0.899
Pf.2.153.0_CDS_at	5	1.409	1.194	1.279
Pf.2.154.0_CDS_at	6	0.924	0.859	0.910
Pf.2.155.0_CDS_at	15	**2.071**	1.183	0.986
Pf.2.156.0_CDS_s_at	24	1.543	1.085	1.465
Pf.2.156.0_CDS_x_at	17	1.479	1.182	1.217
Pf.2.157.0_CDS_s_at	114	**2.341**	0.624	0.573
Pf.2.158.0_CDS_at	8	1.042	0.871	0.981
Pf.2.159.0_CDS_at	7	1.036	0.689	0.755
Pf.2.16.0_CDS_at	220	**0.171**	**0.153**	**0.103**
Pf.2.160.0_CDS_at	5	1.045	1.140	0.974
Pf.2.161.0_CDS_at	8	1.193	0.972	0.840
Pf.2.162.0_CDS_s_at	7	0.961	0.926	0.760
Pf.2.163.0_CDS_at	8	1.117	1.191	1.196
Pf.2.164.0_CDS_at	7	0.897	0.966	0.929
Pf.2.165.0_CDS_at	7	1.083	1.061	0.955
Pf.2.166.0_CDS_at	6	0.823	0.812	0.877
Pf.2.168.0_CDS_at	6	1.166	1.123	0.996
Pf.2.17.0_CDS_at	332	0.781	1.187	1.297
Pf.2.170.0_CDS_at	8	1.057	1.105	1.024
Pf.2.171.0_CDS_at	6	0.998	0.923	0.975
Pf.2.173.0_CDS_at	7	0.875	0.945	0.865
Pf.2.174.0_CDS_s_at	5	0.905	0.879	0.914
Pf.2.175.0_CDS_at	280	**5.869**	**2.276**	1.379
Pf.2.177.0_CDS_at	94	0.962	0.784	0.648
Pf.2.178.0_CDS_at	142	**0.356**	0.610	0.620
Pf.2.18.0_CDS_a_at	215	**0.464**	0.526	**0.465**
Pf.2.180.0_CDS_at	96	1.577	0.821	1.361
Pf.2.181.0_CDS_at	58	0.962	0.932	0.884
Pf.2.182.0_CDS_at	1284	1.309	1.247	1.262
Pf.2.184.0_CDS_at	221	0.887	0.901	1.640
Pf.2.185.0_CDS_at	8	0.982	0.703	0.629
Pf.2.186.0_CDS_at	181	**0.271**	1.288	**0.157**
Pf.2.187.0_CDS_at	790	1.962	1.917	1.808
Pf.2.188.0_CDS_at	172	1.639	0.567	**0.387**
Pf.2.189.0_CDS_at	267	0.683	1.412	1.142
Pf.2.19.0_CDS_a_at	19	1.578	0.609	0.770
Pf.2.190.0_CDS_at	333	**0.318**	**0.150**	**0.166**
Pf.2.192.0_CDS_at	1118	**0.240**	**0.193**	**0.413**
Pf.2.194.0_CDS_at	11	1.087	0.641	0.807
Pf.2.195.0_CDS_a_at	21	0.705	0.609	0.529
Pf.2.196.0_CDS_at	176	**2.329**	1.435	1.661
Pf.2.197.0_CDS_at	70	1.103	1.313	1.206
Pf.2.199.0_CDS_at	931	**2.078**	1.905	1.157
Pf.2.2.0_CDS_at	6	1.073	0.827	0.884
Pf.2.20.0_CDS_at	235	**0.122**	**0.272**	**0.124**
Pf.2.200.0_CDS_at	241	**3.334**	**4.533**	1.500
Pf.2.201.0_CDS_at	101	**2.878**	**2.812**	**3.322**
Pf.2.202.0_CDS_at	25	0.698	**0.336**	**0.470**
Pf.2.203.0_CDS_a_at	70	0.731	0.918	1.139
Pf.2.203.0_CDS_at	128	0.742	1.063	1.473
Pf.2.203.0_CDS_x_at	241	0.562	0.830	1.346

Pf.2.204.0_CDS_at	302	1.560	1.304	0.594
Pf.2.206.0_CDS_at	59	**0.234**	**0.189**	**0.413**
Pf.2.207.0_CDS_at	152	0.702	0.746	1.744
Pf.2.207.0_CDS_x_at	131	0.916	1.110	**2.239**
Pf.2.209.0_CDS_at	898	1.132	1.434	1.279
Pf.2.21.0_CDS_at	830	1.581	1.169	1.336
Pf.2.21.0_UTR_at	1210	1.875	1.567	1.482
Pf.2.21.0_UTR_x_at	471	**2.171**	1.472	1.947
Pf.2.210.0_CDS_at	9	1.251	0.821	**4.479**
Pf.2.211.0_CDS_at	689	**0.416**	**0.406**	**0.417**
Pf.2.212.0_CDS_at	417	1.625	**2.129**	**2.252**
Pf.2.214.0_CDS_at	204	**0.295**	1.203	0.845
Pf.2.215.0_CDS_at	636	**4.748**	**4.195**	**3.369**
Pf.2.218.0_CDS_at	7	1.561	0.895	0.958
Pf.2.22.0_CDS_at	1028	1.200	1.367	1.690
Pf.2.221.0_CDS_at	6	1.080	0.882	1.048
Pf.2.221.0_CDS_x_at	5	1.066	1.047	0.963
Pf.2.222.0_CDS_s_at	119	1.973	1.122	0.516
Pf.2.224.0_CDS_x_at	16	**3.986**	1.479	1.119
Pf.2.225.0_at	951	**0.460**	**0.345**	**0.438**
Pf.2.228.0_at	19	0.961	0.712	**7.549**
Pf.2.23.0_CDS_at	9	0.781	0.646	0.855
Pf.2.234.0_at	11	1.332	0.679	1.033
Pf.2.24.0_CDS_a_at	468	0.554	0.730	0.755
Pf.2.24.0_CDS_at	65	0.618	0.921	0.931
Pf.2.24.1_at	38	0.772	0.763	1.273
Pf.2.240.0_at	64	0.851	0.876	0.540
Pf.2.243.0_at	394	1.039	1.027	1.259
Pf.2.246.0_at	25	1.081	0.700	0.752
Pf.2.25.0_CDS_at	28	0.548	0.612	1.115
Pf.2.258.0_at	452	0.794	0.676	1.117
Pf.2.259.0_at	783	1.366	0.592	1.540
Pf.2.27.0_CDS_at	393	**0.210**	**0.088**	**0.058**
Pf.2.29.0_CDS_at	740	**0.428**	1.076	0.661
Pf.2.3.0_CDS_at	15155	**0.043**	**0.030**	**0.030**
Pf.2.31.0_CDS_at	12	0.677	0.932	0.692
Pf.2.32.0_CDS_a_at	41	1.460	1.995	1.327
Pf.2.34.0_CDS_at	1349	1.398	0.715	1.824
Pf.2.35.0_CDS_at	1059	0.778	0.852	1.409
Pf.2.36.0_CDS_at	1213	0.854	**0.353**	**0.445**
Pf.2.37.0_CDS_at	6242	**0.032**	**0.216**	**0.022**
Pf.2.39.0_CDS_at	1791	**3.479**	**2.343**	1.711
Pf.2.4.0_CDS_a_at	2017	1.582	1.283	1.400
Pf.2.40.0_CDS_s_at	1644	**2.004**	1.660	1.152
Pf.2.41.0_CDS_a_at	3959	0.837	0.820	1.019
Pf.2.42.0_CDS_at	5772	0.954	1.231	1.087
Pf.2.43.0_CDS_at	26	1.834	**0.470**	0.592
Pf.2.44.0_CDS_at	3404	**0.131**	**0.117**	**0.040**
Pf.2.45.0_CDS_at	65	**0.110**	**0.097**	**0.085**
Pf.2.47.0_CDS_at	1053	**0.368**	**0.262**	**0.269**
Pf.2.48.0_CDS_at	1194	**0.045**	**0.102**	**0.013**
Pf.2.49.0_CDS_at	19	**0.467**	0.582	**0.353**
Pf.2.5.0_CDS_at	1066	1.302	1.327	1.500
Pf.2.50.0_CDS_at	447	0.884	0.889	1.340
Pf.2.51.0_CDS_at	652	**0.126**	**0.198**	**0.084**
Pf.2.53.0_CDS_at	46	1.648	1.045	0.666
Pf.2.54.0_CDS_a_at	208	0.686	**0.201**	0.840
Pf.2.55.0_CDS_at	16	0.979	1.057	1.790
Pf.2.56.0_CDS_at	14	**0.491**	0.756	**0.487**
Pf.2.6.0_CDS_at	496	**0.332**	1.247	**0.250**
Pf.2.60.0_CDS_at	307	1.515	1.091	1.886
Pf.2.61.0_CDS_at	46	0.855	0.940	**2.842**

Pf.2.63.0_CDS_at	125	1.116	0.558	1.029
Pf.2.64.0_CDS_at	464	1.579	1.637	1.777
Pf.2.65.0_CDS_at	554	1.378	1.574	1.439
Pf.2.65.0_CDS_x_at	656	**2.040**	1.769	1.454
Pf.2.66.0_CDS_at	628	**0.354**	0.561	1.205
Pf.2.67.0_CDS_at	8	0.797	0.692	0.690
Pf.2.68.0_CDS_at	24	0.765	1.534	**3.927**
Pf.2.7.0_CDS_at	676	**0.475**	0.898	0.539
Pf.2.70.0_CDS_at	99	**4.962**	1.298	1.268
Pf.2.71.0_CDS_at	36	0.510	0.631	1.291
Pf.2.72.0_CDS_at	149	0.553	1.031	1.267
Pf.2.73.0_CDS_at	74	1.381	1.363	1.622
Pf.2.74.0_CDS_at	616	1.851	1.995	1.930
Pf.2.75.0_CDS_at	54	**0.242**	**0.434**	0.504
Pf.2.76.0_CDS_at	57	**0.253**	0.507	0.882
Pf.2.77.0_CDS_at	740	**0.383**	**0.424**	1.251
Pf.2.78.0_CDS_at	1933	0.895	0.958	1.672
Pf.2.79.0_CDS_at	75	0.794	1.689	1.732
Pf.2.8.0_CDS_s_at	5	1.046	0.985	1.150
Pf.2.80.0_CDS_at	1046	0.716	0.719	**0.234**
Pf.2.81.0_CDS_at	645	0.503	1.197	**2.025**
Pf.2.83.0_CDS_at	40	1.449	**2.579**	**3.727**
Pf.2.84.0_CDS_at	419	**2.334**	**2.163**	1.731
Pf.2.86.0_CDS_at	433	0.811	**2.382**	**0.357**
Pf.2.87.0_CDS_at	757	**2.329**	**2.096**	1.779
Pf.2.88.0_CDS_s_at	7	1.567	1.113	0.707
Pf.2.89.0_CDS_at	6	0.904	0.897	1.007
Pf.2.9.0_CDS_a_at	4903	0.947	1.026	1.362
Pf.2.9.0_CDS_x_at	2621	1.097	1.171	1.738
Pf.2.9.1_CDS_a_at	5116	0.914	1.026	1.314
Pf.2.90.0_CDS_at	1017	1.300	**0.496**	1.052
Pf.2.91.0_CDS_a_at	15	0.540	0.585	0.649
Pf.2.94.0_CDS_at	1013	**0.437**	**0.462**	0.964
Pf.2.95.0_CDS_at	542	0.662	0.926	**2.055**
Pf.2.96.0_CDS_at	55	**0.140**	**0.136**	**0.102**
Pf.2.97.0_CDS_at	48	1.891	**0.477**	**0.288**
Pf.2.98.0_CDS_at	56	0.611	0.613	1.533
Pf.2.99.0_CDS_at	70	0.597	**0.360**	1.508
Pf.3.1.0_CDS_a_at	378	**0.019**	**0.025**	**0.025**
Pf.3.1.0_CDS_s_at	253	**0.053**	**0.068**	**0.052**
Pf.3.1.1_CDS_a_at	437	**0.018**	**0.020**	**0.022**
Pf.3.100.0_CDS_at	1183	0.839	**2.028**	**2.493**
Pf.3.103.0_CDS_at	3528	**0.301**	**0.267**	**0.395**
Pf.3.104.0_CDS_at	3769	1.628	1.288	1.439
Pf.3.105.0_CDS_at	210	**4.191**	**3.169**	**2.530**
Pf.3.106.0_CDS_at	935	**0.204**	0.511	**0.433**
Pf.3.109.0_CDS_at	35	1.235	0.717	**2.814**
Pf.3.11.0_CDS_at	176	**0.460**	0.628	0.544
Pf.3.110.0_CDS_at	245	1.209	1.829	1.479
Pf.3.111.0_CDS_at	197	**2.930**	**3.138**	0.839
Pf.3.112.0_CDS_at	79	1.248	1.114	1.288
Pf.3.113.0_CDS_at	974	1.278	1.777	1.795
Pf.3.114.0_CDS_at	927	1.892	1.958	**2.154**
Pf.3.117.0_CDS_at	73	1.190	1.578	**2.045**
Pf.3.119.0_CDS_at	593	0.861	0.740	0.708
Pf.3.12.0_CDS_at	742	0.962	0.579	**0.410**
Pf.3.120.0_CDS_at	151	**0.283**	**0.400**	1.281
Pf.3.121.0_CDS_at	246	**0.205**	**0.247**	0.926
Pf.3.123.0_CDS_at	983	**0.320**	**0.443**	0.510
Pf.3.124.0_CDS_at	1831	**0.358**	0.702	1.475
Pf.3.124.0_CDS_x_at	1863	**0.372**	0.727	1.296
Pf.3.125.0_CDS_x_at	4	1.194	1.192	1.217

Pf.3.126.0_CDS_at	6	1.145	0.980	0.884
Pf.3.127.0_CDS_at	32	1.664	0.689	**0.448**
Pf.3.127.0_CDS_s_at	9	1.443	0.807	0.609
Pf.3.129.0_CDS_at	11	1.007	0.614	0.696
Pf.3.13.0_CDS_at	73	0.520	**0.357**	**0.393**
Pf.3.130.0_CDS_at	110	**3.611**	1.989	0.998
Pf.3.131.0_CDS_at	199	**0.203**	**0.148**	**0.070**
Pf.3.132.0_CDS_at	147	1.368	1.299	1.309
Pf.3.132.0_CDS_x_at	109	1.346	1.206	1.414
Pf.3.133.0_CDS_at	102	1.155	0.774	0.768
Pf.3.134.0_CDS_at	714	**0.305**	**0.416**	**0.334**
Pf.3.135.0_CDS_at	64	1.984	**0.399**	**0.542**
Pf.3.136.0_CDS_a_at	244	**2.416**	**2.102**	**2.143**
Pf.3.137.0_CDS_at	183	0.689	0.570	1.147
Pf.3.138.0_CDS_at	104	**2.173**	1.061	0.987
Pf.3.139.0_CDS_at	24	1.252	0.932	**2.236**
Pf.3.14.0_CDS_at	2416	1.205	1.156	1.248
Pf.3.140.0_CDS_at	9	0.815	0.853	0.932
Pf.3.141.0_CDS_at	115	0.958	0.519	0.536
Pf.3.142.0_CDS_at	647	**0.299**	**0.302**	0.986
Pf.3.143.0_CDS_x_at	483	**2.477**	1.055	**0.334**
Pf.3.144.0_CDS_at	33	1.527	**2.316**	1.241
Pf.3.147.0_CDS_at	1739	**0.409**	0.615	**0.395**
Pf.3.148.0_CDS_at	137	1.816	**2.496**	**3.414**
Pf.3.151.0_CDS_at	6	0.935	0.860	1.200
Pf.3.152.0_CDS_at	141	**4.122**	1.685	0.859
Pf.3.154.0_CDS_at	89	**3.809**	**3.011**	**0.323**
Pf.3.155.0_CDS_at	10	0.761	0.901	1.246
Pf.3.156.0_CDS_at	8	1.876	0.832	0.909
Pf.3.157.0_CDS_at	10	0.707	0.624	0.741
Pf.3.158.0_CDS_at	9	1.118	0.986	0.980
Pf.3.159.0_CDS_at	308	**2.617**	**2.849**	**5.913**
Pf.3.16.0_CDS_at	911	1.589	1.051	1.445
Pf.3.160.0_CDS_at	24	1.156	0.742	0.920
Pf.3.162.0_CDS_at	131	**0.313**	0.985	**2.270**
Pf.3.163.0_CDS_at	44	1.759	**0.301**	**0.226**
Pf.3.164.0_CDS_at	21	**5.118**	1.290	**0.402**
Pf.3.165.0_CDS_at	301	0.557	0.926	1.560
Pf.3.166.0_CDS_at	15	1.534	**2.512**	**3.027**
Pf.3.167.0_CDS_at	353	0.640	0.692	1.310
Pf.3.168.0_CDS_at	10	0.891	0.654	0.724
Pf.3.169.0_CDS_a_at	2613	0.578	0.879	1.305
Pf.3.170.0_CDS_at	8	0.954	0.613	0.596
Pf.3.171.0_CDS_at	464	0.921	1.531	1.903
Pf.3.174.0_CDS_at	41	0.752	0.525	0.703
Pf.3.178.0_CDS_at	12	1.249	1.367	0.786
Pf.3.179.0_CDS_at	5	1.237	1.080	1.024
Pf.3.18.0_CDS_at	855	1.434	0.990	1.570
Pf.3.180.0_CDS_a_at	6	1.045	1.010	0.972
Pf.3.182.0_CDS_at	8	1.090	0.968	0.974
Pf.3.183.0_CDS_at	6	1.004	0.895	0.857
Pf.3.183.0_CDS_x_at	5	1.048	1.127	0.833
Pf.3.184.0_CDS_at	6	0.903	0.969	1.121
Pf.3.185.0_CDS_at	6	1.215	0.905	1.206
Pf.3.186.0_CDS_at	103	**2.478**	1.467	**0.396**
Pf.3.187.0_CDS_at	13	1.236	0.598	0.687
Pf.3.188.0_CDS_at	124	0.961	0.711	1.886
Pf.3.19.0_CDS_at	350	0.960	0.557	0.721
Pf.3.191.0_CDS_at	1065	1.792	**2.056**	**2.448**
Pf.3.193.0_CDS_at	6	1.065	1.193	1.498
Pf.3.194.0_CDS_at	157	0.502	0.803	1.130
Pf.3.195.0_CDS_at	19	0.995	1.997	**2.473**

Pf.3.196.0_CDS_at	20	0.915	1.305	**2.674**
Pf.3.197.0_CDS_at	38	1.686	**0.432**	**0.427**
Pf.3.198.0_CDS_at	560	**2.370**	**2.775**	**2.309**
Pf.3.2.0_CDS_s_at	5871	**0.301**	0.654	**0.165**
Pf.3.200.0_CDS_at	32	**2.235**	1.072	**0.378**
Pf.3.202.0_CDS_at	65	**2.397**	0.863	1.090
Pf.3.203.0_CDS_at	297	**0.461**	1.129	1.450
Pf.3.204.0_CDS_at	184	1.088	**0.122**	**0.492**
Pf.3.205.0_CDS_at	279	1.032	0.772	**0.173**
Pf.3.206.0_CDS_at	630	0.793	1.398	**2.050**
Pf.3.207.0_CDS_at	1527	**0.473**	1.024	1.750
Pf.3.208.0_CDS_at	61	1.037	0.835	1.231
Pf.3.209.0_CDS_at	37	0.840	1.024	1.042
Pf.3.21.0_CDS_at	3594	0.823	0.713	0.857
Pf.3.211.0_CDS_at	36	0.890	1.310	1.175
Pf.3.212.0_CDS_at	11	0.910	1.419	1.822
Pf.3.214.0_CDS_at	26	1.839	**0.361**	**0.434**
Pf.3.216.0_CDS_at	8	0.952	0.669	0.903
Pf.3.217.0_CDS_at	18	**0.431**	0.550	0.761
Pf.3.218.0_CDS_at	143	1.030	**0.469**	0.565
Pf.3.219.0_CDS_at	73	1.236	1.184	1.744
Pf.3.22.0_CDS_at	943	**0.478**	0.952	1.040
Pf.3.220.0_CDS_at	695	1.582	**2.585**	1.808
Pf.3.221.0_CDS_at	89	**7.110**	**9.135**	0.839
Pf.3.222.0_CDS_at	62	**3.935**	**6.514**	**7.539**
Pf.3.223.0_CDS_at	16	1.179	1.400	1.416
Pf.3.224.0_CDS_at	45	**1.726**	**2.651**	**4.222**
Pf.3.225.0_CDS_at	118	1.319	1.040	0.588
Pf.3.226.0_CDS_at	11	0.826	0.953	1.122
Pf.3.228.0_CDS_at	753	0.507	**0.427**	1.679
Pf.3.229.0_CDS_at	28	0.822	0.733	1.738
Pf.3.23.0_CDS_at	775	0.966	1.719	1.380
Pf.3.231.0_CDS_at	212	1.739	1.019	1.606
Pf.3.233.0_CDS_at	853	0.900	1.141	1.786
Pf.3.235.0_CDS_at	9	**2.700**	1.897	**2.609**
Pf.3.236.0_CDS_at	7	2.317	1.051	0.706
Pf.3.238.0_CDS_at	35	1.614	1.674	**2.148**
Pf.3.239.0_CDS_at	20	1.429	0.617	0.449
Pf.3.24.0_CDS_at	7	1.514	1.106	1.259
Pf.3.240.0_CDS_at	707	0.676	1.425	**2.644**
Pf.3.241.0_CDS_at	181	**0.089**	**0.093**	**0.071**
Pf.3.242.0_CDS_s_at	5	0.967	0.998	0.983
Pf.3.252.0_at	2524	1.908	**2.128**	1.678
Pf.3.26.0_CDS_at	417	0.536	0.750	1.702
Pf.3.262.0_at	528	**0.454**	**0.238**	0.765
Pf.3.27.0_CDS_at	1135	0.667	0.606	0.738
Pf.3.278.0_at	70	1.316	1.068	1.177
Pf.3.28.0_CDS_at	36	**0.336**	**0.407**	0.729
Pf.3.29.0_CDS_at	2056	0.568	1.214	0.583
Pf.3.3.0_CDS_at	19	0.924	0.597	0.539
Pf.3.3.1_CDS_at	38	**0.442**	**0.425**	0.763
Pf.3.3.2_s_at	35	1.339	0.702	0.773
Pf.3.31.0_CDS_at	2974	0.697	0.775	0.574
Pf.3.31.1_a_at	677	0.643	0.900	0.795
Pf.3.32.0_CDS_at	102	1.171	1.142	1.653
Pf.3.34.0_CDS_at	559	**0.407**	0.806	**0.423**
Pf.3.35.0_CDS_at	83	**3.375**	1.750	1.743
Pf.3.36.0_CDS_at	15343	1.022	1.071	1.348
Pf.3.37.0_CDS_at	723	0.682	0.887	0.869
Pf.3.38.0_CDS_at	181	**0.370**	**0.431**	**0.279**
Pf.3.39.0_CDS_at	34	1.499	0.990	1.009
Pf.3.39.1_a_at	21	1.074	0.960	0.820

Pf.3.40.0_CDS_at	1113	0.644	0.857	0.609
Pf.3.41.0_CDS_at	159	0.761	1.063	**2.225**
Pf.3.42.0_CDS_at	167	1.235	1.478	**2.030**
Pf.3.42.0_CDS_x_at	155	1.585	1.701	**2.264**
Pf.3.43.0_CDS_at	87	0.579	0.659	1.780
Pf.3.44.0_CDS_at	671	**0.421**	0.992	0.521
Pf.3.45.0_CDS_at	687	0.861	1.371	1.231
Pf.3.46.0_CDS_at	209	1.126	1.657	**3.173**
Pf.3.48.0_CDS_at	1224	0.961	**0.300**	0.845
Pf.3.49.0_CDS_at	6	1.047	0.836	1.289
Pf.3.49.0_CDS_x_at	22	0.930	0.708	0.960
Pf.3.5.0_CDS_at	4853	1.170	**0.476**	1.099
Pf.3.51.0_CDS_at	156	**0.329**	**0.432**	**2.248**
Pf.3.53.0_CDS_at	25	0.663	0.972	0.952
Pf.3.54.0_CDS_at	1173	0.828	0.557	1.691
Pf.3.55.0_CDS_at	257	0.676	0.892	0.671
Pf.3.57.0_CDS_at	11	1.248	0.603	0.774
Pf.3.58.0_CDS_at	393	0.841	1.195	1.143
Pf.3.59.0_CDS_at	69	0.705	0.889	1.161
Pf.3.6.0_CDS_at	258	0.857	1.584	1.386
Pf.3.60.0_CDS_at	9	0.888	0.767	0.648
Pf.3.61.0_CDS_at	242	1.248	1.466	1.959
Pf.3.62.0_CDS_at	338	**2.094**	**2.657**	**2.389**
Pf.3.63.0_CDS_at	664	**2.436**	1.954	1.950
Pf.3.64.0_CDS_at	568	**2.517**	**2.096**	1.551
Pf.3.65.0_CDS_at	1847	0.990	0.842	1.421
Pf.3.66.0_CDS_at	369	**0.424**	**0.210**	**0.173**
Pf.3.7.0_CDS_at	1393	**0.465**	1.005	1.371
Pf.3.70.0_CDS_at	556	1.161	0.885	1.412
Pf.3.71.0_CDS_at	89	0.524	**0.425**	**0.335**
Pf.3.72.0_CDS_at	1502	0.620	1.117	0.943
Pf.3.73.0_CDS_at	17	1.043	1.386	**3.283**
Pf.3.75.0_CDS_at	1853	1.596	1.476	1.322
Pf.3.76.0_CDS_at	7	0.934	0.970	0.803
Pf.3.77.0_CDS_at	34	1.146	0.919	1.205
Pf.3.78.0_CDS_at	289	**2.289**	**2.128**	1.598
Pf.3.79.0_CDS_at	44	**0.469**	**0.217**	**0.419**
Pf.3.8.0_CDS_at	1593	**0.245**	**0.253**	**0.428**
Pf.3.82.0_CDS_at	1150	**0.286**	**0.274**	0.965
Pf.3.83.0_CDS_at	595	1.519	1.552	1.426
Pf.3.84.0_CDS_at	662	**0.196**	**0.193**	1.079
Pf.3.86.0_CDS_x_at	5	0.913	1.000	1.036
Pf.3.87.0_CDS_at	11	1.552	1.831	**2.817**
Pf.3.88.0_CDS_at	120	**0.156**	**0.257**	0.842
Pf.3.89.0_CDS_at	16	0.513	0.921	1.697
Pf.3.9.0_CDS_at	9155	1.119	1.043	1.173
Pf.3.9.0_CDS_x_at	12672	1.027	1.015	1.018
Pf.3.90.0_CDS_at	6	1.871	1.657	2.168
Pf.3.91.0_CDS_at	83	0.637	0.928	**0.278**
Pf.3.92.0_CDS_a_at	671	**0.408**	**0.466**	0.577
Pf.3.93.0_CDS_at	395	**0.383**	0.748	1.342
Pf.3.94.0_CDS_at	305	**2.840**	**3.622**	1.718
Pf.3.96.0_CDS_at	56	1.171	0.894	1.394
Pf.3.97.0_CDS_at	154	0.756	0.704	1.435
Pf.3.98.0_CDS_at	69	**0.470**	0.870	**0.274**
Pf.3.99.0_CDS_at	1517	**0.365**	**0.266**	**0.150**
Pf.4.1.0_CDS_at	6284	1.133	1.103	1.281
Pf.4.10.0_CDS_at	125	**2.399**	**5.159**	**5.078**
Pf.4.100.0_CDS_at	840	1.254	1.271	1.286
Pf.4.101.0_CDS_a_at	124	**0.476**	**4.816**	**3.563**
Pf.4.101.1_at	220	1.294	1.212	1.127
Pf.4.102.0_CDS_at	1109	0.589	1.387	1.170

Pf.4.103.0_CDS_a_at	680	1.407	1.640	1.224
Pf.4.105.0_CDS_at	252	0.556	0.774	**3.201**
Pf.4.106.0_CDS_at	83	0.733	1.419	1.639
Pf.4.107.0_CDS_s_at	1840	1.198	1.134	0.605
Pf.4.107.0_CDS_x_at	339	**3.154**	**2.428**	0.901
Pf.4.109.0_CDS_at	161	0.691	0.888	1.630
Pf.4.11.0_CDS_at	22	1.386	**2.020**	0.504
Pf.4.110.0_CDS_at	128	1.296	1.028	1.771
Pf.4.111.0_CDS_at	9	1.016	0.737	0.717
Pf.4.114.0_CDS_at	71	1.672	1.912	**3.801**
Pf.4.115.0_CDS_at	1276	**0.412**	0.594	0.790
Pf.4.117.0_CDS_at	73	1.168	**0.460**	0.251
Pf.4.118.0_CDS_at	2072	0.767	0.713	1.346
Pf.4.119.0_CDS_x_at	9	0.763	1.137	1.042
Pf.4.120.0_CDS_at	6	0.871	0.755	0.926
Pf.4.121.0_CDS_at	6	0.886	0.963	0.924
Pf.4.122.0_CDS_at	7	0.898	0.922	0.876
Pf.4.123.0_CDS_at	6	1.042	0.888	0.900
Pf.4.124.0_CDS_at	13	1.827	**4.312**	0.957
Pf.4.125.0_CDS_at	22	**2.159**	**2.268**	1.416
Pf.4.126.0_CDS_at	8	1.400	1.305	1.162
Pf.4.127.0_CDS_at	15	0.773	**0.328**	**0.317**
Pf.4.128.0_CDS_at	41	1.246	0.551	0.875
Pf.4.129.0_CDS_at	68	**0.320**	1.273	**2.766**
Pf.4.13.0_CDS_at	437	**2.390**	1.849	1.275
Pf.4.130.0_CDS_a_at	3574	**0.184**	**0.294**	0.732
Pf.4.130.0_CDS_x_at	322	**0.176**	**0.296**	0.832
Pf.4.130.1_CDS_a_at	1838	**0.134**	**0.237**	0.654
Pf.4.134.0_CDS_at	478	**0.464**	0.596	1.799
Pf.4.137.0_CDS_at	35	**0.464**	1.220	**0.386**
Pf.4.138.0_CDS_at	17	0.831	1.506	1.690
Pf.4.139.0_CDS_at	311	0.862	0.849	0.898
Pf.4.14.0_CDS_at	13	0.571	0.779	0.554
Pf.4.140.0_CDS_at	184	0.887	1.346	**2.202**
Pf.4.142.0_CDS_at	45	**3.047**	0.524	0.642
Pf.4.144.0_CDS_at	10	1.123	1.352	0.867
Pf.4.146.0_CDS_at	17	1.774	1.338	0.781
Pf.4.147.0_CDS_at	40	1.946	**0.249**	**0.372**
Pf.4.148.0_CDS_at	149	1.014	1.276	1.392
Pf.4.149.0_CDS_at	10	1.160	0.827	0.695
Pf.4.15.0_CDS_at	9	0.735	1.066	0.910
Pf.4.150.0_CDS_at	83	**2.267**	**0.225**	**0.389**
Pf.4.151.0_CDS_at	243	0.808	0.545	0.633
Pf.4.153.0_CDS_at	22	1.176	0.927	0.559
Pf.4.155.0_CDS_at	11	0.895	**0.449**	**0.462**
Pf.4.157.0_CDS_at	261	**0.111**	**0.090**	**0.102**
Pf.4.158.0_CDS_at	262	**4.199**	**2.219**	**2.459**
Pf.4.159.0_CDS_at	26	0.801	0.728	1.548
Pf.4.16.0_CDS_at	83	0.826	0.836	**0.423**
Pf.4.161.0_CDS_at	5	1.051	1.076	0.953
Pf.4.161.0_CDS_x_at	5	0.881	0.901	0.858
Pf.4.162.0_CDS_at	5	1.139	1.086	1.153
Pf.4.167.0_CDS_at	672	1.572	**0.572**	1.565
Pf.4.168.0_CDS_a_at	51	**5.705**	1.262	0.858
Pf.4.17.0_CDS_at	184	0.703	0.701	0.723
Pf.4.170.0_CDS_at	127	**11.713**	**3.524**	**2.221**
Pf.4.171.0_CDS_at	1152	**4.439**	**4.083**	**3.497**
Pf.4.172.0_CDS_at	6	1.174	0.890	0.928
Pf.4.174.0_CDS_at	8	1.055	0.795	0.899
Pf.4.177.0_CDS_s_at	5	1.067	1.101	1.072
Pf.4.178.0_CDS_x_at	5	1.323	1.189	1.024
Pf.4.179.0_CDS_at	7	1.007	0.869	0.936

Pf.4.18.0_CDS_a_at	41	0.619	0.600	0.546
Pf.4.180.0_CDS_at	6	1.008	0.956	1.020
Pf.4.181.0_CDS_x_at	5	0.903	0.999	0.847
Pf.4.182.0_CDS_at	5	1.273	1.402	1.062
Pf.4.183.0_CDS_s_at	8	1.001	0.986	1.090
Pf.4.184.0_CDS_at	6	0.976	0.967	1.078
Pf.4.185.0_CDS_at	11	0.764	0.863	0.710
Pf.4.186.0_CDS_at	37	**2.013**	1.653	1.792
Pf.4.187.0_CDS_at	315	1.409	**2.959**	1.448
Pf.4.188.0_CDS_at	13	**3.310**	0.540	0.644
Pf.4.189.0_CDS_at	9	1.300	0.722	0.713
Pf.4.19.0_CDS_at	190	**0.276**	**0.359**	0.639
Pf.4.190.0_CDS_at	7	1.608	1.475	1.219
Pf.4.191.0_CDS_at	13	1.169	1.688	0.712
Pf.4.194.0_CDS_at	16	0.539	0.655	0.780
Pf.4.196.0_CDS_at	95	1.347	1.739	1.831
Pf.4.197.0_CDS_at	15	1.525	0.799	1.159
Pf.4.198.0_CDS_at	6	1.118	0.939	0.789
Pf.4.199.0_CDS_at	8	2.380	0.983	0.852
Pf.4.2.0_CDS_a_at	10	1.471	0.855	0.790
Pf.4.2.1_a_at	9	1.078	0.783	0.993
Pf.4.20.0_CDS_at	136	**0.217**	0.851	0.904
Pf.4.200.0_CDS_at	23	0.860	1.310	**2.677**
Pf.4.201.0_CDS_at	6	1.092	1.563	1.379
Pf.4.201.0_CDS_x_at	15	1.867	**2.292**	**2.151**
Pf.4.202.0_CDS_at	153	1.310	0.596	0.589
Pf.4.203.0_CDS_at	23	1.766	1.411	**2.522**
Pf.4.204.0_CDS_at	2695	0.717	0.643	0.898
Pf.4.207.0_CDS_at	16	0.875	0.698	0.956
Pf.4.208.0_CDS_a_at	9	1.354	1.177	0.945
Pf.4.209.0_CDS_at	7	1.658	0.917	0.738
Pf.4.210.0_CDS_at	9	0.603	0.574	0.685
Pf.4.212.0_CDS_at	100	**0.127**	**0.208**	**0.184**
Pf.4.213.0_CDS_at	13	0.789	**0.459**	0.808
Pf.4.214.0_CDS_at	1006	**4.845**	**2.370**	**2.075**
Pf.4.215.0_CDS_at	26	0.955	0.597	0.748
Pf.4.217.0_CDS_at	46	0.961	0.735	1.731
Pf.4.218.0_CDS_at	464	1.346	0.720	0.742
Pf.4.22.0_CDS_at	374	**3.951**	**3.680**	**2.106**
Pf.4.221.0_CDS_at	6	1.157	1.290	0.998
Pf.4.221.1_a_at	7	0.963	1.003	1.041
Pf.4.222.0_CDS_s_at	17	0.807	1.446	0.798
Pf.4.223.0_CDS_x_at	1677	**6.269**	1.992	1.997
Pf.4.224.0_CDS_x_at	1692	**6.011**	1.910	1.975
Pf.4.225.0_CDS_at	5	1.110	0.998	0.927
Pf.4.226.0_CDS_at	150	0.627	1.255	1.276
Pf.4.227.0_CDS_at	89	1.214	**0.446**	1.250
Pf.4.228.0_CDS_at	35	0.617	**0.277**	**0.298**
Pf.4.229.0_CDS_at	143	1.520	1.745	1.816
Pf.4.23.0_CDS_at	164	0.709	0.783	0.845
Pf.4.23.0_CDS_x_at	499	0.871	0.966	0.945
Pf.4.231.0_CDS_at	8	0.848	1.307	0.977
Pf.4.231.0_CDS_x_at	21	0.844	1.263	0.550
Pf.4.232.0_CDS_at	24	0.808	0.624	**0.468**
Pf.4.233.0_CDS_at	124	**0.250**	**0.356**	1.836
Pf.4.234.0_CDS_at	4	1.852	1.559	1.701
Pf.4.235.0_CDS_s_at	6	1.139	1.772	0.980
Pf.4.237.0_CDS_x_at	8	1.300	1.166	0.849
Pf.4.238.0_CDS_at	123	1.350	1.917	**0.385**
Pf.4.238.0_CDS_s_at	5	1.189	0.978	0.945
Pf.4.238.0_CDS_x_at	81	1.304	1.829	**0.335**
Pf.4.24.0_CDS_at	1336	1.921	**2.322**	1.702

Pf.4.242.0_CDS_at	121	1.089	0.547	1.003
Pf.4.243.0_CDS_at	9	1.060	0.825	0.687
Pf.4.246.0_CDS_at	6	1.108	1.007	0.972
Pf.4.247.0_CDS_at	8	1.000	0.796	0.923
Pf.4.248.0_CDS_s_at	1140	1.660	1.287	0.601
Pf.4.249.0_at	472	**0.460**	**0.492**	0.527
Pf.4.25.0_CDS_at	251	0.639	0.665	0.842
Pf.4.250.0_a_at	332	**0.406**	0.575	1.542
Pf.4.26.0_CDS_at	542	**2.390**	1.952	1.475
Pf.4.27.0_CDS_at	186	1.310	1.020	1.542
Pf.4.279.0_at	9	0.924	0.995	0.882
Pf.4.28.0_CDS_a_at	152	1.039	**2.085**	**0.289**
Pf.4.280.0_at	40	1.282	**0.477**	0.735
Pf.4.288.0_at	1044	0.538	**0.307**	1.149
Pf.4.29.0_CDS_at	16	1.560	1.012	0.656
Pf.4.3.0_CDS_a_at	4614	1.517	1.181	1.404
Pf.4.31.0_CDS_at	68	1.934	1.603	**2.881**
Pf.4.32.0_CDS_at	1349	0.572	0.527	1.081
Pf.4.33.0_CDS_at	92	0.781	1.335	0.805
Pf.4.36.0_CDS_x_at	9	0.827	0.676	0.791
Pf.4.37.0_CDS_at	22	0.915	**0.490**	0.381
Pf.4.38.0_CDS_at	42	1.238	1.438	1.336
Pf.4.39.0_CDS_at	765	**0.223**	**0.288**	**0.178**
Pf.4.4.0_CDS_at	885	**0.275**	**0.178**	**0.127**
Pf.4.40.0_CDS_at	110	0.774	**2.287**	**3.594**
Pf.4.41.0_CDS_a_at	1054	**2.609**	**2.454**	1.810
Pf.4.42.0_CDS_at	124	**2.125**	1.592	1.463
Pf.4.43.0_CDS_at	2087	1.043	1.301	1.200
Pf.4.44.0_CDS_at	166	1.656	**2.249**	**2.412**
Pf.4.45.0_CDS_a_at	416	**0.179**	**0.339**	**0.123**
Pf.4.46.0_CDS_at	486	**3.028**	**2.451**	**2.705**
Pf.4.47.0_CDS_at	263	0.588	1.389	1.808
Pf.4.49.0_CDS_at	145	1.085	1.636	1.953
Pf.4.5.0_CDS_at	75	**0.150**	**0.173**	**0.108**
Pf.4.50.0_CDS_at	386	1.566	1.513	1.203
Pf.4.51.0_CDS_at	69	1.680	1.833	1.599
Pf.4.54.0_CDS_at	1190	**3.188**	1.885	1.936
Pf.4.55.0_CDS_at	10	1.311	0.959	0.947
Pf.4.56.0_CDS_at	185	0.566	1.026	1.283
Pf.4.57.0_CDS_at	772	1.503	1.581	1.311
Pf.4.58.0_CDS_at	9	0.688	0.780	1.036
Pf.4.59.0_CDS_at	964	0.640	0.586	0.805
Pf.4.6.0_CDS_at	604	**0.131**	**0.163**	**0.139**
Pf.4.60.0_CDS_a_at	868	**0.196**	**0.280**	0.591
Pf.4.61.0_CDS_at	93	0.714	0.805	1.721
Pf.4.63.0_CDS_at	26	0.998	0.723	**2.047**
Pf.4.65.0_CDS_at	598	**0.261**	0.905	0.748
Pf.4.67.0_CDS_at	323	0.711	0.662	1.342
Pf.4.68.0_CDS_at	3435	**0.105**	**0.213**	**0.070**
Pf.4.69.0_CDS_at	206	**0.101**	**0.087**	**0.089**
Pf.4.7.0_CDS_a_at	5283	0.964	0.806	1.146
Pf.4.70.0_CDS_at	50	1.883	**2.193**	**2.041**
Pf.4.70.0_CDS_x_at	42	**2.240**	**2.171**	**2.115**
Pf.4.71.0_CDS_at	10	1.747	1.625	0.914
Pf.4.73.0_CDS_at	62	0.575	**0.492**	**0.228**
Pf.4.74.0_CDS_at	56	0.620	1.205	1.343
Pf.4.76.0_CDS_at	260	**0.466**	0.714	1.108
Pf.4.77.0_CDS_at	170	**3.410**	**2.668**	**4.372**
Pf.4.78.0_CDS_at	21	1.362	1.536	**2.115**
Pf.4.78.0_CDS_x_at	41	1.207	1.873	1.959
Pf.4.79.0_CDS_at	42	1.154	1.997	**2.636**
Pf.4.8.0_CDS_a_at	61	0.521	0.572	0.660

Pf.4.80.0_CDS_at	1291	**0.476**	**0.331**	**0.217**
Pf.4.83.0_CDS_at	438	1.140	1.902	1.474
Pf.4.84.0_CDS_at	23	0.863	0.918	0.665
Pf.4.86.0_CDS_at	101	**0.476**	1.777	**2.427**
Pf.4.87.0_CDS_at	9	0.734	0.669	1.043
Pf.4.88.0_CDS_at	621	**0.215**	**0.160**	**0.129**
Pf.4.89.0_CDS_a_at	441	1.974	**0.241**	**0.212**
Pf.4.89.1_CDS_at	118	1.177	0.894	0.665
Pf.4.9.0_CDS_at	529	0.500	**0.381**	0.829
Pf.4.90.0_CDS_at	529	1.081	1.323	**2.291**
Pf.4.91.0_CDS_at	750	0.627	1.197	1.782
Pf.4.92.0_CDS_at	209	1.697	1.980	**3.196**
Pf.4.93.0_CDS_at	87	0.737	0.501	0.710
Pf.4.95.0_CDS_at	2317	0.758	0.717	0.824
Pf.4.97.0_CDS_at	114	0.604	1.750	0.551
Pf.4.98.0_CDS_at	11	0.656	0.901	0.968
Pf.4.99.0_CDS_a_at	66	0.653	**0.493**	**0.187**
Pf.5.1.0_CDS_s_at	522	**3.075**	1.720	0.790
Pf.5.10.0_CDS_a_at	1469	1.092	1.108	1.208
Pf.5.100.0_CDS_at	211	0.645	1.220	**2.409**
Pf.5.101.0_CDS_at	1006	1.906	1.482	1.913
Pf.5.104.0_CDS_at	276	0.684	0.702	1.115
Pf.5.105.0_CDS_at	439	1.135	0.970	1.990
Pf.5.106.0_CDS_at	123	**0.274**	**0.391**	0.560
Pf.5.107.0_CDS_at	701	**2.380**	1.607	1.740
Pf.5.108.0_CDS_at	2952	**0.047**	**0.090**	**0.028**
Pf.5.109.0_CDS_at	123	0.619	0.801	1.009
Pf.5.11.0_CDS_at	1692	1.270	1.183	1.189
Pf.5.110.0_CDS_at	27	1.473	1.216	1.065
Pf.5.111.0_CDS_at	726	1.605	**2.139**	1.432
Pf.5.112.0_CDS_at	41	1.350	0.530	0.979
Pf.5.113.0_CDS_at	29	**0.318**	0.941	1.926
Pf.5.114.0_CDS_at	173	**0.410**	0.635	1.070
Pf.5.115.0_CDS_at	1914	**2.514**	1.463	1.723
Pf.5.116.0_CDS_at	723	1.646	1.856	1.668
Pf.5.117.0_CDS_at	38	1.400	**2.194**	**3.652**
Pf.5.118.0_CDS_at	3042	**0.223**	**0.392**	0.742
Pf.5.119.0_CDS_at	601	**4.085**	**3.641**	1.163
Pf.5.119.0_CDS_x_at	3188	**3.400**	**2.893**	0.884
Pf.5.120.0_CDS_at	169	0.573	1.337	1.609
Pf.5.121.0_CDS_at	202	**0.346**	**0.215**	**0.106**
Pf.5.125.0_CDS_at	489	1.056	1.455	1.416
Pf.5.126.0_CDS_at	129	0.513	**0.399**	0.527
Pf.5.128.0_CDS_at	259	1.246	1.096	**1.815**
Pf.5.129.0_CDS_at	13	1.028	1.309	1.179
Pf.5.13.0_CDS_at	7430	1.135	1.105	1.221
Pf.5.130.0_CDS_at	37	0.672	0.645	0.820
Pf.5.132.0_CDS_at	369	1.527	1.341	0.783
Pf.5.134.0_CDS_at	9895	0.735	0.867	0.963
Pf.5.135.0_CDS_at	5	0.955	0.957	0.934
Pf.5.135.0_CDS_x_at	6	0.889	0.979	1.014
Pf.5.136.0_CDS_at	930	**2.589**	**2.533**	**2.378**
Pf.5.137.0_CDS_at	1922	1.924	1.906	1.375
Pf.5.138.0_CDS_at	268	**2.292**	**2.154**	**2.164**
Pf.5.139.0_CDS_at	218	0.624	0.777	1.760
Pf.5.14.1_a_at	824	**0.406**	0.604	0.659
Pf.5.140.0_CDS_at	171	1.526	**2.039**	**2.968**
Pf.5.141.0_CDS_at	342	1.055	1.353	**2.563**
Pf.5.142.0_CDS_at	1390	**0.221**	**0.092**	**0.090**
Pf.5.144.0_CDS_at	31	**0.298**	**0.238**	**0.248**
Pf.5.145.0_CDS_at	13	1.186	1.281	1.695
Pf.5.147.0_CDS_at	4340	1.017	1.061	1.191

Pf.5.15.0_CDS_at	603	1.437	1.819	1.244
Pf.5.150.0_CDS_at	565	**0.208**	**0.133**	**0.133**
Pf.5.152.0_CDS_at	179	0.827	**0.495**	**0.350**
Pf.5.153.0_CDS_at	112	0.592	0.707	0.716
Pf.5.154.0_CDS_at	198	0.535	0.923	1.269
Pf.5.154.0_CDS_x_at	270	0.506	0.805	1.196
Pf.5.155.0_CDS_at	34	1.271	**2.152**	1.656
Pf.5.156.0_CDS_a_at	2301	1.375	1.524	1.275
Pf.5.157.0_CDS_at	8	1.648	0.959	1.161
Pf.5.157.0_CDS_x_at	11	**2.873**	0.844	1.217
Pf.5.158.0_CDS_at	19	1.210	0.734	0.853
Pf.5.159.0_CDS_at	126	1.164	1.783	1.523
Pf.5.16.0_CDS_a_at	239	**0.348**	1.115	0.744
Pf.5.16.2_a_at	407	0.992	1.669	1.451
Pf.5.160.0_CDS_at	10	1.127	1.625	**4.344**
Pf.5.161.0_CDS_at	123	**2.107**	**2.036**	**2.876**
Pf.5.161.0_CDS_x_at	139	**2.698**	**2.504**	**3.305**
Pf.5.162.0_CDS_at	349	1.425	**2.337**	0.889
Pf.5.163.0_CDS_at	80	0.695	0.853	**0.371**
Pf.5.164.0_CDS_at	1584	0.734	1.008	1.553
Pf.5.165.0_CDS_at	66	**2.932**	0.930	**2.189**
Pf.5.166.0_CDS_at	70	1.050	1.062	**2.527**
Pf.5.168.0_CDS_at	297	1.643	1.369	**2.296**
Pf.5.169.0_CDS_at	304	**0.156**	**0.276**	0.899
Pf.5.172.0_CDS_at	247	0.632	1.007	1.916
Pf.5.173.0_CDS_at	116	1.401	1.567	**2.480**
Pf.5.174.0_CDS_x_at	4	1.035	0.928	1.007
Pf.5.176.0_CDS_at	86	1.966	0.933	1.690
Pf.5.178.0_CDS_at	832	**0.190**	**0.195**	0.845
Pf.5.179.0_CDS_at	7	0.664	0.885	1.261
Pf.5.18.0_CDS_at	996	**0.022**	**0.038**	**0.025**
Pf.5.180.0_CDS_at	1274	**0.247**	**0.466**	0.614
Pf.5.182.0_CDS_at	276	0.637	0.534	**0.272**
Pf.5.183.0_CDS_at	48	0.566	**0.370**	**2.816**
Pf.5.183.0_CDS_x_at	58	0.586	0.680	**2.873**
Pf.5.184.0_CDS_at	10	1.036	1.031	1.991
Pf.5.185.0_CDS_at	651	0.770	0.944	1.621
Pf.5.186.0_CDS_at	17	0.676	1.180	**5.001**
Pf.5.187.0_CDS_at	4006	**0.116**	**0.264**	0.817 #8
Pf.5.188.0_CDS_at	8	1.187	0.719	0.995
Pf.5.189.0_CDS_at	777	1.206	1.347	1.044
Pf.5.19.0_CDS_at	23	0.931	**0.494**	0.974
Pf.5.190.0_CDS_at	9	0.989	0.791	0.995
Pf.5.191.0_CDS_at	10	1.030	0.703	0.703
Pf.5.192.0_CDS_at	600	1.059	1.343	1.844
Pf.5.193.0_CDS_at	37	0.670	0.878	1.033
Pf.5.194.0_CDS_at	8	1.190	1.009	1.269
Pf.5.195.0_CDS_at	456	1.050	1.475	1.403
Pf.5.197.0_CDS_at	9	1.777	0.962	0.879
Pf.5.199.0_CDS_at	18	**3.191**	0.935	1.196
Pf.5.2.0_CDS_at	4090	**0.436**	0.504	**0.479**
Pf.5.20.0_CDS_at	7639	0.553	0.607	1.079
Pf.5.202.0_CDS_at	23	0.854	**0.494**	1.238
Pf.5.204.0_CDS_at	113	1.357	1.985	1.863
Pf.5.206.0_CDS_at	415	1.821	1.110	0.935
Pf.5.207.0_CDS_at	16	1.457	0.994	1.153
Pf.5.208.0_CDS_at	683	1.769	1.255	1.839
Pf.5.209.0_CDS_at	28	**4.047**	1.308	**3.199**
Pf.5.21.0_CDS_at	82	0.538	0.558	**0.315**
Pf.5.210.0_CDS_at	37	1.234	0.952	1.590
Pf.5.211.0_CDS_at	148	1.004	0.911	1.223
Pf.5.215.0_CDS_at	338	1.716	**2.112**	1.695
Pf.5.216.0_CDS_at	164	0.751	1.203	**2.660**

Pf.5.217.0_CDS_at	34	0.593	1.713	**2.509**
Pf.5.218.0_CDS_at	313	0.986	1.143	1.398
Pf.5.219.0_CDS_at	9	0.822	0.905	1.097
Pf.5.22.0_CDS_at	820	**0.200**	**0.129**	**0.125**
Pf.5.222.0_CDS_at	167	0.859	0.525	0.763
Pf.5.223.0_CDS_at	2299	**0.109**	**0.084**	**0.054**
Pf.5.225.0_CDS_at	15	1.565	0.793	0.651
Pf.5.226.0_CDS_at	55	1.337	1.375	1.845
Pf.5.227.0_CDS_at	10	0.684	1.049	1.269
Pf.5.228.0_CDS_at	42	1.296	1.822	**2.109**
Pf.5.228.0_CDS_x_at	50	1.610	**2.011**	**2.487**
Pf.5.23.0_CDS_at	190	**0.424**	0.695	0.630
Pf.5.230.0_CDS_at	886	**2.486**	**2.230**	**2.277**
Pf.5.231.0_CDS_at	14	0.815	0.561	0.716
Pf.5.232.0_CDS_at	243	**0.393**	**0.390**	0.654
Pf.5.234.0_CDS_at	65	**0.497**	**0.192**	**0.363**
Pf.5.236.0_CDS_at	196	1.782	**0.194**	0.568
Pf.5.237.0_CDS_at	456	1.147	1.452	1.612
Pf.5.238.0_CDS_at	36	0.948	0.652	0.915
Pf.5.239.0_CDS_at	28	0.495	0.605	0.670
Pf.5.24.0_CDS_at	242	**3.474**	**3.830**	**2.378**
Pf.5.240.0_CDS_at	59	**2.063**	1.073	1.878
Pf.5.241.0_CDS_at	18	**2.109**	0.599	0.757
Pf.5.241.0_CDS_x_at	46	**2.595**	**0.314**	0.562
Pf.5.244.3_a_at	37	1.071	1.052	1.316
Pf.5.245.0_CDS_at	18	**10.146**	**3.590**	1.001
Pf.5.245.0_CDS_x_at	331	**9.585**	**3.810**	0.949
Pf.5.246.0_CDS_x_at	1350	**2.206**	**2.065**	1.851
Pf.5.247.0_CDS_s_at	4037	1.651	1.718	0.832
Pf.5.248.0_CDS_at	6	0.985	0.915	1.087
Pf.5.249.0_CDS_at	7	1.575	2.049	1.000
Pf.5.25.0_CDS_at	519	0.639	0.917	1.520
Pf.5.250.0_CDS_at	28	**2.313**	1.188	1.299
Pf.5.250.0_CDS_x_at	31	**2.882**	1.728	**2.130**
Pf.5.251.0_CDS_x_at	1105	1.475	**0.137**	**0.316**
Pf.5.252.0_CDS_at	6	1.068	1.117	0.916
Pf.5.253.0_CDS_at	5	1.086	1.231	1.117
Pf.5.254.0_CDS_at	6	1.056	0.876	0.808
Pf.5.254.0_CDS_s_at	7	0.893	0.880	0.794
Pf.5.255.0_CDS_at	71	1.649	0.746	**0.287**
Pf.5.256.0_CDS_at	126	1.414	0.561	**0.497**
Pf.5.257.0_CDS_at	503	**0.431**	**0.131**	**0.065**
Pf.5.258.0_CDS_at	237	1.165	0.950	**2.686**
Pf.5.259.0_CDS_at	9	1.157	0.656	0.775
Pf.5.26.0_CDS_at	14	0.703	**2.297**	**4.910**
Pf.5.262.0_CDS_at	16	**2.072**	**2.497**	**2.333**
Pf.5.263.0_CDS_at	260	0.958	1.414	1.866
Pf.5.264.0_CDS_at	43	1.098	1.769	**2.286**
Pf.5.265.0_CDS_at	13	0.774	0.689	0.959
Pf.5.266.0_CDS_at	71	0.619	1.352	**4.787**
Pf.5.268.0_CDS_at	13	0.939	0.645	0.967
Pf.5.27.0_CDS_at	807	1.961	**2.305**	1.396
Pf.5.270.0_CDS_at	389	0.797	0.870	1.820
Pf.5.271.0_CDS_at	470	**0.178**	**0.377**	0.577
Pf.5.272.0_CDS_at	649	**0.450**	0.699	1.236
Pf.5.273.0_CDS_at	32	1.714	**0.227**	**0.328**
Pf.5.274.0_CDS_at	116	**0.366**	**0.289**	**0.335**
Pf.5.275.0_CDS_at	97	0.767	**0.271**	**0.359**
Pf.5.276.0_CDS_at	135	**0.260**	**0.104**	**0.111**
Pf.5.277.0_CDS_at	376	**0.452**	1.223	1.820
Pf.5.278.0_CDS_at	18	0.590	0.877	1.122
Pf.5.279.0_CDS_at	21	**2.974**	1.183	1.068

Pf.5.28.0_CDS_at	224	0.965	1.146	1.391
Pf.5.281.0_CDS_at	94	1.680	**2.375**	**5.257**
Pf.5.282.0_CDS_at	72	1.551	**2.607**	**2.024**
Pf.5.283.0_CDS_at	12	0.727	0.650	0.733
Pf.5.284.0_CDS_at	22	0.990	0.786	1.126
Pf.5.286.0_CDS_a_at	133	1.386	0.516	1.224
Pf.5.287.0_CDS_at	27	**2.355**	**3.287**	**2.492**
Pf.5.288.0_CDS_at	486	1.017	**2.632**	**2.044**
Pf.5.289.0_CDS_at	43	1.547	**0.460**	1.302
Pf.5.29.0_CDS_at	1100	0.675	1.107	0.934
Pf.5.290.0_CDS_at	41	1.206	**0.390**	**0.567**
Pf.5.291.0_CDS_at	182	**0.469**	1.026	1.615
Pf.5.292.0_CDS_at	10457	0.924	0.976	1.255
Pf.5.294.0_CDS_at	50	**2.404**	1.584	**2.284**
Pf.5.295.0_CDS_at	307	0.929	1.639	**0.296**
Pf.5.296.0_CDS_at	99	1.046	**0.416**	**0.546**
Pf.5.298.0_CDS_a_at	6	0.946	0.871	0.766
Pf.5.299.0_CDS_at	18	**2.652**	0.604	0.716
Pf.5.3.0_CDS_s_at	20866	1.180	1.173	1.131
Pf.5.30.0_CDS_at	364	1.029	1.029	**0.396**
Pf.5.300.0_CDS_at	39	**2.946**	0.610	1.303
Pf.5.301.0_CDS_at	959	**3.306**	**2.819**	1.911
Pf.5.302.0_CDS_at	11	1.041	0.611	0.861
Pf.5.303.0_CDS_at	9	1.328	1.736	**2.516**
Pf.5.304.0_CDS_at	8	0.763	0.732	1.222
Pf.5.305.0_CDS_at	33	1.996	**3.331**	0.375
Pf.5.306.0_CDS_at	2684	1.713	1.702	1.669
Pf.5.307.0_CDS_at	717	0.732	1.360	0.700
Pf.5.31.0_CDS_at	1326	**0.273**	**0.165**	**0.078**
Pf.5.310.0_CDS_at	66	0.555	**2.284**	**3.120**
Pf.5.311.0_CDS_at	9	0.858	1.171	0.801
Pf.5.312.0_CDS_at	1165	0.512	0.972	1.241
Pf.5.313.0_CDS_at	191	0.691	0.558	1.378
Pf.5.314.0_CDS_at	501	**2.403**	**0.188**	**0.303**
Pf.5.316.0_CDS_at	576	0.664	1.239	1.810
Pf.5.318.0_CDS_at	122	**3.483**	1.600	1.629
Pf.5.321.0_CDS_at	30	0.705	**0.299**	0.707
Pf.5.322.0_CDS_at	3573	**0.087**	**0.240**	**0.277**
Pf.5.324.0_CDS_at	7	0.694	0.773	0.769
Pf.5.326.0_CDS_at	58	**2.375**	1.827	1.086
Pf.5.327.0_CDS_at	303	1.421	1.574	1.744
Pf.5.328.0_CDS_at	6	1.210	0.954	0.952
Pf.5.328.0_CDS_x_at	7	0.989	0.845	1.018
Pf.5.329.0_CDS_at	5	0.995	1.024	1.119
Pf.5.329.0_CDS_x_at	4	1.277	1.124	1.218
Pf.5.33.0_CDS_at	2119	**0.416**	0.562	1.163
Pf.5.330.0_at	658	1.005	1.201	1.048
Pf.5.34.0_CDS_at	168	0.520	**0.462**	0.740
Pf.5.341.0_at	31	**0.459**	**0.345**	**2.094**
Pf.5.35.0_CDS_at	151	**0.283**	0.516	**0.490**
Pf.5.354.0_at	18	0.937	0.992	1.575
Pf.5.359.0_at	2917	0.521	0.892	0.884
Pf.5.36.0_CDS_at	6	1.191	0.951	1.024
Pf.5.360.0_at	1491	0.958	1.734	1.069
Pf.5.37.0_CDS_at	911	0.655	0.728	1.301
Pf.5.378.0_at	106	1.457	1.281	**4.097**
Pf.5.38.0_CDS_at	13	1.019	0.713	0.998
Pf.5.382.0_s_at	9	1.298	1.187	0.957
Pf.5.39.0_CDS_at	39	**0.488**	0.515	0.881
Pf.5.4.0_CDS_at	36	1.103	**0.337**	**0.362**
Pf.5.40.0_CDS_at	5755	1.025	0.889	1.199
Pf.5.40.0_UTR_at	2328	0.916	0.849	1.215

Pf.5.40.0_UTR_x_at	1162	0.847	0.873	1.228
Pf.5.405.0_at	377	1.205	0.981	1.578
Pf.5.41.0_CDS_at	660	**0.014**	**0.017**	**0.015**
Pf.5.41.0_CDS_x_at	1575	**0.014**	**0.015**	**0.015**
Pf.5.412.0_at	73	1.169	1.352	1.002
Pf.5.42.0_CDS_at	781	0.718	1.045	0.891
Pf.5.43.0_CDS_at	228	**0.292**	0.646	0.592
Pf.5.44.0_CDS_at	135	1.999	1.410	1.823
Pf.5.46.0_CDS_at	291	0.751	1.384	0.540
Pf.5.47.0_CDS_at	196	**0.063**	**0.095**	**0.069**
Pf.5.49.0_CDS_at	526	0.873	0.690	1.314
Pf.5.5.0_CDS_at	6932	0.989	0.560	0.958
Pf.5.50.0_CDS_at	3330	1.488	1.612	1.623
Pf.5.51.0_CDS_at	136	0.614	**0.428**	**2.664**
Pf.5.52.0_CDS_at	656	0.604	1.920	1.054
Pf.5.53.0_CDS_at	10751	0.630	1.034	1.105
Pf.5.54.0_CDS_at	15457	0.802	0.622	0.947
Pf.5.55.0_CDS_at	388	1.616	1.681	**2.231**
Pf.5.57.0_CDS_at	11015	1.080	0.825	1.154
Pf.5.58.0_CDS_at	2175	**2.216**	1.967	1.806
Pf.5.59.0_CDS_at	91	0.612	1.107	1.114
Pf.5.6.0_CDS_at	1960	0.967	0.980	0.870
Pf.5.60.0_CDS_at	27	0.867	1.034	1.978
Pf.5.61.0_CDS_at	4616	1.162	0.891	1.261
Pf.5.62.0_CDS_at	1471	1.630	1.614	1.585
Pf.5.63.0_CDS_at	445	0.928	0.744	0.648
Pf.5.64.0_CDS_a_at	29	**2.088**	0.551	**0.419**
Pf.5.65.0_CDS_at	358	**0.218**	**0.416**	**0.302**
Pf.5.66.0_CDS_at	444	**0.268**	0.956	0.697
Pf.5.67.0_CDS_at	241	0.662	1.009	**0.191**
Pf.5.69.0_CDS_at	21	1.512	1.425	**2.835**
Pf.5.7.0_CDS_a_at	1557	0.967	1.124	1.307
Pf.5.70.0_CDS_at	9	0.657	0.772	1.280
Pf.5.71.0_CDS_at	317	1.132	1.146	1.326
Pf.5.74.0_CDS_at	28	0.764	0.626	1.114
Pf.5.75.0_CDS_at	27	**2.324**	**3.569**	**2.523**
Pf.5.76.0_CDS_at	36	**0.443**	0.696	1.492
Pf.5.77.0_CDS_at	352	**0.475**	0.834	0.868
Pf.5.78.0_CDS_a_at	11	1.505	1.006	1.083
Pf.5.79.0_CDS_at	131	**0.190**	0.531	0.759
Pf.5.8.0_CDS_at	6157	**0.187**	**0.142**	**0.067**
Pf.5.81.0_CDS_at	790	0.555	0.735	0.625
Pf.5.82.0_CDS_at	860	1.486	1.273	1.614
Pf.5.83.0_CDS_a_at	815	**4.486**	**2.469**	1.734
Pf.5.84.0_CDS_at	449	1.616	1.155	0.740
Pf.5.85.0_CDS_at	504	**0.270**	**0.498**	**0.053**
Pf.5.86.0_CDS_at	718	1.637	**2.301**	1.601
Pf.5.87.0_CDS_at	241	1.161	1.907	**2.414**
Pf.5.9.0_CDS_at	2439	**0.050**	**0.080**	**0.024**
Pf.5.90.0_CDS_at	7	1.028	0.778	1.365
Pf.5.91.0_CDS_at	69	1.337	**0.152**	**0.192**
Pf.5.92.0_CDS_at	14	0.942	0.644	0.743
Pf.5.93.0_CDS_a_at	60	1.087	**0.310**	0.598
Pf.5.94.0_CDS_at	5306	1.526	1.263	1.481
Pf.5.96.0_CDS_at	434	1.031	0.935	0.627
Pf.5.97.0_CDS_at	242	0.697	0.814	1.224
Pf.5.99.0_CDS_at	14	0.853	0.714	0.711
Pf.6.1.0_CDS_at	151	0.654	0.637	**0.289**
Pf.6.10.0_CDS_at	1315	1.720	1.336	1.502
Pf.6.100.0_CDS_at	866	0.942	1.475	1.782
Pf.6.100.0_CDS_x_at	828	0.839	1.323	1.806
Pf.6.101.0_CDS_x_at	804	**0.150**	**0.070**	**0.227**

Pf.6.101.1_a_at	817	**0.074**	**0.098**	**0.283**
Pf.6.103.0_CDS_at	321	1.290	1.980	1.458
Pf.6.104.0_CDS_at	147	0.834	1.139	1.563
Pf.6.106.0_CDS_at	7608	**2.028**	1.737	1.679
Pf.6.107.0_CDS_at	520	0.915	1.633	1.330
Pf.6.109.0_CDS_at	35	1.536	**4.210**	0.533
Pf.6.11.0_CDS_at	226	**0.392**	**0.262**	**0.225**
Pf.6.110.0_CDS_a_at	147	**2.953**	**2.864**	1.556
Pf.6.111.0_CDS_at	654	1.373	1.804	1.917
Pf.6.112.0_CDS_at	2066	0.694	0.698	1.233
Pf.6.114.0_CDS_at	296	0.669	**0.369**	1.164
Pf.6.115.0_CDS_at	3796	1.549	1.797	1.920
Pf.6.116.0_CDS_at	25	0.750	0.658	1.461
Pf.6.117.0_CDS_at	25	0.579	**0.462**	1.944
Pf.6.118.0_CDS_at	25	1.552	0.789	0.705
Pf.6.119.0_CDS_at	761	0.783	1.102	0.960
Pf.6.12.0_CDS_at	3855	0.544	0.862	1.048
Pf.6.122.0_CDS_at	7	1.189	0.702	0.923
Pf.6.124.0_CDS_at	10211	**0.491**	0.811	1.202
Pf.6.125.0_CDS_at	128	1.379	1.470	1.077
Pf.6.127.0_CDS_at	54	0.818	1.021	1.655
Pf.6.129.0_CDS_at	1042	0.807	0.756	0.820
Pf.6.130.0_CDS_at	590	0.779	1.464	**3.194**
Pf.6.131.0_CDS_at	387	**2.303**	**2.668**	**2.963**
Pf.6.132.0_CDS_at	3837	1.071	1.138	1.195
Pf.6.134.0_CDS_at	70	0.684	**0.428**	**0.166**
Pf.6.135.0_CDS_at	90	0.603	**0.452**	1.910
Pf.6.136.0_CDS_at	1318	**0.395**	**0.192**	0.571
Pf.6.138.0_CDS_at	10	1.611	1.189	1.184
Pf.6.14.0_CDS_at	308	0.592	1.052	1.627
Pf.6.141.0_CDS_at	466	**0.165**	**0.542**	0.961
Pf.6.144.0_CDS_at	207	1.035	0.688	0.578
Pf.6.145.0_CDS_at	9	0.834	0.782	1.172
Pf.6.146.0_CDS_at	592	**0.196**	**0.466**	**0.380**
Pf.6.147.0_CDS_at	1202	0.912	1.048	1.309
Pf.6.148.0_CDS_at	11	0.717	0.871	1.168
Pf.6.149.0_CDS_at	99	**0.280**	**0.278**	**0.291**
Pf.6.15.0_CDS_at	26	0.517	1.005	**0.380**
Pf.6.150.0_CDS_at	290	0.551	0.726	1.826
Pf.6.151.0_CDS_at	53	**0.309**	0.947	1.276
Pf.6.152.0_CDS_at	79	0.726	1.899	1.880
Pf.6.153.0_CDS_at	180	**0.492**	0.563	0.884
Pf.6.154.0_CDS_at	633	0.773	1.617	**2.017**
Pf.6.155.0_CDS_at	9	0.980	1.409	1.421
Pf.6.157.0_CDS_at	81	**0.469**	1.277	**2.809**
Pf.6.158.0_CDS_at	81	0.608	0.930	**2.162**
Pf.6.159.0_CDS_at	461	0.850	1.033	0.808
Pf.6.16.0_CDS_at	10355	0.837	1.074	0.862
Pf.6.160.0_CDS_at	281	0.872	1.128	**2.034**
Pf.6.161.0_CDS_at	59	0.702	0.729	1.728
Pf.6.162.0_CDS_at	618	0.809	1.817	1.658
Pf.6.163.0_CDS_a_at	4117	**0.247**	**0.212**	**0.421**
Pf.6.163.0_CDS_at	8528	**0.235**	**0.218**	**0.362**
Pf.6.164.0_CDS_at	94	0.711	1.537	1.933
Pf.6.165.0_CDS_a_at	32	1.684	0.637	0.540
Pf.6.167.0_CDS_x_at	10	1.447	1.186	0.917
Pf.6.169.0_CDS_at	6	0.945	1.126	1.021
Pf.6.17.0_CDS_at	11	0.846	0.651	0.560
Pf.6.170.0_CDS_at	7	0.895	1.071	0.921
Pf.6.173.0_CDS_s_at	10	1.246	0.701	0.664
Pf.6.174.0_CDS_s_at	5	1.019	0.884	0.901
Pf.6.174.0_CDS_x_at	6	1.260	1.376	0.984

Pf.6.175.0_CDS_at	186	**2.208**	**2.510**	**2.076**
Pf.6.177.0_CDS_at	958	**0.387**	**0.249**	**0.173**
Pf.6.178.0_CDS_at	16	0.695	1.533	0.941
Pf.6.179.0_CDS_at	181	0.630	0.563	1.566
Pf.6.18.0_CDS_at	1045	1.083	1.064	1.420
Pf.6.180.0_CDS_at	30	0.531	**0.326**	1.004
Pf.6.181.0_CDS_at	133	0.948	**0.458**	**2.895**
Pf.6.182.0_CDS_at	116	0.510	0.674	**0.345**
Pf.6.183.0_CDS_at	63	1.014	0.624	0.523
Pf.6.185.0_CDS_at	45	1.717	1.985	0.920
Pf.6.186.0_CDS_at	127	1.265	1.346	**0.367**
Pf.6.187.0_CDS_at	67	**0.200**	**0.336**	1.040
Pf.6.189.0_CDS_at	1021	0.939	1.184	1.272
Pf.6.19.0_CDS_at	109	0.747	0.973	0.794
Pf.6.191.0_CDS_at	8	0.982	0.752	0.827
Pf.6.192.0_CDS_at	69	**4.278**	**2.075**	1.759
Pf.6.193.0_CDS_at	412	**0.443**	0.614	0.718
Pf.6.194.0_CDS_at	791	1.355	1.834	1.214
Pf.6.195.0_CDS_at	29	0.693	0.727	1.072
Pf.6.197.0_CDS_at	119	0.726	1.033	1.527
Pf.6.198.0_CDS_at	571	**0.208**	**0.324**	**0.467**
Pf.6.2.0_CDS_at	2284	1.212	1.633	1.198
Pf.6.20.0_CDS_at	194	**0.400**	**0.301**	**0.437**
Pf.6.201.0_CDS_at	457	**3.448**	**2.030**	1.436
Pf.6.202.1_at	18	0.814	0.645	0.516
Pf.6.203.0_CDS_at	19	0.958	1.108	1.956
Pf.6.206.0_CDS_at	2555	0.679	1.190	1.321
Pf.6.207.0_CDS_at	131	0.702	0.734	**0.451**
Pf.6.21.0_CDS_at	182	1.157	0.888	**2.187**
Pf.6.211.0_CDS_at	98	1.253	0.701	0.822
Pf.6.212.0_CDS_at	14	0.962	1.321	1.644
Pf.6.213.0_CDS_at	25	0.920	**0.331**	**7.131**
Pf.6.214.0_CDS_at	22	1.786	0.694	1.722
Pf.6.215.0_CDS_at	9	0.937	1.008	1.781
Pf.6.216.0_CDS_at	252	0.961	1.038	1.036
Pf.6.219.0_CDS_at	14	1.313	**0.449**	**0.396**
Pf.6.22.0_CDS_at	397	**3.190**	**3.369**	**2.575**
Pf.6.221.0_CDS_at	179	0.713	1.847	**2.314**
Pf.6.222.0_CDS_at	104	**2.106**	**2.923**	1.793
Pf.6.223.0_CDS_at	6	0.850	0.888	0.908
Pf.6.224.0_CDS_at	16	0.763	0.686	0.870
Pf.6.225.0_CDS_at	152	1.206	**2.800**	**3.162**
Pf.6.227.0_CDS_at	48	0.734	0.764	**2.581**
Pf.6.228.0_CDS_at	34	**0.476**	1.023	**2.557**
Pf.6.229.0_CDS_at	45	1.634	**0.326**	**0.458**
Pf.6.23.0_CDS_a_at	14623	0.976	0.857	0.818
Pf.6.230.0_CDS_at	619	**0.492**	**0.246**	1.271
Pf.6.231.0_CDS_at	29	**0.326**	**0.321**	**0.314**
Pf.6.232.0_CDS_at	66	1.547	**2.392**	1.648
Pf.6.232.0_CDS_x_at	6	1.406	1.931	1.659
Pf.6.233.0_CDS_at	35	0.987	0.532	1.234
Pf.6.234.0_CDS_a_at	10	0.854	0.796	0.897
Pf.6.235.0_CDS_at	55	0.899	**2.304**	**4.578**
Pf.6.235.0_CDS_x_at	46	0.515	1.720	**3.599**
Pf.6.238.0_CDS_s_at	7	**5.073**	**2.003**	1.696
Pf.6.239.0_CDS_s_at	18	1.212	0.637	0.785
Pf.6.239.0_CDS_x_at	7	0.874	0.978	0.795
Pf.6.24.0_CDS_at	1061	1.633	**2.166**	1.491
Pf.6.24.0_UTR_at	531	1.650	1.983	1.406
Pf.6.240.0_CDS_at	5	0.892	0.935	0.911
Pf.6.241.0_CDS_x_at	7	0.976	0.931	0.822
Pf.6.242.0_CDS_s_at	8	1.106	0.938	0.686

Pf.6.243.0_CDS_at	6	1.081	1.281	0.829
Pf.6.244.0_CDS_at	6	0.992	0.858	0.828
Pf.6.244.0_CDS_x_at	6	1.053	0.941	1.065
Pf.6.245.0_CDS_at	8	0.881	1.096	1.147
Pf.6.246.0_CDS_at	6	1.189	1.054	1.033
Pf.6.246.0_CDS_x_at	6	1.520	1.212	1.156
Pf.6.247.0_CDS_at	7	1.376	0.991	0.976
Pf.6.248.0_CDS_at	6	1.068	0.887	0.828
Pf.6.249.0_CDS_at	5	0.944	0.986	0.930
Pf.6.25.0_CDS_at	204	0.710	**0.351**	**0.385**
Pf.6.250.0_CDS_at	11	0.757	0.654	0.586
Pf.6.251.0_CDS_at	6	0.940	0.837	0.865
Pf.6.252.0_CDS_at	5	1.052	1.111	1.116
Pf.6.252.0_CDS_s_at	6	1.133	0.965	1.029
Pf.6.257.0_CDS_at	55	**0.272**	0.712	**3.082**
Pf.6.26.0_CDS_at	921	**3.726**	**2.689**	**2.643**
Pf.6.260.0_CDS_at	112	0.563	**0.471**	0.809
Pf.6.261.0_CDS_at	339	**0.417**	0.750	1.203
Pf.6.262.0_CDS_at	153	**2.247**	1.976	**2.883**
Pf.6.263.0_CDS_at	47	1.407	**2.858**	**3.434**
Pf.6.264.0_CDS_at	1747	**0.493**	0.612	0.778
Pf.6.265.0_CDS_at	34	0.673	0.802	1.314
Pf.6.266.0_CDS_at	10	0.666	0.697	1.133
Pf.6.267.0_CDS_at	166	0.888	1.325	0.907
Pf.6.268.0_CDS_at	1869	0.589	**0.269**	1.099
Pf.6.269.0_CDS_at	111	0.736	**0.336**	**0.423**
Pf.6.27.0_CDS_at	237	**0.202**	**0.356**	**0.112**
Pf.6.271.0_CDS_at	753	0.848	1.061	1.165
Pf.6.273.0_CDS_at	8	0.900	0.606	0.781
Pf.6.274.0_CDS_at	255	1.643	**3.164**	1.926
Pf.6.276.0_CDS_at	27	0.951	0.818	1.095
Pf.6.277.0_CDS_at	784	0.803	1.503	1.343
Pf.6.28.0_CDS_a_at	2127	1.562	1.481	1.587
Pf.6.281.0_CDS_at	11	0.746	0.662	0.997
Pf.6.282.0_CDS_at	2199	0.566	0.501	1.048
Pf.6.284.0_CDS_at	45	0.634	1.084	0.977
Pf.6.285.0_CDS_at	7	0.798	0.735	0.923
Pf.6.286.0_CDS_at	1362	1.307	**2.271**	1.773
Pf.6.287.0_CDS_at	17	1.682	0.735	0.776
Pf.6.287.0_CDS_x_at	6	1.221	0.963	0.781
Pf.6.288.0_CDS_at	7	1.121	1.018	1.033
Pf.6.289.0_CDS_at	6	0.964	1.200	1.192
Pf.6.290.0_CDS_at	12	0.631	0.633	**0.472**
Pf.6.291.0_CDS_at	57	0.622	**0.410**	0.765
Pf.6.292.0_CDS_at	8	0.767	0.872	0.999
Pf.6.293.0_CDS_at	6	1.671	1.478	1.146
Pf.6.295.0_CDS_at	168	**0.156**	**0.165**	**0.134**
Pf.6.296.0_CDS_at	19	1.861	1.594	0.680
Pf.6.297.0_CDS_at	478	1.134	**0.378**	0.548
Pf.6.298.0_CDS_at	77	**0.144**	**0.139**	1.343
Pf.6.299.0_CDS_at	430	**0.163**	**0.161**	**0.188**
Pf.6.3.0_CDS_at	53	1.212	0.760	1.000
Pf.6.300.0_CDS_at	219	0.548	0.704	0.931
Pf.6.301.0_CDS_at	920	**2.444**	**2.047**	1.441
Pf.6.302.0_CDS_at	15	0.838	0.616	0.824
Pf.6.303.0_CDS_at	256	**0.355**	0.502	0.681
Pf.6.305.0_CDS_at	7	1.242	1.204	1.986
Pf.6.307.0_CDS_s_at	80	0.775	1.026	**5.229**
Pf.6.31.0_CDS_at	1443	1.394	1.324	1.197
Pf.6.310.0_CDS_a_at	37	0.685	**0.355**	**0.402**
Pf.6.312.0_CDS_at	8	0.711	0.785	1.660
Pf.6.314.0_CDS_at	138	**0.399**	**0.479**	0.515

Pf.6.316.0_CDS_at	315	1.366	**2.350**	**2.704**
Pf.6.317.0_CDS_at	8	0.971	0.787	1.182
Pf.6.318.0_CDS_x_at	52	1.685	1.553	1.985
Pf.6.319.0_at	31	0.955	1.182	1.473
Pf.6.32.0_CDS_at	122	0.856	1.283	**2.866**
Pf.6.320.0_at	135	0.881	0.603	0.754
Pf.6.328.0_at	218	1.395	1.524	1.585
Pf.6.33.0_CDS_at	1150	1.271	1.818	1.215
Pf.6.34.0_CDS_at	408	**0.496**	0.787	1.006
Pf.6.35.0_CDS_at	172	0.567	0.701	0.833
Pf.6.358.0_at	551	1.540	1.414	**2.077**
Pf.6.36.0_CDS_at	63	0.771	0.709	1.429
Pf.6.37.0_CDS_at	2225	1.098	1.622	1.166
Pf.6.374.0_at	9	1.210	1.401	1.279
Pf.6.379.0_at	45	0.989	**0.335**	0.878
Pf.6.383.0_at	10	0.927	0.687	0.692
Pf.6.39.0_CDS_at	2625	0.696	0.644	1.234
Pf.6.40.0_CDS_at	590	**0.115**	**0.160**	**0.082**
Pf.6.41.0_CDS_at	3593	0.786	0.773	0.989
Pf.6.42.0_CDS_at	6884	1.600	1.541	1.309
Pf.6.43.0_CDS_at	98	0.553	1.256	1.145
Pf.6.44.0_CDS_at	25	0.875	0.676	0.832
Pf.6.45.0_CDS_at	1094	1.835	0.693	**0.342**
Pf.6.46.0_CDS_at	1695	1.143	1.349	1.610
Pf.6.47.0_CDS_at	15	0.753	**0.422**	0.521
Pf.6.49.0_CDS_at	413	1.302	1.902	**2.144**
Pf.6.50.0_CDS_at	50	0.567	0.714	1.002
Pf.6.51.0_CDS_at	3404	1.122	1.623	1.358
Pf.6.52.0_CDS_at	245	1.095	1.547	**0.413**
Pf.6.53.0_CDS_at	608	0.726	0.705	0.937
Pf.6.54.0_CDS_at	102	**0.384**	**0.393**	**0.305**
Pf.6.55.0_CDS_at	373	0.785	0.966	0.937
Pf.6.56.0_CDS_at	375	0.566	1.104	**2.441**
Pf.6.57.0_CDS_at	223	1.177	1.761	1.692
Pf.6.58.0_CDS_at	203	0.819	0.738	0.718
Pf.6.59.0_CDS_s_at	270	1.090	0.596	**0.289**
Pf.6.6.0_CDS_at	921	1.279	1.002	1.093
Pf.6.60.0_CDS_at	8	0.893	0.723	0.758
Pf.6.61.0_CDS_at	529	1.543	**2.053**	1.637
Pf.6.62.0_CDS_at	784	**2.630**	**2.053**	1.704
Pf.6.64.0_CDS_at	235	1.597	1.940	**2.367**
Pf.6.66.0_CDS_at	301	0.768	**0.324**	**0.182**
Pf.6.67.0_CDS_at	431	**0.471**	0.596	1.170
Pf.6.68.0_CDS_at	195	0.612	0.747	1.731
Pf.6.69.0_CDS_at	7	**4.007**	1.103	0.789
Pf.6.71.0_CDS_at	29	0.905	0.655	**0.482**
Pf.6.72.0_CDS_a_at	9	1.100	0.702	0.820
Pf.6.74.0_CDS_at	16	**0.374**	**0.300**	**0.322**
Pf.6.75.0_CDS_at	277	0.661	0.687	0.765
Pf.6.77.0_CDS_at	125	1.358	**2.309**	**2.030**
Pf.6.79.0_CDS_at	724	1.536	1.512	1.597
Pf.6.8.0_CDS_at	721	**0.497**	**0.343**	**0.485**
Pf.6.80.0_CDS_at	306	1.813	1.411	1.830
Pf.6.81.0_CDS_at	35	0.947	0.891	1.485
Pf.6.82.0_CDS_at	177	1.924	1.449	1.352
Pf.6.83.0_CDS_at	7	1.745	1.037	1.144
Pf.6.84.0_CDS_at	112	0.600	1.190	1.288
Pf.6.85.0_CDS_at	148	1.318	1.062	1.013
Pf.6.86.0_CDS_at	660	**0.486**	1.046	0.695
Pf.6.87.0_CDS_at	120	**0.086**	**0.159**	**0.088**
Pf.6.88.0_CDS_at	166	**0.416**	0.726	1.193
Pf.6.89.0_CDS_at	178	0.683	1.358	**2.335**

Pf.6.9.0_CDS_at	269	1.002	1.048	1.580
Pf.6.90.0_CDS_at	256	**2.128**	**2.303**	**2.379**
Pf.6.91.0_CDS_at	144	**0.485**	**0.445**	**2.676**
Pf.6.92.0_CDS_at	2111	**0.373**	0.720	1.012
Pf.6.93.0_CDS_at	90	**0.405**	0.608	0.817
Pf.6.95.0_CDS_at	6	0.895	0.881	0.940
Pf.6.95.0_CDS_x_at	10	0.681	0.739	1.101
Pf.6.97.0_CDS_at	566	**0.224**	0.527	**0.156**
Pf.6.98.0_CDS_at	134	**0.274**	**0.426**	**0.316**
Pf.6.99.0_CDS_at	15	1.724	1.504	**2.641**
Pf.7.1.0_CDS_x_at	1721	**2.109**	**2.205**	1.914
Pf.7.1.0_UTR_a_at	5	1.392	1.300	1.123
Pf.7.1.1_CDS_a_at	6	1.078	1.044	0.849
Pf.7.1.2_CDS_s_at	4403	1.688	1.678	0.799
Pf.7.1.2_UTR_a_at	5	1.315	1.191	1.139
Pf.7.10.0_CDS_at	1665	0.578	0.842	1.203
Pf.7.100.0_CDS_at	3575	0.538	0.615	1.135
Pf.7.102.0_CDS_at	69	0.670	0.766	1.436
Pf.7.103.0_CDS_at	58	0.541	1.227	1.088
Pf.7.105.0_CDS_at	172	1.973	**3.013**	**2.869**
Pf.7.106.0_CDS_at	54	0.589	1.343	**2.926**
Pf.7.108.0_CDS_at	40	1.228	0.642	1.047
Pf.7.109.0_CDS_at	75	0.872	1.222	1.808
Pf.7.110.0_CDS_at	215	0.501	0.523	**0.396**
Pf.7.111.0_CDS_at	217	1.140	**0.151**	**0.263**
Pf.7.112.0_CDS_at	134	**0.453**	1.054	1.712
Pf.7.113.0_CDS_at	485	1.898	0.632	1.615
Pf.7.114.0_CDS_at	1049	1.056	1.058	1.607
Pf.7.116.0_CDS_at	1164	1.075	**2.006**	1.071
Pf.7.118.0_CDS_at	1890	**0.496**	0.779	0.903
Pf.7.119.0_CDS_at	381	**0.451**	**0.350**	**0.470**
Pf.7.120.0_CDS_at	263	**2.154**	1.463	**2.424**
Pf.7.123.0_CDS_at	23	1.583	**2.429**	1.443
Pf.7.124.0_CDS_at	449	1.716	1.395	**2.118**
Pf.7.125.0_CDS_at	199	1.714	1.487	**2.607**
Pf.7.127.0_CDS_at	7291	0.582	0.750	0.952
Pf.7.129.0_CDS_at	707	0.852	0.976	1.885
Pf.7.13.0_CDS_at	67	**0.244**	**0.296**	**0.175**
Pf.7.130.0_CDS_at	353	**0.187**	0.731	**0.384**
Pf.7.131.0_CDS_at	575	1.261	1.939	1.887
Pf.7.132.0_CDS_at	134	**0.286**	0.594	1.477
Pf.7.133.0_CDS_at	646	**0.348**	**0.405**	1.413
Pf.7.135.0_CDS_x_at	40	0.898	0.755	0.670
Pf.7.136.0_CDS_at	5	1.134	1.052	1.172
Pf.7.137.0_CDS_at	14	0.790	0.633	0.536
Pf.7.138.0_CDS_at	340	1.277	1.390	1.697
Pf.7.139.0_CDS_at	287	0.507	**0.497**	**0.382**
Pf.7.139.0_CDS_x_at	379	0.520	0.517	**0.357**
Pf.7.14.0_CDS_at	4247	1.438	1.079	1.221
Pf.7.140.0_CDS_at	478	0.689	1.291	1.257
Pf.7.141.0_CDS_at	2196	0.955	1.458	1.697
Pf.7.142.0_CDS_at	10	0.705	0.503	**0.496**
Pf.7.143.0_CDS_at	275	1.676	1.484	1.627
Pf.7.144.0_CDS_at	283	1.007	0.630	**0.401**
Pf.7.145.0_CDS_at	208	**0.310**	0.500	**0.560**
Pf.7.146.0_CDS_at	418	**2.217**	1.844	1.676
Pf.7.147.0_CDS_at	14	1.982	**3.965**	0.639
Pf.7.148.0_CDS_at	5	1.117	0.918	0.918
Pf.7.148.0_CDS_x_at	5	0.966	0.874	0.881
Pf.7.15.0_CDS_at	42	0.802	0.869	1.364
Pf.7.151.0_CDS_at	116	**3.764**	1.036	1.070
Pf.7.152.0_CDS_at	40	**5.313**	**2.895**	**0.315**

Pf.7.153.0_CDS_at	6	1.792	1.816	2.292
Pf.7.153.0_CDS_x_at	9	1.393	1.274	1.379
Pf.7.154.0_CDS_at	11761	1.484	1.604	1.427
Pf.7.155.0_CDS_at	141	**2.876**	**2.053**	1.176
Pf.7.156.0_CDS_at	143	**3.139**	0.729	**0.499**
Pf.7.157.0_CDS_at	6	0.943	0.814	0.895
Pf.7.158.0_CDS_at	32	1.052	**0.363**	**0.474**
Pf.7.16.0_CDS_at	276	0.538	1.002	0.900
Pf.7.162.0_CDS_at	89	**4.280**	**2.626**	**2.007**
Pf.7.164.0_CDS_at	74	1.558	1.240	0.944
Pf.7.165.0_CDS_at	1012	1.090	1.619	1.554
Pf.7.166.0_CDS_at	430	1.189	0.536	0.653
Pf.7.167.0_CDS_at	19	1.225	1.202	**0.404**
Pf.7.168.0_CDS_at	8	0.910	1.058	0.892
Pf.7.169.0_CDS_x_at	8	**2.654**	1.216	0.994
Pf.7.17.0_CDS_at	437	1.407	1.306	0.990
Pf.7.170.0_CDS_at	5	1.028	1.061	1.121
Pf.7.171.0_CDS_at	7	1.371	1.252	1.000
Pf.7.171.0_CDS_x_at	9	0.956	1.027	0.888
Pf.7.172.0_CDS_at	490	**0.255**	**0.408**	1.111
Pf.7.174.0_CDS_at	20	1.012	0.923	1.265
Pf.7.176.0_CDS_at	197	**2.064**	**0.358**	**0.487**
Pf.7.177.0_CDS_x_at	11	1.223	0.535	0.767
Pf.7.178.0_CDS_at	177	0.750	0.741	**0.480**
Pf.7.179.0_CDS_at	60	0.881	0.995	1.193
Pf.7.18.0_CDS_at	637	**2.018**	**2.047**	1.466
Pf.7.180.0_CDS_at	94	**0.489**	**0.279**	**0.489**
Pf.7.181.0_CDS_at	1999	0.778	0.525	1.212
Pf.7.182.0_CDS_at	159	1.459	**2.449**	**2.794**
Pf.7.184.0_CDS_at	7	1.045	0.869	0.976
Pf.7.185.0_CDS_at	13	1.382	0.691	0.969
Pf.7.186.0_CDS_at	4034	0.558	0.829	1.397
Pf.7.187.0_CDS_at	459	0.530	0.804	1.909
Pf.7.188.0_CDS_at	121	1.309	1.700	**2.120**
Pf.7.189.0_CDS_at	171	0.688	1.531	1.160
Pf.7.191.0_CDS_at	324	0.559	0.638	1.715
Pf.7.194.0_CDS_at	21	1.181	1.307	1.680
Pf.7.195.0_CDS_at	10	1.010	1.763	1.238
Pf.7.196.0_CDS_at	12	1.172	0.606	0.574
Pf.7.197.0_CDS_at	172	1.101	1.330	**2.549**
Pf.7.198.0_CDS_at	141	1.348	0.726	0.855
Pf.7.199.0_CDS_at	84	0.590	**0.308**	0.696
Pf.7.2.0_CDS_s_at	12069	1.293	1.397	1.151
Pf.7.20.0_CDS_at	45	1.005	0.897	**0.224**
Pf.7.200.0_CDS_at	7	1.000	0.990	1.214
Pf.7.201.0_CDS_at	24	**3.016**	1.232	**2.610**
Pf.7.202.0_CDS_at	563	0.909	1.269	1.319
Pf.7.203.0_CDS_at	316	0.735	0.894	**2.505**
Pf.7.205.0_CDS_x_at	5	1.379	1.131	1.004
Pf.7.206.0_CDS_at	155	0.550	1.286	**2.415**
Pf.7.207.0_CDS_at	690	**0.319**	1.210	**2.220**
Pf.7.209.0_CDS_at	101	0.559	0.559	1.915
Pf.7.211.0_CDS_at	63	0.720	**0.312**	**0.201**
Pf.7.212.0_CDS_a_at	39	1.666	1.519	1.518
Pf.7.213.0_CDS_at	39	0.682	**0.242**	0.658
Pf.7.214.0_CDS_at	19	0.940	0.922	0.870
Pf.7.215.0_CDS_at	6	1.082	0.820	0.838
Pf.7.216.0_CDS_at	175	1.036	1.277	1.589
Pf.7.217.0_CDS_at	123	0.891	**0.169**	1.093
Pf.7.218.0_CDS_x_at	263	**3.267**	**0.435**	**0.431**
Pf.7.22.0_CDS_at	1689	**0.432**	**0.394**	**0.498**
Pf.7.220.0_CDS_at	836	1.259	0.967	1.242

Pf.7.221.0_CDS_at	48	0.632	0.515	1.054
Pf.7.221.0_CDS_x_at	497	0.578	**0.479**	0.853
Pf.7.222.0_CDS_at	11	1.406	**2.059**	1.197
Pf.7.223.0_CDS_at	6	1.041	1.143	1.053
Pf.7.224.0_CDS_s_at	383	1.619	1.544	**2.061**
Pf.7.225.0_CDS_at	6	1.078	0.967	1.114
Pf.7.226.0_CDS_s_at	6	0.976	1.024	0.897
Pf.7.228.0_CDS_at	6	0.840	0.963	0.906
Pf.7.229.0_CDS_x_at	4	1.300	1.350	1.022
Pf.7.23.0_CDS_at	589	**0.298**	**0.268**	0.759
Pf.7.230.0_CDS_at	53	**3.207**	0.987	0.956
Pf.7.231.0_CDS_s_at	40	1.936	**3.843**	**0.462**
Pf.7.232.0_CDS_at	6	1.765	1.333	0.829
Pf.7.233.0_CDS_at	5	1.245	0.988	1.017
Pf.7.234.0_CDS_at	115	**2.704**	**2.079**	1.508
Pf.7.234.0_CDS_x_at	314	**3.553**	**2.143**	1.684
Pf.7.235.0_CDS_at	8	1.176	1.039	1.078
Pf.7.237.0_CDS_at	15	1.254	1.120	1.730
Pf.7.238.0_CDS_at	737	1.040	1.064	**2.396**
Pf.7.24.0_CDS_at	297	1.050	**0.352**	**0.156**
Pf.7.240.0_CDS_at	12	1.128	1.088	0.695
Pf.7.241.0_CDS_at	46	**2.158**	1.207	**0.248**
Pf.7.242.0_CDS_at	15	**0.499**	0.862	0.809
Pf.7.243.0_CDS_at	553	1.708	1.734	1.728
Pf.7.244.0_CDS_at	737	**0.262**	**0.402**	1.484
Pf.7.245.0_CDS_at	2661	0.730	0.961	1.316
Pf.7.248.0_CDS_at	18	1.563	**2.616**	1.964
Pf.7.248.0_CDS_x_at	14	1.635	**2.666**	1.710
Pf.7.250.0_CDS_at	5	1.006	0.924	0.974
Pf.7.250.0_CDS_x_at	7	1.325	1.171	0.715
Pf.7.251.0_CDS_at	6	0.973	0.972	1.028
Pf.7.252.0_CDS_at	6	0.911	0.997	0.849
Pf.7.252.0_CDS_s_at	211	1.842	0.666	**0.492**
Pf.7.252.0_CDS_x_at	6	0.862	0.907	0.907
Pf.7.253.0_CDS_x_at	5	1.076	1.010	0.762
Pf.7.254.0_CDS_at	9	1.465	2.203	0.676
Pf.7.255.0_CDS_x_at	24	0.789	0.765	0.736
Pf.7.256.0_CDS_x_at	4	1.072	0.885	0.930
Pf.7.258.0_CDS_at	112	**0.219**	**0.338**	1.191
Pf.7.260.0_CDS_at	308	**2.578**	0.996	**0.490**
Pf.7.261.0_CDS_at	40	1.356	0.509	**0.346**
Pf.7.262.0_CDS_at	7	1.177	0.873	0.794
Pf.7.264.0_CDS_at	331	1.082	1.636	**2.447**
Pf.7.265.0_CDS_at	14	0.880	1.167	1.781
Pf.7.266.0_CDS_at	33	1.090	**0.294**	0.543
Pf.7.267.0_CDS_at	6	1.034	1.022	0.984
Pf.7.267.0_CDS_x_at	7	0.956	0.893	0.926
Pf.7.268.0_CDS_at	203	**0.394**	**0.184**	1.159
Pf.7.269.0_CDS_at	7	1.009	0.978	1.120
Pf.7.27.0_CDS_s_at	8063	1.122	1.245	1.340
Pf.7.271.0_CDS_at	50	1.714	**0.381**	0.772
Pf.7.272.0_CDS_at	11	1.372	0.585	1.262
Pf.7.274.0_CDS_at	106	1.922	**3.352**	**2.845**
Pf.7.275.0_CDS_at	13	**3.084**	1.731	**4.824**
Pf.7.276.0_CDS_at	15	0.835	1.661	**2.041**
Pf.7.277.0_CDS_at	975	0.685	0.554	1.188
Pf.7.279.0_CDS_at	135	0.502	1.611	**3.774**
Pf.7.28.0_CDS_at	279	0.530	1.356	**5.453**
Pf.7.281.0_CDS_at	16	0.762	**0.426**	0.623
Pf.7.283.0_CDS_at	789	0.707	1.583	1.476
Pf.7.284.0_CDS_at	86	1.082	1.047	1.289
Pf.7.285.0_CDS_at	1680	1.401	1.602	1.296

Pf.7.286.0_CDS_at	657	0.527	0.701	0.948
Pf.7.287.0_CDS_at	20	0.659	**0.432**	0.540
Pf.7.288.0_CDS_at	7	0.963	0.808	0.771
Pf.7.29.0_CDS_at	1511	1.448	1.086	0.908
Pf.7.290.0_CDS_at	384	**0.378**	**0.077**	**0.086**
Pf.7.292.0_CDS_at	6	1.055	1.053	0.998
Pf.7.293.0_CDS_at	8	0.786	0.800	0.738
Pf.7.294.0_CDS_at	6	0.931	0.854	0.956
Pf.7.3.0_CDS_at	322	**0.139**	0.528	**0.149**
Pf.7.30.0_CDS_at	71	1.801	0.837	0.830
Pf.7.31.0_CDS_a_at	16	1.531	1.020	0.968
Pf.7.310.0_at	24	1.045	0.946	1.270
Pf.7.32.0_CDS_at	332	**0.347**	**0.355**	0.893
Pf.7.329.0_at	6	1.222	1.732	1.377
Pf.7.332.0_at	122	1.153	1.373	1.505
Pf.7.335.0_at	671	**0.074**	**0.079**	**0.055**
Pf.7.36.0_CDS_at	765	**0.184**	**0.552**	**0.254**
Pf.7.36.0_CDS_s_at	3567	**0.255**	0.546	**0.270**
Pf.7.37.0_CDS_at	4971	**2.165**	1.988	1.673
Pf.7.38.0_CDS_at	305	**5.414**	**3.732**	**2.745**
Pf.7.39.0_CDS_at	215	**0.478**	**0.197**	**0.327**
Pf.7.4.0_CDS_at	1934	**0.201**	**0.338**	0.724
Pf.7.40.0_CDS_at	398	0.630	0.502	**0.398**
Pf.7.41.0_CDS_at	886	**2.106**	**2.373**	1.921
Pf.7.42.0_CDS_at	14	0.908	1.143	1.305
Pf.7.44.0_CDS_at	379	0.678	1.009	0.885
Pf.7.5.0_CDS_at	4879	0.822	0.767	0.904
Pf.7.5.0_CDS_x_at	3452	0.852	0.771	0.924
Pf.7.50.0_CDS_at	7	0.874	0.739	0.795
Pf.7.50.0_CDS_x_at	6	1.079	0.934	1.014
Pf.7.51.0_CDS_at	426	**2.006**	1.502	1.335
Pf.7.52.0_CDS_at	373	**0.390**	0.914	1.427
Pf.7.53.0_CDS_at	40	**0.382**	**0.324**	0.741
Pf.7.54.0_CDS_at	335	1.455	1.348	1.500
Pf.7.55.0_CDS_at	107	1.683	1.411	1.826
Pf.7.55.0_CDS_x_at	205	1.536	1.523	1.662
Pf.7.55.0_UTR_at	910	1.213	1.321	1.277
Pf.7.56.0_CDS_at	108	**0.128**	**0.112**	**0.191**
Pf.7.58.0_CDS_at	282	**4.157**	**4.195**	**4.056**
Pf.7.59.0_CDS_at	455	**4.259**	**4.397**	**3.926**
Pf.7.60.0_CDS_at	459	0.725	0.643	0.537
Pf.7.61.0_CDS_at	747	**0.429**	**0.401**	0.734
Pf.7.62.0_CDS_at	11	1.072	0.934	0.854
Pf.7.63.0_CDS_at	1191	**3.733**	**2.565**	**2.385**
Pf.7.64.0_CDS_a_at	2669	**0.268**	**0.220**	**0.139**
Pf.7.64.0_CDS_s_at	705	**0.262**	**0.199**	**0.138**
Pf.7.65.0_CDS_at	24	1.084	0.945	1.418
Pf.7.66.0_CDS_at	240	**5.809**	**6.765**	**2.762**
Pf.7.67.0_CDS_at	819	0.882	1.360	0.734
Pf.7.68.0_CDS_a_at	56	0.733	0.761	0.962
Pf.7.69.0_CDS_at	66	**0.365**	**0.335**	0.678
Pf.7.70.0_CDS_at	85	0.691	0.901	1.503
Pf.7.71.0_CDS_at	262	0.739	0.604	0.568
Pf.7.73.0_CDS_at	723	1.862	1.923	**2.163**
Pf.7.76.0_CDS_at	329	1.328	**2.081**	1.706
Pf.7.77.0_CDS_at	68	1.627	1.762	**2.590**
Pf.7.78.0_CDS_at	149	0.780	0.781	1.864
Pf.7.79.0_CDS_at	859	**2.218**	**2.167**	1.499
Pf.7.8.0_CDS_at	1656	0.815	0.773	0.917
Pf.7.80.0_CDS_a_at	120	**0.474**	0.549	1.474
Pf.7.80.1_a_at	135	**0.339**	0.589	1.229
Pf.7.81.0_CDS_at	849	0.741	0.825	1.313

Pf.7.83.0_CDS_at	105	**4.302**	1.144	0.840	
Pf.7.84.0_CDS_at	29	0.846	0.692	0.826	
Pf.7.85.0_CDS_at	69	1.307	1.216	**2.161**	
Pf.7.86.0_CDS_at	3784	**2.037**	**2.016**	1.693	
Pf.7.86.0_CDS_x_at	3192	1.948	1.954	1.746	
Pf.7.87.0_CDS_at	7533	1.442	1.381	1.370	
Pf.7.9.0_CDS_at	12	0.834	**0.489**	0.577	
Pf.7.90.0_CDS_at	9	1.023	0.705	1.049	
Pf.7.91.0_CDS_at	28	0.873	1.296	0.897	
Pf.7.92.0_CDS_at	392	0.831	1.060	0.660	
Pf.7.93.0_CDS_at	6	0.835	0.884	0.933	
Pf.7.94.0_CDS_at	433	**0.366**	**0.444**	**0.497**	
Pf.7.95.0_CDS_at	305	0.607	1.356	1.456	
Pf.7.96.0_CDS_at	29	1.144	1.207	0.942	
Pf.7.97.0_CDS_at	329	**0.326**	0.932	0.632	
Pf.7.98.0_CDS_at	1896	1.826	1.137	1.377	
Pf.8.1.0_CDS_a_at	2325	1.529	1.170	1.124	
Pf.8.10.0_CDS_at	282	**0.276**	0.546	**0.343**	
Pf.8.100.0_CDS_at	38	1.087	0.664	**0.390**	
Pf.8.101.0_CDS_at	640	**0.123**	**0.094**	**0.112**	
Pf.8.102.0_CDS_at	249	0.607	0.504	0.924	
Pf.8.104.0_CDS_at	7	1.045	0.695	0.807	
Pf.8.105.0_CDS_at	37	0.978	0.776	0.682	
Pf.8.106.0_CDS_at	206	1.570	0.587	0.826	
Pf.8.107.0_CDS_at	462	1.469	1.175	1.874	
Pf.8.108.0_CDS_at	98	1.788	1.416	**2.102**	
Pf.8.109.0_CDS_at	1273	1.545	1.415	1.304	
Pf.8.110.0_CDS_at	68	1.299	0.690	0.751	
Pf.8.111.0_CDS_at	414	**4.974**	**3.354**	**3.041**	
Pf.8.113.0_CDS_at	17	1.615	1.018	0.629	
Pf.8.114.0_CDS_at	2548	0.860	1.043	1.532	
Pf.8.116.0_CDS_at	364	0.648	1.311	0.959	
Pf.8.118.0_CDS_at	20	0.519	**0.489**	0.801	
Pf.8.119.0_CDS_at	48	1.984	**2.231**	1.119	
Pf.8.121.0_CDS_at	6	1.070	0.921	1.005	
Pf.8.123.0_CDS_at	32	0.912	**0.442**	0.571	
Pf.8.124.0_CDS_at	897	**2.476**	**2.334**	**2.625**	
Pf.8.125.0_CDS_at	62	0.825	1.794	**4.794**	
Pf.8.126.0_CDS_at	62	**0.179**	**0.447**	0.959	#9
Pf.8.127.0_CDS_at	87	**3.008**	1.883	1.562	
Pf.8.128.0_CDS_at	24	1.047	1.628	1.392	
Pf.8.129.0_CDS_at	1419	1.688	**2.751**	**2.053**	
Pf.8.130.0_CDS_at	68	0.649	0.531	**0.363**	
Pf.8.131.0_CDS_at	698	**0.335**	1.027	1.355	
Pf.8.132.0_CDS_at	230	1.578	**2.240**	1.925	
Pf.8.135.0_CDS_at	4232	0.937	0.881	0.982	
Pf.8.136.0_CDS_a_at	38	0.545	0.614	**0.405**	
Pf.8.137.0_CDS_at	129	0.807	0.871	0.761	
Pf.8.138.0_CDS_at	414	1.306	1.556	**2.165**	
Pf.8.139.0_CDS_at	28	1.369	1.735	0.723	
Pf.8.14.0_CDS_at	298	**3.186**	**2.537**	1.310	
Pf.8.140.0_CDS_at	881	1.751	**2.033**	1.549	
Pf.8.141.0_CDS_at	59	1.300	**2.321**	**2.767**	
Pf.8.142.0_CDS_a_at	728	0.769	**0.258**	**0.258**	
Pf.8.142.0_CDS_x_at	94	0.645	**0.296**	**0.343**	
Pf.8.143.0_CDS_at	816	**2.062**	1.383	1.831	
Pf.8.144.0_CDS_at	11	1.482	0.820	0.801	
Pf.8.145.0_CDS_at	96	0.526	0.616	1.470	
Pf.8.146.0_CDS_at	174	0.652	0.897	**2.513**	
Pf.8.147.0_CDS_at	28	**0.481**	**0.221**	**0.383**	
Pf.8.148.0_CDS_at	7	1.063	1.414	2.213	
Pf.8.15.0_CDS_at	23	1.059	0.830	0.560	
Pf.8.150.0_CDS_at	77	1.200	0.967	1.446	

Pf.8.151.0_CDS_at	386	**0.405**	0.946	0.701
Pf.8.152.0_CDS_x_at	4	0.957	0.945	0.998
Pf.8.153.0_CDS_at	202	**4.642**	**3.177**	**3.706**
Pf.8.154.0_CDS_at	210	0.966	**2.092**	**0.330**
Pf.8.155.0_CDS_at	158	**2.439**	**2.825**	1.026
Pf.8.156.0_CDS_at	12	1.727	0.798	0.675
Pf.8.157.0_CDS_at	28	0.897	0.700	1.607
Pf.8.158.0_CDS_at	8	1.076	0.714	0.967
Pf.8.16.0_CDS_at	921	**0.409**	**0.442**	0.868
Pf.8.160.0_CDS_at	14	0.880	0.605	0.580
Pf.8.162.0_CDS_at	7408	**0.333**	**0.307**	0.792
Pf.8.163.0_CDS_at	69	1.251	0.596	0.987
Pf.8.166.0_CDS_at	16	1.977	0.836	1.754
Pf.8.167.0_CDS_at	22	**3.013**	**2.394**	1.154
Pf.8.17.0_CDS_at	38	**0.364**	**0.415**	**0.309**
Pf.8.170.0_CDS_at	30	1.164	0.606	0.657
Pf.8.171.0_CDS_at	12	**2.629**	0.935	1.091
Pf.8.172.0_CDS_at	34	1.169	1.025	**1.949**
Pf.8.173.0_CDS_at	16	0.656	0.850	**1.779**
Pf.8.174.0_CDS_at	28	0.970	**3.225**	**5.086**
Pf.8.175.0_CDS_at	12	0.990	0.856	1.087
Pf.8.177.0_CDS_at	14	0.698	0.701	1.025
Pf.8.178.0_CDS_at	114	1.251	1.171	**2.262**
Pf.8.179.0_CDS_at	22	0.845	1.305	**2.507**
Pf.8.18.0_CDS_at	362	1.608	1.281	1.172
Pf.8.180.0_CDS_at	121	**3.989**	**6.366**	**3.997**
Pf.8.181.0_CDS_at	680	**2.885**	**3.196**	**2.425**
Pf.8.182.0_CDS_at	320	**0.403**	**0.437**	1.065
Pf.8.183.0_CDS_at	153	1.269	1.101	1.211
Pf.8.184.0_CDS_at	499	0.891	1.081	1.221
Pf.8.186.0_CDS_at	876	0.861	1.046	1.829
Pf.8.187.0_CDS_at	104	0.953	0.791	0.630
Pf.8.189.0_CDS_at	57	**2.139**	**0.469**	**0.386**
Pf.8.19.0_CDS_a_at	58	0.501	**0.489**	**0.340**
Pf.8.191.0_CDS_at	8	1.401	1.696	1.772
Pf.8.191.0_CDS_x_at	7	1.096	1.176	1.308
Pf.8.194.0_CDS_at	34	1.837	1.001	1.424
Pf.8.196.0_CDS_at	19	1.588	0.938	0.975
Pf.8.197.0_CDS_at	12	1.026	0.937	1.076
Pf.8.198.0_CDS_at	22	0.646	0.885	0.949
Pf.8.199.0_CDS_at	58	1.225	1.468	**2.409**
Pf.8.20.0_CDS_at	384	**2.025**	1.658	1.334
Pf.8.200.0_CDS_at	684	**0.163**	**0.360**	0.508
Pf.8.201.0_CDS_at	28	0.835	0.610	1.551
Pf.8.202.0_CDS_at	103	1.991	1.600	1.921
Pf.8.203.0_CDS_at	103	1.580	0.533	0.629
Pf.8.205.0_CDS_at	16	0.939	**0.498**	0.665
Pf.8.206.0_CDS_at	68	1.857	1.992	**0.372**
Pf.8.207.0_CDS_at	971	**0.320**	**0.146**	**0.107**
Pf.8.208.0_CDS_at	115	**0.453**	0.600	0.632
Pf.8.21.0_CDS_at	986	0.820	0.825	1.319
Pf.8.210.0_CDS_at	157	1.176	1.572	1.835
Pf.8.211.0_CDS_at	39	**0.477**	**0.358**	**0.338**
Pf.8.212.0_CDS_at	282	0.884	1.266	1.407
Pf.8.213.0_CDS_at	16	1.009	1.349	1.208
Pf.8.216.0_CDS_at	854	**2.435**	**2.278**	1.902
Pf.8.217.0_CDS_at	27	1.137	0.800	0.732
Pf.8.219.0_CDS_at	7	0.867	0.675	0.810
Pf.8.22.0_CDS_at	8123	0.917	0.937	1.231
Pf.8.220.0_CDS_at	5	0.957	0.951	0.999
Pf.8.220.0_CDS_x_at	5	0.913	0.945	0.982
Pf.8.221.0_CDS_at	231	0.602	0.987	0.625

Pf.8.222.0_CDS_at	605	1.061	0.903	1.422
Pf.8.225.0_CDS_at	6	1.049	1.072	0.912
Pf.8.226.0_CDS_s_at	5	1.026	1.022	0.996
Pf.8.227.0_CDS_x_at	8	1.840	1.400	0.648
Pf.8.228.0_CDS_s_at	12	1.833	0.903	0.725
Pf.8.228.0_CDS_x_at	8	1.010	1.276	1.295
Pf.8.23.0_CDS_at	13	0.912	0.980	0.546
Pf.8.230.0_CDS_at	12	**3.885**	**2.978**	**2.659**
Pf.8.231.0_CDS_at	594	0.610	0.742	1.955
Pf.8.233.0_CDS_at	104	**2.007**	0.555	0.694
Pf.8.234.0_CDS_x_at	7	1.216	1.536	1.064
Pf.8.235.0_CDS_at	33	0.921	1.712	**0.329**
Pf.8.236.0_CDS_at	1103	1.064	1.117	1.499
Pf.8.237.1_a_at	13	1.620	0.858	0.987
Pf.8.238.0_CDS_at	16	0.887	1.031	0.754
Pf.8.239.0_CDS_at	41	1.011	1.603	1.521
Pf.8.24.0_CDS_at	826	1.365	1.993	1.153
Pf.8.240.0_CDS_at	72	0.929	1.170	1.500
Pf.8.243.0_CDS_at	19	**0.417**	**0.295**	**0.324**
Pf.8.244.0_CDS_at	347	1.710	1.772	1.673
Pf.8.246.0_CDS_at	176	**0.484**	0.730	1.295
Pf.8.247.0_CDS_at	89	1.080	1.509	1.197
Pf.8.248.0_CDS_at	563	**0.287**	**0.357**	0.733
Pf.8.250.0_CDS_at	42	**0.482**	**0.344**	**3.395**
Pf.8.251.0_CDS_at	195	**0.351**	0.767	0.671
Pf.8.252.0_CDS_at	1182	1.322	1.613	1.722
Pf.8.253.0_CDS_at	264	0.548	1.460	**2.304**
Pf.8.254.0_CDS_at	76	0.951	1.599	1.889
Pf.8.255.0_CDS_at	7	**12.025**	2.226	1.848
Pf.8.256.0_CDS_at	537	**0.453**	0.620	**0.272**
Pf.8.257.0_CDS_at	65	0.731	**0.246**	**0.155**
Pf.8.258.0_CDS_at	17	1.363	0.739	1.404
Pf.8.259.0_CDS_at	31	**0.446**	**0.416**	0.751
Pf.8.261.0_CDS_at	146	0.934	**2.104**	**4.010**
Pf.8.262.0_CDS_at	21	**2.633**	1.335	1.037
Pf.8.264.0_CDS_at	132	0.929	1.506	**2.681**
Pf.8.265.0_CDS_at	410	1.359	1.725	**2.143**
Pf.8.267.0_CDS_a_at	444	1.501	0.949	**0.180**
Pf.8.269.0_CDS_at	853	**0.310**	**0.235**	**0.313**
Pf.8.27.0_CDS_at	125	0.711	0.789	1.191
Pf.8.271.0_CDS_at	101	0.516	0.521	1.593
Pf.8.272.0_CDS_at	11	1.108	0.561	0.592
Pf.8.273.0_CDS_at	73	0.824	0.535	0.890
Pf.8.274.0_CDS_at	62	1.301	0.567	0.805
Pf.8.275.0_CDS_at	12	**2.910**	0.830	1.036
Pf.8.278.0_CDS_at	470	**0.291**	**0.272**	1.228
Pf.8.279.0_CDS_at	133	**0.421**	0.913	**2.725**
Pf.8.280.0_CDS_at	47	1.292	1.667	0.922
Pf.8.281.0_CDS_at	6	0.966	0.957	0.981
Pf.8.282.0_CDS_at	6	1.211	1.634	0.889
Pf.8.283.0_CDS_at	5	1.086	1.004	0.968
Pf.8.286.0_CDS_at	1155	1.312	1.811	1.690
Pf.8.287.0_CDS_at	348	0.773	0.908	1.557
Pf.8.288.0_CDS_at	300	1.050	1.005	1.605
Pf.8.29.0_CDS_at	7829	1.009	0.835	0.908
Pf.8.290.0_CDS_at	863	1.117	**0.427**	**0.161**
Pf.8.292.0_CDS_at	24	0.679	0.552	0.554
Pf.8.294.0_CDS_at	686	**2.051**	1.843	1.960
Pf.8.295.0_CDS_at	308	1.515	0.649	1.448
Pf.8.296.0_CDS_at	9	**3.548**	1.558	0.837
Pf.8.3.0_CDS_at	53	1.983	0.554	0.500
Pf.8.306.0_at	1049	0.647	1.573	**0.204**

Pf.8.31.0_CDS_at	3017	0.885	0.680	1.095
Pf.8.317.0_at	26	1.149	**0.483**	1.036
Pf.8.32.0_CDS_at	1739	**0.276**	**0.328**	**0.051**
Pf.8.33.0_CDS_at	1837	1.329	1.413	1.225
Pf.8.339.0_at	7	0.933	1.103	1.078
Pf.8.34.0_CDS_at	217	1.604	1.618	1.157
Pf.8.344.0_at	7	0.934	1.038	1.242
Pf.8.36.0_CDS_at	251	1.018	**2.087**	1.687
Pf.8.368.0_at	3161	0.891	**0.275**	1.178
Pf.8.37.0_CDS_at	15455	0.557	0.551	0.794
Pf.8.375.0_at	162	1.095	1.072	1.288
Pf.8.38.0_CDS_at	766	0.886	1.142	1.276
Pf.8.382.0_at	691	0.586	0.522	**0.323**
Pf.8.39.0_CDS_at	105	0.885	**0.231**	1.006
Pf.8.40.0_CDS_at	67	0.543	1.357	1.320
Pf.8.40.0_CDS_x_at	51	0.598	1.461	1.349
Pf.8.41.0_CDS_at	26	0.740	0.867	1.300
Pf.8.42.0_CDS_at	9	0.729	1.013	0.571
Pf.8.43.0_CDS_at	1241	**0.464**	0.779	1.109
Pf.8.44.0_CDS_at	348	0.549	0.616	0.787
Pf.8.46.0_CDS_at	4887	**0.079**	**0.067**	**0.025**
Pf.8.47.0_CDS_at	1120	1.200	1.550	1.707
Pf.8.49.0_CDS_at	284	0.503	0.502	**0.156**
Pf.8.5.0_CDS_a_at	2824	**2.118**	1.268	1.275
Pf.8.52.0_CDS_at	313	0.763	0.804	1.663
Pf.8.53.0_CDS_a_at	5178	1.246	0.919	1.038
Pf.8.54.0_CDS_at	553	1.960	0.938	0.640
Pf.8.55.0_CDS_at	235	1.016	1.310	1.539
Pf.8.57.0_CDS_a_at	878	1.090	1.040	1.156
Pf.8.58.0_CDS_at	20	**0.461**	**0.495**	0.731
Pf.8.59.0_CDS_at	418	0.564	**0.336**	**0.411**
Pf.8.6.0_CDS_at	4178	0.988	1.064	1.143
Pf.8.60.0_CDS_at	250	1.294	1.643	1.503
Pf.8.61.0_CDS_at	19	1.079	1.444	1.070
Pf.8.63.0_CDS_a_at	12	0.699	0.747	0.554
Pf.8.64.0_CDS_at	321	**0.434**	**0.204**	**0.109**
Pf.8.65.0_CDS_at	3217	1.488	1.902	1.618
Pf.8.66.0_CDS_at	187	**0.166**	**0.162**	**0.297**
Pf.8.67.0_CDS_at	161	0.719	1.278	1.137
Pf.8.68.0_CDS_at	448	0.707	0.728	1.074
Pf.8.69.0_CDS_at	84	**0.403**	0.691	0.578
Pf.8.69.0_CDS_x_at	53	**0.413**	0.881	0.689
Pf.8.70.0_CDS_a_at	63	1.345	0.541	0.719
Pf.8.71.0_CDS_at	139	**0.290**	0.748	0.802
Pf.8.72.0_CDS_at	2523	**2.018**	1.364	1.602
Pf.8.73.0_CDS_at	482	1.371	0.966	1.155
Pf.8.75.0_CDS_at	42	**0.292**	**0.400**	**0.284**
Pf.8.78.0_CDS_at	607	0.508	0.745	1.179
Pf.8.80.0_CDS_at	127	0.891	0.711	**0.419**
Pf.8.81.0_CDS_at	7	1.367	1.060	1.756
Pf.8.83.0_CDS_at	411	1.675	1.909	1.845
Pf.8.84.0_CDS_at	50	**0.499**	0.957	**0.320**
Pf.8.86.0_CDS_at	320	1.297	1.512	**2.189**
Pf.8.87.0_CDS_at	8	1.087	0.954	0.943
Pf.8.88.0_CDS_at	7	0.885	0.797	0.824
Pf.8.90.0_CDS_at	914	**2.606**	**2.275**	1.537
Pf.8.90.1_CDS_at	11	1.157	1.317	0.788
Pf.8.91.0_CDS_at	279	0.826	0.852	0.554
Pf.8.92.0_CDS_at	37	1.217	1.102	1.918
Pf.8.94.0_CDS_at	230	1.125	1.055	1.615
Pf.8.96.0_CDS_at	919	0.615	0.558	**0.237**
Pf.8.97.0_CDS_at	3082	1.248	1.141	1.186

Pf.8.98.0_CDS_at	46	**14.954**	**2.367**	0.947 #10
Pf.8.99.0_CDS_at	6	1.162	1.018	0.967
Pf.9.10.0_CDS_a_at	385	1.563	1.528	1.418
Pf.9.100.0_CDS_at	15	0.718	1.474	**2.757**
Pf.9.101.0_CDS_at	77	0.695	0.563	0.556
Pf.9.102.0_CDS_at	1816	1.331	1.613	1.136
Pf.9.103.0_CDS_at	11	1.191	0.961	0.610
Pf.9.104.0_CDS_a_at	841	1.558	1.627	**2.248**
Pf.9.105.0_CDS_at	1831	**0.248**	**0.483**	1.217
Pf.9.106.0_CDS_at	236	1.720	**2.120**	0.933
Pf.9.108.0_CDS_a_at	84	**0.453**	0.601	0.755
Pf.9.109.0_CDS_at	354	**0.325**	**0.370**	**0.212**
Pf.9.11.0_CDS_at	948	**0.092**	**0.174**	**0.129**
Pf.9.112.0_CDS_a_at	23	0.917	0.538	**0.452**
Pf.9.113.0_CDS_at	30	1.243	1.135	1.810
Pf.9.114.0_CDS_at	76	**0.137**	**0.226**	**0.172**
Pf.9.115.0_CDS_at	245	**2.118**	**3.470**	**3.675**
Pf.9.117.0_CDS_at	42	**0.405**	0.599	0.659
Pf.9.118.0_CDS_at	99	1.003	1.544	1.317
Pf.9.12.0_CDS_at	11714	1.133	0.836	1.140
Pf.9.120.0_CDS_at	5	1.233	0.967	1.085
Pf.9.122.0_CDS_at	475	**0.318**	1.566	1.002
Pf.9.124.0_CDS_at	87	1.258	1.531	**2.697**
Pf.9.125.0_CDS_at	15	1.477	1.300	0.796
Pf.9.126.0_CDS_at	101	0.568	0.872	0.722
Pf.9.127.0_CDS_at	75	0.699	**0.378**	**0.253**
Pf.9.128.0_CDS_at	65	0.792	0.566	0.628
Pf.9.129.0_CDS_at	1291	**0.301**	**0.494**	**0.313**
Pf.9.13.0_CDS_at	26	0.543	**0.214**	**0.224**
Pf.9.131.0_CDS_at	5	1.042	1.148	1.120
Pf.9.132.0_CDS_at	201	1.844	1.453	1.511
Pf.9.133.0_CDS_at	67	0.566	0.513	**0.370**
Pf.9.134.0_CDS_at	269	0.829	0.607	0.567
Pf.9.135.0_CDS_at	689	**0.287**	0.594	1.067
Pf.9.137.0_CDS_at	293	**0.316**	0.759	1.197
Pf.9.138.0_CDS_at	342	0.573	1.255	1.604
Pf.9.139.0_CDS_at	4505	0.564	0.753	0.903
Pf.9.14.0_CDS_x_at	1347	1.053	0.918	1.308
Pf.9.14.1_a_at	1593	1.149	0.959	1.270
Pf.9.142.0_CDS_at	6	1.255	1.080	1.956
Pf.9.143.0_CDS_at	115	0.647	1.165	1.414
Pf.9.144.0_CDS_a_at	17	1.988	**3.067**	**3.733**
Pf.9.146.0_CDS_at	53	1.213	1.496	1.806
Pf.9.146.0_CDS_x_at	42	0.962	1.326	1.936
Pf.9.147.0_CDS_at	159	0.937	**3.491**	**2.342**
Pf.9.148.0_CDS_at	192	0.769	0.773	0.640
Pf.9.149.0_CDS_at	182	1.149	0.922	0.771
Pf.9.150.0_CDS_at	781	0.918	0.909	1.246
Pf.9.152.0_CDS_at	175	**3.698**	**3.153**	1.228
Pf.9.153.0_CDS_at	114	**0.415**	0.555	0.670
Pf.9.154.0_CDS_at	68	1.179	1.064	1.318
Pf.9.156.0_CDS_at	582	**3.404**	1.031	1.804
Pf.9.158.0_CDS_a_at	436	**0.470**	0.686	0.832
Pf.9.160.0_CDS_at	473	1.898	**2.335**	1.898
Pf.9.163.0_CDS_at	1660	0.802	0.619	1.051
Pf.9.165.0_CDS_at	12	1.600	**0.378**	0.775
Pf.9.167.0_CDS_at	2656	1.656	1.486	1.339
Pf.9.168.0_CDS_at	277	**0.487**	**0.193**	**0.124**
Pf.9.169.0_CDS_at	164	1.470	0.993	1.800
Pf.9.17.0_CDS_at	368	**0.475**	0.696	0.790
Pf.9.170.0_CDS_at	552	0.555	0.732	0.578
Pf.9.171.0_CDS_at	37	0.613	**0.483**	0.660
Pf.9.172.0_CDS_at	368	1.285	0.806	1.036

Pf.9.173.0_CDS_at	600	**0.276**	**0.450**	0.743
Pf.9.174.0_CDS_at	1674	1.784	**0.463**	1.932
Pf.9.175.0_CDS_at	29	**2.680**	**0.464**	0.537
Pf.9.176.0_CDS_at	274	1.088	1.341	0.778
Pf.9.177.0_CDS_at	2392	**2.051**	**2.645**	1.582
Pf.9.178.0_CDS_at	14	0.910	1.000	0.802
Pf.9.179.0_CDS_at	62	1.148	1.095	1.338
Pf.9.18.0_CDS_at	11	0.910	0.888	0.870
Pf.9.181.0_CDS_at	11	0.582	0.669	0.623
Pf.9.182.0_CDS_at	236	0.756	**0.385**	**0.275**
Pf.9.183.0_CDS_at	3784	0.768	1.002	0.885
Pf.9.184.0_CDS_at	436	**2.494**	1.922	**2.675**
Pf.9.185.0_CDS_at	88	0.635	**0.365**	0.944
Pf.9.186.0_CDS_at	769	1.823	1.830	1.454
Pf.9.188.0_CDS_s_at	235	1.581	0.532	**0.392**
Pf.9.188.0_CDS_x_at	7	1.010	0.994	1.016
Pf.9.189.0_CDS_s_at	39	1.969	**0.283**	**0.358**
Pf.9.189.0_CDS_x_at	33	**0.430**	**0.397**	**0.284**
Pf.9.19.0_CDS_at	68	**0.319**	0.586	**0.345**
Pf.9.190.0_CDS_at	5	1.287	1.118	1.184
Pf.9.191.0_CDS_at	6	0.845	0.930	0.984
Pf.9.192.0_CDS_at	7	0.880	0.891	0.793
Pf.9.193.0_CDS_at	6	1.090	0.991	1.117
Pf.9.195.0_CDS_at	158	1.457	0.647	**0.380**
Pf.9.196.0_CDS_at	881	0.577	1.139	**2.052**
Pf.9.196.0_CDS_x_at	622	0.615	1.180	**2.688**
Pf.9.197.0_CDS_at	8	0.846	1.108	2.173
Pf.9.198.0_CDS_at	5	1.575	1.123	0.958
Pf.9.199.0_CDS_at	9	0.887	1.453	1.928
Pf.9.2.0_CDS_at	10710	0.780	0.791	0.859
Pf.9.20.0_CDS_at	5544	1.146	1.136	1.251
Pf.9.200.0_CDS_at	186	**2.330**	**2.294**	**2.945**
Pf.9.201.0_CDS_at	370	**2.471**	**2.592**	**2.665**
Pf.9.202.0_CDS_at	27	0.620	**0.411**	**0.278**
Pf.9.203.0_CDS_at	524	1.049	0.781	**2.082**
Pf.9.204.1_a_at	8	0.980	0.623	0.550
Pf.9.205.0_CDS_at	2093	1.242	1.920	1.455
Pf.9.206.0_CDS_at	12	1.981	**2.172**	**2.293**
Pf.9.207.0_CDS_at	779	0.814	1.126	1.836
Pf.9.208.0_CDS_at	877	0.601	0.836	1.124
Pf.9.209.0_CDS_a_at	256	0.823	0.577	1.921
Pf.9.21.0_CDS_at	241	**0.116**	**0.293**	**0.403**
Pf.9.210.0_CDS_at	9	0.945	0.915	1.118
Pf.9.211.0_CDS_a_at	614	**0.222**	**0.291**	**0.307**
Pf.9.213.0_CDS_at	13	1.157	0.508	1.050
Pf.9.214.0_CDS_at	35	0.644	1.036	0.960
Pf.9.215.0_CDS_at	451	0.985	1.183	1.595
Pf.9.219.0_CDS_at	34	1.538	0.664	1.425
Pf.9.22.0_CDS_at	22	**0.327**	0.771	1.113
Pf.9.221.0_CDS_at	13	1.231	1.166	1.271
Pf.9.222.0_CDS_at	25	**2.058**	0.556	0.714
Pf.9.224.0_CDS_at	6	1.057	0.954	0.981
Pf.9.225.0_CDS_at	263	1.580	1.069	1.186
Pf.9.226.0_CDS_at	16	0.824	**0.425**	0.508
Pf.9.228.0_CDS_at	365	1.108	**0.372**	**0.112**
Pf.9.229.0_CDS_at	55	0.966	**0.203**	0.621
Pf.9.23.0_CDS_at	1045	**0.177**	**0.100**	**0.067**
Pf.9.230.0_CDS_at	17	**0.454**	1.784	0.512
Pf.9.231.0_CDS_at	9	1.018	0.825	0.909
Pf.9.233.0_CDS_at	8	1.270	0.846	0.884
Pf.9.235.0_CDS_at	207	0.602	1.025	1.279
Pf.9.237.0_CDS_at	12	0.889	0.962	0.983

Pf.9.238.1_a_at	8	0.806	0.872	0.940
Pf.9.238.1_at	26	0.803	0.831	0.704
Pf.9.239.0_CDS_at	9	1.071	0.877	1.165
Pf.9.24.0_CDS_at	42	0.586	**0.403**	**0.382**
Pf.9.240.0_CDS_at	1249	1.585	1.528	**2.311**
Pf.9.241.0_CDS_at	19	1.946	1.585	**2.381**
Pf.9.243.0_CDS_at	374	0.654	0.926	1.293
Pf.9.244.0_CDS_at	7	1.615	0.941	0.933
Pf.9.245.1_a_at	6	1.107	0.629	0.924
Pf.9.246.0_CDS_at	13	1.824	**2.044**	1.755
Pf.9.247.0_CDS_at	29	0.883	0.757	0.833
Pf.9.248.0_CDS_at	26	1.717	1.078	**0.357**
Pf.9.249.0_CDS_at	594	0.540	**0.423**	**0.129**
Pf.9.25.0_CDS_at	29	1.068	**0.296**	**0.493**
Pf.9.250.0_CDS_at	188	1.218	0.573	0.710
Pf.9.251.0_CDS_at	46	1.876	**2.054**	**3.550**
Pf.9.252.0_CDS_at	635	1.188	1.697	**2.070**
Pf.9.253.0_CDS_at	1086	0.919	1.170	1.555
Pf.9.254.0_CDS_at	84	1.414	1.170	0.889
Pf.9.255.0_CDS_at	735	1.835	1.404	1.749
Pf.9.256.0_CDS_at	33	0.883	1.185	**2.490**
Pf.9.258.0_CDS_at	40	**8.094**	**12.926**	1.561
Pf.9.26.0_CDS_a_at	120	0.971	**0.478**	0.971
Pf.9.260.0_CDS_at	8	1.463	0.728	0.718
Pf.9.261.0_CDS_at	85	0.587	**0.187**	**0.225**
Pf.9.262.0_CDS_at	21	1.359	0.937	0.812
Pf.9.262.1_a_at	39	**2.313**	**2.683**	1.030
Pf.9.263.0_CDS_at	244	0.634	1.398	1.812
Pf.9.264.0_CDS_at	19	**0.459**	0.721	1.291
Pf.9.265.0_CDS_at	10	2.004	0.655	1.131
Pf.9.266.0_CDS_at	392	0.919	0.634	0.832
Pf.9.267.0_CDS_at	517	**6.084**	**2.579**	0.984 #11
Pf.9.268.0_CDS_at	24	1.110	1.548	**4.417**
Pf.9.269.0_CDS_at	206	**3.791**	**4.943**	**2.188**
Pf.9.27.0_CDS_at	2189	0.907	0.755	0.982
Pf.9.270.0_CDS_at	9	**4.572**	1.272	0.850
Pf.9.272.0_CDS_at	6	0.802	0.828	0.831
Pf.9.273.0_CDS_at	6	0.765	0.906	0.828
Pf.9.274.0_CDS_at	5	0.923	0.960	1.050
Pf.9.275.0_CDS_at	6	0.977	0.790	0.980
Pf.9.276.0_CDS_at	27	1.646	0.813	0.628
Pf.9.276.0_CDS_x_at	113	1.826	0.681	**0.489**
Pf.9.277.0_CDS_s_at	8	1.138	0.911	0.690
Pf.9.279.0_CDS_at	6	1.026	1.000	0.975
Pf.9.280.0_CDS_at	5	1.083	1.018	1.028
Pf.9.281.0_CDS_at	5	1.001	0.909	1.008
Pf.9.282.0_CDS_at	6	1.099	0.937	0.897
Pf.9.283.0_CDS_at	6	0.902	0.902	0.912
Pf.9.285.0_CDS_at	6	0.986	0.983	0.975
Pf.9.286.0_CDS_at	6	1.052	0.846	0.915
Pf.9.289.0_CDS_at	371	0.727	**0.493**	1.088
Pf.9.29.0_CDS_at	22	0.574	**0.441**	**0.376**
Pf.9.290.0_CDS_at	13	1.689	0.855	1.636
Pf.9.291.0_CDS_at	117	0.605	0.692	1.391
Pf.9.292.0_CDS_at	23	**0.423**	**0.300**	**0.237**
Pf.9.293.0_CDS_at	27	**2.332**	0.878	1.058
Pf.9.294.0_CDS_at	58	1.303	0.901	**0.477**
Pf.9.295.0_CDS_at	11	1.214	0.912	1.094
Pf.9.296.0_CDS_at	62	**3.297**	**3.101**	**3.112**
Pf.9.297.0_CDS_at	483	**0.255**	0.668	1.465
Pf.9.298.0_CDS_at	777	1.479	1.148	1.315
Pf.9.299.0_CDS_at	6	1.150	1.011	0.990
Pf.9.299.0_CDS_x_at	6	1.169	1.041	1.154

Pf.9.3.0_CDS_at	22	1.343	0.953	0.553
Pf.9.30.0_CDS_at	8	1.079	0.714	1.101
Pf.9.300.0_CDS_at	25	1.068	**0.394**	0.714
Pf.9.301.0_CDS_at	965	**0.464**	**0.288**	**0.177**
Pf.9.302.0_CDS_at	732	**0.075**	**0.220**	**0.355**
Pf.9.303.0_CDS_at	56	1.426	**0.256**	**0.447**
Pf.9.304.0_CDS_at	141	0.885	1.777	1.554
Pf.9.305.0_CDS_at	5	**4.526**	1.505	1.275
Pf.9.306.0_CDS_at	85	0.887	0.955	1.189
Pf.9.307.0_CDS_at	30	1.215	1.313	**0.372**
Pf.9.308.0_CDS_at	12	1.128	**0.490**	0.632
Pf.9.309.0_CDS_at	2087	**0.074**	**0.089**	**0.305**
Pf.9.31.0_CDS_at	46	**2.460**	1.306	1.769
Pf.9.310.0_CDS_at	9	1.447	**2.288**	**4.555**
Pf.9.311.0_CDS_at	60	0.848	0.969	1.599
Pf.9.313.0_CDS_at	9	1.153	0.723	0.774
Pf.9.314.0_CDS_at	11	0.839	0.799	0.572
Pf.9.315.0_CDS_at	16	1.116	0.550	0.857
Pf.9.316.0_CDS_at	61	0.520	0.560	0.260
Pf.9.317.0_CDS_a_at	899	1.711	1.780	1.572
Pf.9.318.0_CDS_at	45	1.406	2.193	2.730
Pf.9.32.0_CDS_at	1722	0.160	0.287	0.128
Pf.9.321.0_CDS_at	9	2.792	0.689	0.716
Pf.9.322.0_CDS_at	1700	0.177	0.294	0.509
Pf.9.323.0_CDS_at	6	1.743	**2.627**	3.542
Pf.9.324.0_CDS_at	179	1.264	1.654	3.545
Pf.9.325.0_CDS_at	92	0.783	0.568	2.866
Pf.9.326.0_CDS_at	99	0.789	0.952	1.743
Pf.9.328.0_CDS_at	58	1.775	1.374	3.906
Pf.9.329.0_CDS_at	190	0.729	0.171	0.130
Pf.9.33.0_CDS_at	22	0.578	**0.215**	0.550
Pf.9.330.0_CDS_at	300	0.286	0.624	0.793
Pf.9.331.0_CDS_at	44	1.240	0.740	1.441
Pf.9.332.0_CDS_at	450	0.809	1.218	2.129
Pf.9.333.0_CDS_at	24	1.649	2.155	1.999
Pf.9.334.0_CDS_at	1047	0.509	0.482	0.650
Pf.9.335.0_CDS_at	501	3.159	3.628	2.885
Pf.9.336.0_CDS_at	32	0.853	0.643	1.178
Pf.9.337.0_CDS_at	189	1.189	0.944	2.523
Pf.9.338.0_CDS_at	905	1.614	1.435	1.667
Pf.9.339.0_CDS_at	177	0.547	1.584	1.914
Pf.9.34.0_CDS_at	759	0.636	0.940	0.818
Pf.9.340.0_CDS_at	57	1.646	0.662	0.820
Pf.9.341.0_CDS_at	76	0.964	0.820	0.921
Pf.9.343.0_CDS_at	1183	1.898	2.010	1.585
Pf.9.345.0_CDS_at	7	0.948	0.808	0.861
Pf.9.346.0_CDS_at	227	0.748	1.307	1.037
Pf.9.347.0_CDS_at	20	2.166	2.177	1.060
Pf.9.35.0_CDS_at	770	0.165	0.339	0.281
Pf.9.350.0_CDS_at	155	0.618	0.830	0.926
Pf.9.351.0_CDS_at	41	**0.352**	**0.330**	0.603
Pf.9.352.0_CDS_at	35	0.626	0.889	0.692
Pf.9.353.0_CDS_at	30	0.657	0.620	0.693
Pf.9.355.0_CDS_at	135	0.649	0.662	0.791
Pf.9.357.0_CDS_at	1829	0.527	0.973	1.059
Pf.9.358.0_CDS_at	398	**2.316**	0.653	0.993
Pf.9.359.0_CDS_a_at	41	**3.381**	**2.466**	**2.604**
Pf.9.36.0_CDS_at	36	0.833	0.592	**0.207**
Pf.9.360.0_CDS_at	859	1.120	1.776	1.963
Pf.9.361.0_CDS_at	2050	**4.736**	1.922	1.920
Pf.9.363.0_CDS_at	7	1.030	1.069	1.153
Pf.9.364.0_CDS_at	8	0.891	0.878	0.891

Pf.9.365.0_CDS_x_at	2352	1.192	0.658	0.615
Pf.9.368.0_at	10	0.972	0.783	0.699
Pf.9.374.0_a_at	7	0.847	1.044	0.936
Pf.9.38.0_CDS_at	298	**0.229**	1.025	0.552
Pf.9.39.0_CDS_at	57	**3.463**	0.849	**0.218**
Pf.9.4.0_CDS_at	5525	0.884	0.945	0.836
Pf.9.40.0_CDS_at	441	0.780	1.313	1.769
Pf.9.400.0_at	130	0.743	0.730	1.281
Pf.9.41.0_CDS_at	75	**0.197**	**0.141**	**0.437**
Pf.9.41.0_CDS_x_at	241	**0.153**	**0.131**	**0.305**
Pf.9.415.0_at	310	**2.116**	1.238	0.949
Pf.9.42.0_CDS_at	2653	1.422	1.432	1.156
Pf.9.43.0_CDS_at	44	0.932	0.524	0.987
Pf.9.43.0_CDS_x_at	34	0.907	0.544	1.130
Pf.9.433.0_at	136	1.906	**0.363**	**0.432**
Pf.9.436.0_at	10	1.267	0.926	1.242
Pf.9.44.0_CDS_at	195	**2.016**	**2.289**	**2.350**
Pf.9.45.0_CDS_at	107	0.579	1.218	1.103
Pf.9.47.0_CDS_at	26	1.188	1.317	1.675
Pf.9.47.0_CDS_x_at	54	1.230	1.631	1.498
Pf.9.472.0_at	14042	**0.055**	**0.079**	**0.023**
Pf.9.485.0_at	120	0.863	1.299	1.213
Pf.9.486.0_at	1107	1.979	1.181	1.482
Pf.9.49.0_CDS_at	6344	0.721	1.074	1.165
Pf.9.5.0_CDS_a_at	10847	**0.050**	**0.094**	**0.027**
Pf.9.50.1_CDS_at	527	1.354	1.295	**0.538**
Pf.9.51.0_CDS_at	740	**0.330**	1.021	**0.407**
Pf.9.52.0_CDS_at	1354	0.790	0.801	0.683
Pf.9.53.0_CDS_at	27	**0.417**	**0.344**	0.885
Pf.9.54.0_CDS_at	220	**2.505**	**2.018**	1.699
Pf.9.55.0_CDS_at	380	**0.207**	**0.281**	**0.142**
Pf.9.56.0_CDS_at	60	1.116	0.705	1.797
Pf.9.57.0_CDS_at	332	**0.556**	0.907	**0.262**
Pf.9.58.0_CDS_at	76	1.068	0.887	1.084
Pf.9.59.0_CDS_at	35	**0.317**	**0.319**	**0.293**
Pf.9.6.0_CDS_a_at	6	0.974	0.970	0.895
Pf.9.6.0_CDS_s_at	6079	**0.037**	**0.128**	**0.050**
Pf.9.6.1_CDS_a_at	5	0.946	1.013	1.012
Pf.9.6.2_CDS_s_at	971	**0.110**	**0.248**	**0.132**
Pf.9.60.0_CDS_at	1646	1.655	1.435	1.806
Pf.9.61.0_CDS_at	40	1.382	0.960	1.457
Pf.9.62.0_CDS_at	473	0.833	1.167	1.303
Pf.9.63.0_CDS_at	129	1.638	**2.529**	1.525
Pf.9.65.0_CDS_at	53	**0.360**	0.584	0.613
Pf.9.68.0_CDS_at	72	**0.132**	**0.072**	**0.074**
Pf.9.69.0_CDS_at	231	1.036	**0.484**	0.566
Pf.9.7.0_CDS_at	9158	**0.166**	**0.201**	**0.079**
Pf.9.70.0_CDS_at	43	**2.296**	1.326	1.277
Pf.9.71.0_CDS_at	7	0.871	0.669	0.546
Pf.9.72.0_CDS_a_at	15	0.706	0.616	0.930
Pf.9.74.0_CDS_at	3259	**0.154**	**0.340**	**0.193**
Pf.9.75.0_CDS_at	1289	0.615	0.609	0.997
Pf.9.76.0_CDS_at	1164	**0.392**	0.645	0.843
Pf.9.78.0_CDS_at	28	0.923	0.542	0.660
Pf.9.79.0_CDS_at	802	0.954	0.983	1.552
Pf.9.8.0_CDS_at	524	**2.444**	1.806	**0.490**
Pf.9.80.0_CDS_at	872	1.332	1.283	1.633
Pf.9.81.0_CDS_at	663	0.942	0.764	1.082
Pf.9.82.0_CDS_at	65	0.685	0.694	**0.483**
Pf.9.83.0_CDS_at	81	**0.459**	0.738	0.797
Pf.9.84.0_CDS_at	1314	**0.318**	0.517	0.685
Pf.9.85.0_CDS_at	3144	0.809	1.144	1.251

Pf.9.86.0_CDS_at	1309	0.945	1.059	1.354
Pf.9.87.0_CDS_at	398	**2.498**	**2.604**	1.279
Pf.9.88.0_CDS_at	33	0.691	0.592	0.696
Pf.9.89.0_CDS_at	1498	1.281	1.549	1.568
Pf.9.91.0_CDS_at	290	**0.277**	**0.287**	1.305
Pf.9.93.0_CDS_at	692	**2.241**	**2.234**	1.750
Pf.9.94.0_CDS_at	5	1.317	1.171	1.087
Pf.9.95.0_CDS_at	1312	**4.320**	**2.503**	1.479
Pf.9.96.0_CDS_at	363	**0.102**	**0.265**	**0.479**
Pf.9.98.1_a_at	54	**0.487**	**0.425**	**0.333**
Pf.9.99.1_a_at	14	0.897	**0.373**	0.507
Pf.UN_1.3.0_CDS_at	24	1.427	1.387	1.237
Pf.UN_1.4.0_CDS_at	78	**2.357**	0.904	0.962
Pf.UN_2.5.0_CDS_at	25	1.059	1.380	1.091
Pf.UN_3.6.0_CDS_at	73	**4.105**	1.959	1.610
Pf.UN_4.1.0_CDS_at	208	0.643	**0.374**	**0.354**
Pf.UN_4.10.0_CDS_at	53	1.420	**0.202**	**0.333**
Pf.UN_4.11.0_CDS_x_at	8	1.119	0.779	0.814
Pf.UN_4.2.0_CDS_at	86	1.678	**0.495**	**8.540**
Pf.UN_4.7.0_CDS_at	65	0.997	**0.323**	1.660
Pf.UN_4.8.0_CDS_at	109	**3.899**	0.620	0.645
Pf.UN_4.9.0_CDS_x_at	8	1.270	0.648	0.601
Pfa3D7_hrpII_at	6	1.230	0.735	0.864
Pfa3D7_hrpII_x_at	6	1.049	0.882	1.136
Pfa3D7_hrpIII_at	53	1.660	**0.371**	0.726
Pfa3D7_hrpIII_x_at	50	1.599	**0.423**	0.674
U09839.1_s_at	6	2.166	1.418	1.502
U27339.1_at	6	1.236	0.951	0.934
U60602.1_at	6	0.949	0.937	1.085
U60603.1_x_at	12	0.895	0.437	0.402
U82507.1_RC_x_at	11	0.856	0.637	0.753
U82507.1_x_at	6	1.123	1.061	0.833
U82508.1_s_at	6	0.977	1.040	1.071
U82509.1_x_at	6	0.973	0.945	1.074
X00245.1_RC_x_at	8	0.815	0.834	0.852
X00245.1_x_at	5	1.052	0.941	1.000
X02406.1_RC_s_at	1450	0.642	0.841	**0.398**
X02406.1_s_at	29053	**0.171**	**0.456**	**0.174**
X03144.1_at	1727	**2.628**	0.952	0.781
X13014.1_RC_at	343	0.905	1.161	1.644
X15063.1_RC_at	132	1.098	**0.081**	**0.195**
X15063.1_RC_x_at	123	0.840	**0.098**	**0.207**
X17483.1_s_at	3233	1.192	1.556	1.235
X17484.1_s_at	161	**2.172**	**2.555**	**2.379**
X17486.1_RC_at	7	0.920	0.983	1.427
X17486.1_s_at	8	1.084	1.345	1.942
X71408.1_s_at	5129	1.524	0.938	1.307
X71409.1_at	6	0.988	0.944	1.144
X87840.1_s_at	5	1.119	0.999	0.953
Y09693.1_RC_at	74	1.495	1.791	0.841
Y09693.1_s_at	485	0.576	0.961	0.528
Y13404.1_at	6	1.054	0.939	1.103
Y13404.1_RC_at	10	1.035	0.656	0.769
Y13404.1_RC_x_at	13	1.189	0.542	0.656
Y13404.1_x_at	9	0.897	0.709	0.748
Y13405.1_at	26	**2.895**	0.509	0.520
Y13405.1_RC_at	59	**2.817**	**0.412**	1.507
Y13405.1_s_at	5	1.153	0.934	0.931
Y13405.1_x_at	23	**3.069**	0.562	0.576
Y13406.1_RC_at	5	1.153	1.035	1.271
Y13406.1_s_at	7	**5.034**	**11.447**	1.483
Y13406.1_x_at	6	0.959	0.963	0.901
Y13407.1_at	7	0.938	1.166	0.959

Y13407.1_RC_at	8	1.087	1.236	0.789
Y13408.1_at	7	1.146	**3.172**	1.851

Fold changes in bold font are >2.0 or <0.5.

covered with gray	signal <21
#1–#11	selected
