# Supplementary material for: Profiling molecular factors associated with pyknosis and developmental arrest induced by an opioid receptor antagonist and dihydroartemisinin in Plasmodium falciparum
Source: PLoS One. 2017 Sep 21;12(9):e0184874. doi: 10.1371/journal.pone.0184874 (PMC5608265; doi:10.1371/journal.pone.0184874)
Supplement: S2 Table — (DOC) [file pone.0184874.s002.doc]

**S2**. Gene IDs and target description of the selected transcripts

|  |  |  |  |
| --- | --- | --- | --- |
| selected | ProbeSetID | Pfdb(a) | Target description |
| #1 | Pf.10.211.0_CDS_at | PF3D7_1037100 | pyruvate kinase 2, putative |
| #2 | Pf.13_1.278.0_CDS_at | PF3D7_1355600 | conserved Plasmodium protein, unknown function |
| #3 | Pf.13_1.316.0_CDS_at | PF3D7_1331300 | signal peptidase 21 kDa subunit |
| #4 | Pf.14.38.0_CDS_at | PF3D7_1419800.2 | glutathione reductase |
| #5 | Pf.14.380.0_CDS_at | PF3D7_1408000 | plasmepsin II |
| #6 | Pf.14.538.0_CDS_at | PF3D7_1472300 | conserved Plasmodium membrane protein, unknown function |
| #7 | Pf.14.68.0_CDS_at | PF3D7_1448400 | ubiquitin-protein ligase, putative (HRD3) |
| #8 | Pf.5.187.0_CDS_at | PF3D7_0508300 | triose phosphate transporter (PfoTPT) |
| #9 | Pf.8.126.0_CDS_at | PF3D7_0828200 | leucine-tRNA ligase |
| #10 | Pf.8.98.0_CDS_at | PF3D7_0830500 | tryptophan/threonine-rich antigen |
| #11 | Pf.9.267.0_CDS_at | PF3D7_0935600 | gametocyte-implicated protein (Fragment) |

(a) *P. falciparum* genomic database
